# The Postcranial Skeleton of an Exceptionally Complete Individual of the Plated Dinosaur *Stegosaurus stenops* (Dinosauria: Thyreophora) from the Upper Jurassic Morrison Formation of Wyoming, U.S.A.

**DOI:** 10.1371/journal.pone.0138352

**Published:** 2015-10-14

**Authors:** Susannah Catherine Rose Maidment, Charlotte Brassey, Paul Michael Barrett

**Affiliations:** 1 Department of Earth Science and Engineering, Imperial College, South Kensington Campus, London SW7 2AZ, United Kingdom; 2 Department of Earth Sciences, The Natural History Museum, Cromwell Road, London SW7 5BD, United Kingdom; Raymond M. Alf Museum of Paleontology, UNITED STATES

## Abstract

Although *Stegosaurus* is one of the most iconic dinosaurs, well-preserved fossils are rare and as a consequence there is still much that remains unknown about the taxon. A new, exceptionally complete individual affords the opportunity to describe the anatomy of *Stegosaurus* in detail for the first time in over a century, and enables additional comparisons with other stegosaurian dinosaurs. The new specimen is from the Red Canyon Ranch Quarry, near Shell Wyoming, and appears to have been so well preserved because it was buried rapidly in a pond or body of standing water immediately after death. The quarry is probably located in the middle part of the Morrison Formation, which is believed to be Tithonian in age in this area. The specimen is referable to *Stegosaurus stenops* based on the possession of an edentulous anterior portion of the dentary and elevated postzygapophyses on the cervical vertebrae. New information provided by the specimen concerns the morphology of the vertebrae, the iliosacral block and dermal armor. Several aspects of its morphology indicate the individual was not fully skeletally mature at the time of death, corroborating a previous histological study.

## Introduction

*Stegosaurus* is the best-known member of the clade of armored dinosaurs (thyreophorans) known as Stegosauria, or the plated dinosaurs. The stegosaurs are characterized by the possession of two parasagittal rows of hypertrophied dermal armorplates and/or spines extending from the neck to the end of the tail and range from Middle Jurassic to Early Cretaceous in age [1]. Stegosaurian remains have been reported from all continents except Australia and Antarctica [1].

Despite its status as one of the most iconic and easily recognizable dinosaurs, well-preserved remains of *Stegosaurus* are surprisingly rare. Most *Stegosaurus* specimens comprise either isolated elements or associated and/or articulated partial skeletons that are composed of less than half of the skeleton (SCRM and PMB pers. obs. 2004–2014). A few more complete individuals are known (e.g. USNM 4934; DMNH 2818); however, these are ‘road kill’ specimens, in which either many of the bones are crushed flat (and in some cases it is impossible to distinguish between individual elements) or the individual elements have been prepared and preserved in such a way that they are obscured in all but one or two standard views. As a result of these factors, *Stegosaurus* remains relatively understudied by the standards of other Upper Jurassic dinosaur taxa, and there is still much about its anatomy and palaeobiology that remains unknown. Indeed, the last detailed description of the taxon was given in 1914 [2] and this remains the definitive description of *Stegosaurus* to this day.

**Fig 1:**
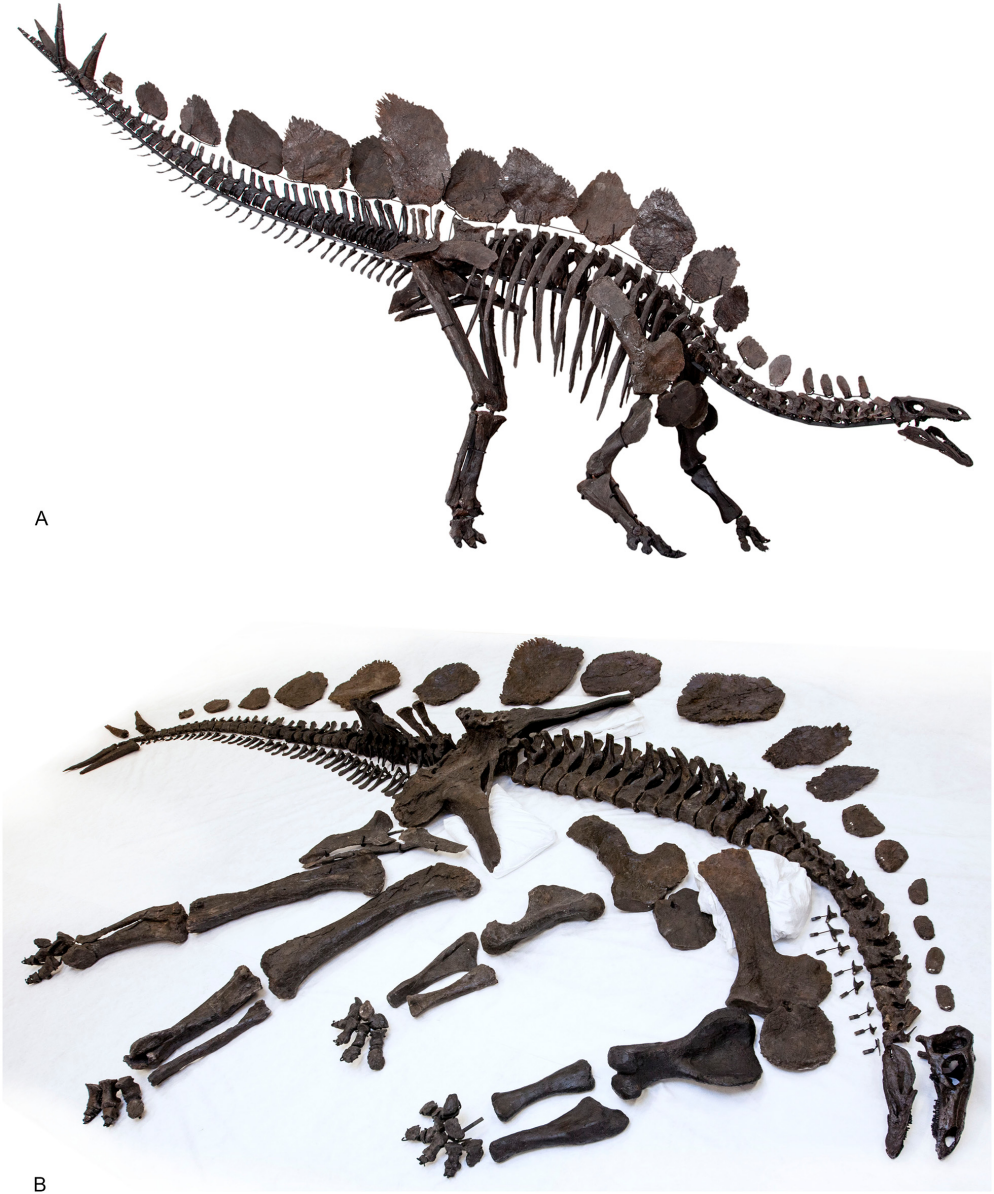
**A**, mounted skeleton in right lateral view and **B**, laid out with missing elements reconstructed before mounting. Images copyright The Natural History Museum.

The discovery, therefore, of an essentially complete, relatively well-preserved individual of *Stegosaurus stenops* (NHMUK PV R36730; Fig 1) from the Red Canyon Ranch quarry, near Shell, Wyoming, U.S.A. in 2003, affords the opportunity to re-examine the anatomy of *Stegosaurus* in the light of a century’s worth of palaeontological discovery. When [2] was writing about *Stegosaurus*, only two other taxa, *Dacentrurus* (then referred to as *Omosaurus*) and *Loricatosaurus* (then known as ‘*Stegosaurus priscus*’) were known. By contrast, subsequent discoveries in Africa, Europe, and especially China, have led to the recognition of a further 11 stegosaur genera. The purpose of this paper is to provide a new, detailed description of the postcranial anatomy of *Stegosaurus stenops* based on NHMUK PV R36730, and to compare it with all other stegosaur genera. We also provide detailed illustrations and measurements of all skeletal elements and a three-dimensional model of the specimen (SI 1). A full description of the skull will be provided elsewhere.

## Discovery of the Specimen

**Fig 2:**
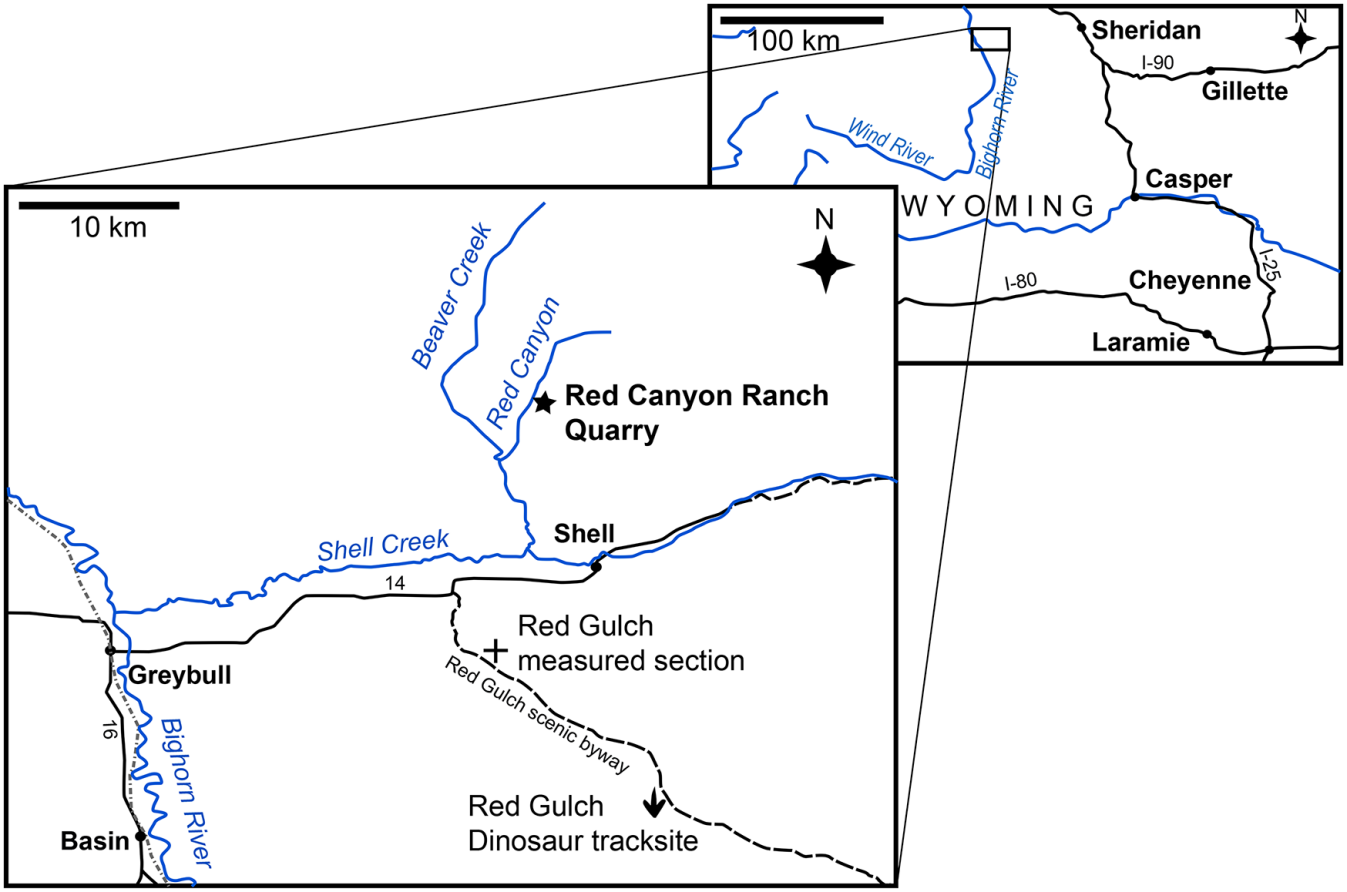
Maps showing the location of the Red Canyon Ranch quarry in Wyoming. The Howe and Howe-Stephens quarries are located 41 km to the WNW of the Red Canyon Ranch quarry.

**Fig 3:**
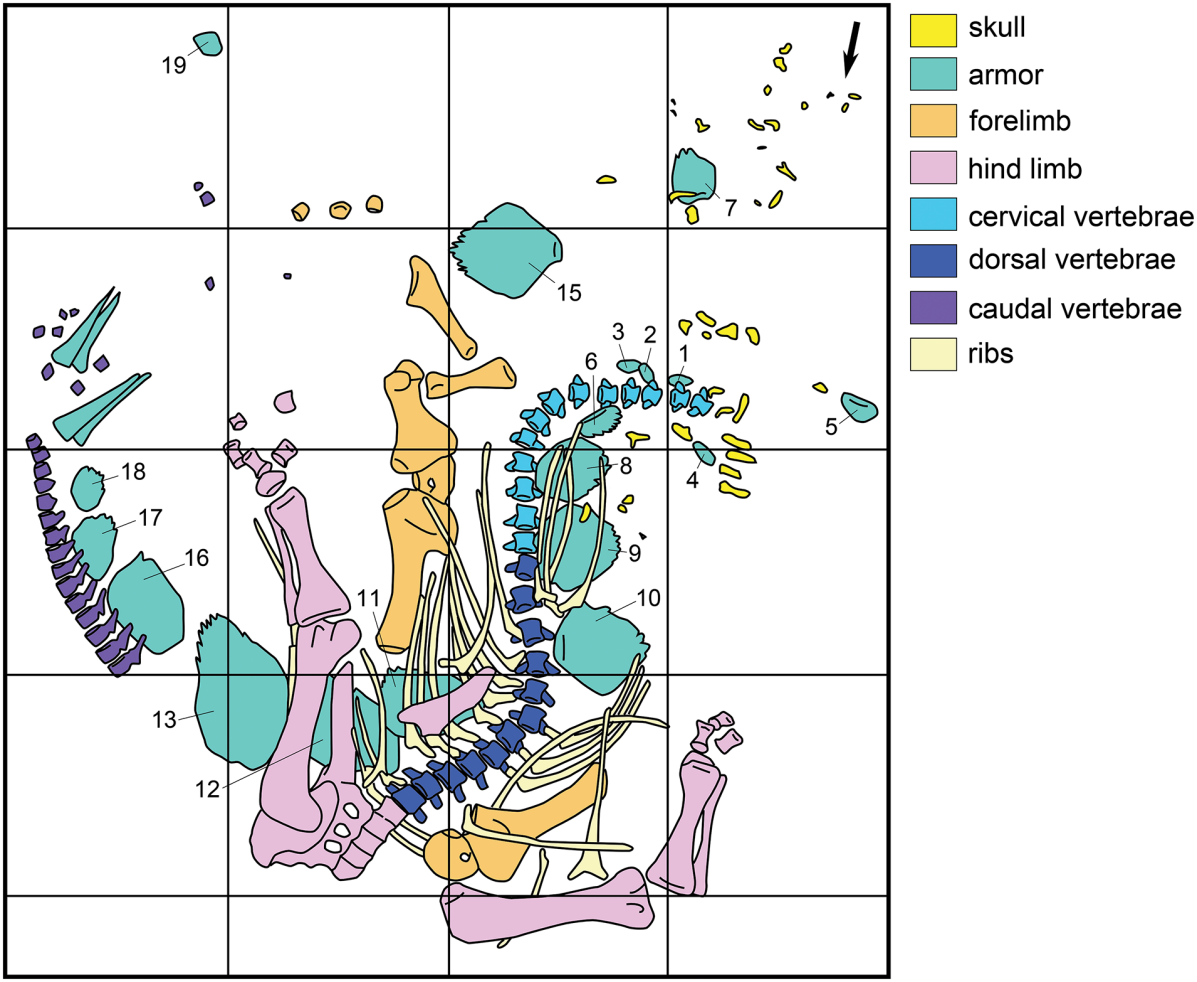
Quarry map drawn during excavation of NHMUK PV R36730. The arrow in the top right hand corner points towards north; squares are 1x1 m; numbers refer to plates in the order described below.

NHMUK PV R36730 was excavated from the Morrison Formation of the Red Canyon Ranch quarry, near Shell, Wyoming, NE1/4 NW1/4 S28, T54NR91W; N44°37.952’ W107°48.868’ (Fig 2). The specimen was discovered in 2003 by Bob Simon who, late one evening during a severe windstorm, was moving a bulldozer and accidentally grazed the side of a hill. The next morning, Mr. Simon noted bones at the site where the bulldozer had accidentally removed some weathered material, and started to excavate what he later recognized as the base of a tail. Poor weather prevented more extensive exploration of the discovery that year and the remainder of the specimen was excavated in 2004 by Mr Simon, his staff, and Kirby Siber and colleagues from the Sauriermuseum, Aathal, Switzerland, where the specimen was later prepared. The quarry map drawn during excavation of the specimen is reproduced in Fig 3. While at the Sauriermuseum, the specimen was known by the specimen number SMA RCR0603. No permits were required for the described study, which complied with all relevant regulations, as the fossil was found on private land.

## Geological Setting

Unfortunately, the sediments encasing NHMUK PV R36730 were removed entirely during the excavation of the specimen and other dinosaur specimens that were found nearby. It is not possible to trace horizons laterally close to the quarry for both this reason and the presence of a fault that trends NE-SW immediately southeast of the discovery site, which appears to downthrow sediments to the northwest of it by at least 3 m. Sediments immediately below the discovery site comprise 60 cm of drab olive-green claystone overlain by 15 cm of finely laminated, very fine-grained sands grading upwards to olive-green siltstones and finally 2 cm of white, fine, quartz-rich sand containing mm-scale iron concretions. The sediment around the specimen contained large numbers of freshwater bivalves of the species *Unio lapilloides* [3]. The bivalves represent a death assemblage as their valves are open, however the valves remain articulated, indicating rapid burial.

Cyclic layers of mudclast conglomerates fining to ripple cross-laminated fine-grained calcareous sand and eventually black claystone about 2.5m in thickness are present about 10 m to the north of the discovery site, at approximately the same stratigraphic level. Organic material and iron concretions are abundant in these beds. The proportion of conglomerate to sand decreases upwards through the sequence, with the top of the section being dominated by very fine grained, white, ripple cross-laminated calcareous sand with iron concretions. NHMUK PV R36730 was not found within a mudclast conglomerate, however, so these beds must be laterally restricted and did not extend to the quarry site.

Prior to excavation, the postcranial elements were in either full or semi-articulation and none had been transported for any distance from the rest of the carcass (Fig 3), suggesting that burial occurred before substantial decay or scavenging could occur. By contrast, the cranial elements were disarticulated and scattered within a small area. This, in combination with some movement of the pectoral girdles and forelimb elements suggests that some elements were potentially moved from their original skeletal positions by gentle hydraulic action before final burial.

The presence of freshwater bivalves, the almost fully articulated and highly complete state of the skeleton, and the presence of fine-grained deposits underlying the quarry site (see above) suggest rapid burial in a body of standing water. High-energy deposits lateral to the quarry site are suggestive of crevasse-splay deposits and indicate proximity to a river channel; thus the animal may have died in a pond or oxbow lake.

**Fig 4:**
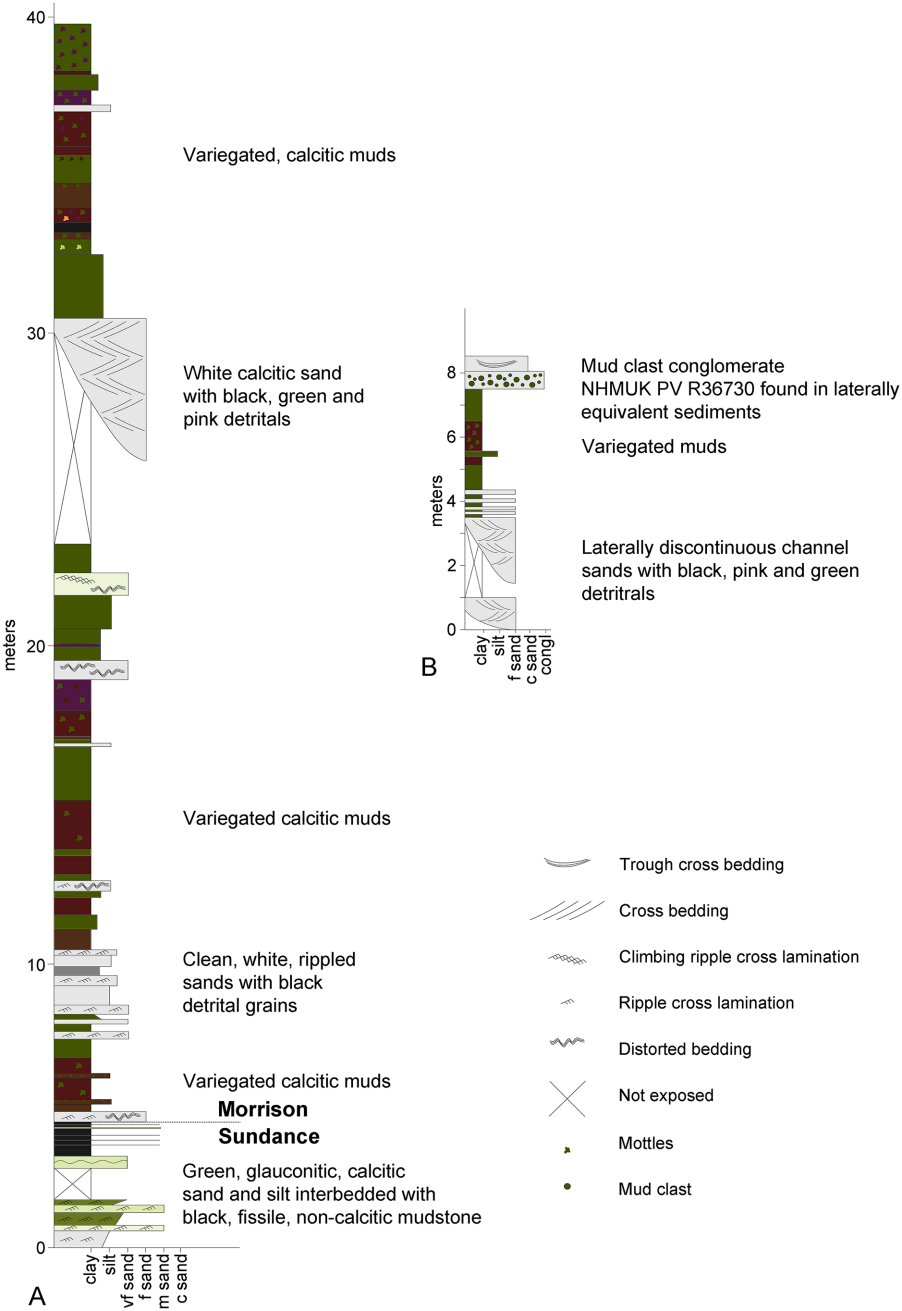
Geological setting of the Red Canyon Ranch quarry. **A**, sedimentary log of a measured section of the Morrison Formation at Red Gulch (for location see Fig 1); **B**, composite sketch log of the Morrison Formation exposed at the Red Canyon Ranch quarry. NHMUK R36730 was found in fine-grained sediment laterally equivalent to the mud clast conglomerate. **c**, coarse; **congl**, conglomerate; **f**, fine; **m**, medium; **vf**, very fine.

The Morrison Formation at the Red Canyon Ranch quarry site is not well exposed and is cut by numerous faults of unknown offset. It is difficult, therefore, to correlate the quarry site with the excellent exposures of the Morrison Formation along the nearby Red Gulch scenic byway. However, approximately 4 m below the discovery site there are two laterally discontinuous, thick channel sandstones. A section measured just off of the Red Gulch scenic byway is dominated by red, purple and green mudstones and siltstones, with occasional thin (< 30 cm) very fine sands, except in its middle third, where several thicker (up to 4 m), laterally discontinuous channel sands are observed (this appears to be Unit II of [4]; Fig 4). Mudstones and silts immediately above and below the channel sands are green to brown in colour, in contrast with the reds and purples of the upper and lower thirds of the section. It seems possible, therefore, that the section exposed at the Red Canyon Ranch may correlate with the middle part of the Morrison Formation at Red Gulch (Fig 4). The Howe and Howe-Stephens quarries, which are close by and have both produced a diverse range of dinosaur taxa, also appear to be from the middle part of the Morrison Formation in this area [5,6]. A radiometric date of 151.5±3 million years was obtained from a bentonite near the base of the Morrison Formation at the nearby Red Gulch dinosaur tracksite [7], indicating that the entirety of the Morrison Formation in this area could have been deposited during the Tithonian.

## Systematic Palaeontology

DINOSAURIA [8]
ORNITHISCHIA [9]
THYREOPHORA [10] (*sensu* [11])
STEGOSAURIA [12]
STEGOSAURIDAE [13]
*Stegosaurus* [12]

### Synonymy

*Diracodon* [14]
*Hypsirhophus* [15]
*Wuerhosaurus* [16]
*Hesperosaurus* [17]

### Diagnosis

From [18]. *Stegosaurus* possesses the following autapomorphies: (1) quadrate-squamosal-paroccipital process articulation overhangs the retroarticular process of the lower jaw; (2) postzygapophyses on posterior cervical vertebrae are elongated posteriorly and overhang the back of the centrum; (3) transverse processes on anterior caudal vertebrae (except for caudals one and two) project ventrally rather than laterally; (4) large; rectangular acromial process of the scapula; (5) hypertrophied lateral process (supra-acetabular process) diverges at an angle of 90 degrees from the preacetabular process of the ilium; (6) medial process present on the postacetabular process of the ilium.

*Stegosaurus stenops* [19]

### Synonymy

*Hypsirhophus discursus* [15]: 181 (*partim)*
*Stegosaurus ungulatus* [20]: 504
*Diracodon laticeps* [14]: 421
*Stegosaurus duplex* [19]: 416

### Revised diagnosis

Modified from [18]. Differs from all other stegosaurs in the following characteristics: (7) portion of the dentary anterior to the tooth row and posterior to the predentary edentulous; (8) dorsally elevated postzygapophysis of cervical vertebrae; (9) bifurcated summits of neural spines of the anterior and middle caudal vertebrae; (10) presence of dermal ossicles embedded in the skin on the underside of the cervical region.

### Holotype

USNM 4934, an almost complete skeleton.

### Referred specimens

Skeletons of varying completeness include: NHMUK PV R36730; AMNH 650; AMNH 470; AMNH 5752; BYU 12290; CEUM uncatalogued; CM 11341 (composite); DINO 2438; DMNH 1483; DMNH 2818; LHNB(CN) 1; USNM 4714; USNM 4936; USNM 6531; USNM 6646; YPM 1853; YPM 1856 (composite); YPM 1858. A full list of referred material can be found in [18] as referred material of *S*. *armatus*.

### Occurrence

All specimens except for LHNB(CN) 1 are known from the Upper Jurassic Morrison Formation of the western U.S.A. LHNB(CN) 1 is from the Upper Jurassic Lourinhã Group of Portugal [18, 21].

### Systematic remarks

The International Commission on Zoological Nomenclature (ICZN) recently stabilized the taxonomy of *Stegosaurus* by replacing the former type species, *Stegosaurus armatus*, with the better known species *Stegosaurus stenops* [22, 23].

NHMUK PV R36730 is referred to *Stegosaurus* because it possesses characters 1, 2, 4, and 5 from the generic diagnosis. Characters 3 and 6 are not preserved. The specimen is referred to *S*. *stenops* because it possesses characters 7 and 8 from the specific diagnosis. Character 9 is not preserved. No evidence for the presence of dermal ossicles in the cervical region was preserved in NHMUK PV R36730. It is possible, therefore, that this characteristic may vary with sex, ontogeny (as NHMUK PV R36730 was not fully skeletally mature at time of death; see below) or taxonomically. However, many of the bones of NHMUK PV R36730 were in poor condition when excavated [24] and it is possible that the ossicles were lost taphonomically. Further discoveries will have to be made to determine whether the presence of dermal ossicles on the underside of the cervical region is a reliable diagnostic character for *S*. *stenops*.

[18] also listed an unexpanded end of the pubic shaft as a specific character. However, NHMUK PV R36730 possesses a slightly expanded distalpubic shaft, and there appears to be a spectrum among stegosaurs from slightly expanded to greatly expanded distal pubic shafts (see pubis description below): thus, this character is removed as a diagnostic character of *Stegosaurus stenops* herein.

### Material

The postcranial skeleton of NHMUK PV R36730 is substantially complete, missing several cervical ribs, the centra of caudals 1–3, caudal vertebrae 4–19, most of the haemal arches, the entire left forelimb, the right manus and coracoid, the left ilium, some pedal phalanges and a single large plate from the pelvic region. Consequently, all major body regions are represented, and the only regions that are entirely absent are the base of the tail and the manūs (corresponding left or right elements are available for all other missing elements). Caudal vertebrae 4–19 were present originally, but were too poorly preserved to recover from the excavation [24].

## Description

### Presacral vertebrae

The presacral vertebral column was found in almost full articulation, so the locations of all vertebrae are known. There are 27 presacral vertebrae. Posterior to the atlas and axis, presacrals 3–11 bear a parapophysis located on the centrum and have cervical ribs. In presacrals 12–14 the location of the parapophysis is difficult to determine due to poor preservation, but presacral 14 is the first that bears a dorsal rib. Presacral 27 is fused to the sacrum and is a dorsosacral. There are therefore 13 cervical and 14 dorsal vertebrae. Measurements of all vertebrae can be found in Table 1.

**Table 1:** Vertebral measurements, in mm. **CD**, caudal vertebrae; **CV,** cervical vertebrae; **D**, dorsal vertebrae. Centrum lengths, widths and heights represent the maximum measurement; neural canal width and height are measured posteriorly and represent the maximum measurement; neural arch height is measured vertically from the base of the neural canal to the top of the neural spine.

	Centrum length	Anterior facet width	Anterior facet height	Posterior facet width	Posterior facet height	Neural canal width (measured posteriorly)	Neural canal height (measured posteriorly)	Neural arch height
**Atlas**	25	58	19			30	44	
**Axis**	68	48	29	33	45	19	24	
**CV3**	66	33	46	46	47	22	24	
**CV4**	71	41	46	41	46	25	29	
**CV5**	77	49	43	55	52	26	30	
**CV6**	80							
**CV7**	80							
**CV8**	76	62	46	73	41	26	23	
**CV9**	86					27	21	
**CV10**	84					21	20	
**CV11**	72					18	21	
**CV12**	100	65	85	99	72	33	33	103
**CV13**	93	91	79	87	84	37	35	128
**D1**	68	83	89	77	82	33	51	163
**D2**	80	104	79	99	88	33	37	162
**D3**	90	92	86	95	88	25	36	181
**D4**	90	84	87	91	83	20	19	189
**D5**	87	87	85	80	84	14	19	196
**D6**	93	82	74	87	79	23	31	201
**D7**	93	89	86	88	95	22	32	207
**D8**	92	92	80	91	96	24	30	195
**D9**	87	83	84	86	97	18	22	204
**D10**	81	82	97	75	95	18	26	209
**D11**	80	83	98	85	99	17	26	189
**D12**	82	84	101	81	104	16	22	175
**D13**	68	69	88	78	91	17	22	159
**CD1**								237
**CD2**								220
**CD3**								217
**CD21**	67	58	71	57	71			127
**CD22**	59	61	74	60	74			128
**CD23**	58	62	76	57	71			112
**CD24**	62	55	71	54	74			112
**CD25**	61	50	67	56	75			98
**CD26**	59	56	67	54	69			100
**CD27**	61	55	64	50	65			96
**CD28**	55	50	61	51	65			83
**CD29**	58	53	59	49	66			73
**CD30**	55	49	56	45	56			65
**CD31**	54	44	51	43	53			50
**CD32**	53	47	48	39	50			49
**CD33**	51	43	49	38	49			40
**CD34**	46	38	48	36	46			32
**CD35**	49	37	39	33	43			26
**CD36**	42	36	44	35	40			21
**CD37**	39	28	35	27	30			25
**CD39**	33	27	29	24	28			19
**CD40**	31	21	25	21	24			17
**CD41**	29	21	20	21	20			17
**CD42**	26	20	21	20	19			8
**CD43**	25	17	19	15	17			12
**CD44**	25	16	17	19	20			10
**CD45**	22	14	16	13	17			7

Twenty-seven presacrals are also known in the holotype, USNM 4934 [2]. [2] considered that these represented 10 cervicals and 17 dorsals, although expressed uncertainty about his assignments to the cervical and dorsal column. Thirteen cervical vertebrae are present in *Stegosaurus mjosi* but there are only 13 dorsals in this species (including two dorsosacrals), giving 26 presacral vertebrae in total (DMNH 29431; [17]). In *Dacentrurus* sp. from Iberia (= *Miragaia*), there were at least 17 cervical vertebrae (ML 433; [25]), but the total number of presacrals is unknown as the dorsals are not preserved. Nine cervical vertebrae are known in *Huayangosaurus taibaii*, along with 16 dorsals, giving a total presacral count of 25 (ZDM T7001; [26]). The holotype of *Gigantspinosaurus sichuanensis* (ZDM 0019) preserves eight cervical and 16 dorsal vertebrae, although it is not clear if this is the entire presacral column [27]. *Jiangjunosaurus* has at least 11 cervical vertebrae [28]. Sixteen dorsals (including three dorsosacrals) are present in *Dacentrurus armatus* (NHMUK OR46013). The number of presacral vertebrae therefore seems to vary interspecifically in stegosaurs and this feature may have taxonomic value.

In NHMUK PV R36730, cervical vertebrae get proportionately larger posteriorly along the neck, but otherwise maintain a similar morphology until the cervical-dorsal transition, between cervicals 11 and 13. In the transitional cervicals, the postzygapophyses become increasingly shorter and become elevated relative to the prezygapophyses, and the neural spines become proportionately more elongate. From dorsal 1 to dorsal 7, the vertebral centra become more elongate relative to their widths, but this decreases from dorsal 7 to dorsal 13. The dorsal neural arch pedicels becoming increasingly elongated dorsally from dorsal 1 to about dorsal 8, whereupon they maintain a similar height to dorsal 10, before become shorter until they are not elongated dorsal to the neural canal in dorsal 13. Transverse processes also become elevated to an increasing degree between dorsals 1 and about dorsal 8, but from dorsal 11 decrease in height to project laterally in dorsal 13. The parapophysis migrates dorsally from a location just dorsal to the neurocentral suture in dorsal 1 to a location at the base of the transverse process in dorsal 5. It is maintained at this location in the subsequent dorsal vertebrae.

#### Atlas (Fig 5)

**Fig 5:**
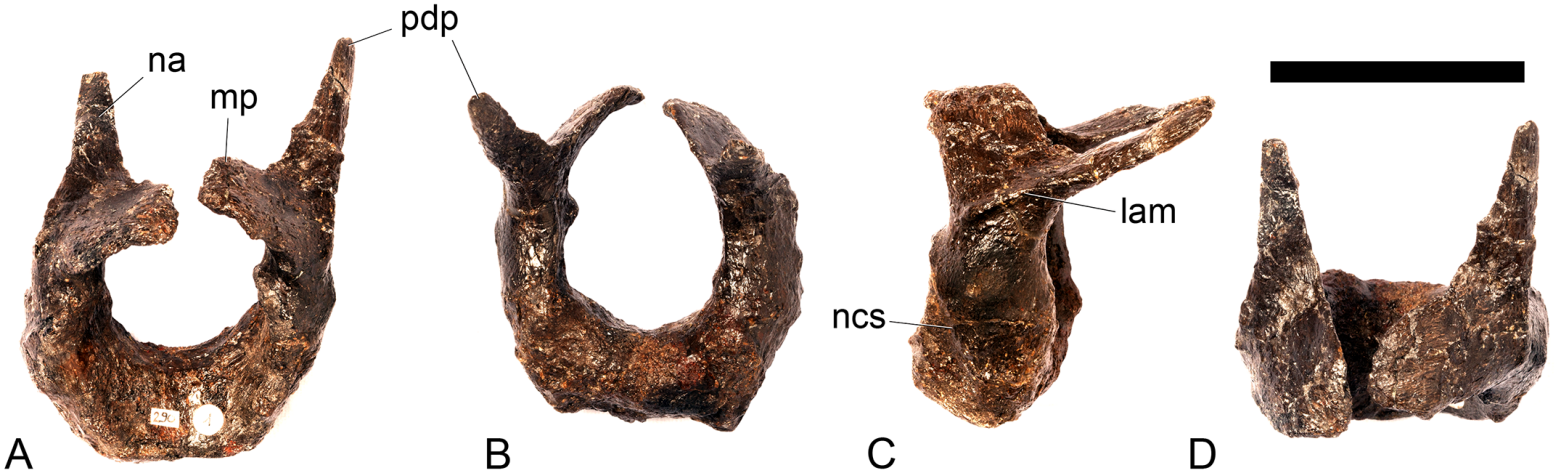
Atlas. **A**, anterior, **B**, posterior, **C**, left lateral and **D**, dorsal views. **Lam**, lamina; **mp**, medial process; **na**, neural arch; **ncs**, neurocentral suture; **pdp**, posterodorsal process. Scale bar equal to 5 cm.

The atlas and axis remain as separate elements and are unfused. The atlas is complete and well preserved, missing only the distal end of the right neural arch. It is slightly distorted obliquely so that the right neural arch is more anteriorly positioned than the left arch. The atlas is a U-shaped ring: the neural arches do not meet dorsally and are not fused to each other (Fig 5A and 5B). The neural arches are fused to the atlantal intercentrum although neurocentral sutures are clearly visible both medially and laterally (Fig 5C), as in *Kentrosaurus* (MB R.4778). The neural arches are unfused to the intercentrum in the holotype of *S*. *stenops*, USNM 4934 [2], and *S*. *mjosi* [17].

The dorsal surface of the atlantal intercentrum is saddle-shaped, being anteroposteriorly convex and transversely concave. The ventral surface of the intercentrum bears two prominent ridges that extend posterolaterally from the anterolateral corners of the anterior margin of the intercentrum for its entire length. A very subtle midline ridge separates two laterally positioned ventral cavities. The posterior surface of the intercentrum is slightly roughened. Both lateral surfaces of the atlas possess small irregular rugosities, which are asymmetrically positioned in anterior view and lie adjacent to the neurocentral suture. In lateral view, the neurocentral suture is V-shaped.

The neural arches are triradiate in lateral view, and each comprises a ventrally-directed pedicle attaching to the intercentrum (Fig 5C), a lobate, dorsoventrally compressed medial process, which roofs the neural canal, and a posterodorsally extending process that is elongate, dorsoventrally compressed and tapers to a sharp point posteriorly (Fig 5D). The latter process is the equivalent of a postzygapophysis and bears striations on both the dorsal and ventral surfaces of its distal tips. A lamina continuous with the lateral margin of the posterodorsal process extends anteriorly along the lateral surface of the neural arch to a point almost level with its anterior margin.

#### Axis (Fig 6)

**Fig 6:**
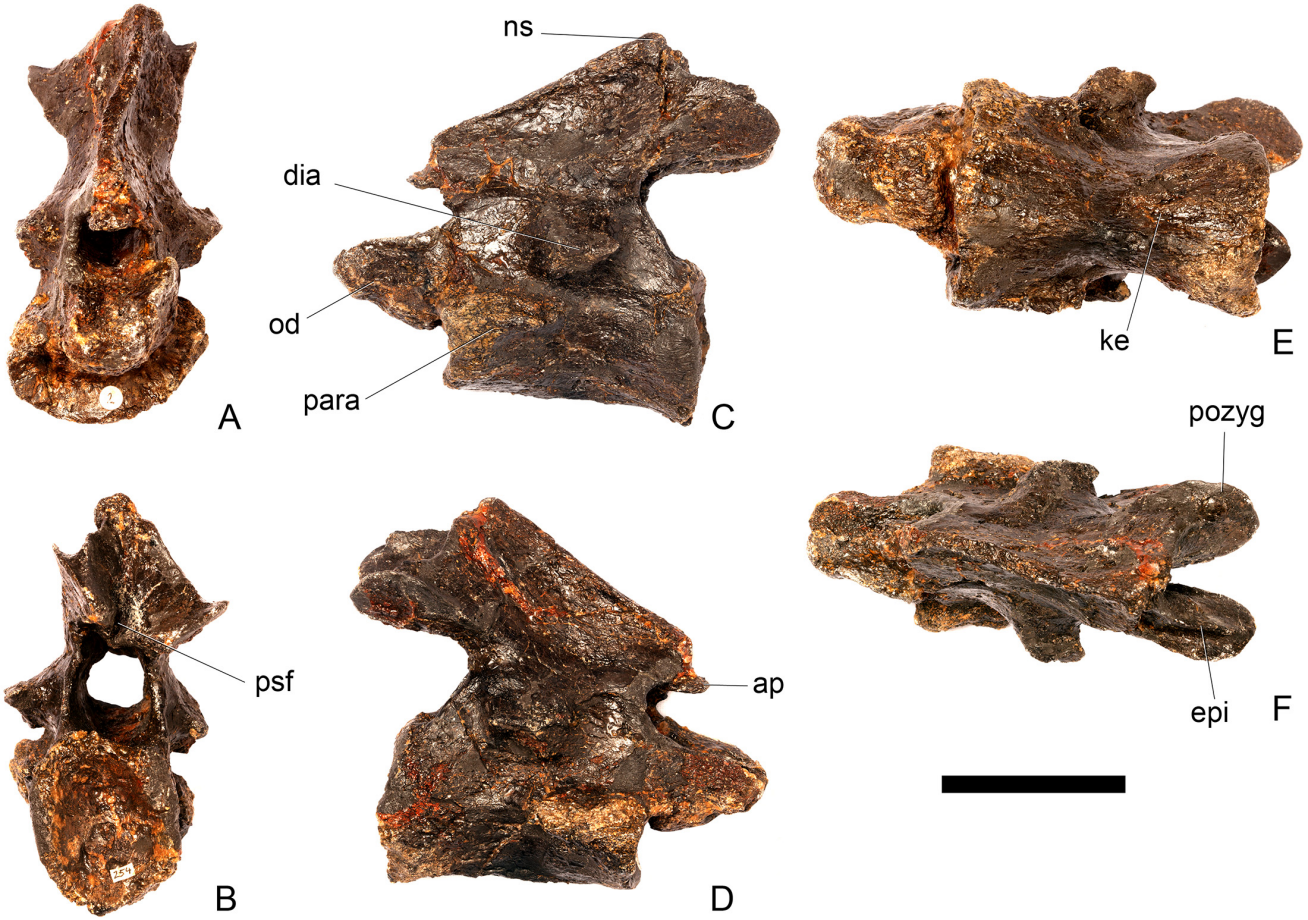
Axis. **A,** anterior, **B**, posterior, **C**, left lateral, **D**, right lateral, **E**, ventral and **F**, dorsal view. **Ap**, anteriorly projecting process; **dia**, diapophysis; **epi**, epipophyses; **ke**, keel; **ns**, neural spine; **od**, odontoid process; **para**, parapophysis; **pozyg**, postzygapophysis; **psf**, postspinal fossa. Scale bar equal to 5 cm.

The axis is complete, but slightly crushed transversely. The suture between the odontoid process and the centrum is visible indicating that the two structures are not fully fused (Fig 6C and 6E). The odontoid is also unfused to the axis in *S*. *mjosi* [17], but is fused in *Loricatosaurus* (NHMUK PV R3167; [29]: fig 13M-P). The odontoid is wedge-shaped in lateral view (Fig 6C) and has a blunt U-shaped outline in dorsal view (Fig 6F). Its dorsal surface bears a broad, shallow longitudinal groove continuous with the neural canal. The ventral surface is strongly convex. The anterior margins of the odontoid have been slightly eroded.

The centrum is longer anteroposteriorly than it is wide transversely and is hourglass-shaped in ventral view (Fig 6A, 6C and 6E). The anterior surface is dorsoventrally compressed; it consists of two parasagitally positioned depressions separated by a vertical midline swelling, and is pitted and rugose. In lateral view, parapophyses are present as distinct, wedge-shaped swellings that extend posteriorly from the anterior margin of the centrum, which terminate about halfway along it (Fig 6C and 6D). The parapophyses are located roughly equidistant from the dorsal and ventral margins of the centrum. The lateral surfaces of the centrum ventral to the parapophyses are gently concave anteroposteriorly and dorsoventrally. A prominent midline keel extends along the full length of the ventral margin of the centrum, separating the concave lateral surfaces (Fig 6E). The ventral margin of the axis of *S*. *mjosi* (DNMH 29431 [this specimen is a cast of the holotype, HMNH 001]) appears to be more concave upwards in lateral view than that of NHMUK PV R36730, which is straight. It is also straight in *Loricatosaurus priscus* (NHMUK PV R3167; [29]: fig 13M) and *Kentrosaurus* (MB R.4779). The posterior articular facet is deeply concave, elliptical in outline and taller than wide. The dorsal margin of the centrum is shallowly excavated by the neural canal.

The neural aches of the axis are fused with each other dorsally (Fig 6F). The neurocentral suture is indistinct and appears to be almost fully fused, with the exception of a short section along the posterior part of the left lateral surface. The neural arch lacks distinct prezygapophyses, but has a neural spine, postzygapophyses, and epipophyses. In lateral view the neural arch is sub-triangular with the anterior margin of the arch extending posterodorsally at an angle of approximately 40 degrees to the horizontal (Fig 6C and 6D). The neural arch appears to extend at a steeper angle to the horizontal in *S*. *mjosi* (DMNH 29431). A small, sharp anterior projection arising from the anterior margin of the arch slightly overhangs the anterior margin of the centrum. The lateral surfaces of the neural arch are gently concave and meet dorsally to form a narrow flat ridge. The neural canal has a circular outline in anterior view, becoming more ellipsoid and increasing in size posteriorly.

The neural spine is low, triangular and situated just dorsal to the level of the epipophyses. In contrast, the neural spine of the axis of *Jiangjunosaurus* is sub-rectangular in lateral view [28]. Postzygapophyses extend for a short distance posterior to the posterior margin of the centrum and in dorsal view diverge from the midline at an angle of approximately 20 degrees (Fig 6F). The left postzygapophysis is steeply inclined, forming an angle of approximately 80 degrees with the horizontal, whereas the right postzygapophysis is oriented at an angle of approximately 40 degrees. The prezygapophyses of cervical (Cv) 3 are inclined at a high angle, so the steeper angle is likely to be the natural condition. The postzygapophyses have flat, laterally facing articular facets that expand slightly transversely towards their distal ends, before terminating in a bluntly rounded margin (Fig 6F). Sharp, ridge-like epipophyses are present, arising on the dorsal surfaces of the postzygapophyses, a short distance anterior to their posterior margins. The epipophyses are continuous with sharp ridges that extend anterodorsally to merge with neural spine in lateral and dorsal views (Fig 6F). A shallow postspinal fossa separates the bases of the postzygapophyses.

In lateral view, the wedge-shaped diapophyses are positioned just dorsal to the neurocentral junction, at a point approximately halfway along the neural arch. They are anteroposteriorly short, shorter than the parapophyses, and extend posterolaterally in dorsal view. A faint horizontal ridge extends anteriorly from the anterior margin of the base of the diapophyses, terminating on the anterior margin of the neural arch. In overall morphology the axis is similar to that of *Loricatosaurus priscus* (NHMUK PV R3167; [29]: fig 13 M-P).

#### Cervical vertebra (Cv) 3 (Fig 7)

**Fig 7:**
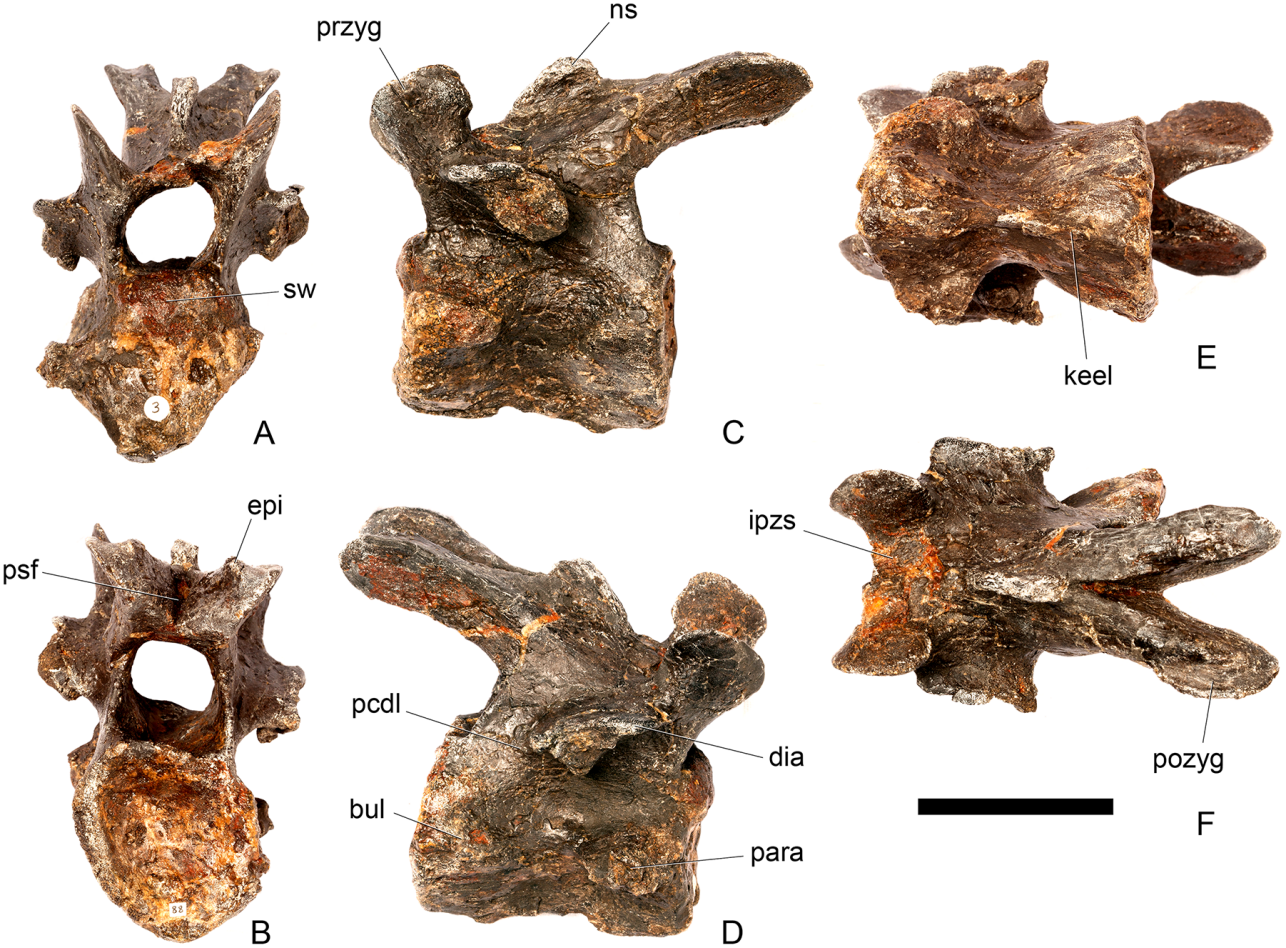
Cervical vertebra three. **A**, anterior, **B**, posterior, **C**, left lateral, **D**, right lateral, **E**, ventral and **F**, dorsal view. **Bul,** bulge; **dia**, diapophysis; **epi**, epipophysis; **ipzs**, interprezygapophyseal shelf; **keel**, ventral keel; **ns**, neural spine; **para**, parapophysis; **pcdl**, posterior centrodiapophyseal lamina; **pozyg**, postzygapophysis; **przyg**, prezygapophysis; **psf**, postspinal fossa; **sw**, swelling. Scale bar equal to 5 cm.

Cv3 is generally well preserved although it is obliquely sheared slightly so that left side of the element projects higher than the right side. This is most noticeable anteriorly (Fig 7A).

The centrum is longer anteroposteriorly than it is wide transversely and the ventral margin is straight, as it is in *Loricatosaurus* (NHMUK PV R3167; [29]: fig 13Q–T) and *Kentrosaurus* (MB R.4780). In contrast, the ventral margin of the centrum of Cv3 in *S*. *mjosi* (DNMH 29431) and *Dacentrurus* sp. (ML 433) is concave upwards, and the centrum of *Dacentrurus* sp. (ML 433) appears to be relatively longer. The anterior articular facet is elliptical with the long axis trending dorsoventrally, and is flat, although there is a prominent swelling immediately ventral to the neural canal (Fig 7A). The lateral surface of the centrum is divided into two concavities dorsally and ventrally which are separated by a gentle horizontal ridge that extends posteriorly from the parapophyses and increases in prominence to become a bulge proximal to the posterior articular facet (Fig 7C and 7D), as is also present in the holotype of *S*. *stenops* (USNM 4934; [2]) and in *Kentrosaurus* (MB R. 4780). The ridge is located approximately equidistant from the dorsal and ventral margin of the centrum in lateral view. The right parapophysis is better preserved than the left, which is slightly eroded. The right parapophysis is rectangular in dorsal view, being longer anteroposteriorly than it is dorsoventrally, and projects laterally (Fig 7F). In lateral view the parapophysis is triangular in cross-section, with the apex pointing dorsally (Fig 7D). The anterior margin of the parapophysis is continuous with the anterior articular facet of the centrum. The parapophyses are located halfway up the centrum in lateral view. The posterior articular facet is concave, shield-shaped in outline, and the dorsal margin, where it forms the base of the neural canal, is flattened (Fig 7B). The lateral margins are roughened. Ventrally, a strong keel extends anteroposteriorly for almost the full length of the centrum, fading out posteriorly (Fig 7E). [2] also noted a ventral keel in USNM 4934, as did [17] in *S*. *mjosi*. The centrum is shallowly excavated dorsally. No pneumatic foramina or nutrient foramina are present, in contrast to *Jiangjunosaurus*, in which large nutrient foramina are present on the lateral surfaces of the cervical centra [28].

The neural arch is fused to the centrum and the neurocentral suture is indistinct except at the base of the anterior margin of the left neural arch, where it is visible. The neural canal is rounded anteriorly and posteriorly, but is larger in posterior aspect (Fig 7A and 7B). The prezygapophyses arise from the anterolateral margin of the neural arch, project anterodorsally, and are lobate in lateral view (Fig 7A and 7C). They extend very slightly further anteriorly than the anterior surface of the centrum. By comparison, the prezygapophyses of *Dacentrurus* sp. (ML 433) project much further anteriorly than the anterior articular facet of the centrum in Cv3. The articular surfaces of the prezygapophyses extend sub-vertically, face medially, and are widely separated from each other (Fig 7A), as they are in USNM 4934 and *S*. *mjosi* (DMNH 29431). Between the prezygapophyses, the neural arch is flat and horizontally orientated to form an intraprezygapophyseal shelf (Fig 7A and 7F). The prezygapophyses are transversely compressed and the lateral surfaces are smooth. A small neural spine arises on the midline immediately posterior to the prezygapophyses and extends posterodorsally (Fig 7C and 7D). The anterior surface of the neural spine is flat and the surface striated. Dorsally, the neural spine is triangular in cross-section. [2] suggested that a true neural spine was not present on the anterior cervical vertebrae, but that a transversely narrow ridge was present in the same location. The postzygapophyses extend posterodorsally from the posterior margin of the neural arch beyond the posterior articular facet for a distance approximately equal to half of their total length (Fig 7C and 7D). The postzygapophyses are tongue-shaped and separated by a V-shaped groove of about 20 degrees from the midline in dorsal view (Fig 7F). The articular facets of the postzygapophyses face ventrolaterally at an angle of about 70 degrees to the horizontal and are flat. Ridges extend from the base of the neural spine along the dorsal surface of the postzygapophyses and terminate in epipophyses posteriorly. The epipophyses are slightly transversely expanded relative to the ridge. The ridges define the lateral margins of a shallow postspinal fossa (Fig 7F). Diapophyses project laterally from the sides of the neural arch. They are rectangular in dorsal view, longer anteroposteriorly than they are transversely, and extend from the midpoint of the centrum anteriorly to the base of the prezygapophyses (Fig 7A–7D). The left diapophysis is teardrop-shaped with the apex pointing anteriorly in cross-section, while the right diapophysis is triangular with the apex pointing ventrally. The diapophyses are about twice the length of those of the axis and extend further anteriorly. The articular surfaces of the diapophyses are flat to very slightly concave. A subtle ridge extends from the posterior margin of the diapophyses posteroventrally a short distance onto the neural arch. This ridge is in an equivalent position to the posterior centrodiapophyseal lamina of saurischians (PCDL: see [30] for the lamina terminology used throughout; Fig 7D). In *Dacentrurus* sp. (ML 433) cervical ribs are fused to the diapophyses and parapophyses of the entire cervical series, in contrast to the condition in NHMUK PV R36730 where they are all unfused.

#### Cervical vertebra 4 (Fig 8)

**Fig 8:**
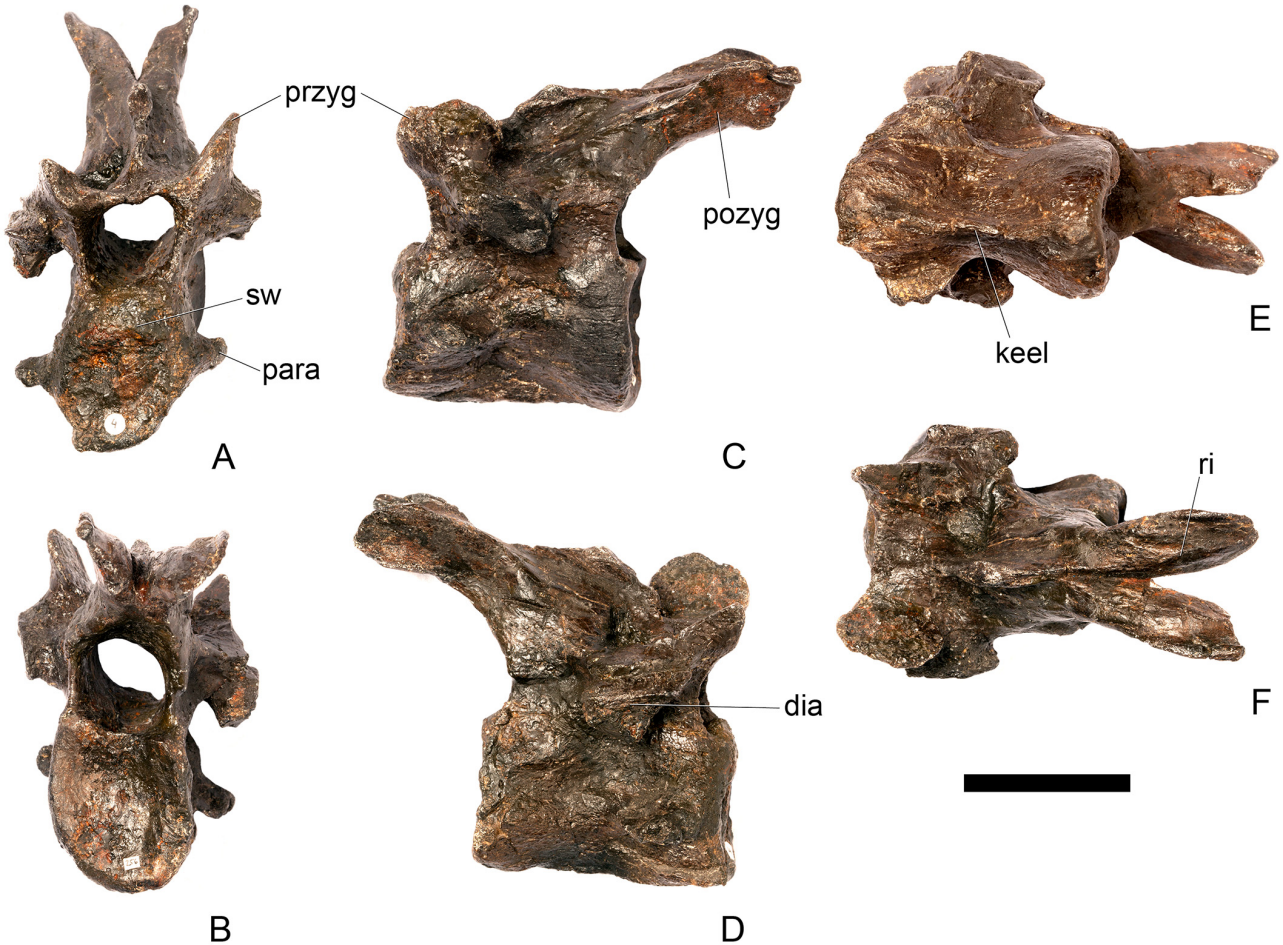
Cervical vertebra four. **A**, anterior, **B**, posterior, **C**, left lateral, **D**, right lateral, **E**, ventral and **F**, dorsal view. **Dia**, diapophysis; **keel**, ventral keel; **para**, parapophysis; **pozyg**, postzygapophysis; **przyg**, prezygapophysis; **ri**, ridge; **sw**, swelling. Scale bar equal to 5 cm.

Cv4 is well-preserved although the right prezygapophyses has been crushed and is angled ventrally, and the neural spine is broken dorsally. It generally resembles Cv3, except for the following differences. On the anterior surface of the centrum, the low swelling that covers the dorsal third of the surface is not as extensively developed as it is in Cv3 (Fig 8A). The parapophyses of Cv4 are shifted slightly posteriorly from the anterior margin of the centrum in lateral view, and have sub-elliptical cross-sections (Fig 8C and 8D). The ridge extending horizontally along the lateral surface of the centrum is positioned in a more dorsal location than in Cv3, so that the ventral concavity is more extensive than the dorsal one. The ventral keel in Cv4 extends from the anterior surface of the centrum continuously along its length to the posterior margin (Fig 8E). The prezygapophyses are not as steeply inclined as they are in Cv3. They are inclined at an angle of about 30 degrees to the horizontal with articular facets facing dorsomedially (Fig 8A). The prezygapophyses do not extend further anteriorly than the anterior border of the centrum. The postzygapophyses are curved, and are relatively and absolutely longer than in cervical three. They are also inclined further dorsally than those of Cv3, and their articular facets are inclined at an angle of approximately 80 degrees to the horizontal (Fig 8C and 8D). The postzygapophyses of Cv4 in *S*. *mjosi* (DNMH 29431) appear slightly shorter and are not as dorsally inclined as in NHMUK PV R36730. Epipophyses are absent from Cv4 and have been replaced by a low ridge that is confluent with the posterior margin of the neural spine (Fig 8F). The diapophyses are teardrop-shaped in cross-section in lateral view with the apex pointing anterodorsally.

#### Cervical vertebra 5 (Fig 9)

**Fig 9:**
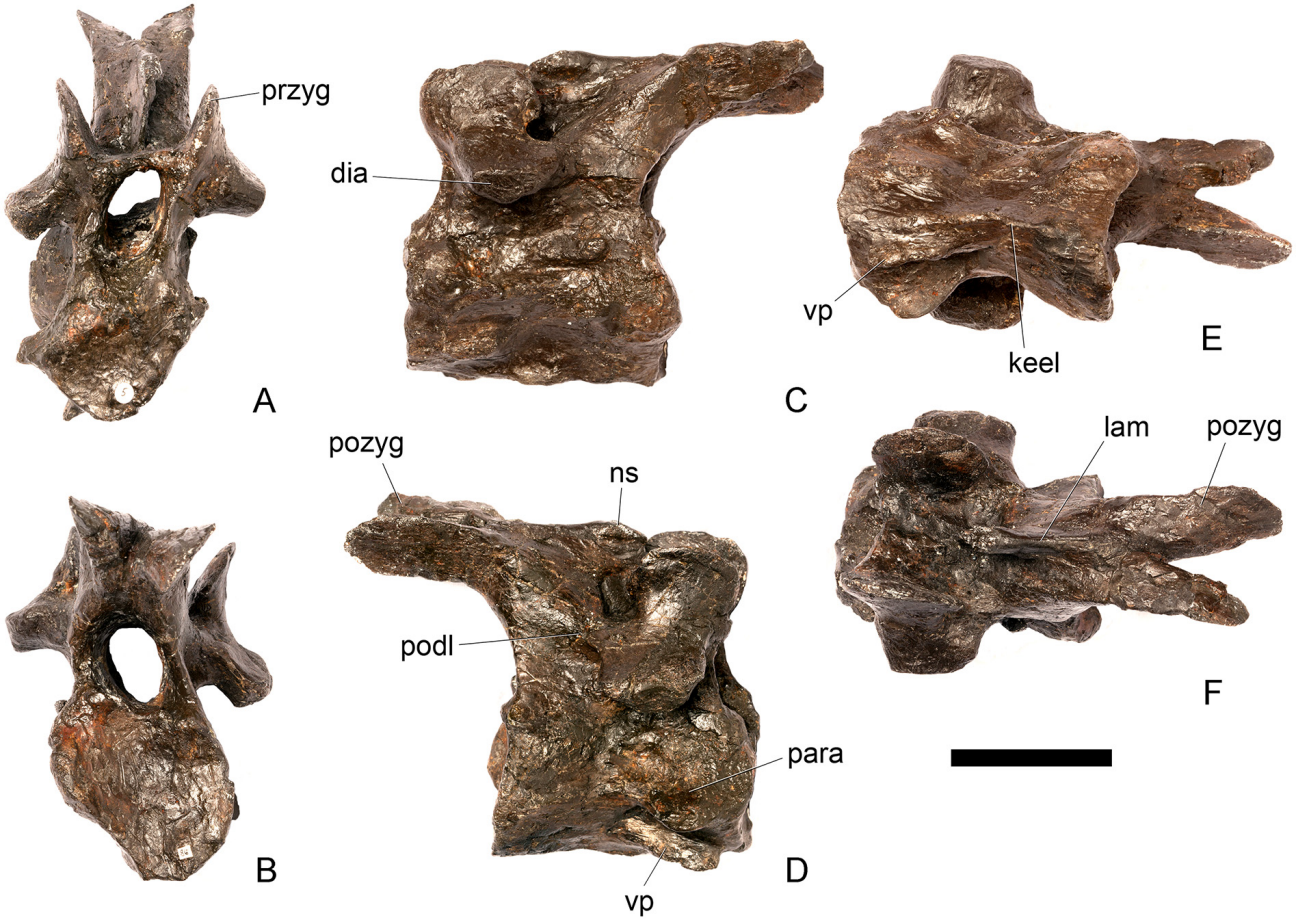
Cervical vertebra five. **A**, anterior, **B**, posterior, **C**, left lateral, **D**, right lateral, **E**, ventral and **F**, dorsal view. **Dia**, diapophysis; **keel**, ventral keel; **lam**, lamina; **para**, parapophysis; **podl**, postzygodiapophyseal lamina; **pozyg**, postzygapophysis; **przyg**, prezygapophysis; **vp**, ventral process. Scale bar equal to 5 cm.

Cv5 is generally well-preserved but is obliquely sheared so that the left side is higher than the right side and posteriorly a small portion of the left ventrolateral centrum is missing. It generally resembles Cv4, differing from it in the following respects. The anterior articular facet of the centrum is gently concave, and the dorsal swelling is reduced in prominence relative to that of Cv4 (Fig 9A). A shallow concavity is present dorsal to the parapophysis in lateral view (Fig 9C). The posterior surface of the centrum is larger than the anterior surface. Ventrally, either side of the keel laterally, just posterior to the anterior margin are two small processes that are anteroposteriorly elongate and transversely compressed (Fig 9E). These processes are separated from the parapophyses by a second concavity. The prezygapophyses project dorsally and their articular facets are sub-vertical and face medially, and are more similar to those of Cv3 than Cv4, perhaps suggesting that Cv4 is slightly deformed in this region. The neural spine extends vertically from the midline about level with the posterior margin of the prezygapophyses in lateral view (Fig 9D). It is transversely compressed, and expands slightly at its apex. The neural spine of Cv5 is much better developed and projects further dorsally in *Huayangosaurus* (ZDM T7001). A ridge extends posteriorly from the posterior margin of the neural spine and terminates at the point where the postzygapophyses diverge (Fig 9F). There is no postspinal fossa. Posterior to the neural spine, the neural arch expands dorsally relative to the condition in the preceding vertebrae, so that the bases of the postzygapophyses are at the same level as the top of the neural spine. The postzygapophyses extend posteriorly from this point, so that they are almost horizontal in lateral view (Fig 9C and 9D). This has the effect of increasing the height of the neural arch. The dorsomedial surfaces of the postzygapophyses are convex and no epipophyses or ridges are present (Fig 9F). The postzygapophyses in *Dacentrurus* sp. (ML 433) and *Huayangosaurus* (ZDM T7001) do not extend as far posteriorly as in NHMUK PV R36730. The diapophyses are teardrop–shaped in lateral view with the apex pointing posteriorly. Ridges extend from the posterior margin of the diapophyses towards to base of the postzygapophyses in a position equivalent to the postzygodiapophyseal lamina (PODL) of saurischians, although they do not extend far enough to reach the postzygapophyses themselves (Fig 9D).

#### Cervical vertebra 6 (Fig 10)

**Fig 10:**
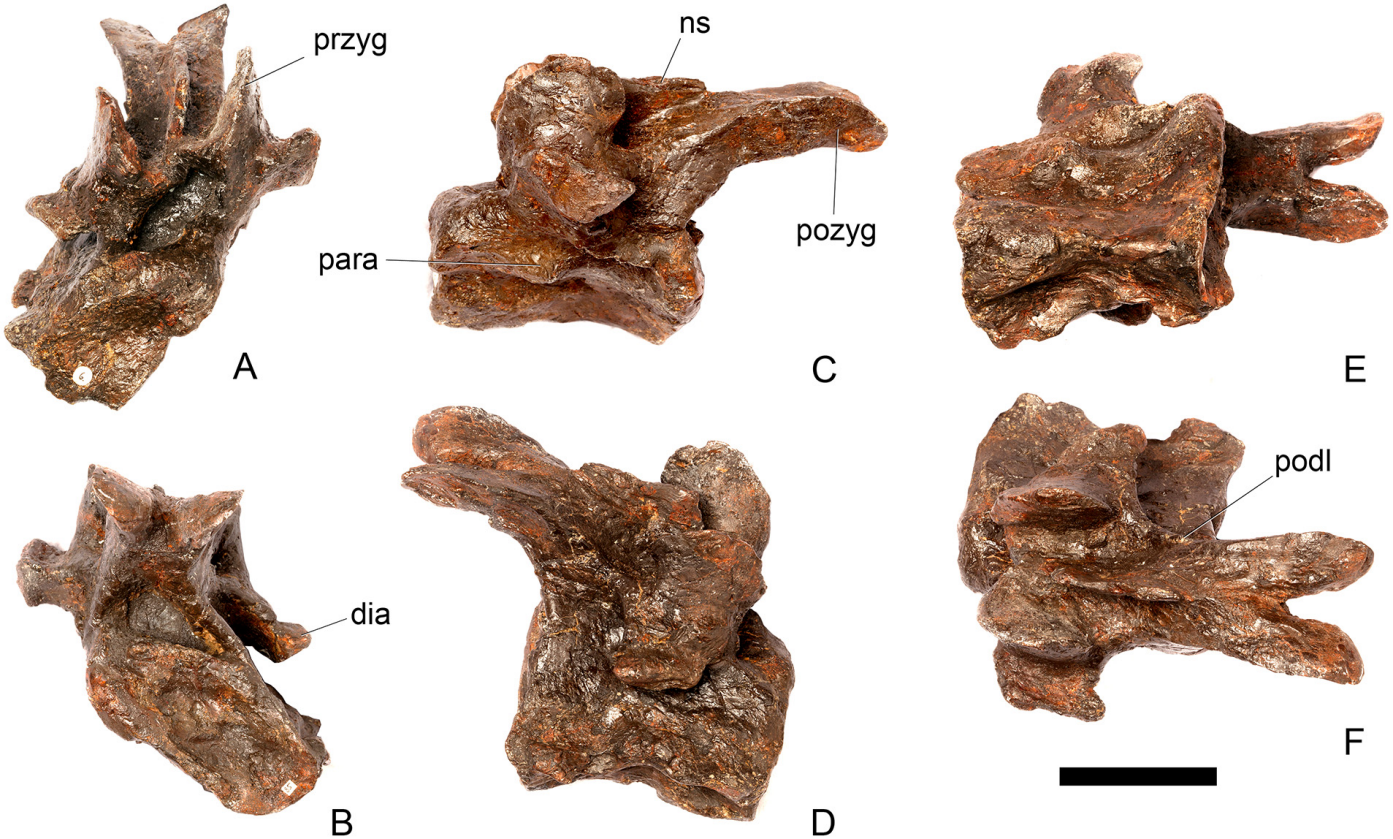
Cervical vertebra six. **A**, anterior, **B**, posterior, **C**, left lateral, **D**, right lateral, **E**, ventral and **F**, dorsal view. **Dia**, diapophysis; **ns**, neural spine; **para**, parapophysis; **podl**, postzygodiapophyseal lamina; **pozyg**, postzygapophysis; **przyg**, prezygapophysis. Scale bar equal to 5 cm.

Cv6 is largely complete but strongly deformed and has been obliquely sheared so that the left side is higher than the right. As far as can be determined, it is very similar to Cv5, with the following exceptions. The parapophyses are no longer distinct processes on stalks but appear to be rugosities, although they are not well preserved (Fig 10C and 10D). The neural arch appears to have shifted slightly posteriorly relative to the centrum so that it is set back from the anterior articular facet (Fig 10C and 10D). In contrast, the neural arch of Cv6 in *Dacentrurus* sp. (ML 433) is not shifted posteriorly and the prezygapophyses still overhang the anterior articular facet. The neurocentral suture is not visible. The base of the neural spine extends further anteriorly so that the lamina that forms the anterior surface of the spine is obscured by the prezygapophyses in lateral view (Fig 10D). The PCDL does not appear to be as prominent as in the preceding vertebrae, whereas the PODL is more prominent than it is in Cv5.

#### Cervical vertebra 7 (Fig 11)

Cv7 is largely complete but some reconstruction is present on the left side. It is also deformed by oblique shearing so that the right side is depressed relative to the left side. The combination of reconstruction and shearing mean that some details of the anatomy are obscured: the positions of the parapophyses, the shapes of the anterior and posterior articular surfaces, the presence or absence of a ventral keel and concavities lateral to it, the presence or absence of a ridge on the lateral side of the centrum, and the orientation of the diapophyses cannot be determined.

**Fig 11:**
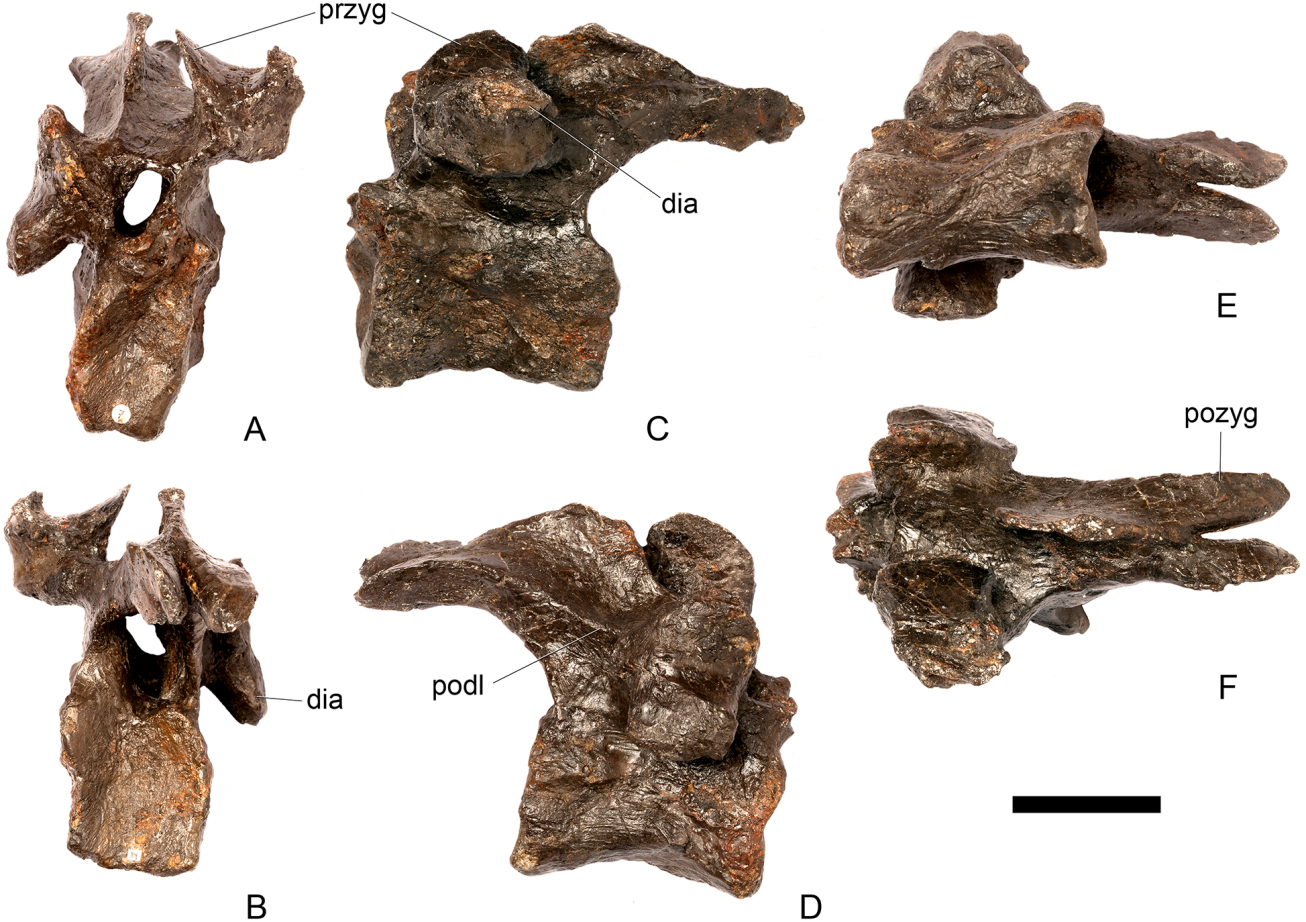
Cervical vertebra seven. **A**, anterior, **B**, posterior, **C**, left lateral, **D**, right lateral, **E**, ventral and **F**, dorsal view. **Dia**, diapophysis; **podl**, postzygodiapophyseal lamina; **pozyg**, postzygapophysis; **przyg**, prezygapophysis. Scale bar equal to 5 cm.

Despite this reconstruction and deformation, enough detail is preserved to determine that Cv7 differs from the preceding vertebrae in the following ways. The neurocentral suture is not visible. The prezygapophyses are very large fan-shaped processes (Fig 11C and 11D). They differ noticeably from the prezygapophyses of Cv7 in *Dacentrurus* sp. (ML 433) because in the latter they project anterolaterally, overhanging the anterior articular facet. The postzygapophyses extend sub-parallel to each other, and are separated by a narrow notch (Fig 11F). In comparison to *Dacentrurus* sp. (ML 433) and *Kentrosaurus* (MB R.4783), the postzygapophyses of NHMUK PV R36730 overhang the posterior articular facet to a greater degree. The diapophyses are robust processes with a sub-elliptical cross-section in lateral view. They extend further anteriorly than in preceding vertebrae, extending across the lateral surfaces of the prezygapophyses and reaching their anterior margin (Fig 11C and 11D). In *Dacentrurus* sp. (ML 433) and *Huayangosaurus* (ZDM T7001; [31]: Fig 11), the diapophyses are located ventral to the small neural spine and do not extend across the lateral surfaces of the prezygapophyses, while in *Kentrosaurus* (MB R.4783) the diapophyses are located on the lateral surface of the prezygapophyses, and are thus more elevated than in NHMUK PV R36730. There is no evidence for a PCDL on either side of Cv7, but the centrum and neural arch is damaged on both sides in this region. The PODL extends onto the lateral surface of the postzygapophysis (Fig 11D). In comparison with the same vertebra in *Dacentrurus* sp. (ML 433), the centrum of NHMUK PV R36730 is anteroposteriorly shorter.

#### Cervical vertebra 8 (Fig 12)

**Fig 12:**
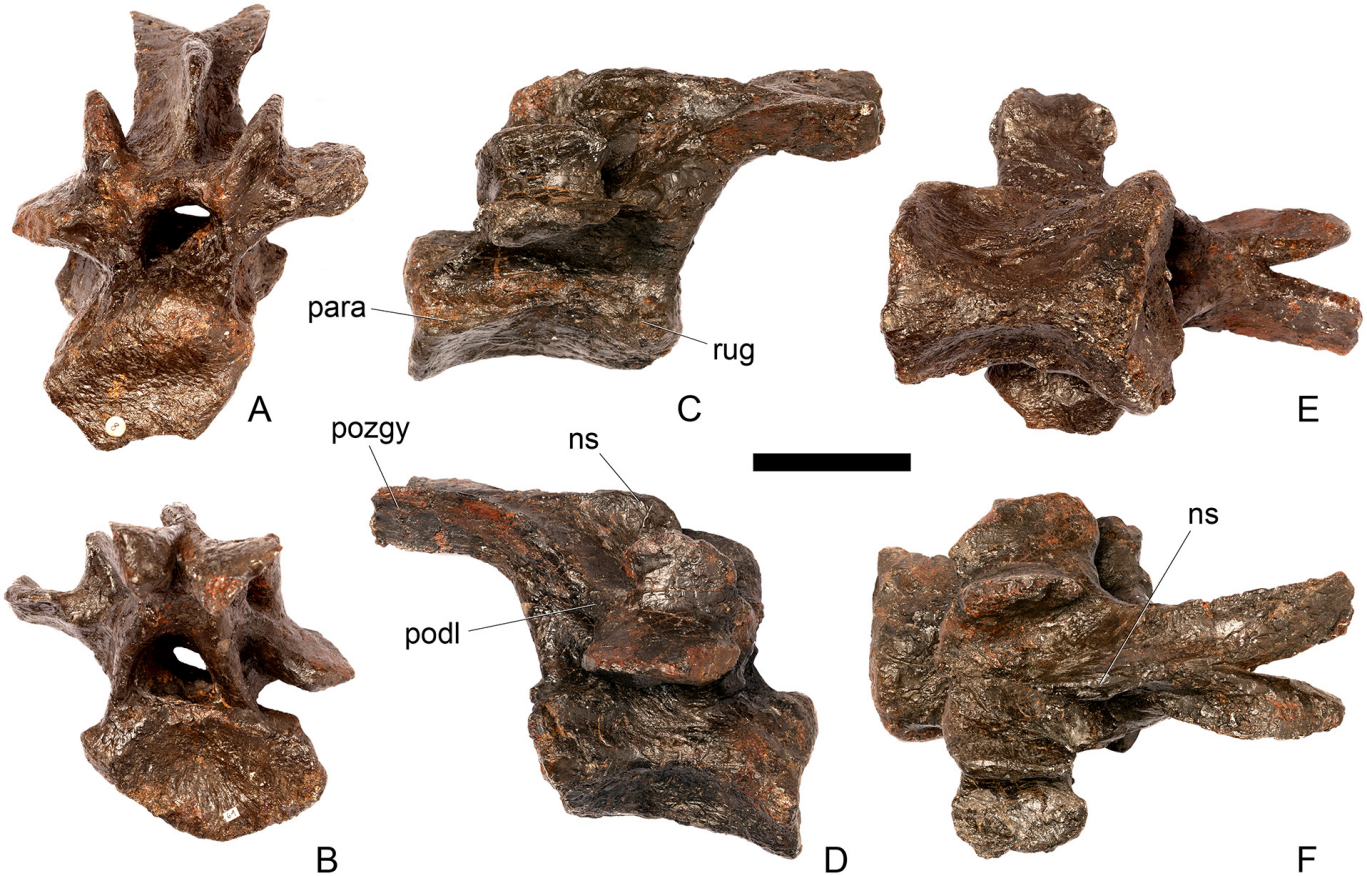
Cervical vertebra eight. **A**, anterior, **B**, posterior, **C**, left lateral, **D**, right lateral, **E**, ventral and **F**, dorsal view. **Ns**, neural spine; **para**, parapophysis; **podl**, postzygodiapophyseal lamina; **pozyg**, postzygapophysis; **rug**, rugosity. Scale bar equal to 5 cm.

Cv8 is slightly obliquely sheared so that the left side is located further dorsally than the right. It is very similar to Cv5–7, differing only in the following respects. The parapophyses are reduced relative to the more anterior cervical vertebrae, and are present as low swellings in lateral view that are triangular in outline with the apex pointing posteriorly. The ridge extending posteriorly from their posterior margin is maintained and, as in the preceding cervicals, terminates in a low rugosity close to the lateral surface of the posterior articular facet (Fig 12C). In ventral view, the rugose processes lateral to the midline keel observed in Cv5 are present but reduced in prominence and are anteroposteriorly shorter than they are in the latter (Fig 12E). The neural spine is a transversely compressed plate that is slightly transversely expanded dorsally and flat on top. In lateral view, the anterior margin of the neural spine is gently S-shaped and its dorsal margin is located at approximately the same height as the dorsal margins of the postzygapophyses, so that the neural spine is continuous with the postzygapophyseal pedicel (Fig 12C and 12D). The neural spine of Cv8 in *Dacentrurus* sp. (ML 433) is better developed, forming a distinct, dorsally projecting process that extends above the level of the postzygapophyses. The postzygapophyses project posterior to the centrum in lateral view, and extend horizontally from the neural arch (Fig 12C and 12D). The PODL extends posteriorly from the diapophyses to the dorsal surface of the postzygapophyses and continues to form the lateral margin of the postzygapophyseal articular facet (Fig 12D).

#### Cervical vertebra 9 (Fig 13)

**Fig 13:**
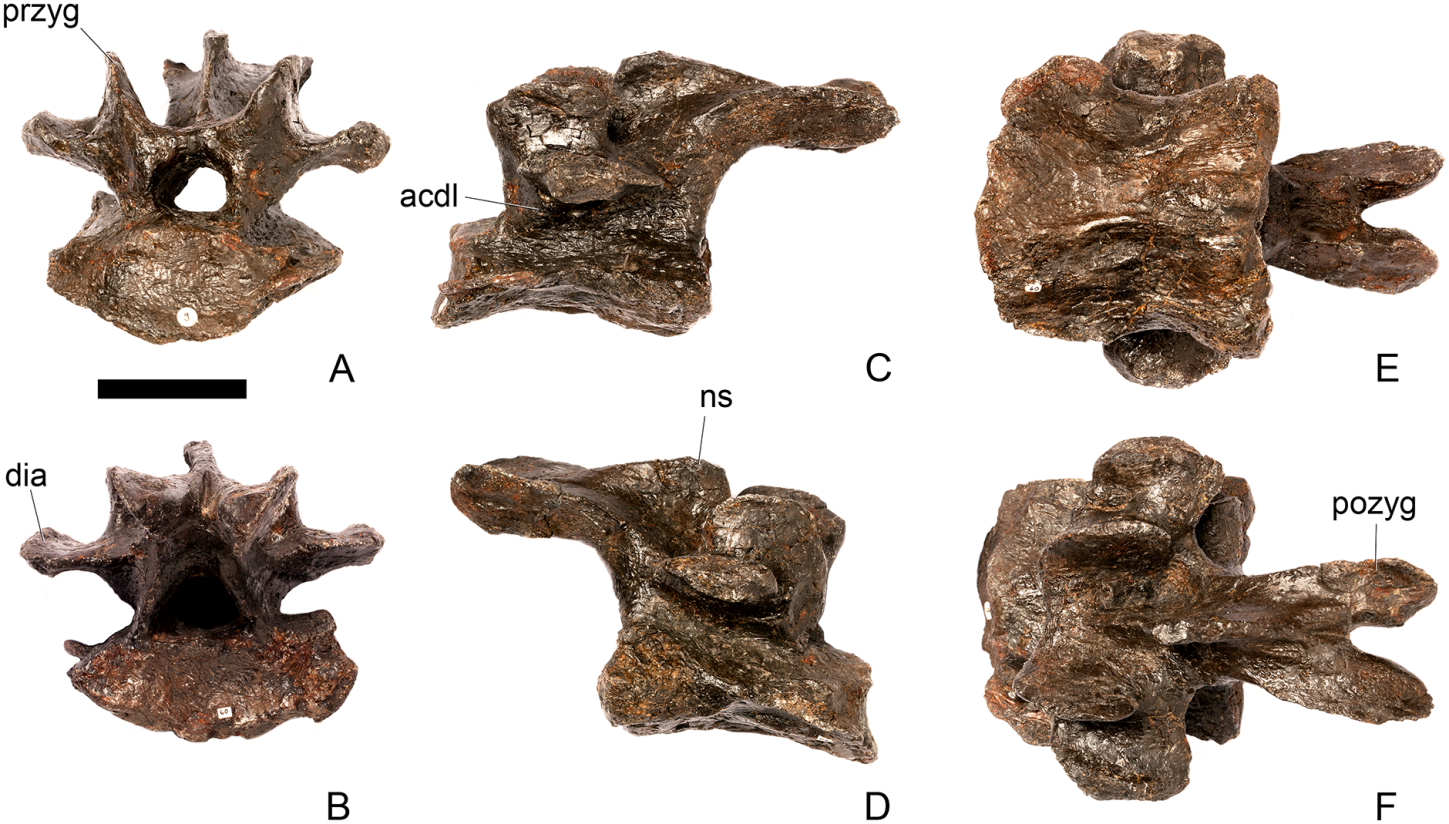
Cervical vertebra nine. **A**, anterior, **B**, posterior, **C**, left lateral, **D**, right lateral, **E**, ventral and **F**, dorsal view. **Dia**, diapophysis; **ns**, neural spine; **para**, parapophysis; **pozyg**, postzygapophysis. Scale bar equal to 5 cm.

The centrum of Cv9 has been strongly dorsoventrally compressed and most of its anatomy is obscured by crushing. Ventrally, the centrum is covered in plaster. The vertebra is very similar to Cv8 except in the following respects. The anterior margin of the neural spine angles dorsoventrally from its base before curving dorsally about one-third of the way up the spine, in contrast to the S-shaped anterior margin in Cv8. The postzygapophyses project posterodorsally from the posterior neural arch (Fig 13C and 13D). A very weak PCDL is present that extends from about halfway along the posteroventral surface of the diapophyses towards the presumed location of the neurocentral suture. Another weak buttress, corresponding in position to the anterior centrodiapophyseal lamina (ACDL), extends from the anteroventral diapophysis anteroventrally to the presumed location of the neurocentral suture level with the anterior margin of the neural arch (Fig 13C). A shallow concavity on the anterolateral surface of the neural arch is bounded posteriorly by the ACDL, anteriorly by the lateral margin of the neural canal, and dorsally by the prezygapophyses (Fig 13A). By comparison with *Dacentrurus* sp. (ML 433), the neural arch is set back from the anterior articular facet in NHMUK PV R36730, as in the preceding vertebrae. The neural arch is not set back from the anterior articular facet in *Huayangosaurus* (ZDM T7001; [31]: fig 11).

#### Cervical vertebra 10 (Fig 14)

**Fig 14:**
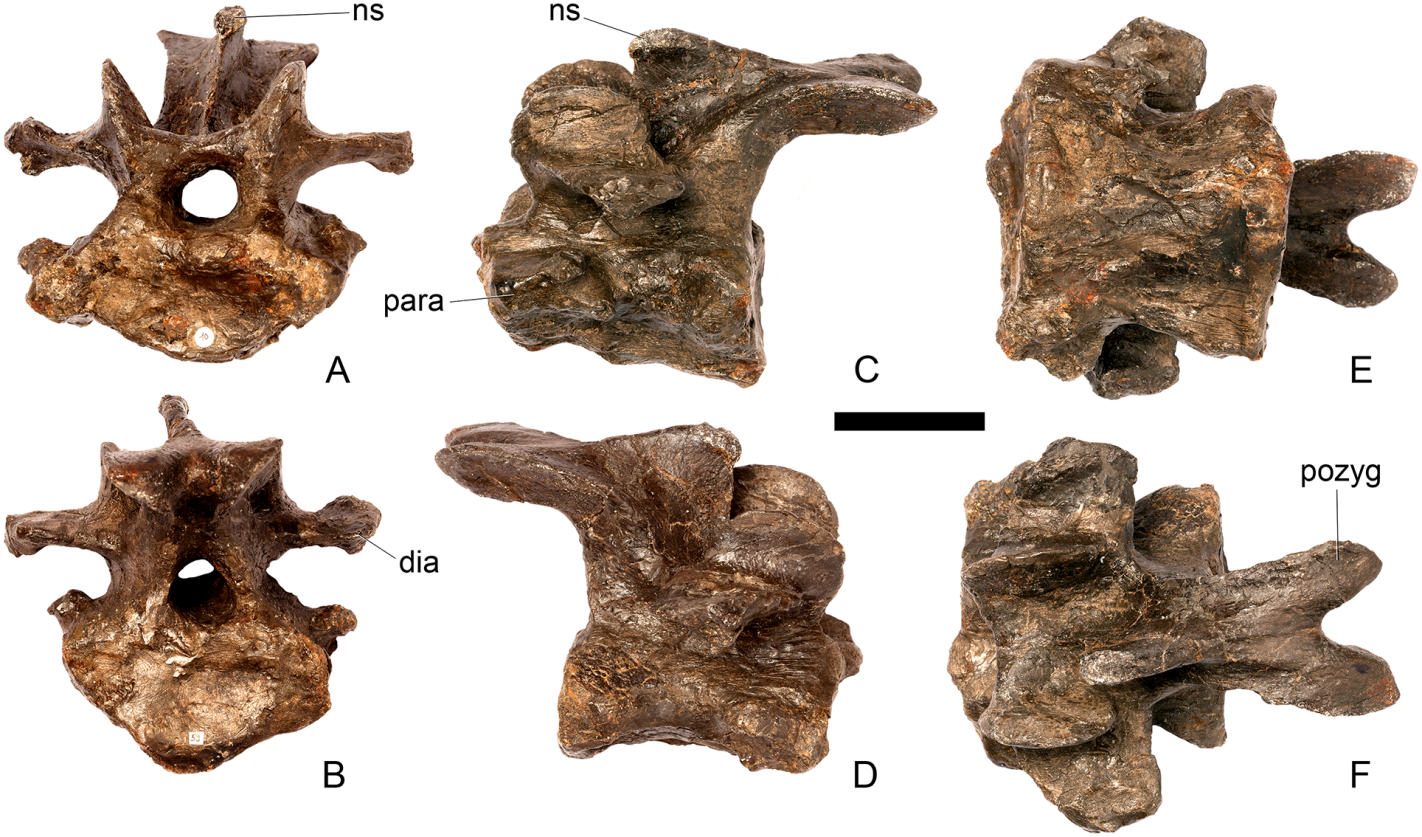
Cervical vertebra 10. **A**, anterior, **B**, posterior, **C**, left lateral, **D**, right lateral, **E**, ventral and **F**, dorsal view. **Acdl**, anterior centrodiapophyseal lamina; **dia**, diapophysis; **ns**, neural spine; **pozyg**, postzygapophysis; **przyg**, prezygapophysis. Scale bar equal to 5 cm.

The centrum of Cv10 is dorsoventrally crushed. The left side of the neural arch has been extensively restored with plaster, and the left prezygapophyses, parapophysis and diapophysis are all reconstructed. Cv10 is very similar to the preceding vertebrae except in the following respects. The parapophysis bifurcates posteriorly in lateral view, so that it is shaped like a ‘Y’ lying on its side, with a shallow fossa between the two arms of the ‘Y’ (Fig 14C and 14D). The dorsal arm of the ‘Y’ projects more strongly than the ventral arm. The anterior margin of the neural spine is concave in lateral view, curving posterodorsally from its base and then curving anterodorsally close to its top (Fig 14D). The transverse expansion at the top of the neural spine is better developed than on the preceding vertebrae (Fig 14C and 14D), but the neural spine is not as well developed as it is on Cv10 of *Dacentrurus* sp. (ML 433), or in Cv7 of *Huayangosaurus* (ZDM T7001; [31]: fig 11) in which it extends dorsal to the level of the postzygapophyses. The postzygapophyses diverge from the midline at an angle of around 20 degrees, and are joined together for more of their length than in preceding vertebrae (Fig 14F). The postzygapophyses of *Huayangosaurus* (ZDM T7001; [31]: fig 11) do not project beyond the posterior articular facet, in contrast to the condition in NHMUK PV R36730 (Fig 14C and 14D). The posterior margin of the neural arch ventral to the postzygapophyses is straight, while it is more curved in the preceding vertebrae and in *Dacentrurus* sp. (ML 433). This results in elevation of the postzygapophyses relative to those of Cv7, for example. [2] noted that the height of the neural arches increased posteriorly along the cervical column in USNM 4934 and it seems likely that this the same occurs in NHMUK PV R36730. The diapophyses of Cv10 are slightly longer than those of Cv9, projecting further laterally (Fig 14A and 14B). In lateral view the right diapophysis is triangular in lateral cross-section with the apex pointing ventrally (Fig 14D).

#### Cervical vertebra 11 (Fig 15)

**Fig 15:**
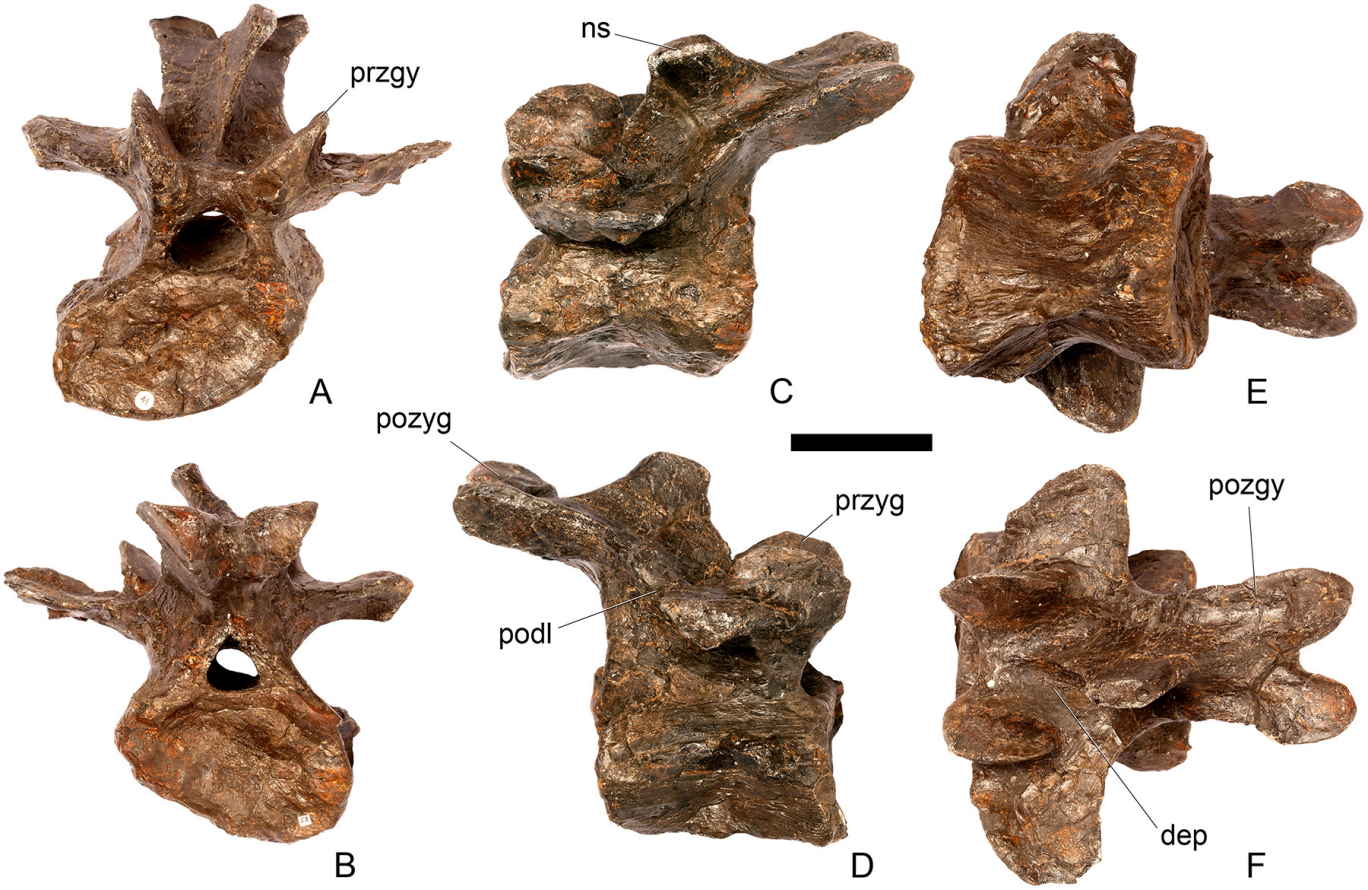
Cervical vertebra 11. **A**, anterior, **B**, posterior, **C**, left lateral, **D**, right lateral, **E**, ventral and **F**, dorsal view. **Dep**, longitudinal depression; **ns**, neural spine; **podl**, postzygodiapophyseal lamina; **pozgy**, postzygapophysis; **przyg**, prezygapophysis. Scale bar equal to 5 cm.

Cv11 is poorly preserved. The centrum is dorsoventrally crushed and sheared obliquely so that the left side is higher than the right. Anteriorly, laterally and ventrally it is covered in plaster and details of its anatomy are therefore obscured. The neural arch appears to have shifted slightly posteriorly along the neurocentral suture, which can be seen posterolaterally. This has the effect of causing the dorsal margin of the posterior articular facet to overhang the rest of the facet, and is likely a preservational artifact (Fig 15B). The left diapophysis and postzygapophysis are reconstructed. However, some anatomical details of the neural arch are preserved that allow comparisons with the preceding vertebrae. The prezygapophyses are angled dorsolaterally, and in comparison with the preceding vertebrae their articular surfaces face more dorsomedially, diverging from the vertical plane at an angle of around 30 degrees (Fig 15A). The anterior margin of the neural spine is anteriorly concave in lateral view, becoming vertical in its dorsal part (Fig 15D). The spine forms more of a distinct process than in the preceding vertebrae; it projects well above the level of the postzygapophyses and terminates in a sub-rectangular apex (Fig 15C and 15D), as it does in *Dacentrurus* sp. (ML 433). Its posterior margin is shallowly concave in lateral view and extends ventrally to meet the bases of the postzygapophyses. In anterodorsal view, shallow, longitudinal depressions lie adjacent to the base of the spine in the area between the spine and the prezygapophyses, and these merge into the intraprezygapophyseal shelf (Fig 15F). The dorsal margins of the postzygapophyses are situated at a level higher than the prezygapophyses, in contrast to the condition in the preceding vertebrae (Fig 15C and 15D). The postzygapophyses are separated from each other by a notch that extends for one-third of the length of the postzygapophyses; this notch is shorter than in preceding vertebrae (Fig 15F). There are faint indications of the PCDL and ACDL, but the PODL is well-developed, as in the preceding vertebrae.

#### Cervical vertebra 12 (Fig 16)

The centrum of Cv12 is poorly preserved, crushed ventrally, and the lateral surfaces have been restored with plaster: however, it is longer anteroposteriorly than it is wide transversely across the articular facets. This contrasts with the condition in *Dacentrurus armatus* (NHMUK OR46013; [29]: fig 2A–D), where two probable posterior cervical centra are wider transversely across the articular facets than they are long anteroposteriorly. Damage and restoration to the centrum of NHMUK PV R36730 means that the location of the parapophyses and the presence or absence of a ventral keel cannot be determined.

**Fig 16:**
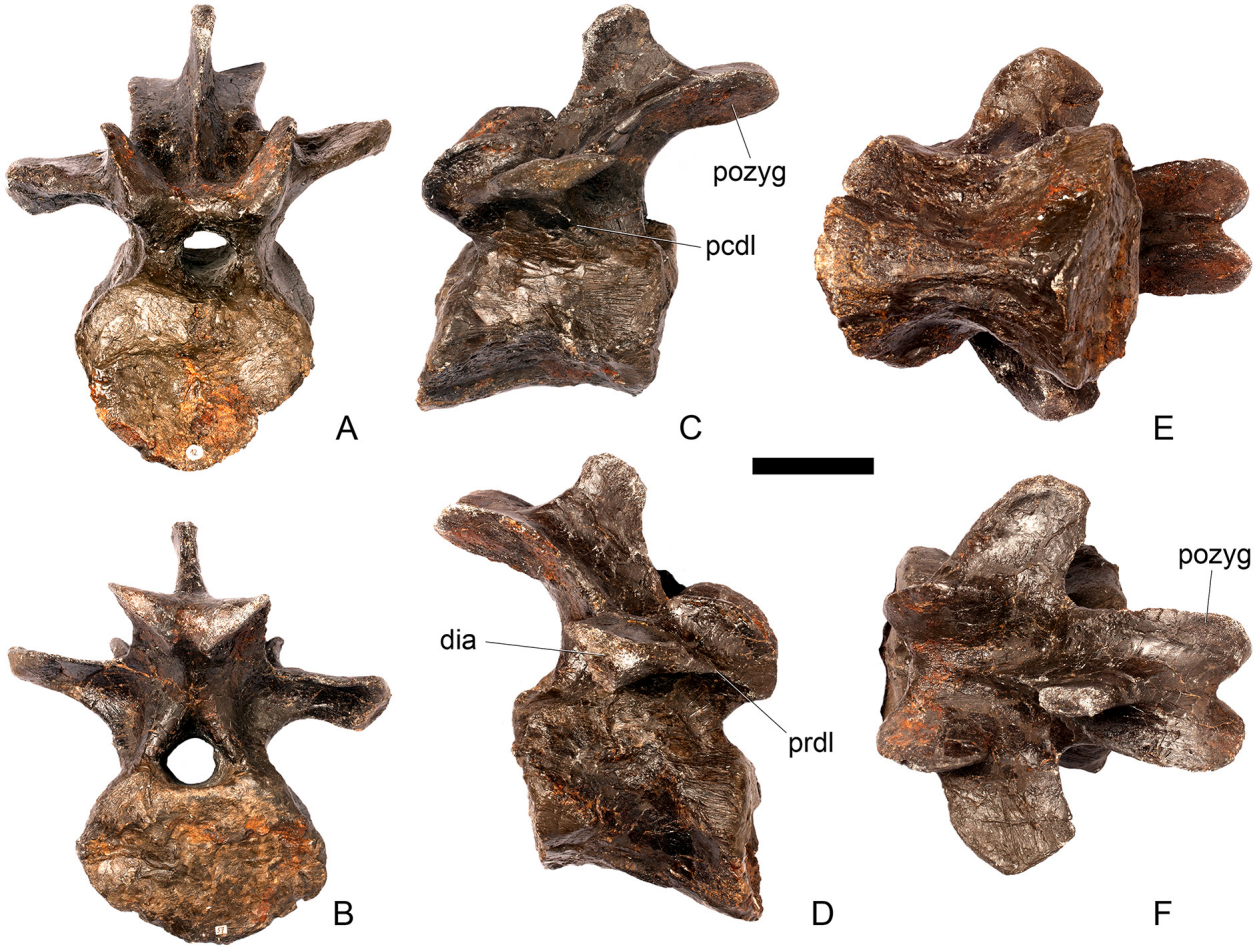
Cervical vertebra 12. **A**, anterior, **B**, posterior, **C**, left lateral, **D**, right lateral, **E**, ventral and **F**, dorsal view. **Dia**, diapophysis; **pcdl**, posterior centrodiapophyseal lamina; **pozyg**, postzygapophysis; **prdl**, prezygodiapophyseal lamina. Scale bar equal to 5 cm.

Cv12 is similar to the preceding vertebrae except in the following respects. Although the neural arch is slightly set back from the anterior margin of the centrum, it is set back to a lesser degree than in preceding vertebrae (Fig 16C and 16D) and is similar to *Dacentrurus* sp. (ML 433) in this regard. The postzygapophyses project posterodorsally and do not extend as far beyond the posterior margin of the centrum as they do in the preceding vertebrae (Fig 16C and 16D). They are fused along their midline for almost their entire length (Fig 16F). As in the preceding vertebrae, the diapophyses are triangular in lateral cross-section with the apex pointing ventrally. This ventral apex is continues medially along the ventral diapophysis and extends onto the lateral surface of the neural arch posteroventral to the diapophysis to form the PCDL (Fig 16C). The anterior margin of the diapophysis continues as a ridge onto the lateral surface of the prezygapophyses, terminating at a point just posterior to their anterior margins. This ridge is equivalent to the prezygapophyseal diapophyseal lamina (PRDL; Fig 16D). The ACDL is absent. Ventral to the PRDL, a shallow fossa covers the surface of the neural arch. Posterior to the PCDL a second shallow fossa is present (Fig 16B and 16C).

#### Cervical vertebra 13 (Fig 17)

**Fig 17:**
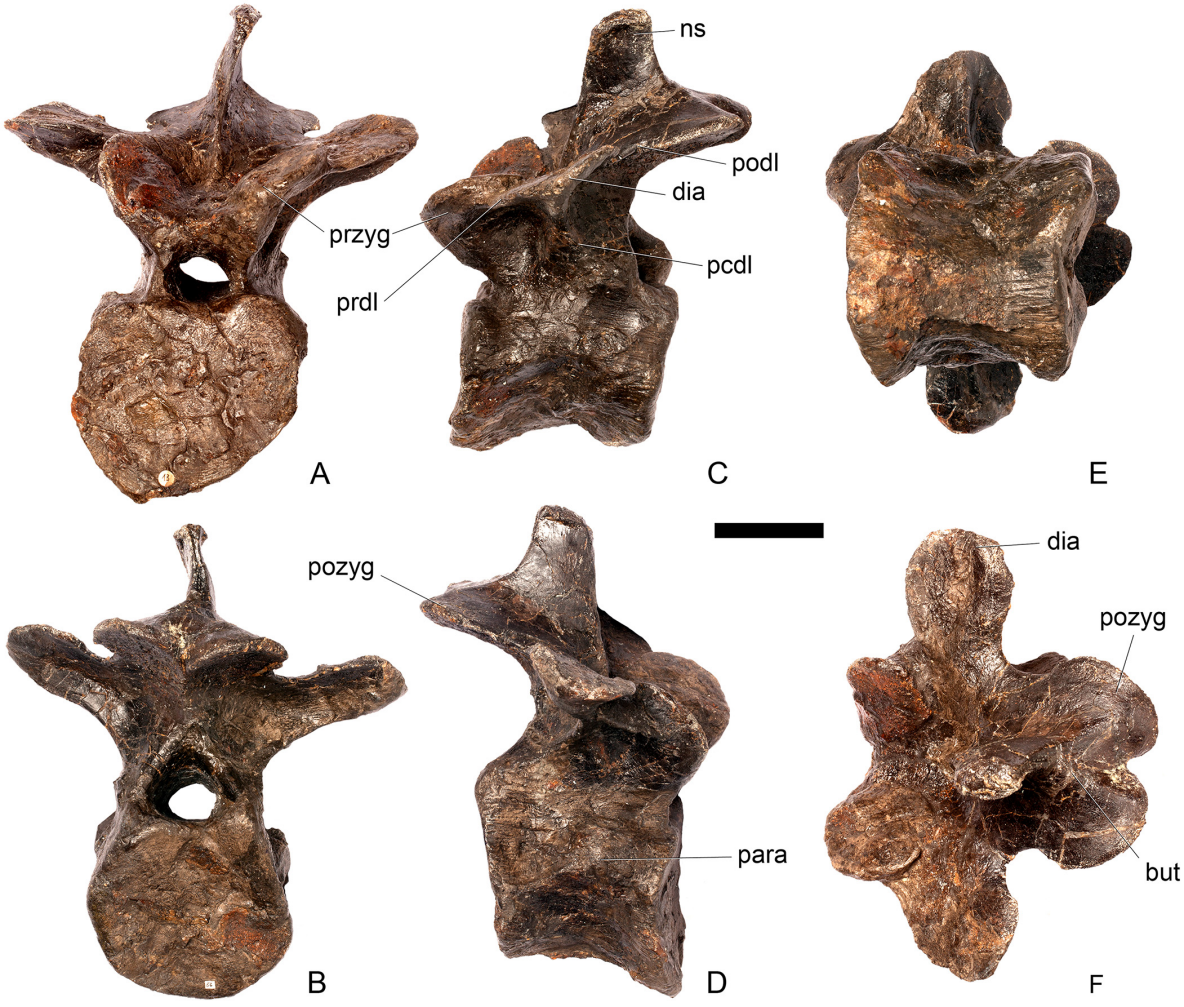
Cervical vertebra 13. **A**, anterior, **B**, posterior, **C**, left lateral, **D**, right lateral, **E**, ventral and **F**, dorsal view. **But**, buttress; **dia**, diapophysis; **ns**, neural spine; **para**, parapophysis; **pcdl**, posterior centrodiapophyseal lamina; **podl**, postzygodiapophyseal lamina; **pozyg**, postzygapophysis; **prdl**, prezygodiapophyseal lamina; **przyg**, prezygapophysis. Scale bar equal to 5 cm.

Cv13 is the last vertebra to bear a cervical rib and is in many respects transitional in morphology between the cervical and dorsal vertebrae. In overall morphology it is more similar to Cv17 of *Dacentrurus* sp. (ML 433) than it is to Cv13 of the latter taxon. The centrum of Cv13 in NHMUK PV R36730 is slightly sheared anteroposteriorly and transversely so that it has a parallelogram-shaped outline in lateral view. In anterior view the articular surface has suffered some damage and has been skimmed with plaster so its original shape is difficult to determine. The surface appears to be flat, lacking the dorsal bulge seen in the preceding vertebrae, although this could be due to damage (Fig 17A). The posterior articular surface appears to have been sub-circular in outline and very gently concave (Fig 17B). The lateral surfaces of the centrum are anteroposteriorly concave and divided into a small dorsal and much more extensive ventral portion by a longitudinal ridge, as in the preceding cervicals. On the right, the parapophysis is a large irregular swelling set posterior to the anterior margin of the centrum in lateral view (Fig 17D); the centrum is damaged on the left side in this area. The ventral margin of the centrum has been slightly eroded and it is not clear if a keel was present.

The neurocentral suture cannot be seen because the area is covered in plaster on both sides. In anterior view the neural canal is circular in outline (Fig 17A) and in posterior view it is teardrop-shaped with the apex pointing dorsally, as in the preceding cervicals (Fig 17B). The prezygapophyses are inclined dorsolaterally with articular surfaces facing dorsomedially at an angle of approximately 45 degrees to vertical (Fig 17A). This angle is greater than in the preceding vertebrae, and results in the dorsal margins of the prezygapophyses lying at almost the same level as the diapophyses rather than projecting dorsally to them. The articular facets of the prezygapophyses are ovate in outline and are longer anteroposteriorly than they are broad dorsoventrally, as in the other cervical vertebrae. The surfaces are flat to gently convex (Fig 17F). The bases of the prezygapophyses are closer to each other than in the preceding vertebrae with a consequent narrowing of the intraprezygapophyseal shelf (Fig 17A). The anterior margin of the neural arch ventral to the prezygapophyses is almost flush with the anterior margin of the centrum in lateral view (Fig 17C and 17D), in contrast with the preceding vertebrae, where it is set back from the anterior margin of the centrum, but similar to the posterior cervical vertebrae of *Loricatosaurus* (MHNH(BR) 001; [32]: pl. 1), *Kentrosaurus* (MB R.4787) and *Huayangosaurus* (ZDM T7001; [31]: fig 11). The neural arch of the last cervical of *Loricatosaurus* (MHNH(BR) 001; [32]: pl.1), *Huayangosaurus* (ZDM T7001; [31]: fig 11), and the posterior cervicals in *Kentrosaurus* (MB R.4786, R.4787; [33]: fig 13) is more dorsally elongate than that of NHMUK PV R36730, however. The anterior margin of the neural spine of NHMUK PV R36730 extends almost vertically in lateral view and unlike in the preceding vertebrae the base of the neural spine is positioned posterior to the posterior margin of the prezygapophyses (Fig 17C and 17D). The neural spine is more elongate than in other cervicals, is sub-rectangular in outline in lateral view, and has an almost straight posterior margin (Fig 17C and 17D). As in the preceding vertebrae the spine remains a transversely compressed plate whose apex bears a slight transverse expansion (Fig 17A and 17B). In *S*. *mjosi* (DMNH 29431) the neural spine of Cv12 is developed to a similar degree to that of Cv13 in NHMUK PV R36730. As in Cv12, the postzygapophyses are fused for most of their length in dorsal view, and are separated only by a shallow V-shaped notch at their distal ends. Subtle rounded buttresses extend along the dorsal margins of the postzygapophyses to merge with the posterior margin of the spine (Fig 17F). The articular surfaces of the postzygapophyses are gently concave transversely. They are elliptical in outline, being longer anteroposteriorly than they are wide transversely, and are relatively broader transversely than in the preceding vertebrae. Postzygapophyses extend for only a short distance beyond the neural arch in lateral view, in contrast to the preceding vertebrae, where they overhang the posterior margin of the centrum (Fig 17C and 17D). Postzygapophyses in the last cervical of *Loricatosaurus* (MHNH(BR) 001; [32]: pl. 1) and *Huayangosaurus* (ZDM T7001; [31]: fig 11) do not significantly overhang the posterior articular facet. In dorsal view, the diapophyses expand anteroposteriorly as they extend laterally and terminate in a bluntly rounded apex (Fig 17F). The diapophyses extend strictly laterally. In lateral view, the diapophyses are sub-triangular in cross-section with the apex pointing ventrally. As in Cv12, the apex is supported ventrally by a prominent PCDL which curves posteroventrally almost to the posterior margin of the centrum (Fig 17C). A PCDL can also be observed in this location in the final cervical of *Loricatosaurus* (MHNH(BR) 001; [32]: pl. 1), *Huayangosaurus* (ZDM T7001) and in *Kentrosaurus* (MB R.4787). In lateral view a distinct PRDL connects the lateral surface of the prezygapophysis with the anterior margin of the diapophysis (Fig 17C). The PRDL, prezygapophyses and anterior margin of the diapophysis frame two distinct fossae, one dorsal to the PRDL and a shallower but more extensive fossa ventral to the PRDL. Similarly, the PCDL, posterior margin of the diapophysis and PODL frame an extensive fossa on the posterodorsal surface of the neural arch that faces posterolaterally (Fig 17B).

#### Dorsal vertebra 1 (Fig 18)

In overall morphology, the dorsal (D) vertebrae are very similar to those of *Loricatosaurus* (NHMUK PV R3167; MHNH(BR) 001; [29]: fig 14A–R; [32]: pl. 2).

**Fig 18:**
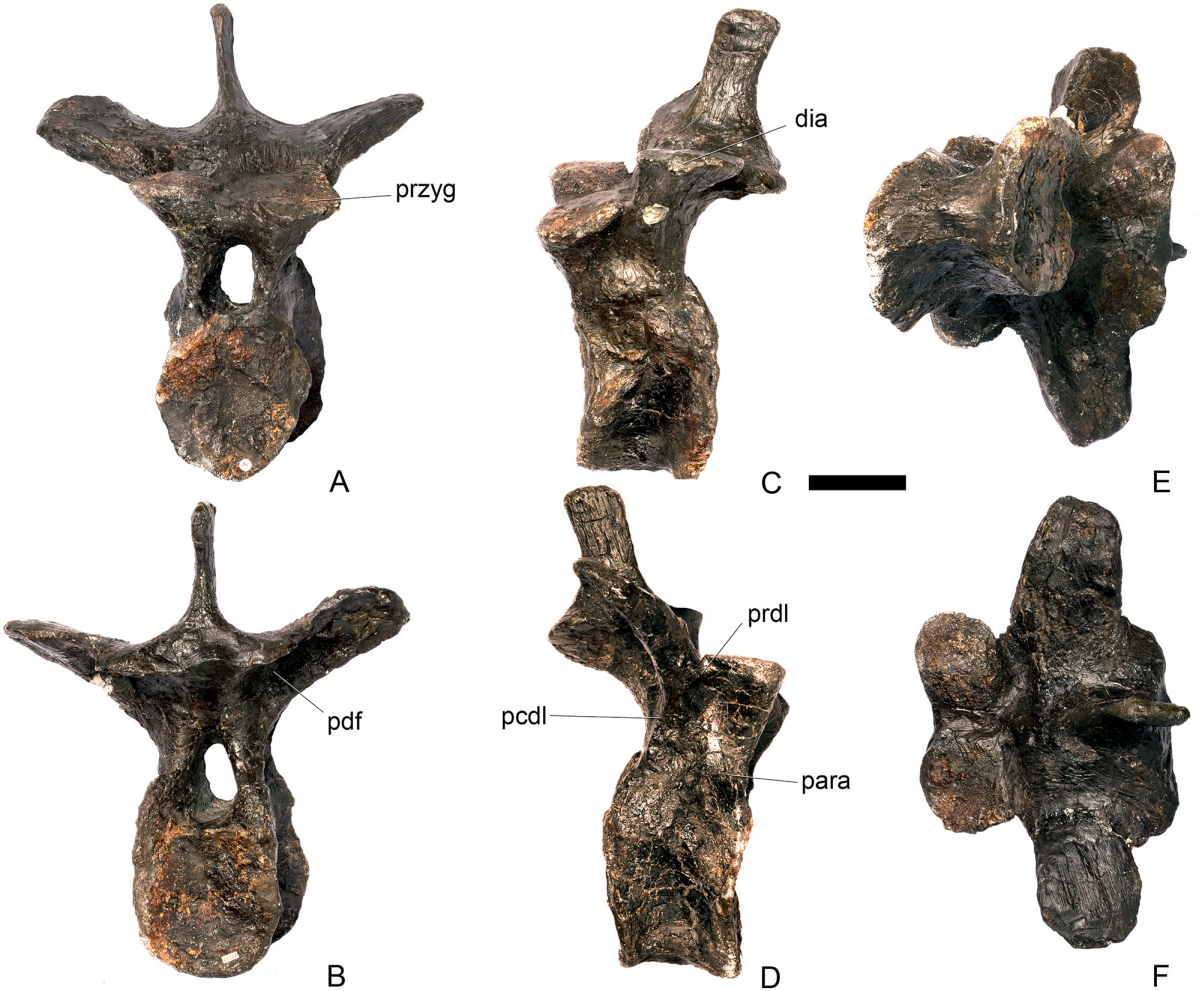
Dorsal vertebra one. **A**, anterior, **B**, posterior, **C**, left lateral, **D**, right lateral, **E**, ventral and **F**, dorsal view. **Dia**, diapophysis; **pcdl**, posterior centrodiapophyseal lamina; **pdf**, posterior fossa on diapophysis; **prdl**, prezygodiapophyseal lamina; **przyg**, prezygapophysis. Scale bar equal to 5 cm.

The centrum of D1 is obliquely sheared such that the right side projects further anteriorly than the left hand side. Ventrally, the centrum is crushed and plaster covers the surface, so the presence or absence of a keel cannot be determined. The neural spine, postzygapophyses, the left diapophysis and the area between the prezygapophyses and the base of the spine have been extensively reconstructed.

The centrum is slightly longer anteroposteriorly than it is tall dorsoventrally or wide transversely, and this is true of all of the dorsals, as in *Loricatosaurus* (NHMUK PV R3167; [29]: fig 14A–R), *Kentrosaurus* [33] and *Huayangosaurus* [31]. The opposite is true in *S*. *mjosi* (DMNH 29431), *Dacentrurus armatus* (NHMUK OR46013; [29]: fig 2E, F) and *Chungkingosaurus* (CV 206); the dorsal centra are shorter anteroposteriorly than they are tall dorsoventrally or wide transversely. The anterior articular facet of the centrum is sub-circular in outline and flat, with a central concavity that might be due to erosion (Fig 18A). This contrasts with the condition in *Chungkingosaurus* (CV 206), where the anterior articular facet is convex and the centrum is opisthocoelous, a feature not previously noted by either [34] or [35]. The posterior articular facet is also sub-circular in outline and flat with a small central pit (Fig 18B). The lateral surfaces of the centrum are not well preserved, but appear to be anteroposteriorly concave. In *Gigantspinosaurus* (ZDM 0019) the lateral surfaces of the centra ventral to the neurocentral suture are more deeply excavated, leading [27]: 233 to describe them incorrectly as pleurocoels.

The neurocentral suture is not visible on either side, although on the left this area is covered with plaster. In anterior and posterior view, the neural canal is oval and taller dorsoventrally than it is wide transversely, although the posterior opening is larger than the anterior one (Fig 18A and 18B). In *Kentrosaurus* (MB R.1930, R.1931; [36]: pl. 3.11–15), the neural canals of all dorsals are much larger than in NHMUK PV R36730, expanding to fill the whole of the neural arch from the top of the centrum to the base of the prezygapophyses [33]. On the right side in lateral view a rugose swelling is present on the anteroventral margin of the neural arch and anterodorsal margin of the centrum. This rugosity represents the parapophysis, which has migrated dorsally relative to its position in the cervical vertebrae (Fig 18D). This area is reconstructed on the left side. Prezygapophyses are stout processes that project dorsally from the anterolateral margins of the neural canal. The articular surfaces of the prezygapophyses are sub-circular and slightly convex in dorsal view (Fig 18F). They face dorsally, being oriented horizontally in anterior view (Fig 18A). The prezygapophyses of *Chungkingosaurus* (CV 206) are relatively smaller and are separated from the diapophyses by a much smaller gap than in NHMUK PV R36730. The base of the right diapophysis is preserved, but the tip is reconstructed. The diapophysis projects dorsolaterally and is L-shaped in lateral cross-section, with a short anteroventral margin and elongate posterodorsal margin (Fig 18D). The anteroventral margin is continuous with a prominent PCDL that extends posteroventrally (Fig 18D). It does not reach the level of the neurocentral suture, but the vertebra is damaged in this region. The PCDL and diapophysis bound a deep posterolaterally facing fossa that extends for a short distance along the posteroventral surface of the diapophysis (Fig 18B). The anteroventral margin of the diapophysis and posteroventral margin of the prezygapophysis bound a shallow fossa (Fig 18D). Dorsal to this fossa is a very short PRDL, which is much reduced relative to that in Cv13. The PRDL extends to the posterior margin of the prezygapophysis rather than extending across its lateral surface (Fig 18D).

#### Dorsal vertebra 2 (Fig 19)

**Fig 19:**
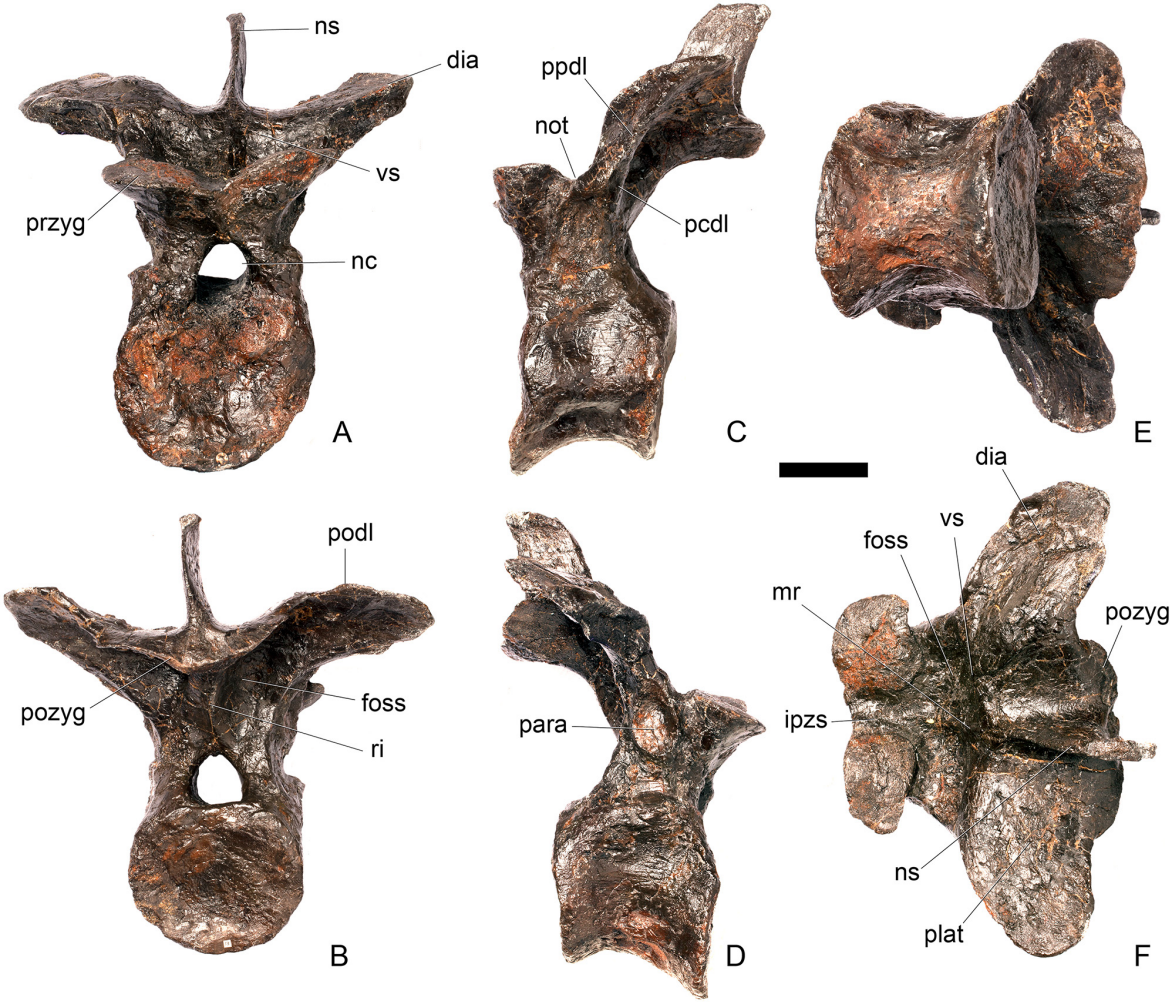
Dorsal vertebra two. **A**, anterior, **B**, posterior, **C**, left lateral, **D**, right lateral, **E**, ventral and **F**, dorsal view. **Dia**, diapophysis; **foss**, fossa; **ipzs**, intraprezygapophyseal shelf; **mr**, midline ridge; **nc**, neural canal; **not**, notch; **ns**, neural spine; **para**, parapophysis; **pcdl**, posterior centrodiapophyseal lamina; **plat**, platform; **podl**, postzygodiapophyseal lamina; **pozyg**, postzygapophysis; **ppdl**, paradiapophyseal lamina; **prdl**, prezygodiapophyseal lamina; **przyg**, prezygapophysis; **ri**, ridge; **vs**, vertical sheet. Scale bar equal to 5 cm.

D2 is well preserved. In anterior view the anterior articular surface of the centrum is sub-elliptical, being wider transversely than it is tall dorsoventrally, and gently concave (Fig 19A). The posterior surface is sub-circular in outline, also gently concave, and is slightly smaller in overall dimensions than the anterior surface (Fig 19B). In lateral view the centrum is anteroposteriorly concave. Ventrally, there is no midline keel and the ventral surface is broad, merging gently into the lateral surfaces (Fig 19E).

The neurocentral suture cannot be distinguished but this area is poorly preserved. In anterior view the neural canal is sub-elliptical with the long axis vertical; in posterior view it is teardrop-shaped with the apex pointing dorsally and smaller than in anterior view. The prezygapophyses are supported on stout pedicles, and the distance between the dorsal margins of the prezygapophyses and dorsal margin of the neural canal is increased in depth relative to preceding presacrals. This has the effect of increasing the height of the neural arch. In anterior view the prezygapophyses are orientated almost horizontally with their articular surfaces facing dorsally, as in D1 (Fig 19A and 19B). In contrast, the prezygapophyses of the anterior dorsals of *Huayangosaurus* (ZDM T7001; [31]: figs 13–17) are angled dorsomedially. In dorsal view the articular surfaces of the prezygapophyses are ovate in outline and gently convex; they are separated along the midline by a gently concave area representing a much reduced intraprezygapophyseal shelf (Fig 19F). Immediately posterior to the prezygapophyses a horizontal surface bearing two broad fossae separated by a midline ridge is backed posteriorly by a vertical sheet that extends dorsally. The midline ridge extends dorsally on this vertical surface, eventually merging with the anterior surface of the neural spine (Fig 19A and 19F). The dorsal margins of the diapophyses, the postzygapophyses and the neural spine arise from a continuous, flat, horizontally-inclined platform extending posteriorly from this vertical sheet (Fig 19F). These features give the neural arch a ‘stepped’ morphology in lateral view and result in an increase in height of the neural arch relative to preceding vertebrae (Fig 19C and 19D). The vertical sheet, ‘stepped’ morphology, and consequent elongation of the neural arch are absent in the dorsals of *Huayangosaurus* (ZDM T7001; [31]: figs 13–17) in which the prezygapophyses and postzygapophyses are located at the same level in lateral view. In lateral view the PRDL of NHMUK PV R36730 is absent and the prezygapophysis is separated from the rest of the neural arch by a distinct notch (Fig 19C). The parapophyses have migrated entirely onto the lateral surface of the neural arch and are situated posterior and slightly ventral to the prezygapophyses. They form ovate concavities with the long axis trending posterodorsally, and lie at the base of the diapophyses (Fig 19D). In anterior view the diapophyses project laterally and are situated dorsal to the level of the prezygapophyses (Fig 19A). In dorsal view they extend posterolaterally maintaining a constant width along their length prior to terminating in a bluntly rounded apex (Fig 19F). In lateral view the diapophyses have an L-shaped cross-section. The anterior margin of the diapophysis is gently excavated forming a shallow trough that extends laterally. This trough is bounded ventrally by a sharp buttress forming the ventral margin of the diapophysis. This buttress bifurcates ventrally forming a prominent PCDL which extends to the centrum, and a faint but distinct ridge that extends to the anterodorsal margin of the parapophysis, in the equivalent position of the paradiapophyseal lamina (PPDL; [30]; Fig 19C). The posterior margin of the diapophysis, the PCDL and the postzygapophyses frame a deep and extensive posteriorly-facing fossa (Fig 19B). In posterior view, the diapophyses terminate above the level of the postzygapophyses, in contrast with the condition in the cervical vertebrae, where the postzygapophyses were dorsal to or level with the diapophyses (Fig 19B). The posterior margins of the diapophyses are confluent with the posterior margin of the postzygapophyses, forming a postzygodiapophyseal lamina (PODL; [30]; Fig 19B). In posterior view the two large fossae on the posterior surface of the neural arch are separated by a low midline ridge which supports the postzygapophyses ventrally (Fig 19B). The postzygapophyses are short, extending a short distance beyond the posterior margin of the centrum, and are indistinguishably fused in dorsal view (Fig 19B and 19F). In ventral view the articular facets of the postzygapophyses are transversely concave, have elliptical outlines and face ventrally. Ventrally, the articular surfaces of the postzygapophyses remain separate from each other, although they are linked by a midline sheet of bone. In posterior view subtle buttresses extend from the top of the postzygapophyses to merge with the base of the neural spine (Fig 19B). The neural spine is sub-triangular in outline with gently convex anterior and dorsal margins and a straight to slightly concave posterior margin (Fig 19D). The neural spine is a transversely compressed plate with a transversely expanded tip (Fig 19A). The neural spine of *Dacentrurus* sp. (ML 433) differs from that of NHMUK PV R36730 in that it is lobate in lateral view, with a rounded dorsal margin, and it bears an anteriorly directed flange about halfway up its length. It appears to project more strictly dorsally than that of NHMUK PV R36730, which is slightly posteriorly inclined.

#### Dorsal vertebra 3 (Fig 20)

**Fig 20:**
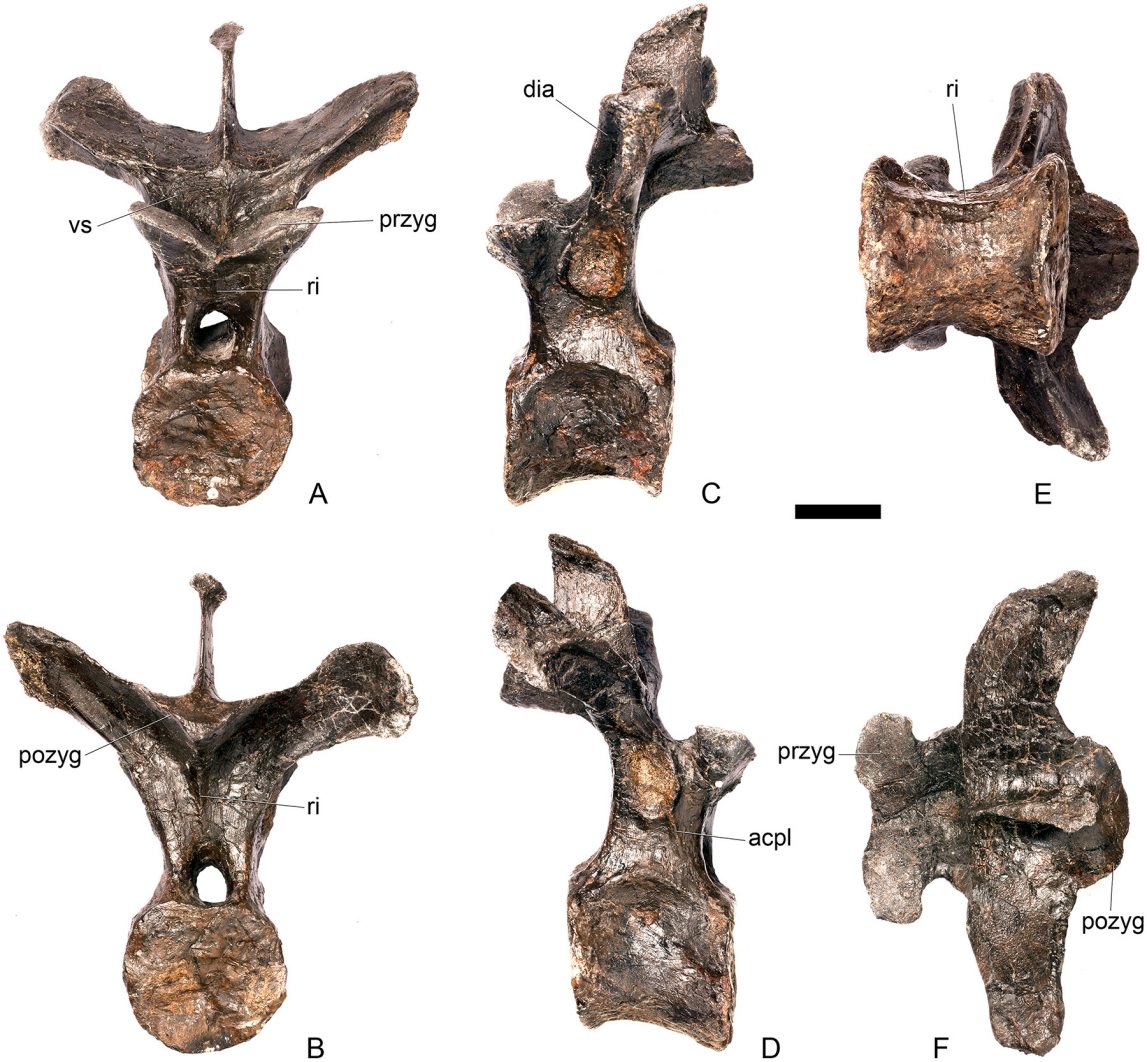
Dorsal vertebra three. **A**, anterior, **B**, posterior, **C**, left lateral, **D**, right lateral, **E**, ventral and **F**, dorsal view. **Acpl**, anterior centroparapophyseal lamina; **pozyg**, postzygapophysis; **przyg**, prezygapophysis; **ri**, ridge; **vs**, vertical sheet. Scale bar equal to 5 cm.

D3 is similar in morphology to D2 in most respects, but differs from it in the following ways. On the centrum, a faint ridge extends from the posterior margin anteriorly for three-quarters of the length of the centrum and separates the lateral and ventral surfaces (Fig 20E). In anterior view, a faint ridge arises at the top of the neural canal and extends dorsally to the point where the prezygapophyses meet on the midline (Fig 20A). As in preceding dorsals, the prezygapophyses are supported on pedicles that are convex laterally but are further elevated in height relative to the position of the neural canal. In contrast to D1 and D2, the articular surfaces of the prezygapophyses are sub-rectangular with the long axis transverse, face dorsomedially, and are confluent on the midline so that the intraprezygapophyseal shelf is entirely absent (Fig 20F). The parapophyses are similar in morphology and location to those of D2, except that a ridge extends from the anteroventral corner of the parapophysis anteroventrally towards the neurocentral suture, eventually merging with the neural arch before it reaches the presumed location of the suture itself. This ridge is in the equivalent position to the anterior centroparapophyseal lamina (ACPL) of sauropods [30] (Fig 20D). Diapophyses are sub-triangular in cross-section with the apex pointing ventrally. The anterior margins of the diapophyses form sharp ridges that extend medially meeting on the midline at the base of the neural spine (Fig 20D). This feature marks the boundary between the vertically inclined sheet arising posterior to the prezygapophyses and the horizontally inclined dorsal surface of the rest of the neural arch (Fig 20A). In posterior view, the postzygapophyses form a V-shaped wedge and a subtle ridge extends from the dorsal margin of the neural canal dorsally to meet the ventral apex of the wedge (Fig 20B). Dorsally, the postzygapophyses are undivided and anteroposteriorly short (Fig 20F). The articular surfaces of the postzygapophyses face ventrolaterally, are sub-triangular, and and meet on the midline ventrally (Fig 20B).

#### Dorsal vertebra 4 (Fig 21)

**Fig 21:**
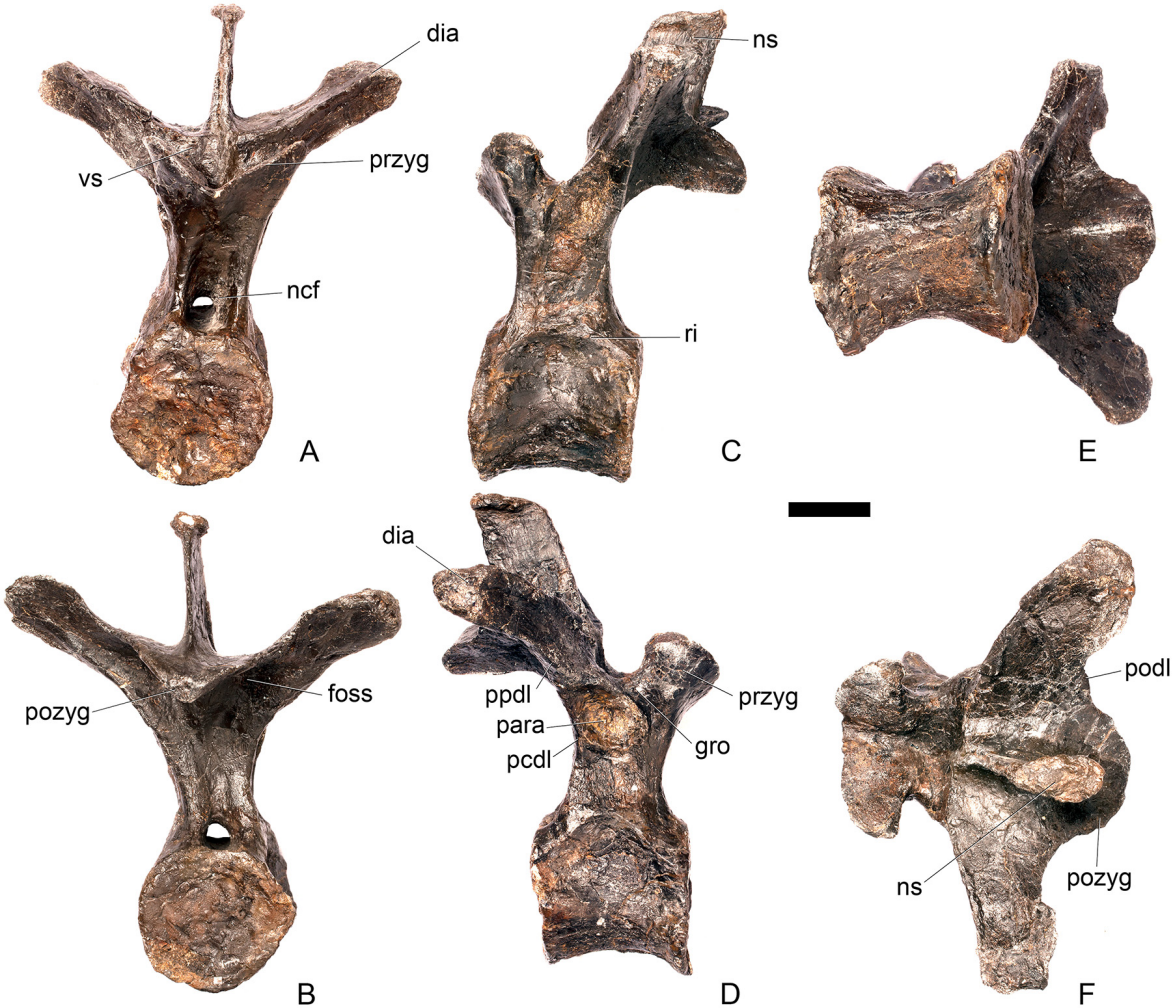
Dorsal vertebra four. **A**, anterior, **B**, posterior, **C**, left lateral, **D**, right lateral, **E**, ventral and **F**, dorsal view. **Dia**, diapophysis; **foss**, fossa; **ncf**, fossa surrounding neural canal; **ns**, neural spine; **para**, parapophysis; **pcdl**, posterior centrodiapophyseal lamina; **podl**, postzygodiapophyseal lamina; **pozyg**, postzygapophysis; **ppdl**, paradiapophyseal lamina; **przyg**, prezygapophysis; **ri**, ridge; **vs**, vertical sheet. Scale bar equal to 5 cm.

D4 is well preserved. The centrum is almost amphiplatyan with only a slight depression in the ventral part of the anterior articular surface. Both the anterior and posterior articular surfaces are sub-circular in outline (Fig 21A and 21B). In lateral view, the surfaces of the centrum are gently longitudinally concave and dorsoventrally convex (Fig 21C and 21D). A distinct keel is absent ventrally, with the lateral surfaces blending into a gently convex ventral surface (Fig 21E). In ventral view the centrum is slightly waisted.

In lateral view the base of the neural arch bears a raised upwardly convex ridge forming a parabola-like structure. The area immediately below this ridge is strongly concave and merges with the lateral surface of the centrum, whereas the area above it is convex and merges dorsally into the surface of the neural arch (Fig 21C). The neurocentral suture is visible as a faint line on both sides of the vertebra but appears to be fused.

In anterior view the neural canal has an elongate elliptical outline with the long axis of the ellipse extending dorsoventrally; however, the neural canal narrows posteriorly so that the actual opening is considerably smaller than the fossa surrounding it (Fig 21A). In posterior view the neural canal is sub-ovate and is not surrounded by the extensive fossa seen in anterior view (Fig 21B). In lateral view the neural arch has increased in height relative to preceding dorsals, caused primarily by extension of the neural arch pedicles and dorsal extension of the portion of the neural arch bearing the diapophyses and postzygapophyses (Fig 21C and 21D). The parapophysis is situated anteroventral to the base of the diapophysis and is separated from the base of the prezygapophysis by a shallow groove (Fig 21D). The parapophysis has an ovate outline with its long axis trending posterodorsally. It is deeply concave and surrounded by a raised rim. Its anterior margin is continuous with a faint but distinct ACPL, and its posterior margin is confluent with a buttress-like PCDL. Its posterodorsal corner is linked to the tip of the diapophysis by a robust PPDL (Fig 21D). In anterior view, the prezygapophyses diverge from each other at an angle of approximately 40 degrees from vertical (Fig 21A). The prezygapophyses are borne on stout pedicles; the articular surfaces are sub-triangular in outline and flat, and the pedicles have a sub-triangular transverse cross-section (Fig 21F). The area posterior to the prezygapophyses bears a shallow midline concavity which turns upwards at approximately 90 degrees to form a vertical sheet bearing a faint midline ridge that is continuous with the neural spine dorsally (Fig 21A and 21F). In anterior view the diapophyses extend dorsolaterally, forming an angle of approximately 40 degrees to vertical. The anterior margins of the diapophyses bear a prominent lamina that extends to the base of the anterior margin of the neural spine (Fig 21A). In lateral view the diapophyses have a sub-triangular cross-section; their posterior margin forms a web of bone that links with the postzygapophyses to form a PODL (Fig 21C and 21D). The area between the PODL and the PCDL is excavated by a deep fossa. In posterior view the postzygapophyses are angled at approximately 40 degrees from horizontal and are confluent on the midline forming a V-shaped wedge. The midline of this structure extends into a short, sharp ridge that merges into the neural arch pedicel a short distance ventrally and does not reach the dorsal margin of the neural canal (Fig 21B). The articular facets of the postzygapophyses are large, gently concave and have a sub-ovate outline (Fig 21E). The neural arch pedicle dorsal to the neural canal opening is gently concave transversely. In lateral view the neural spine is sub-rectangular with straight anterior, dorsal and posterior margins (Fig 21D). In anterior view it is a transversely compressed plate with a slightly expanded dorsal spine table (Fig 21A). The anterior margin of the spine bears a shallow groove and is slightly broader than the narrow posterior margin. In dorsal view the spinal table has an elongate, oval outline (Fig 21F). The spine projects dorsally and slightly posteriorly. The postzygapophyses considerably overhang the posterior margin of the centrum. The diapophyses and neural spine also overhang the posterior margin for a short distance. In comparison the prezygapophyses project only slightly beyond the anterior margin of the centrum (Fig 21C and 21D).

#### Dorsal vertebra 5 (Fig 22)

**Fig 22:**
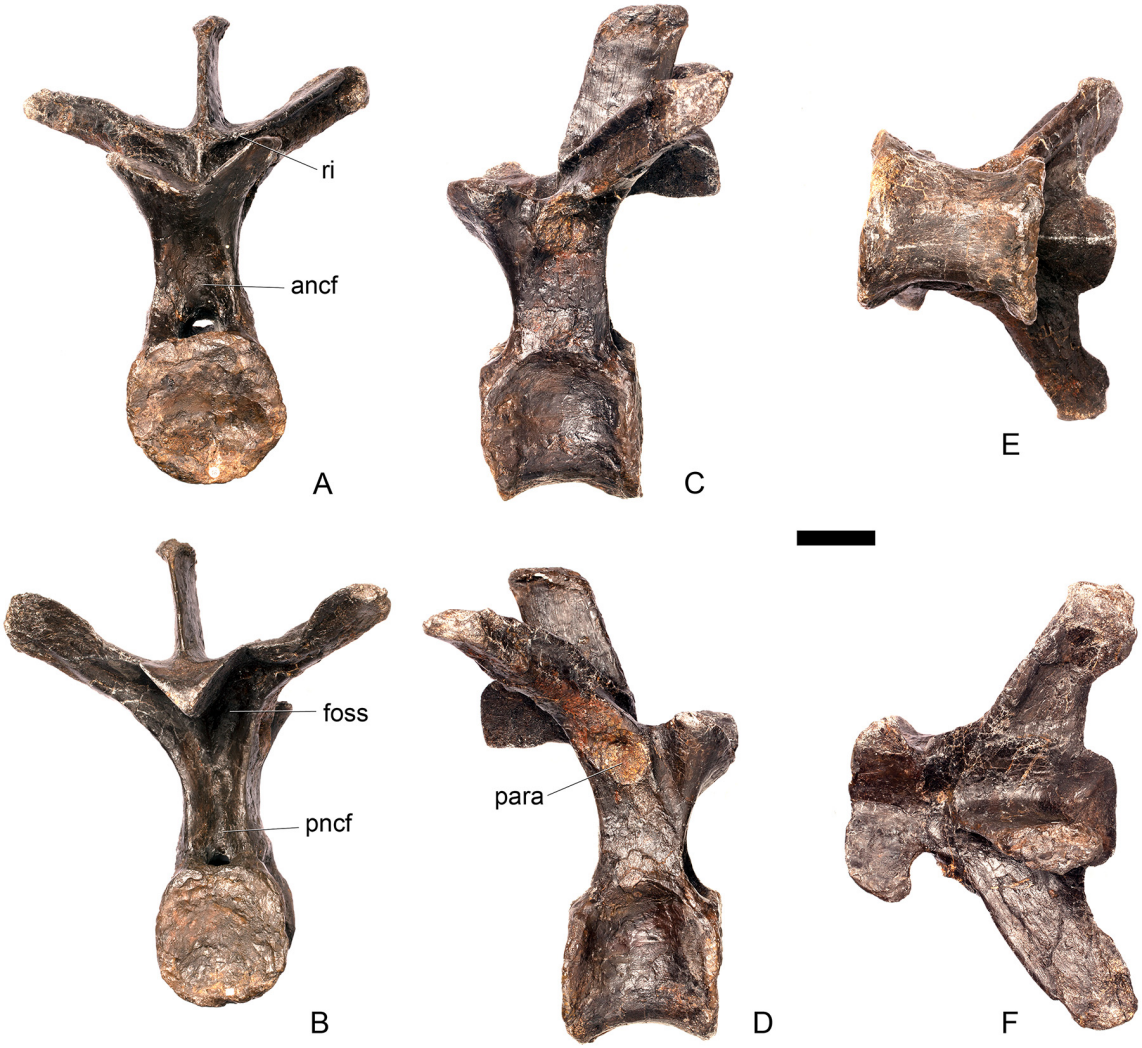
Dorsal vertebra five. **A**, anterior, **B**, posterior, **C**, left lateral, **D**, right lateral, **E**, ventral and **F**, dorsal view. **Ancf**, fossa surrounding neural canal on anterior surface; **foss**, fossa; **para**, parapophysis; **pncf**, fossa surrounding neural canal on posterior surface; **ri**, ridge. Scale bar equal to 5 cm.

D5 is well preserved although the neural spine is reconstructed. It is very similar in morphology to D4, differing only in the following respects. In both anterior and posterior views, the neural canal is situated in a dorsoventrally elongate fossa that extends further dorsally than the neural canal opening (Fig 22A and 22B). Although a fossa is also present anteriorly on D4, it is much more pronounced on D5. In *S*. *homheni* (IVPP V4006), this fossa is particularly pronounced, and is bounded by sharp laminae that extend dorsally from the lateral margins of the neural canal and meet below the prezygapophyses [18]. In lateral view, the parapophysis, which is similar in morphology to that of D4, has migrated slightly dorsally on the neural arch so that it is located at the same level as the prezygapophyses (Fig 22C and 22D). Ridges linking the anterior surfaces of the diapophyses to the base of the anterior margin of the neural spine are more prominent in D5 than they were in D4 (Fig 22A). In posterior view, lateral to the postzygapophyses, the neural arch is shallowly excavated to form two dorsoventrally elongate fossae that become confluent ventral to a midline ridge extending ventrally from the postzygapophyses. Further ventrally still, this fossa becomes confluent with the fossa surrounding the neural canal (Fig 22B).

#### Dorsal vertebra 6 (Fig 23)

**Fig 23:**
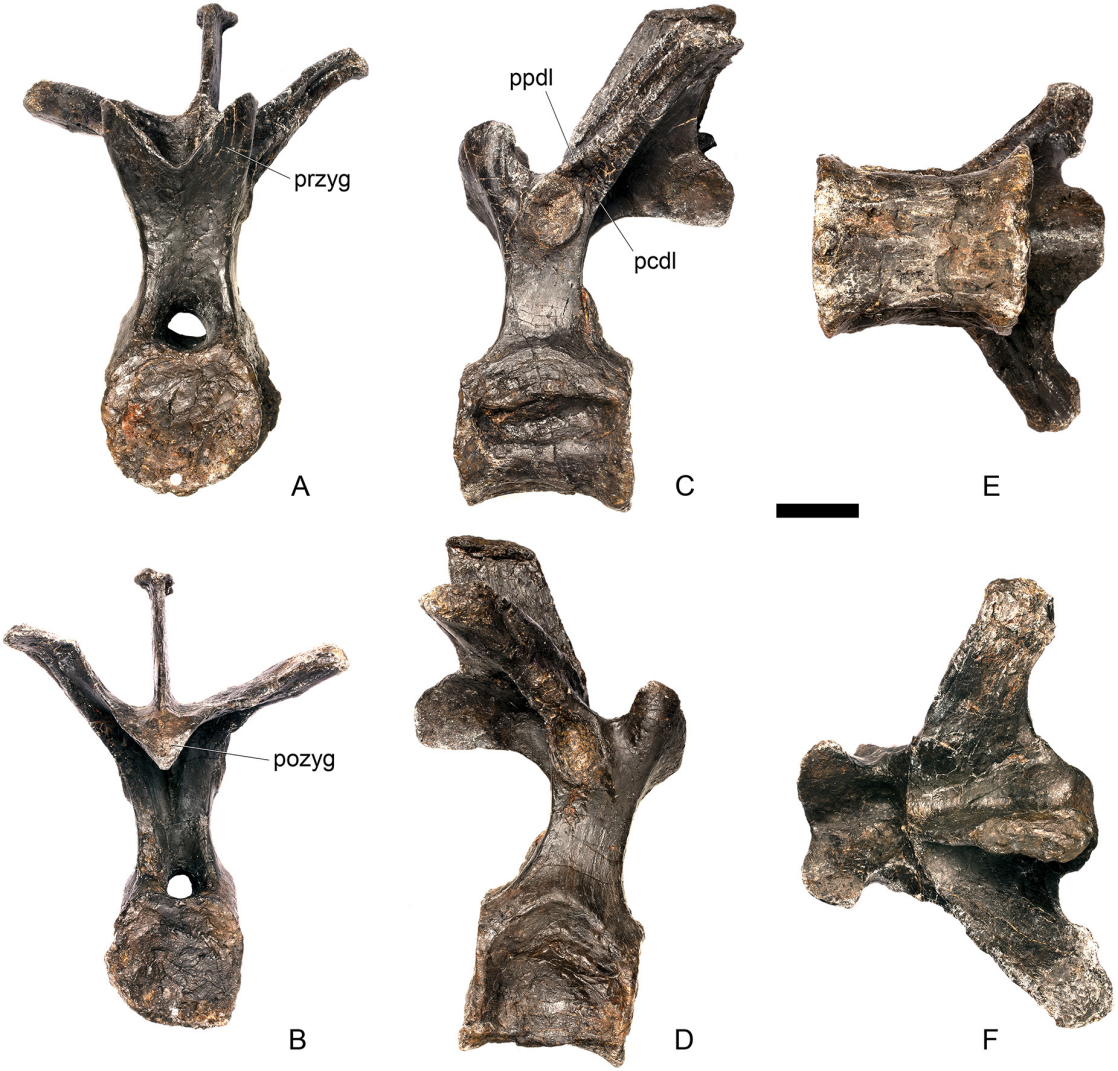
Dorsal vertebra six. **A**, anterior, **B**, posterior, **C**, left lateral, **D**, right lateral, **E**, ventral and **F**, dorsal view. **Pcdl**, posterior centrodiapophyseal lamina; **pozyg**, postzygapophysis; **ppdl**, paradiapophyseal lamina; **przyg**, prezygapophysis. Scale bar equal to 5 cm.

D6 has suffered some crushing of the centrum that has produced an artificial groove on the left-hand side. The neural spine and at least part of the right prezygapophysis are reconstructed. The morphology of D6 is essentially identical to that of D4 and D5 with the following exceptions. The neural arch pedicles beneath the parapophyses are taller than in the preceding vertebrae, thereby increasing the elongation of the neural arch (Fig 23C and 23D). This elongation of the neural arch is not present in *S*. *mjosi* (DMNH 29431), *Huayangosaurus* (ZDM T7001; [31]: fig 15–17) or *Gigantspinosaurus* (ZDM 0019; [37]; [27]: fig 132d), so that prezygapophyses in all of the dorsals arise immediately dorsal to the neural canal. In contrast, elongation of the neural arch in *S*. *homheni* (IVPP V4006) appears to be greater than that in NHMUK PV R36730. In anterior view, the prezygapophyses are more steeply angled than in D5, with the articular facets forming an angle of 30 degrees to vertical (Fig 23A). In lateral view, the ACPL is very faint and essentially absent. The PCDL has bifurcated to form a clear PCDL and PPDL with a groove in between them (Fig 23C). In posterior view, the postzygapophyses are more steeply angled than in D5, forming an angle of around 40 degrees to vertical (Fig 23B). Fossae surrounding the neural canal openings in anterior and posterior views are present but shallower than they are in D5, although this may have been affected by restoration (Fig 23A and 23B).

#### Dorsal vertebra 7 (Fig 24)

**Fig 24:**
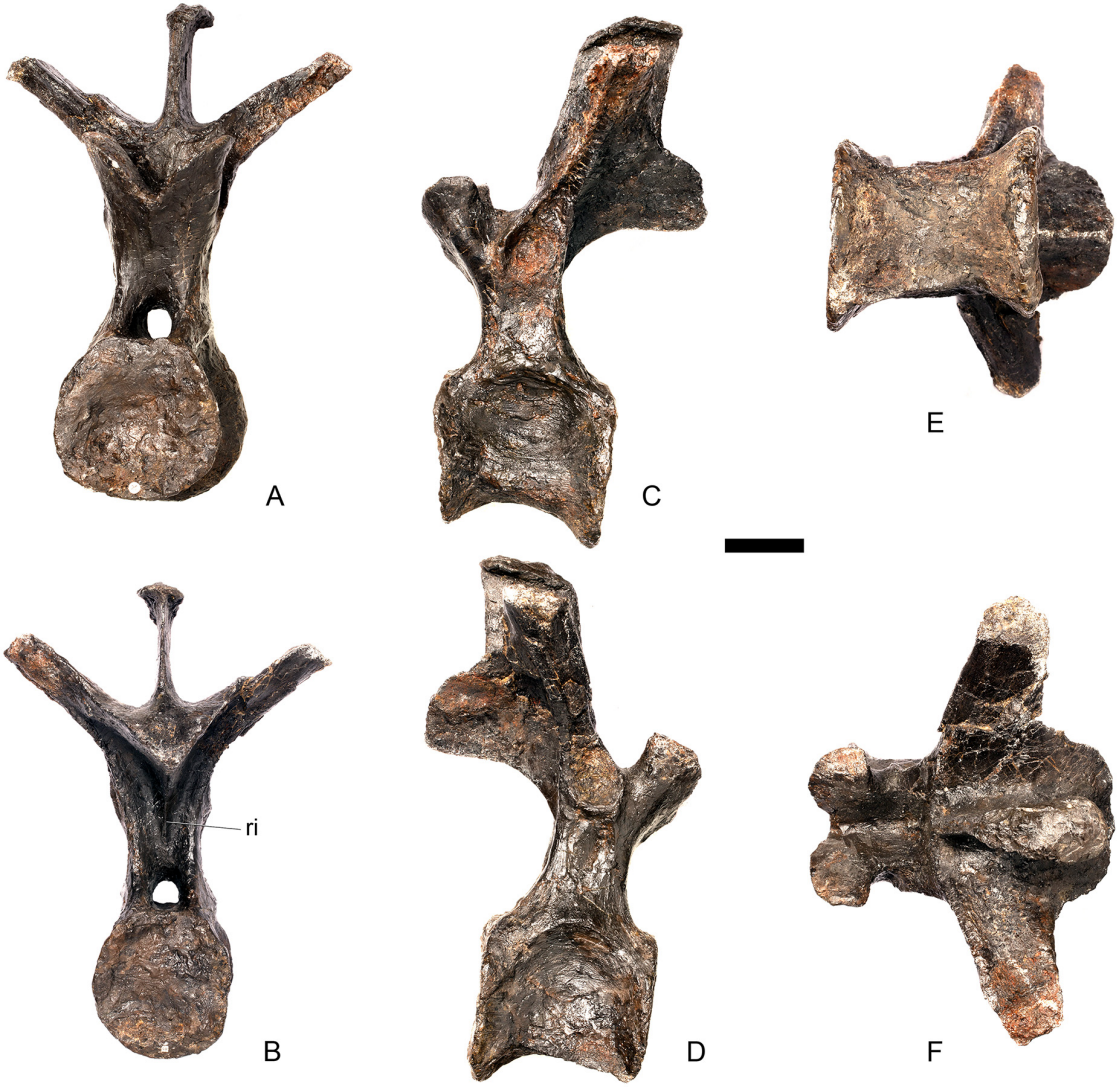
Dorsal vertebra seven. **A**, anterior, **B**, posterior, **C**, left lateral, **D**, right lateral, **E**, ventral and **F**, dorsal view. **Ri,** ridge. Scale bar equal to 5 cm.

The centrum of D7 is transversely crushed ventrally, and the top of the neural spine and distal ends of the diapophyses are reconstructed. However, it appears to be identical in most respects to D6. The only notable difference between the two is that the ridge ventral to the postzygapophyses extends further ventrally onto the neural arch in D7 than it does in D6 (Fig 24B).

#### Dorsal vertebra 8 (Fig 25)

**Fig 25:**
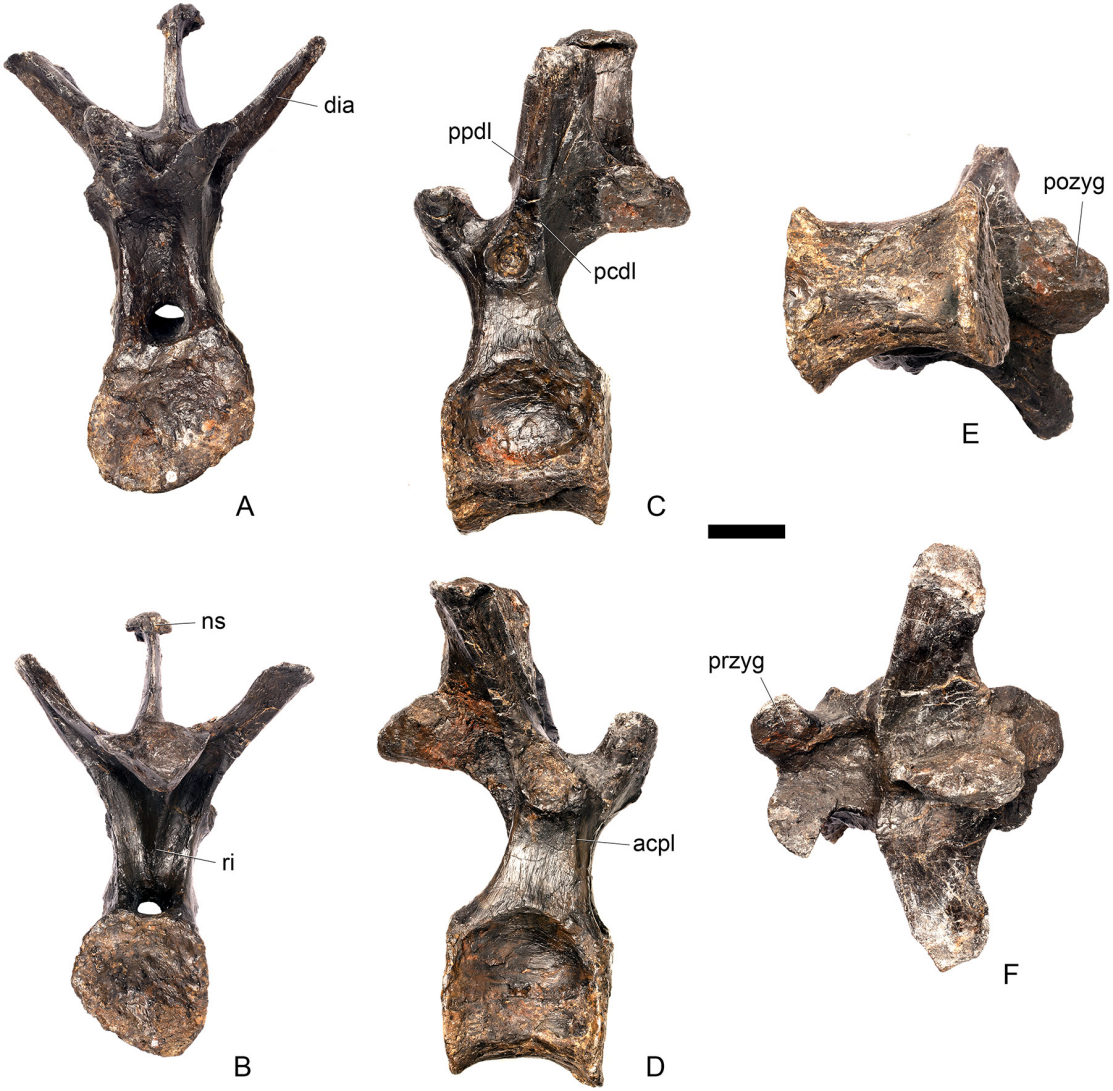
Dorsal vertebra eight. **A**, anterior, **B**, posterior, **C**, left lateral, **D**, right lateral, **E**, ventral and **F**, dorsal view. **Acpl**, anterior centroparapophyseal lamina; **dia**, diapophysis; **ns**, neural spine; **pcdl**, posterior centrodiapophyseal lamina; **pozyg**, postzygapophysis; **ppdl**, paradiapophyseal lamina; **przyg**, prezygapophysis; **ri**, ridge. Scale bar equal to 5 cm.

The centrum of D8 has been slightly deformed with some erosion of the ventral surface. Otherwise, the vertebra is essentially complete although the lower parts of the neural arch have been extensively skimmed with plaster. D8 is very similar to D6 and D7, differing from them only in the following respects. In lateral view, a faint ACPL can be seen anteroventral to the parapophysis, and there is slightly less separation between the PCDL and the PPDL (Fig 25C and 25D). The diapophyses are more steeply angled than they are on preceding vertebrae, at about 50 degrees from vertical. This results in the distal ends of the diapophyses being located at approximately the same level at the top of the neural spine (Fig 25A). [2] noted that the diapophyses reached their highest elevation in the mid-dorsal vertebrae in the holotype of *S*. *stenops* (USNM 4934) and this also appears to be true in *Dacentrurus armatus* (NHMUK OR46013). In *S*. *mjosi*, (DMNH 29431; [17]) the neural spine is much longer than the diapophyses, so that even at their highest elevation, the distal ends are not at the same level as the top of the spine. In *Kentrosaurus*, the diapophyses are elevated to a much greater degree than in NHMUK PV R36730, forming an angle of about 10 degrees to the vertical ([33]: figs 16–18; [34]: pl. 3), while in *Gigantspinosaurus* (ZDM 0019; [37]; [27]: fig 132d) the diapophyses are not elevated and remain more or less horizontal along the dorsal series. The top of the neural spine in NHMUK PV R36730 is more expanded transversely relative to the width of the spine relative to D6 and D7 (Fig 25A, 25B and 25F). The postzygapophyseal articular facets have increased in size relative to those of D6 and D7, and are roughly double the size of the prezygapophyses. The midline ridge extending ventrally from the postzygapophyses is increased in length, now reaching the top of the neural canal. Consequently, the fossa surrounding the neural canal in posterior view that was observed in D5–7 is absent (Fig 25B).

#### Dorsal vertebra 9 (Fig 26)

**Fig 26:**
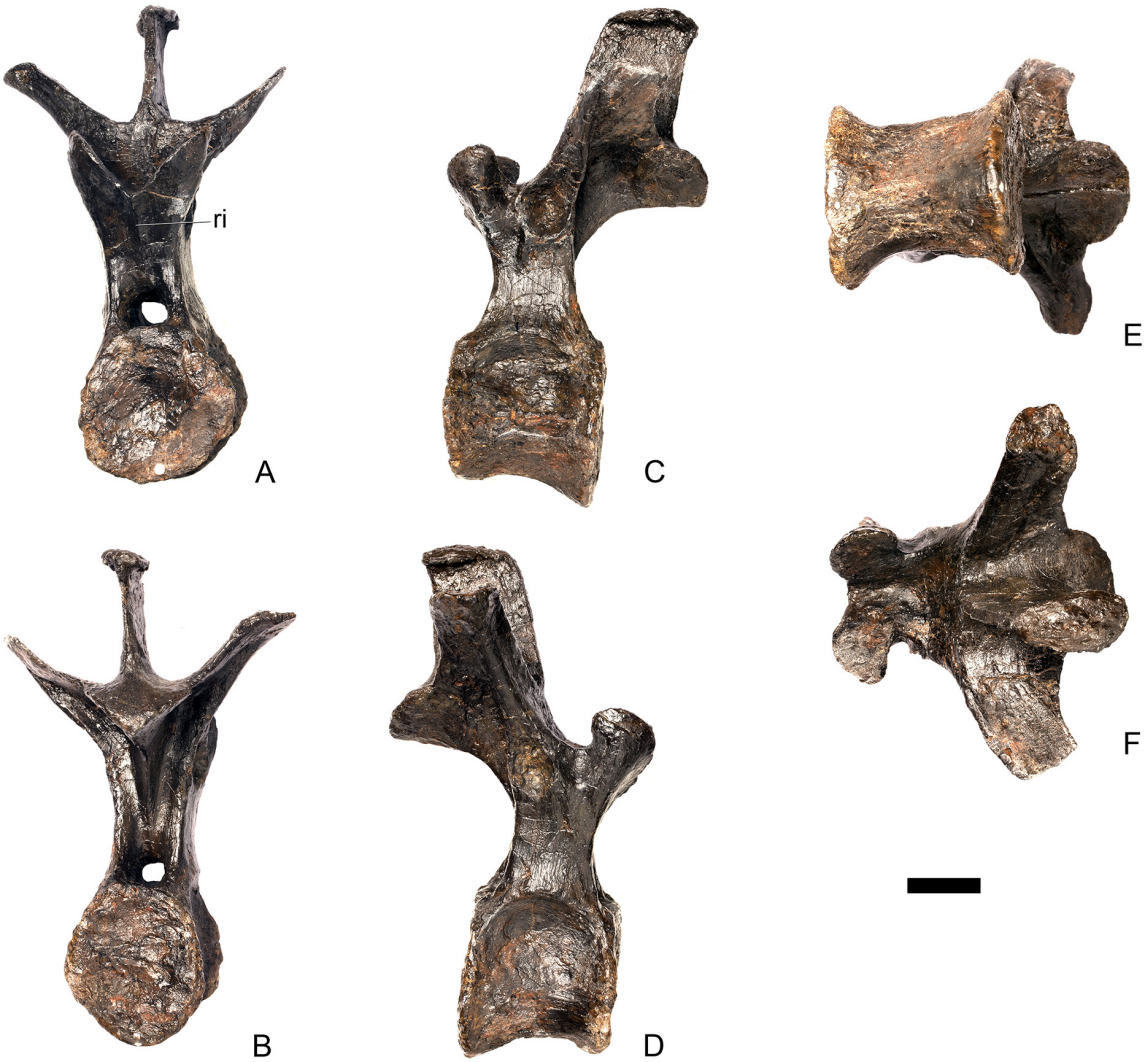
Dorsal vertebra nine. **A**, anterior, **B**, posterior, **C**, left lateral, **D**, right lateral, **E**, ventral and **F**, dorsal view. **Ri,** ridge. Scale bar equal to 5 cm.

The end of the left diapophysis of D9 is broken, while the right diapophysis and parts of the right prezygapophysis are reconstructed. The remaining features are well preserved although the base of the neural arch has been skimmed with plaster. D9 is similar to the preceding vertebrae in most respects. However, in anterodorsal view, the fossa located posterior to the prezygapophyses is absent, as is the midline ridge that extends from the fossa to the base of the neural spine in the preceding vertebrae (Fig 26A and 26F). In anterior view, the fossa surrounding the neural canal is greatly reduced in prominence (Fig 26A). The ridges extending from the anterior margins of the diapophyses to the base of the anterior margin of the neural spine are less well developed in D9 than they were in preceding vertebrae (Fig 26A). Finally, in anterior view, a ridge extends ventrally from the ventral margins of the prezygapophyses where they meet. This ridge appears to be incipient on preceding vertebrae but forms a clear structure in D9 (Fig 26A).

#### Dorsal vertebra 10 (Fig 27)

**Fig 27:**
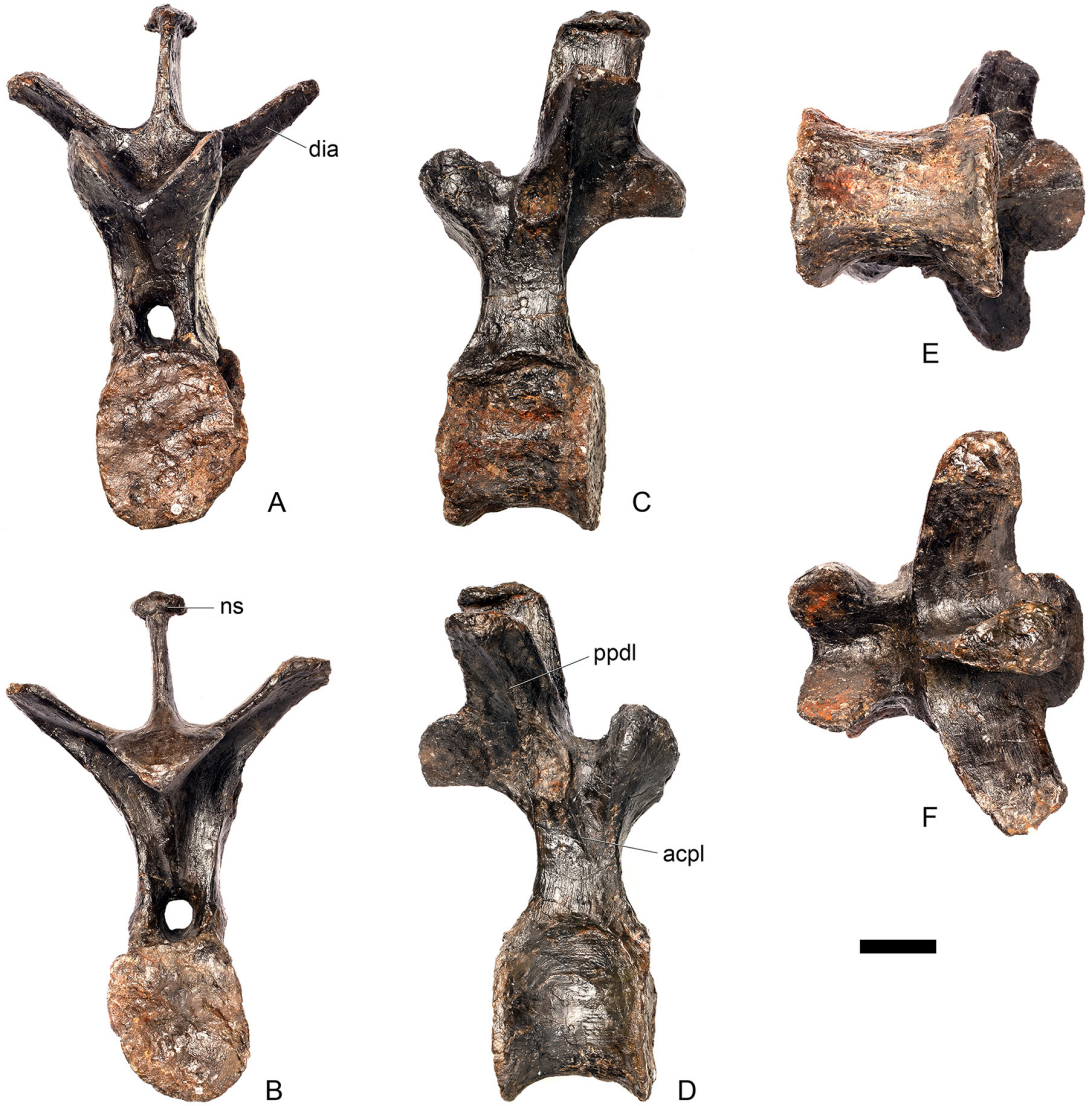
Dorsal vertebra 10. **A**, anterior, **B**, posterior, **C**, left lateral, **D**, right lateral, **E**, ventral and **F**, dorsal view. **Acpl**, anterior centroparapophyseal lamina; **dia**, diapophysis; **ns**, neural spine; **ppdl**, paradiapophyseal lamina. Scale bar equal to 5 cm.

D10 is similar in most respects to D9, differing from it in the following ways. The articular surfaces of the centrum are sub-elliptical in outline, being dorsoventrally taller than they are wide transversely. In anterior view, the fossa surrounding the neural canal is absent (Fig 27A). Diapophyses extend at a lower angle than they do in D8 and D9, forming an angle of 65 degrees from vertical (Fig 27A). As in D8 and D9, the ACPL is present as a short ridge in lateral view, but there is no differentiation between the PCDL and the PPDL, with a single lamina corresponding to the PPDL being present (Fig 27D). The top of the neural spine appears to be expanded transversely to a greater degree than seen in previous vertebrae (Fig 27A, 27B and 27F).

The mid-posterior dorsals of *Dacentrurus armatus* (NHMUK OR46013; [29]: fig 3A, B, G–N) appear to be morphologically similar to those of NHMUK PV R36730, although the neural canal is relatively larger, excavates more deeply into the top of the centrum and is more clearly teardrop-shaped in posterior view. The mid-posterior dorsals of *Kentrosaurus* have more dorsally elongated neural arches and much larger neural canals, which occupy the whole of the elongated neural arch, than those of NHMUK PV R36730 ([33]: figs 16–22).

#### Dorsal vertebra 11 (Fig 28)

**Fig 28:**
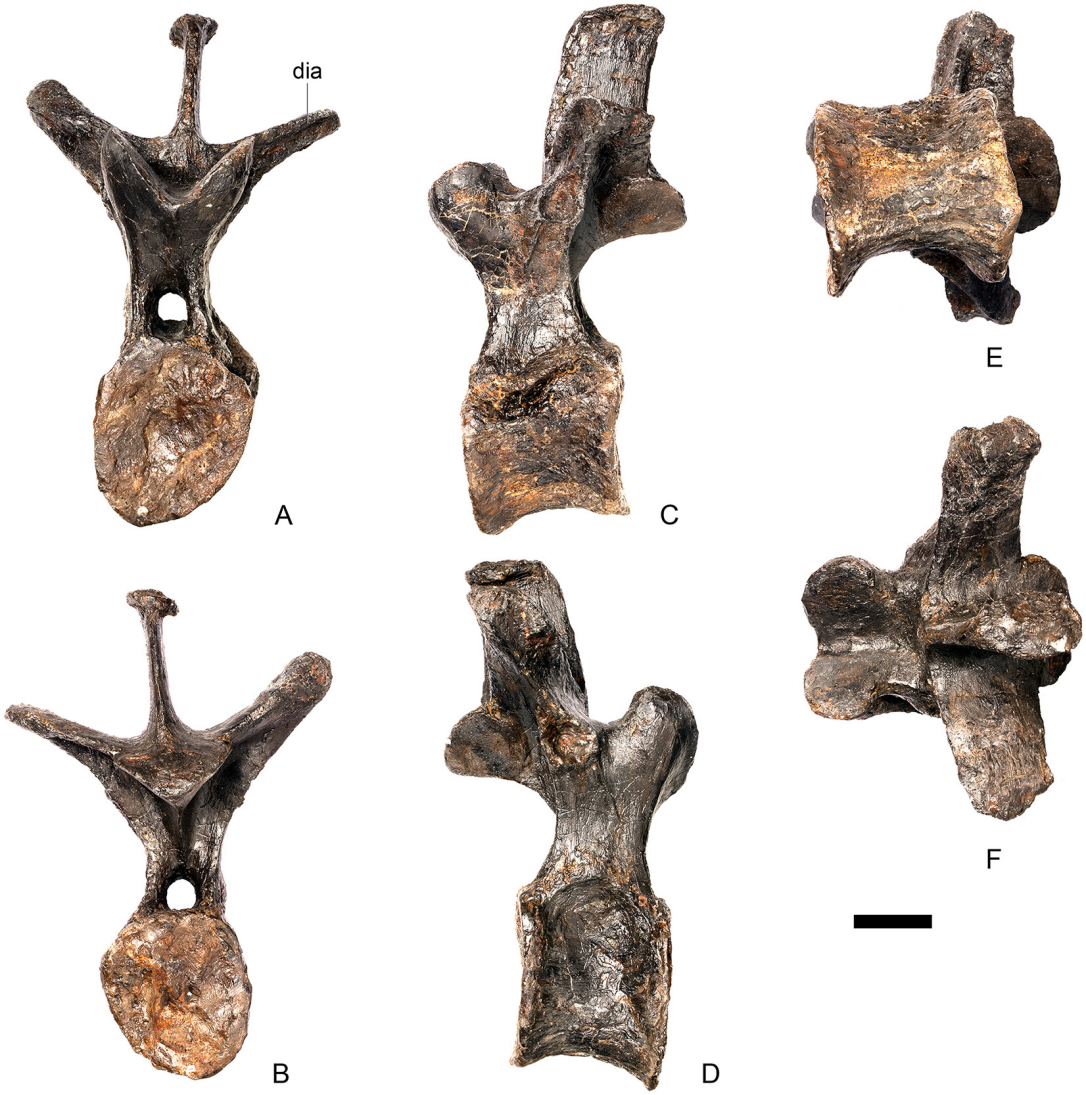
Dorsal vertebra 11. **A**, anterior, **B**, posterior, **C**, left lateral, **D**, right lateral, **E**, ventral and **F**, dorsal view. **Dia**, diapophysis. Scale bar equal to 5 cm.

A large indentation due to crushing is present on the centrum of D11 and the right prezygapophysis is reconstructed. The vertebra is very similar in morphology D10, although the diapophyses appear to project at a lower angle. They are, however, slightly distorted (Fig 28A). The neural arch is less expanded dorsally so the pedicle area is shorter in comparison with preceding vertebrae (Fig 28C and 28D). [2] noted that the posterior dorsals of the holotype of *S*. *stenops* (USNM 4934) has neural arches that are lower than those of the mid-dorsals. In other respects, the vertebra appears identical to that of D10.

#### Dorsal vertebra 12 (Fig 29)

**Fig 29:**
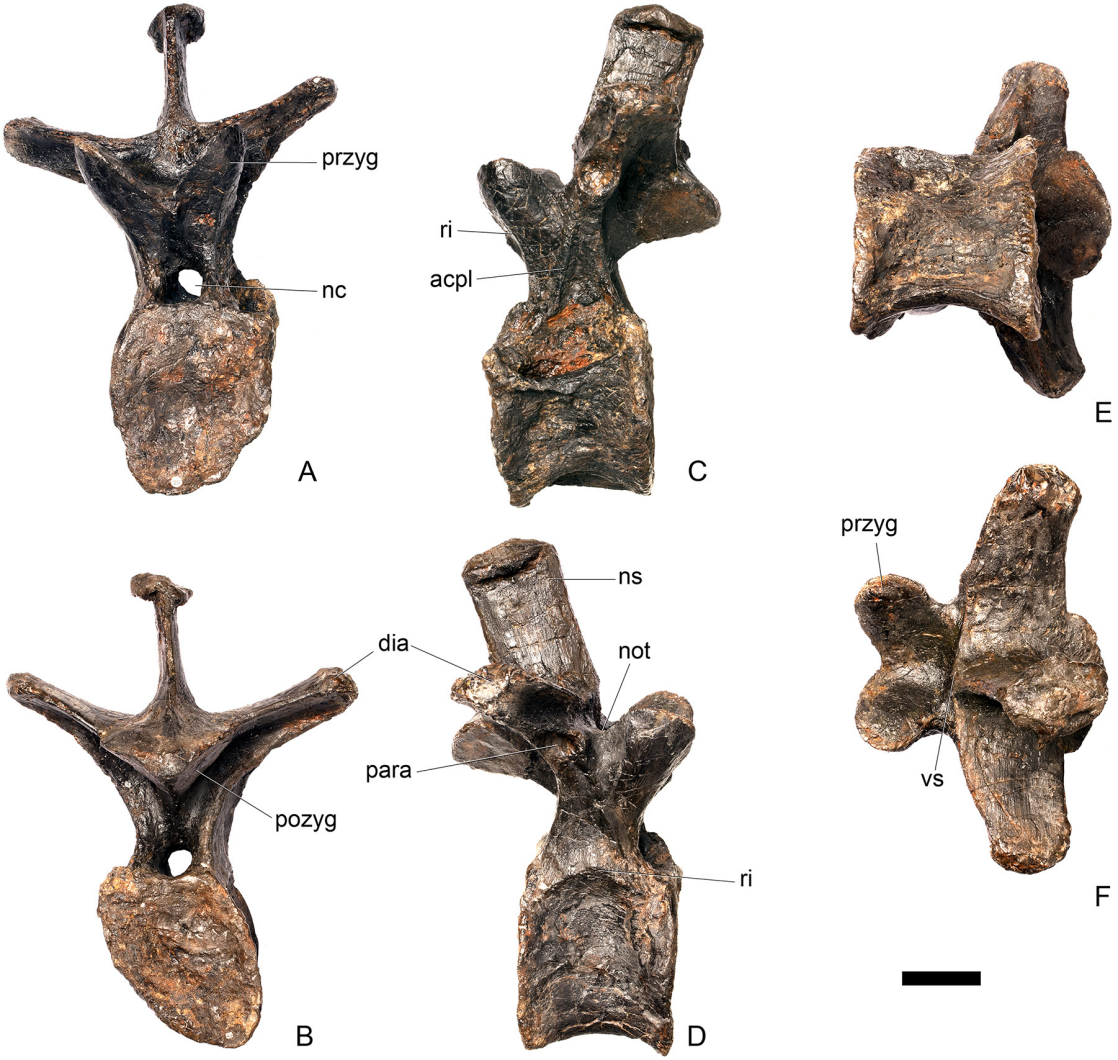
Dorsal vertebra 12. **A**, anterior, **B**, posterior, **C**, left lateral, **D**, right lateral, **E**, ventral and **F**, dorsal view. **Acpl**, anterior centroparapophyseal lamina; **dia**, diapophysis; **nc**, neural canal; **not**, notch; **ns**, neural spine; **para**, parapophysis; **pozyg**, postzygapophysis; **przyg**, prezygapophysis; **ri**, ridge; **vs**, vertical sheet. Scale bar equal to 5 cm.

D12 differs from preceding vertebrae in a number of respects. As with many of the vertebrae, it is well preserved, although surfaces are skimmed with plaster. The centrum has been crushed obliquely so that the right hand side extends ventrally further than the left hand side, and as in dorsal 11 there is a large fossa on the right side of the centrum that is probably due to crushing, although its presence on two vertebrae suggest it could have been a pathology. The anterior articular facet is generally flat with a shallow central concavity; the posterior articular facet is gently concave (Fig 29A and 29B). The better preserved right lateral side of the centrum is dorsoventrally and anteroposteriorly concave (Fig 29D). In ventral view a keel is present although this is likely due to crushing. In lateral view, in the location of the neurocentral suture, there is an upwardly convex parabola-shaped ridge that defines the lateral surface of the centrum from the neural arch, as in preceding vertebrae (Fig 29D). In lateral view, the neural arch is slightly set back from the anterior articular facet. The neural arch is much shorter than in mid-dorsal vertebrae and in anterior and posterior views the bases of the prezygapophyses and postzygapophyses respectively arise from immediately above the neural canal opening (Fig 29A and 29B). The neural canal is rounded in outline in anterior and posterior view. The prezygapophyses are stout, dorsolaterally projecting processes that have strongly convex ventrolateral surfaces. A subtle ridge arises from the lateral surface of the prezygapophysis and extends ventrally to merge with the anterior margin of the neural arch (Fig 29A). The articular surfaces of the prezygapophyses are sub-ovate in outline and gently convex on the surface. They merge into each other ventrally on the midline, describing a deep transversely concave surface (Fig 29F). In anterior view, a midline ridge extends from the base of the prezygapophyses to the dorsal margin of the neural canal (Fig 29A). The vertical sheet arises immediately posterior to the prezygapophyses. A midline ridge extends up it to the base of the neural spine (Fig 29A and 29F). The neural spine is transversely compressed, rectangular in lateral view, and the spine table is more expanded than in preceding vertebrae (Fig 29A, 29D and 29F). The diapophyses extend at a much lower angle than in preceding vertebrae, projecting at around 20 degrees to the horizontal (Fig 29A). In lateral view, the diapophyses are T-shaped in cross-section with the downstroke of the T forming a prominent ridge that extends from the end of the diapophysis to the dorsal margin of the parapophysis (Fig 29D). The ridge extending from the anterior surface of the diapophyses to the base of the anterior neural spine is present (Fig 29F). The parapophysis is ellipsoid in outline, smaller than in the mid-dorsals, and strongly concave. It is situated on a small pedestal at the base of the diapophysis and level with the prezygapophyses (Fig 29C and 29D). The prezygapophyses are separated from the diapophysis and parapophysis by a notch (Fig 29D). A prominent ridge extends from the ventral parapophysis anteroventrally to the location of the neurocentral suture (ACPL; Fig 29C). Posterior to the ACPL the neural arch is gently concave. A second ridge extends from the posterior surface of the parapophysis posteroventrally to form the posterior surface of the neural arch. In posterior view the postzygapophyses form a solid triangular wedge, the posterior margin of which is continuous with the posterior margin of the diapophyses (Fig 29B). The articular surfaces of the postzygapophyses are flat, sub-triangular, face ventrolaterally and are joined on their midline. A prominent ridge extends from the ventral margin of the postzygapophyses to the dorsal margin of the neural canal. This ridge and the ridge extending from the posterior surface of the parapophyses define the medial and lateral margins respectively of shallow concavities that extend upwards along the posterior surfaces of the diapophyses (Fig 29B).

#### Dorsal vertebra 13 (Fig 30)

**Fig 30:**
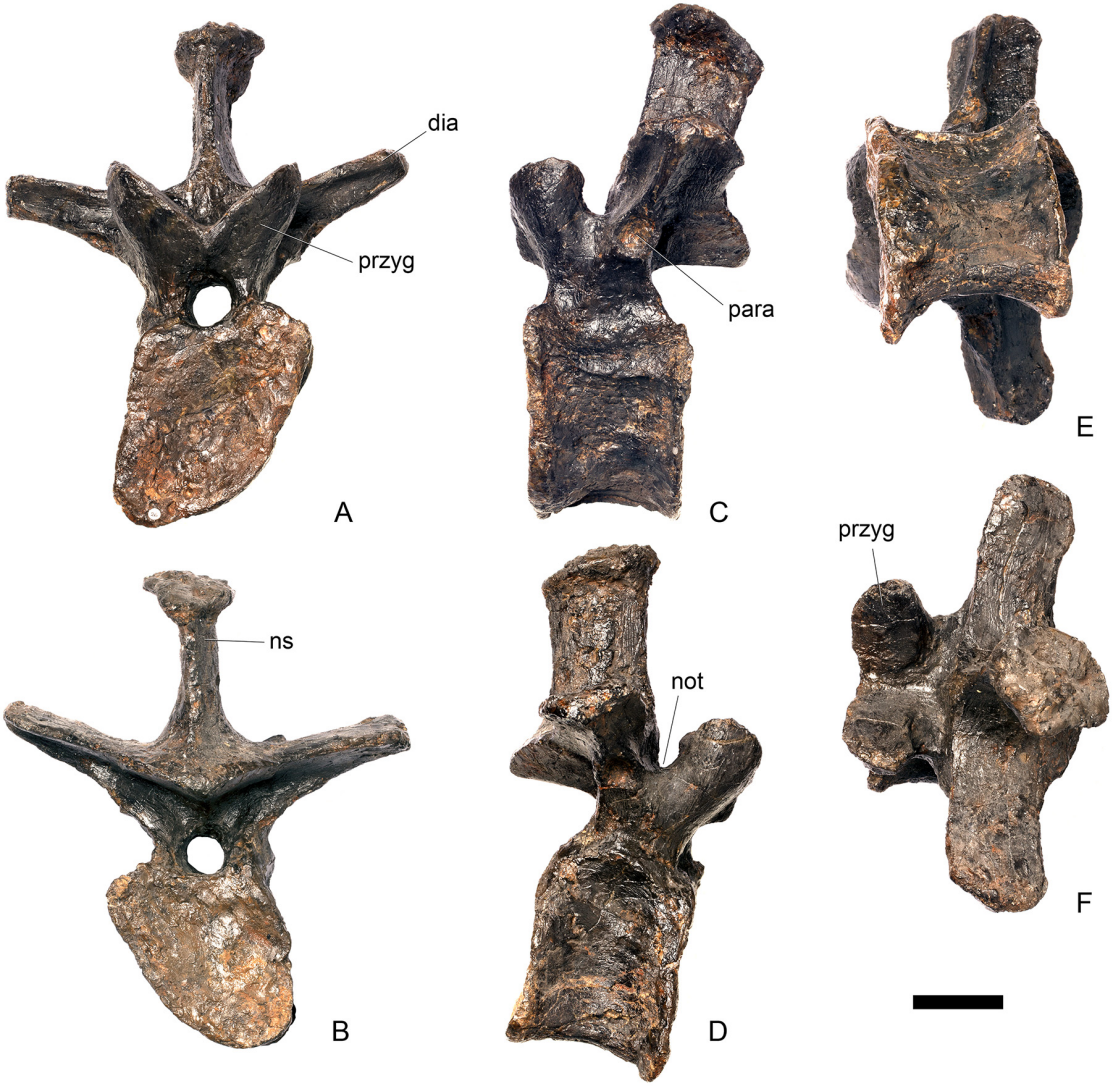
Dorsal vertebra 13. **A**, anterior, **B**, posterior, **C**, left lateral, **D**, right lateral, **E**, ventral and **F**, dorsal view. **Dia**, diapophysis; **not**, notch; **ns**, neural spine; **para**, parapophysis; **przyg**, prezygapophysis. Scale bar equal to 5 cm.

The centrum of D13 is crushed obliquely such that the left side projects further dorsally than the right side. It is similar to D12 except in the following ways. The neural arch is even shorter such that the bases of the diapophyses and prezygapophyses essentially extend directly from the top of the canal with little intervening neural arch (Fig 30A and 30B). The prezygapophyses are relatively and absolutely smaller, and are tongue-shaped rather than ovate (Fig 30F). Consequently, the notch between the diapophyses and the prezygapophyses is larger (Fig 30D). The parapophysis is smaller and located slightly further along the diapophysis, about one-third of the way along in anterior view. Consequently, it is now angled so that it faces ventrolaterally (Fig 30C and 30D). In anterior view the diapophyses extend at a lower angle and are only slightly elevated from the horizontal, forming an angle of about 10 degrees (Fig 30A). The neural spine is not as transversely compressed as in preceding vertebrae and the spine table is again more enlarged transversely than on preceding vertebrae (Fig 30A, 30D and 30F).

#### Dorsal vertebra 14 (dorsosacral vertebra; Fig 31)

**Fig 31:**
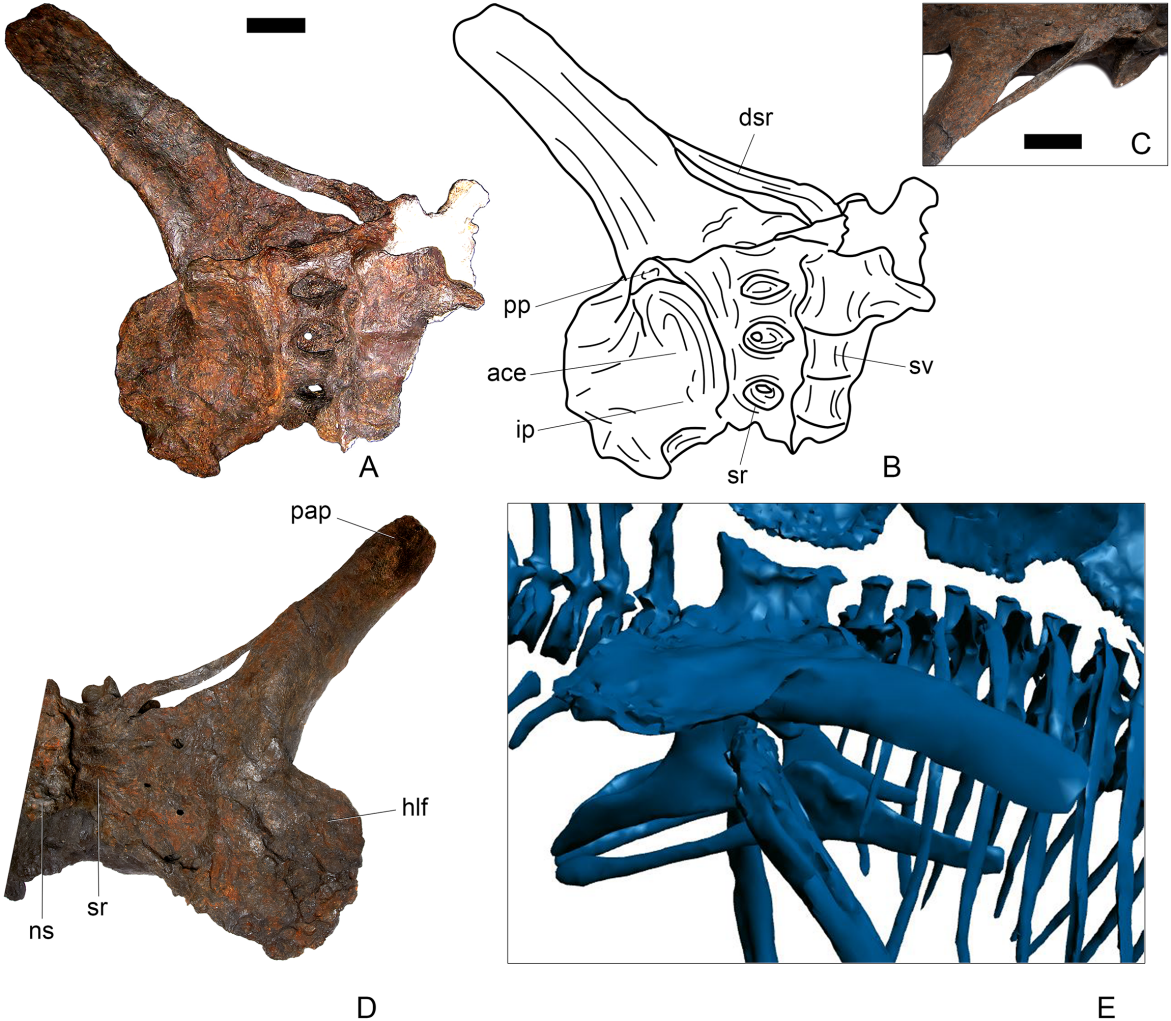
Iliosacral block. Photograph (**A**) and interpretive line drawing (**B**) of right side of iliosacral block in ventral view, photographed before restoration for mounting. **C**, right side of iliosacral block in dorsal view. **C**, detail of dorsosacral rib and its attachment to preacetabular process, anteromedial oblique view. **D**, pelvis in right lateral view, taken from the 3D photogrammetric model (S1 Fig). **Ace**, acetabulum; **dsr**, dorsosacral rib; **hlf**, hypertrophied lateral flange; **ip**, ischiadic peduncle; **ns**, neural spines; **pap**, preacetabular process; **pp**, pubic peduncle; **sr**, sacral ribs; **sv**, sacral vertebrae. Scale bars equal to 10 cm.

A dorsosacral vertebra, corresponding to D14, is poorly preserved but appears to have been fused to the sacral vertebrae. The 27^th^ presacral is also coossifed with the sacrum in the holotype of *S*. *stenops* (USNM 4934; [2]). The centrum and prezygapophyses are entirely reconstructed; all that remains of the vertebra is the neural spine, which is fused to that of the first sacral vertebra, and they can be differentiated by a groove between them (Fig 31D and 31E). The spine extends vertically, is compressed transversely, and expanded dorsally to a degree greater than that seen in preceding dorsals. The spine table is heavily eroded. The dorsosacral vertebra bears a rib that contacts the ilium (Fig 31A–31D). This rib is dorsoventrally compressed, elongate and tapers distally. It extends anterolaterally from the base of the neural spine and contacts the preacetabular process of the ilium approximately one-third of the way along the length of the latter. The distal end of the rib appears to be fused to the preacetabular process of the ilium, although the junction between them can still be distinguished (Fig 31C). The rib is separated from the anterior margin of the sacricostal yoke by a slit-like fenestra. A similar dorsosacral rib, articulating with the preacetabular process of the ilium, is present in USNM 4934 (*S*. *stenops*: [2]), and is also present in an iliosacral block preserved in the quarry sandstone in the visitor centre at Dinosaur National Monument (SCRM, pers. obs. 2014). A dorsosacral rib with the same morphology is present in a stegosaurian iliosacral block referred to *Gigantspinosaurus* ([27]: fig 136). Such ribs are also found in ankylosaurs, such as *Euoplocephalus* ([38]: fig 2). In contrast, the dorsosacral vertebra of *S*. *homheni* (IVPP V4006) and the final dorsosacral of *Dacentrurus* (NHMUK OR46013; MIGM 4953; MIGM 5872; [29]: fig 5A, B; [39]: fig 4), which are also fused to the sacral vertebrae, bear transverse processes that extend laterally and their dorsal margins fuse posteriorly with the first sacral vertebra to contribute to a solid sacral yoke. The dorsosacral vertebrae of *Kentrosaurus* (MB R.3615.1–8, R.4572, R.3617) appear to have had free transverse processes that did not fuse to the sacral ribs posteriorly.

### Sacral vertebrae (Fig 31)

Along with the dorsosacral vertebra (D14), four further sacral (S) vertebrae are fused together (Fig 31A and 31B). The sacrum of USNM 4934 (*S*. *stenops*) comprises a dorsosacral and five further co-ossified vertebrae. [2] interpreted the sixth fused vertebra as a caudosacral, homologous to the first caudal, and noted a large degree of variation in the number of fused vertebrae comprising the sacrum in *Stegosaurus*. In *S*. *mjosi*, there are five fused vertebrae representing two dorsosacrals and three sacrals, with space for an unfused fourth sacral (DMNH 29431, [17]). In *S*. *homheni* (IVPP V4006; [34]: fig 89.1) there are five fused vertebrae, representing a dorsosacral and four sacrals. In *Dacentrurus armatus* (NHMUK OR46013; [29]: fig 5A, B) there are seven fused vertebrae, representing two or three dorsosacrals and four or five sacrals. In *Dacentrurus* sp. (MIGM 4953; [39]: fig 9E) there are six fused vertebrae representing three dorsosacrals and three sacrals and an additional sacral rib with a broken vertebra, while in *Dacentrurus* sp. (MIGM 5872) there are also six fused vertebrae, but these seem to represent two dorsosacrals and four sacrals. In *Kentrosaurus* (MB R.3613; MB R.3618; MB R.3615.1–8; MB R.3617; MB R.4572; MB R.3619; [36]: pl. 2), the number of fused vertebrae forming the sacrum varies from four to seven, and there appear to be both dorsosacrals and caudosacrals added to the sacral rod. In *Huayangosaurus* there are four or five fused vertebrae, with the additional one being a dorsosacral [26]. In *Chungkingosaurus* (CV 206; [34]: fig 92; [35]: fig 2b) there are five fused vertebrae comprising four sacrals and a dorsosacral. In *Tuojiangosaurus* (CV 209/210; [34]; [35]: fig 2a) and *Gigantspinosaurus* [37; 27] there are four fused vertebrae.

The vertebrae are very poorly preserved. Only the ventral and right lateral surfaces of the centra are preserved, and before reconstruction the centra were eroded and hollowed out (Fig 31A and 31B). The posterior part of S4 is reconstructed. In ventral view, the centra appear to have been waisted, with anteroposteriorly concave ventral and lateral surfaces that are not separated by breaks in slope but indistinguishably merge into one another (Fig 31A and 31B). The centra are fused to each other but can be distinguished by ridges between them representing the junctions between the articular surfaces. No ventral keel is present (Fig 31A and 31B). [2] noted that a keel was present on the sacral vertebrae of USNM 4934 (*S*. *stenops*) but not on a specimen he referred to as *S*. *ungulatus* (YPM 1858), a species considered here to be a junior synonym of *S*. *stenops*. A keel is also present in *S*. *mjosi* (DMNH 29431), *S*. *homheni* (IVPP V4006) and *Huayangosaurus* ([31]: fig 29), but not in *Kentrosaurus* (MB R.3613; R.3617; R.3619) or *Gigantspinosaurus* [27]. The presence of a ventral keel on the sacral vertebrae may indicate a specific difference among *Stegosaurus* individuals, but at present sample sizes are too small to test this proposal. Dorsally, the neural spines of the sacral vertebrae are fused to each other and to that of the dorsosacral, but they can be distinguished from each other by vertically extending grooves (Fig 31E). The spines are transversely compressed but appear to be thicker transversely than those of the dorsal vertebrae. The dorsal tips of the spines are transversely expanded (Fig 31D), as in USNM 4934 [2] and DMNH 29431, and indistinguishably fused to each other to form an elongate, sub-elliptical spine table that is rugose and heavily eroded. [2] placed considerable emphasis on the height of the neural spines of the sacrum, considering that those of *S*. *ungulatus*, as illustrated by Marsh, were much greater in height. However, Marsh’s illustrations of the iliosacral block of *S*. *ungulatus* are erroneous: the preacetabular processes are shown to project in the sagittal plane (e.g. [2]: fig 22; [40]: pl. 21) in a manner unlike that of any known stegosaur. [2]: p.66, p.67] noted that other aspects of Marsh’s reconstruction of *S*. *ungulatus* were also in error (e.g., scapula, coracoid). Thus it seems possible that Marsh’s reconstruction of the height of the neural spines could also be in error. At the very least it seems unwise to consider the height of the neural spines of the sacral vertebrae to be a diagnostic character at specific level until measurements can be provided to confirm Marsh’s reconstructions.

Ribs are preserved only on the right-hand side. In ventral view, there are four sacral ribs that are anteroposteriorly expanded medially close to the centra and distally, where they are fused to each other and the medial surface of the acetabulum (Fig 31A and 31B), as in other stegosaurs including *S*. *mjosi* (DMNH 29431), *Dacentrurus armatus* (NHMUK OR46013), *Kentrosaurus* (MB R.3613; [36]: pl. 2.3), *Tuojiangosaurus* (CV 209/210; [34]; [35]: fig 2a), *Gigantspinosaurus* ([27]: fig 134), *Chungkingosaurus* (CV 206; [34]; [35]: fig 2b) and *Huayangosaurus* (ZDM T7001; [31]: fig 29; [26]: fig 5a). The ribs are separated from each other by three sub-elliptical fenestrae, the largest of which is located between the second and third sacral ribs, and the smallest of which is between the third and fourth sacral ribs, as in *Kentrosaurus* (MB R.3613; [36]: pl. 2.3). The anterior margin of the first rib is straight, while its posterior margin is anteriorly convex. Medially, the base of the rib appears to contact both the dorsosacral vertebra and S1, although more of the rib was borne by S1 (Fig 31A and 31B). The rib extends laterally to contact the medial surface of the acetabulum. In anterior view, the sacral rib is separated from the diapophysis dorsal to it by a fenestra, so that this vertebra appears to be transitional in morphology between a dorsal and a true sacral. This suggests that S1 is a modified dorsal, and that S2 and S3 are probably homologous to those in the primitive ornithischian sacrum, a conclusion also reached by [2].

The base of the second sacral rib primarily contacts the centrum of S1 but a small part also contacts the anterior part of the S2 centrum. Its anterior margin is anteriorly concave while its posterior margin is anteriorly convex (Fig 31A and 31B). The rib is dorsoventrally tall and anteroposteriorly compressed, extending to the dorsal surface of the sacral yoke: a separate diapophysis is not present. The rib extends laterally and is fused to the medial surface of the acetabulum. The third sacral rib is borne equally by the centra of S2 and S3. It is short and stout, being anteroposteriorly broader and transversely shorter than the preceding ribs. Its anterior margin is anteriorly concave while its posterior margin is anteriorly convex (Fig 31A and 31B). As in the preceding rib, there is no separate diapophysis and the rib extends laterally to fuse with the medial surface of the acetabulum. The fourth sacral rib is the shortest transversely. Medially it is borne equally by S3 and S4. Its anterior margin is anteriorly concave while its posterior margin is anteriorly convex. The rib extends anterolaterally to the posterior part of the medial surface of the acetabulum; it is dorsoventrally tall with no separate diapophysis (Fig 31A and 31B).

In dorsal view, the diapophysis of S1 and the dorsal surfaces of the second, third and fourth sacral ribs are co-ossified to form a flat sacral yoke (Fig 31D). The yoke is perforated by two small fenestrae between the diapophysis of S1 and the second sacral rib, two small fenestrae between the second and third sacral ribs, and two slightly larger fenestrae between the third and fourth sacral ribs. A solid sacral yoke unperforated by foramina is present in *S*. *mjosi* (DMNH 29431), *Dacentrurus* (NHMUK OR46013; MIGM 5872; [39]: fig 4B) and *Kentrosaurus* (MB R.4800) but the shield is perforated by large fenestrae between sacral ribs in *Tuojiangosaurus* (CV 209/210; [34]; [35]: fig 2a), *Chungkingosaurus* (CV 206; [34]; [35]; fig 2b), *Gigantspinosaurus* ([27]: fig 134) and *Huayangosaurus* (ZDM T7001; [31]: fig 29; [26]: fig 5a). The fenestrae between the sacral ribs of NHMUK PV R36730 are much smaller than those between the ribs of stegosaurs in which the sacral yoke is perforate, and their presence may be related to ontogeny (the specimen was not fully grown at time of death; see Ontogenetic Stage, below) or perhaps due to poor preservation. The sacral yoke is fused laterally to the right ilium and is continuous with the dorsal surface of the latter. The suture between the ribs and the ilium is not clear, although a subtle ridge in this region may mark the line of fusion.

### Caudal vertebrae

Caudal (Cd) vertebrae 1–3, 20–37, and 39–45 are preserved. Caudals 4–19 were present but too poorly preserved and weathered to recover [24]. A size mismatch between Cd37 and Cd39 suggests one may be missing in the sequence. [2] considered that there were at least 45 vertebrae in the tail, and perhaps as many as 49, based on the large collection at the USNM.

Progressing posteriorly from caudal 20 to the end of the tail, the vertebral centra become proportionately smaller. Neural spines decrease in height until caudal 35, where they disappear entirely.

#### Caudal vertebra 1 (Fig 32)

**Fig 32:**
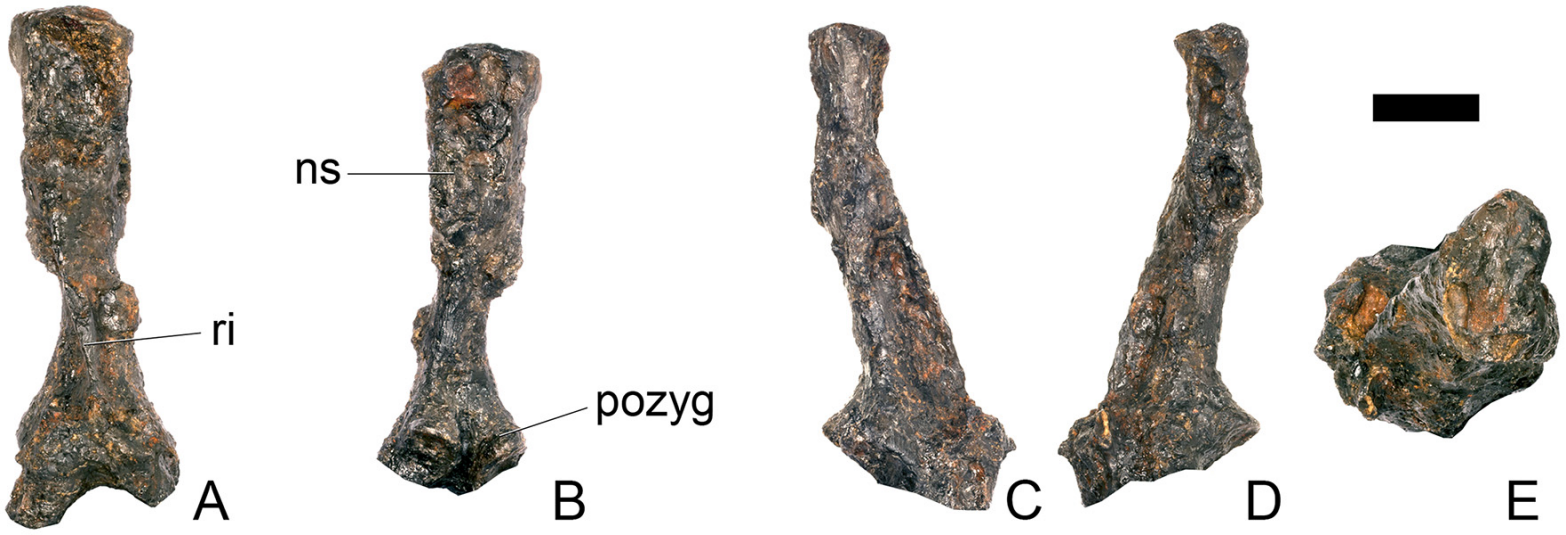
Caudal vertebra one. **A**, anterior, **B**, posterior, **C**, right lateral, **D**, left lateral, and **E**, dorsal view. N**s**, neural spine; **pozyg**, postzygapophysis; **ri**, ridge. Scale bar equal to 5 cm.

Only the neural spine and postzygapophyses are preserved. In anterior view, the neural spine narrows transversely as it extends dorsally until a point approximately halfway along its length, when it expands again towards its summit (Fig 32A). The lateral margins of the spine are therefore concave in anterior view, as in the anterior caudals of *Loricatosaurus* (MHNH(BR) 001; [32]: pl. 2), but in contrast to the condition in *Gigantspinosaurus* ([27]: fig 132 E, F) and *Huayangosaurus* ([31]: figs 20 and 21) in which the tops of the anterior caudals are not expanded. The anterior margin of the spine bears a sharp midline ridge that merges into the main body of the spine about halfway along its length (Fig 32A), as in *Dacentrurus armatus* (NHMUK OR46013: [29]: fig 8A, B). It is not possible to tell if the spine is complete dorsally due to poor preservation, but there is no indication that the tip was bifid. [2] noted that the neural spines bifurcated dorsally in the mid-caudal vertebrae of *Stegosaurus*. In lateral view, the spine extends dorsally and slightly posteriorly (Fig 32C and 32D). The spine has a sub-elliptical cross-section dorsally, becoming tear-drop shaped due to the presence of the anterior ridge ventrally. In posterior view, the postzygapophyses have articular facets that are orientated at 10–15 degrees to horizontal, facing mainly ventrally and only slightly laterally. They are separated on the midline by a shallow cleft. The articular facets are shallowly concave (Fig 32B).

#### Caudal vertebra 2 (Fig 33)

**Fig 33:**
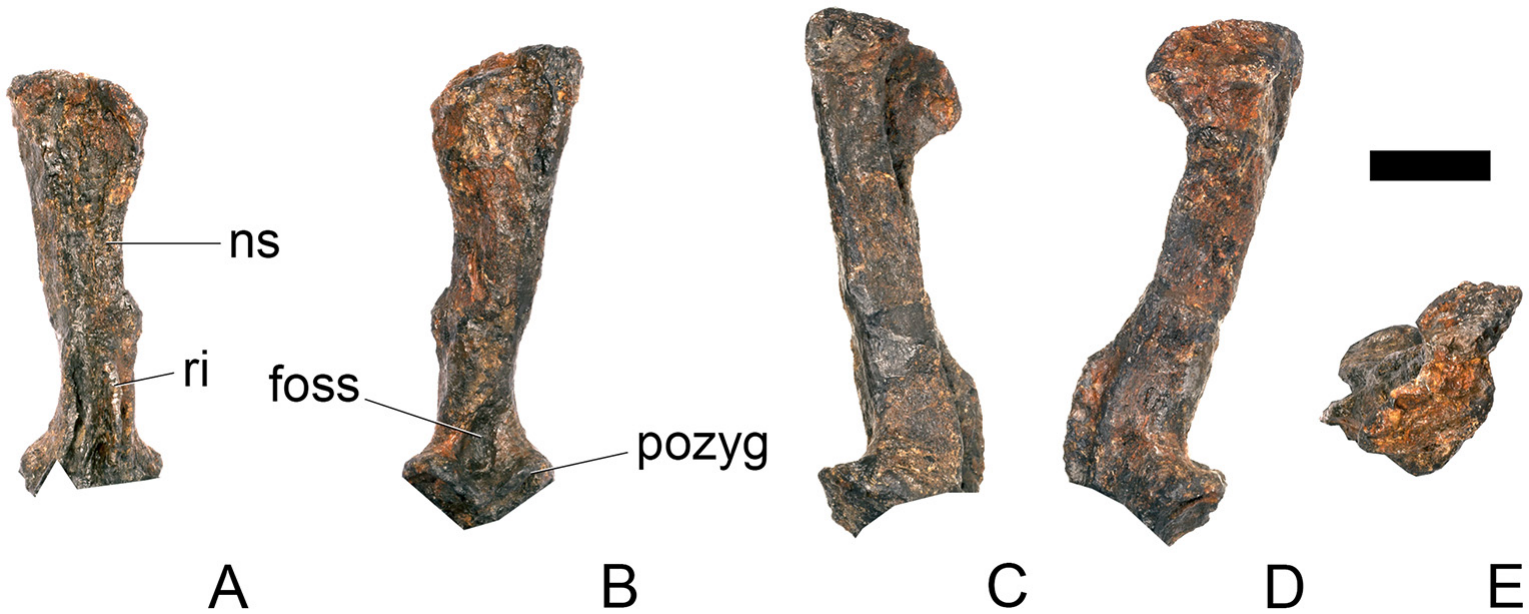
Caudal vertebra two. **A**, anterior, **B**, posterior, **C**, right lateral, **D**, left lateral, and **E**, dorsal view. **Foss**, fossa; **ns**, neural spine; **pozyg**, postzygapophysis; **ri**, ridge. Scale bar equal to 5 cm.

As in Cd1, only the neural spine and postzygapophyses are preserved, although the degree of preservation is better. The tip of the neural spine is sheared so that the left side extends further anteriorly than the right. In anterior view, a prominent ridge is present on the ventral third of the anterior surface of the spine, and deep furrows are present adjacent to it (Fig 33A). The remainder of the anterior surface of the spine is flat except where the aforementioned deformation creates a strong concavity at the tip. The spine has a sub-quadrate cross-section immediately dorsal to the termination of the anterior ridge. As in Cd1 the lateral margins of the spine are concave in anterior view with the tip being transversely broadened (Fig 33A). The tip of the neural spine is also transversely broadened in *Dacentrurus armatus* (NHMUK OR46013; [29]: fig 8A, B) and *Loricatosaurus* (MHNH(BR) 001). In dorsal view, the tip of the spine has a dumbbell-shaped outline perhaps suggesting that it may have been bifid, although poor preservation makes this observation inconclusive (Fig 33E). In lateral view the spine projects dorsally and slightly posteriorly (Fig 33C and 33D), as in *Loricatosaurus* (MHNH(BR) 001; [32]: fig 1). Posteriorly, the articular surfaces of the postzygapophyses remain at a low angle to the horizontal and they are fused ventrally on the midline, in contrast to the condition in caudal one (Fig 33B). The posterior surface of the spine between the postzygapophyses is marked by a shallow elliptical fossa.

#### Caudal vertebra 3 (Fig 34)

**Fig 34:**
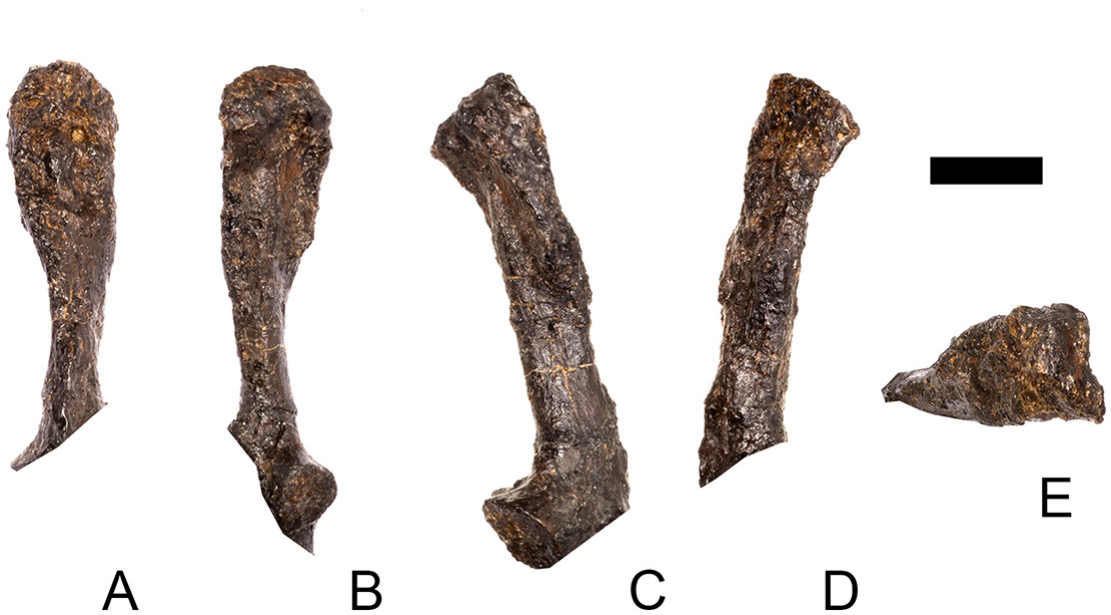
Caudal vertebra three. **A**, anterior, **B**, posterior, **C**, right lateral, **D**, left lateral, and **E**, dorsal view. Scale bar equal to 5 cm.

Only the neural spine and right postzygapophysis remain. In anterior view, the midline ridge present in Cd1–2 is reduced to a low swelling. The lateral surfaces remain concave in anterior view and the tip of the spine is expanded transversely (Fig 34A). In dorsal view, the tip of the spine is anteroposteriorly crushed but appears to have had a dumbbell-shaped outline similar to that of Cd2 (Fig 34E). Ventrally, the spine is teardrop-shaped in cross-section. Although the postzygapophyses are incomplete it appears that they were not confluent on the midline but were separated by a shallow cleft, as in caudal one. There is also no evidence for the shallow fossa dorsal to the postzygapophyses on the neural spine (Fig 34B). The articular surface of the right postzygapophysis is angled at around 20 degrees to the horizontal, facing posteroventrolaterally. The articular facet has a sub-oval outline.

#### Caudal vertebra 20 (Fig 35)

**Fig 35:**
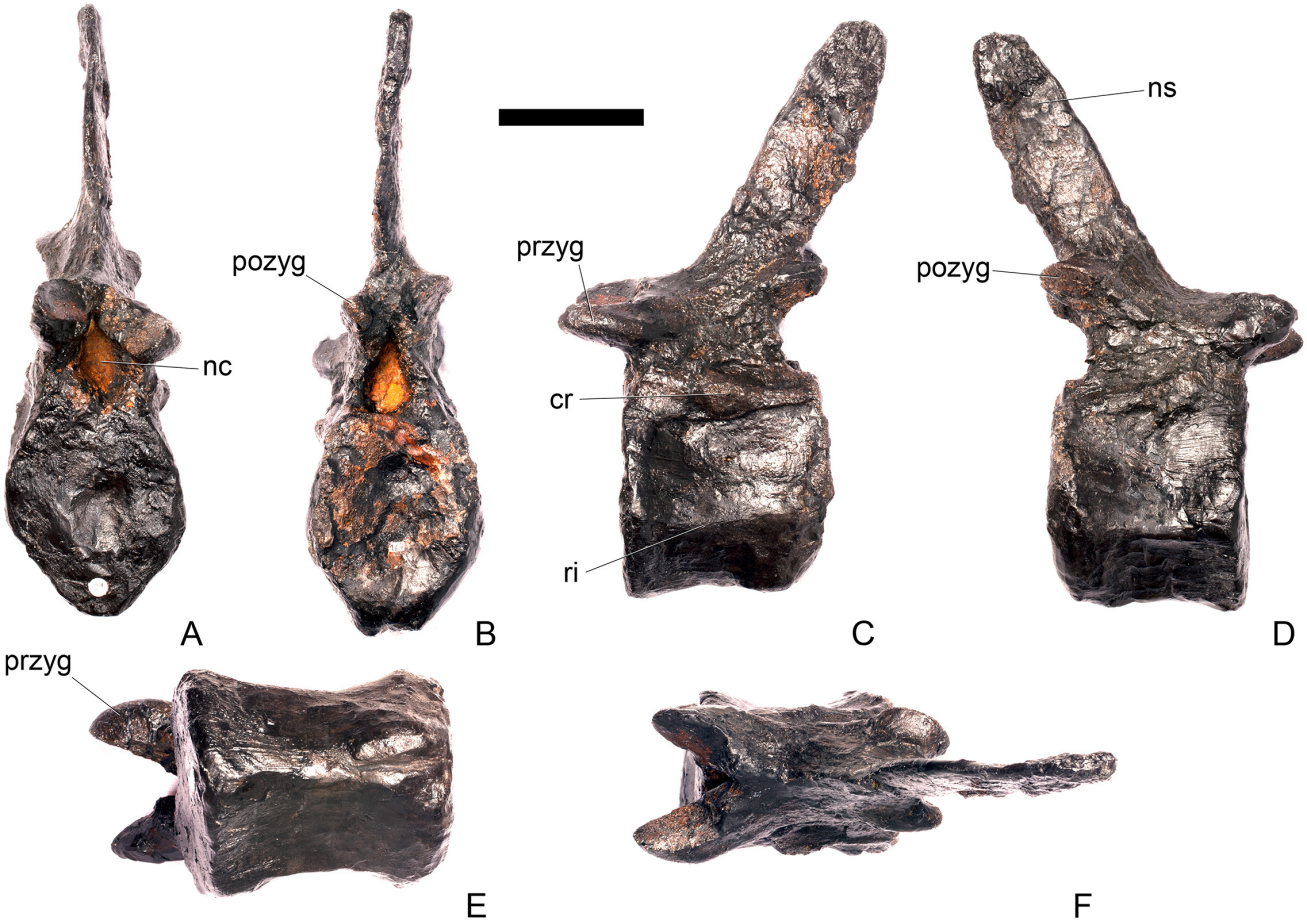
Caudal vertebra 20. **A**, anterior, **B**, posterior, **C**, left lateral, **D**, right lateral, **E**, ventral and **F**, dorsal view. **Cr**, caudal rib; **nc**, neural canal; **ns**, neural spine; **pozyg**, postzygapophysis; **przyg**, prezygapophysis; **ri**, ridge. Scale bar equal to 5 cm.

Cd20 is complete, but the right lateral and ventral sides of the centrum and the anterior articular facet are coated in plaster. The right prezygapophysis is reconstructed. The centrum is longer anteroposteriorly than it is wide transversely, and taller dorsoventrally than it is long anteroposteriorly. The anterior and posterior articular surfaces are pentagonal in outline with the apex pointing ventrally (Fig 35A and 35B). The anterior articular surface is flat with a slight central concavity, while the posterior articular surface is concave. In lateral view, the centrum bears a strong anteroposteriorly extending ridge that connects the anterior and posterior articular surfaces. This ridge divides the lateral surface of the centrum into two, one part of which faces dorsolaterally while the other faces ventrolaterally (Fig 35C and 35D). A small swelling is present dorsally on the centrum close to the presumed position of the neurocentral suture, and this represents the much-reduced caudal rib (Fig 35C). It is positioned equidistant from the anterior and posterior margins. [2] suggested that caudal ribs disappeared around Cd17 or 18 in USNM 4934, and they disappear after Cd19 in *S*. *mjosi* (DMNH 29431). In *Kentrosaurus* (MB R.4800; [33]: pl. 14), however, small swellings are present to at least Cd29. Ventrally, the chevron facets are obscured by plaster, and although a keel is reconstructed it is not clear if this represents a genuine underlying feature.

In anterior and posterior views, the neural canal is teardrop-shaped, and the opening is larger posteriorly (Fig 35A and 35B). The left prezygapophysis is finger-like, projects anteriorly and extends anterior to the articular surface of the centrum (Fig 35C), similar to that of *Gigantspinosaurus* (ZDM 0019) and *Huayangosaurus* ([31]: fig 22), but in contrast to the condition in the mid-caudals of *Loricatosaurus* (NHMUK PV R3167; [29]: fig 17P), in which the prezygapophyses are much smaller and do not extend over the anterior articular facet. In *Kentrosaurus* (MB R.4800; [33]: pl. 14) the prezygapophyses extend over the anterior articular facet but differ from those of NHMUK PV R36730 in that they also extend slightly dorsally. The articular surface of the prezygapophysis in NHMUK PV R36730 angles at about 40 degrees to vertical and appears to have been separated on the midline from its counterpart by a deep cleft (Fig 35A). The lateral margin of the prezygapophysis is strongly convex in lateral view, so that its transverse cross-section is sub-triangular with a flat medial surface and the apex pointing laterally (Fig 35C). The postzygapophyses are situated dorsal to the prezygapophyses and do not extend beyond the posterior margin of the centrum (Fig 35C and 35D), in contrast to the condition in *Loricatosaurus* (NHMUK PV R3167; [29]: fig 17P) where they overhang the posterior articular facet. In *Kentrosaurus* (MB R.4800; [33]: pl. 14) the postzygapophyses are much larger, more distinct processes, although they do not overhang the posterior articular facet because the whole of the neural arch is anteriorly inclined. The articular facets of the postzygapophyses of NHMUK R36730 are oval in outline and they angle at 45 degrees to vertical (Fig 35B and 35D). They are separated from each other by a cleft. In lateral view, the neural spine is rectangular. It arises from immediately posterior to prezygapophyses and extends posterodorsally as a transversely compressed plate (Fig 35C and 35D). Its transverse width is constant along the length of the spine and it is not transversely expanded dorsally (Fig 35A and 35B). [2] considered that transverse expansion and bifurcation of the tops of the neural spines continued as far down the tail as Cd25 or 26 in *Stegosaurus*, but transverse expansion is only present until Cd17 or 18 in *S*. *mjosi* (DMNH 29431). The neural spines of the mid-caudals in *Kentrosaurus* (MB R.4800; [33]: pl. 14) curve anteriorly, being hook-shaped in lateral view, and are not expanded dorsally.

#### Caudal vertebra 21 (Fig 36)

**Fig 36:**
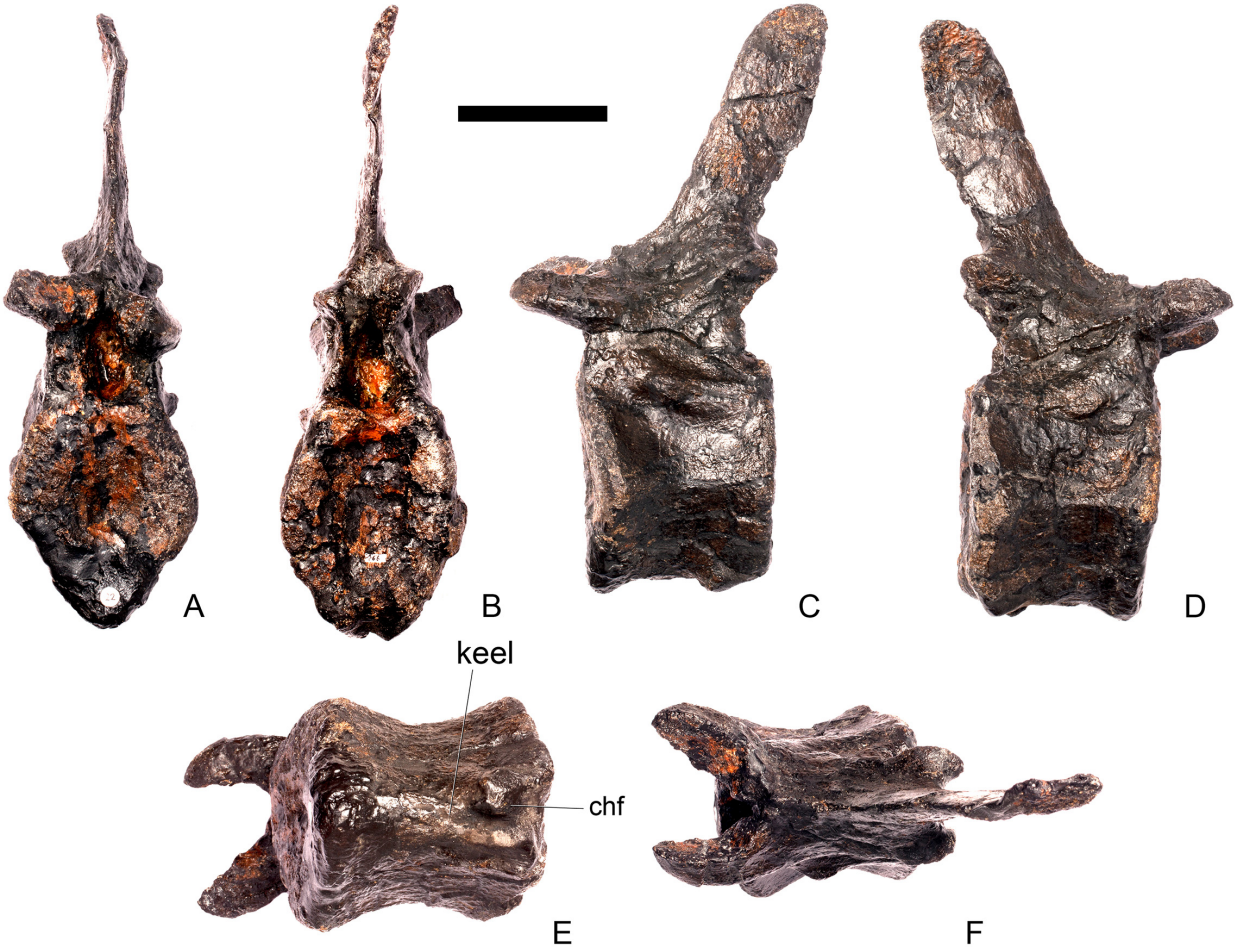
Caudal vertebra 21. **A**, anterior, **B**, posterior, **C**, left lateral, **D**, right lateral, **E**, ventral and **F**, dorsal view. **Chf**, chevron facet; **keel**, ventral keel. Scale bar equal to 5 cm.

Cd21 is essentially identical to Cd20, but is much better preserved and slightly smaller. It appears to be complete except for the right anteroventral corner of the centrum, which is reconstructed and thus lacks the anterior chevron facet. The better preservation of Cd21 allows morphological details of the ventral centrum to be determined. In ventral view, a posterior chevron facet is present (Fig 36E). It has a sub-triangular outline that is bisected ventrally by a shallow midline cleft. The mid-caudal vertebrae of *Loricatosaurus* (NHMUK PV R3167; [29]: fig 17P, R, S) also have prominent anterior chevron facets, which are not observed on NHMUK PV R36730. The ventrolaterally facing portions of the lateral surfaces of the centrum converge ventrally at an acute angle, forming a broad ventral keel (Fig 36E). The ventral margin of the centrum is concave in lateral view.

#### Caudal vertebra 22 (Fig 37)

**Fig 37:**
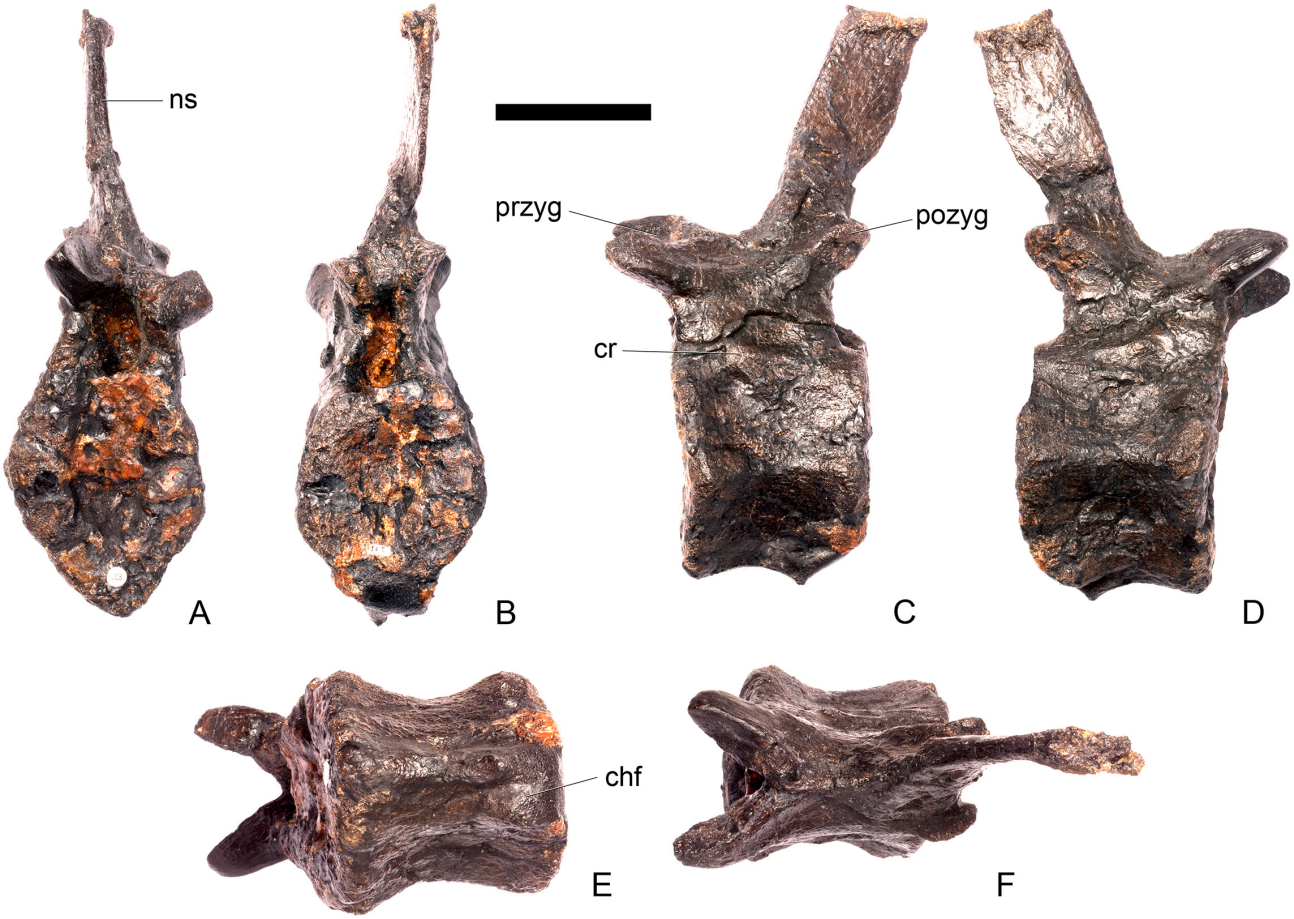
Caudal vertebra 22. **A**, anterior, **B**, posterior, **C**, left lateral, **D**, right lateral, **E**, ventral and **F**, dorsal view. **Chf**, chevron facet; **cr**, caudal rib; **ns**, neural spine; **pozyg**, postzygapophysis; **prezyg**, prezygapophysis. Scale bar equal to 5 cm.

Cd22 is similar in most respects to Cd20 and 21. The right prezygapophysis is reconstructed but the vertebra is otherwise complete. The centrum is well preserved and very similar to that of Cd21 except that better preservation of the anterior margin shows that there is no anterior chevron facet (Fig 37E). The bifid margin of the posterior chevron facet is confluent with a groove that extends anteriorly about halfway along the centrum (Fig 37E). Laterally, the caudal rib is further reduced to a very low swelling (Fig 37C). The anterior articular facet is slightly transversely narrower than the posterior articular facet. Prezygapophyses and postzygapophyses are situated at approximately the same level on the neural arch (Fig 37C and 37D). The neural spine is similar to those of Cd20 and 21, but the tip is slightly expanded transversely (Fig 37A and 37B).

#### Caudal vertebra 23 (Fig 38)

**Fig 38:**
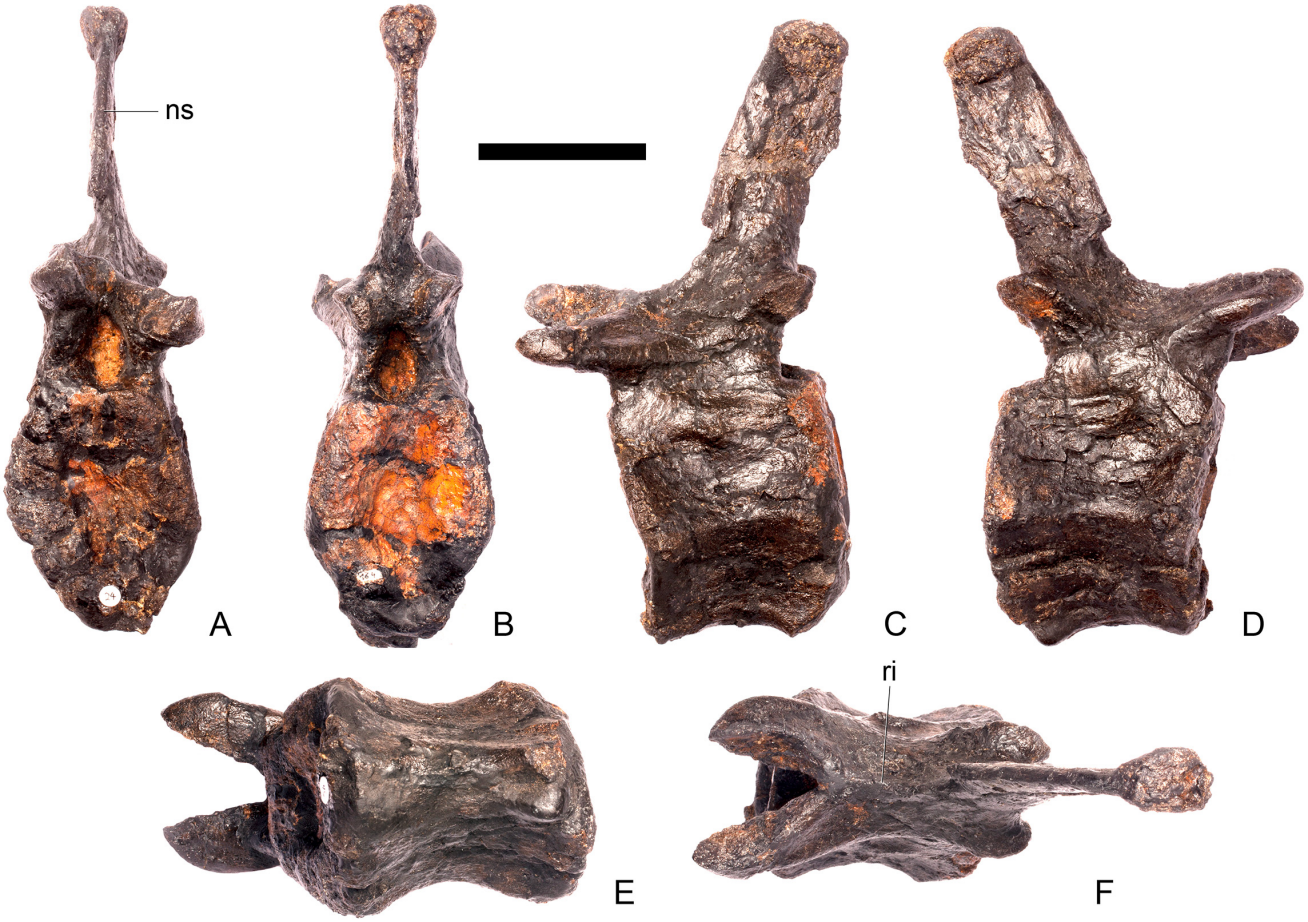
Caudal vertebra 23. **A**, anterior, **B**, posterior, **C**, left lateral, **D**, right lateral, **E**, ventral and **F**, dorsal view. **Ns**, neural spine; **ri**, ridge. Scale bar equal to 5 cm.

Cd23 is complete and essentially identical to Cd22 except that the articular surfaces of the centrum are slightly more deeply excavated (Fig 38A and 38B). The dorsal end of the neural spine is transversely expanded more than in preceding vertebrae into a small spine table (Fig 38A and 38B). Cd23 appears to be slightly transversely narrower than the preceding vertebrae. A low midline ridge that extends posteriorly from the confluence of the prezygapophyses is continuous with the anterior margin of the neural spine (Fig 38F).

#### Caudal vertebra 24 (Fig 39)

**Fig 39:**
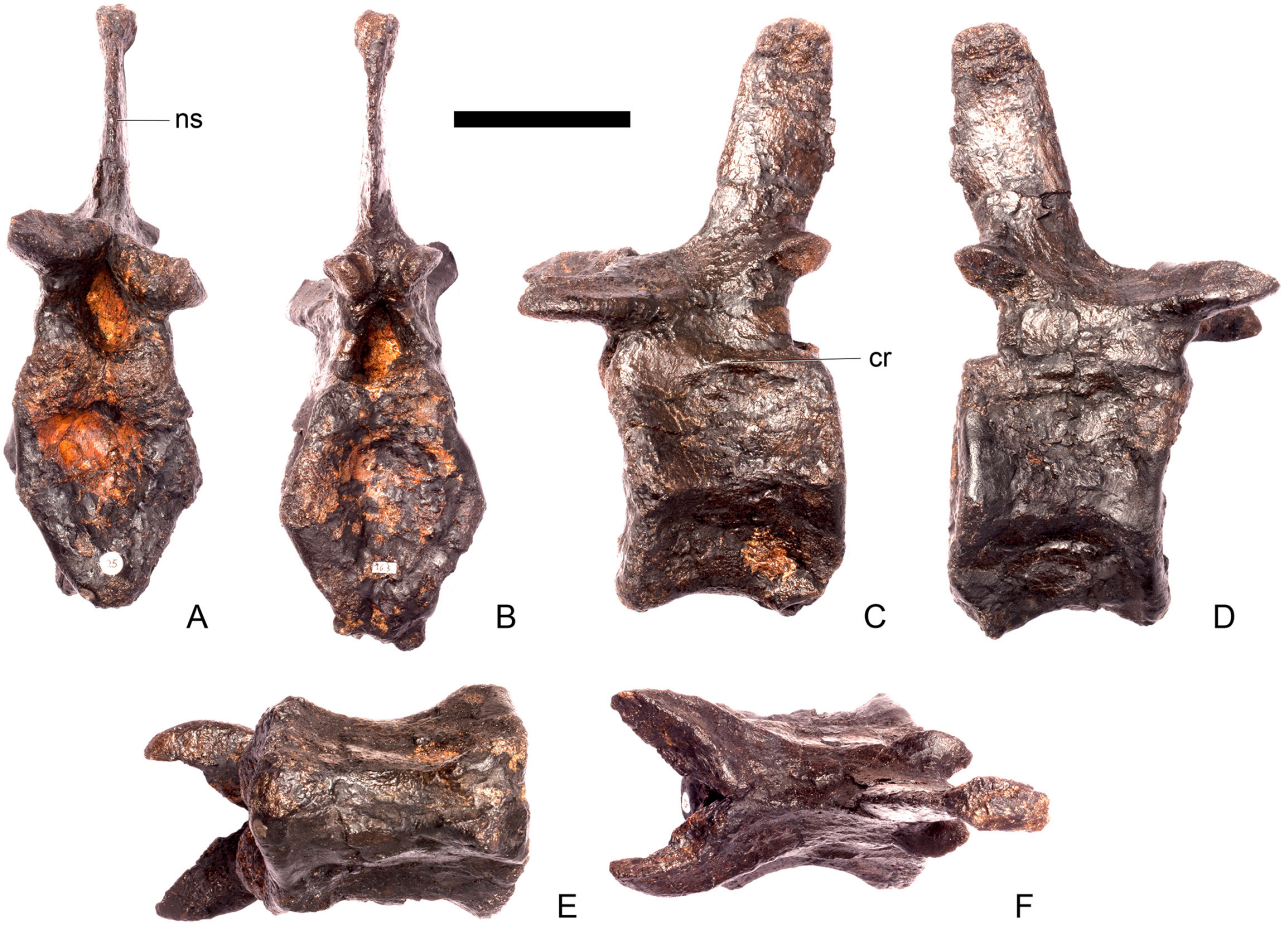
Caudal vertebra 24. **A**, anterior, **B**, posterior, **C**, left lateral, **D**, right lateral, **E**, ventral and **F**, dorsal view. **Cr**, caudal rib; **ns**, neural spine. Scale bar equal to 5 cm.

Cd24 is complete and very similar to Cd23. It is further compressed transversely relative to preceding vertebrae. Laterally, the caudal rib swelling is very small and indistinct (Fig 39C). The neural spine tip is transversely expanded although not to the same degree as observed in Cd23. The neural spine is smaller relatively and absolutely.

#### Caudal vertebra 25 (Fig 40)

Cd25 is complete and essentially identical to Cd24. In lateral view, the anteroposteriorly extending ridge on the side of the centrum has migrated slightly dorsally so that the top one third of the centrum faces dorsolaterally while the lower two thirds face ventrolaterally.

**Fig 40:**
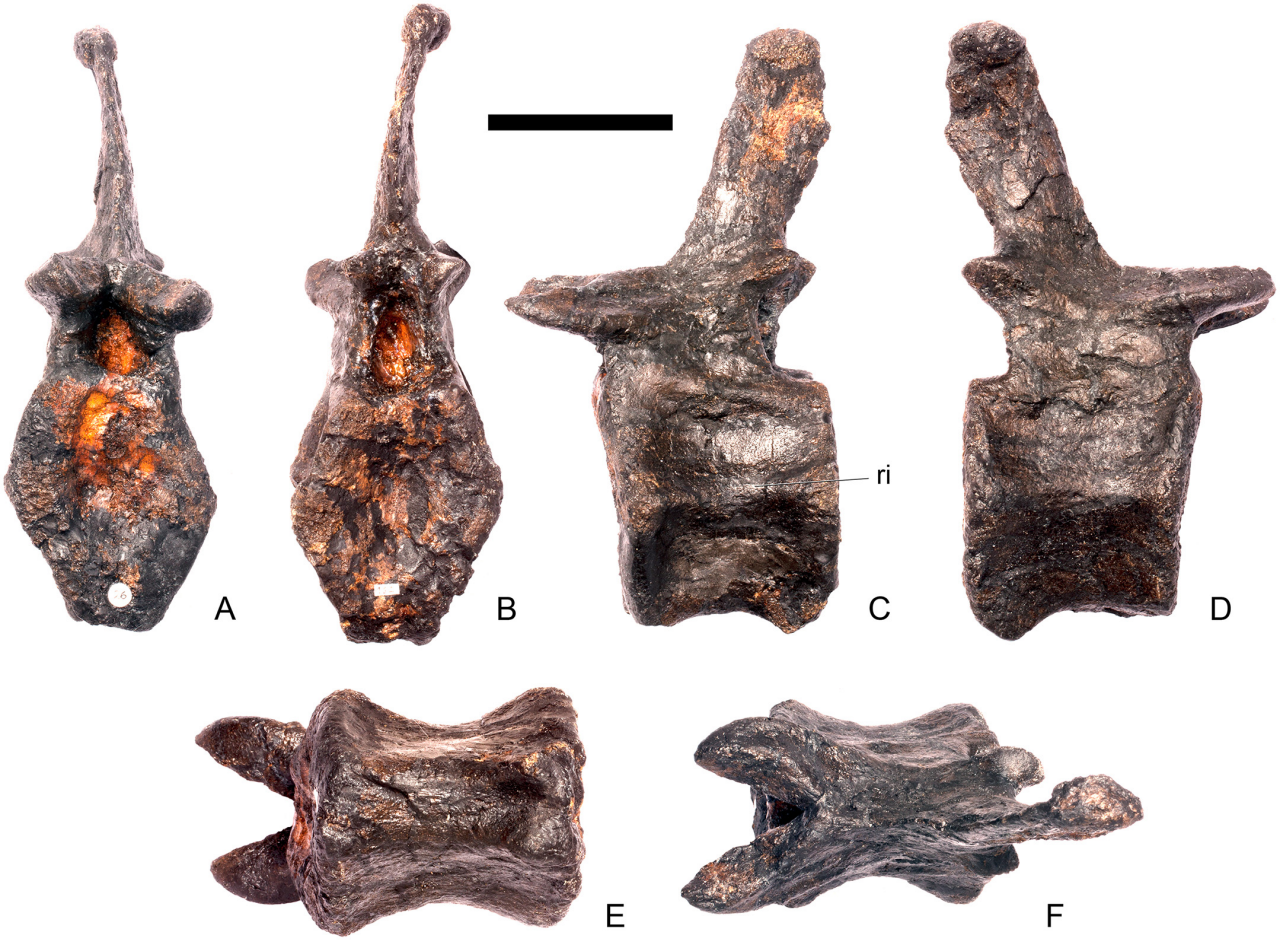
Caudal vertebra 25. **A**, anterior, **B**, posterior, **C**, left lateral, **D**, right lateral, **E**, ventral and **F**, dorsal view. **Ri**, ridge. Scale bar equal to 5 cm.

#### Caudal vertebra 26 (Fig 41)

The ventral margin of the centrum of Cd26 is reconstructed. In all other respects it is the same as Cd25, except that the dorsal part of the neural spine is transversely compressed and not expanded.

**Fig 41:**
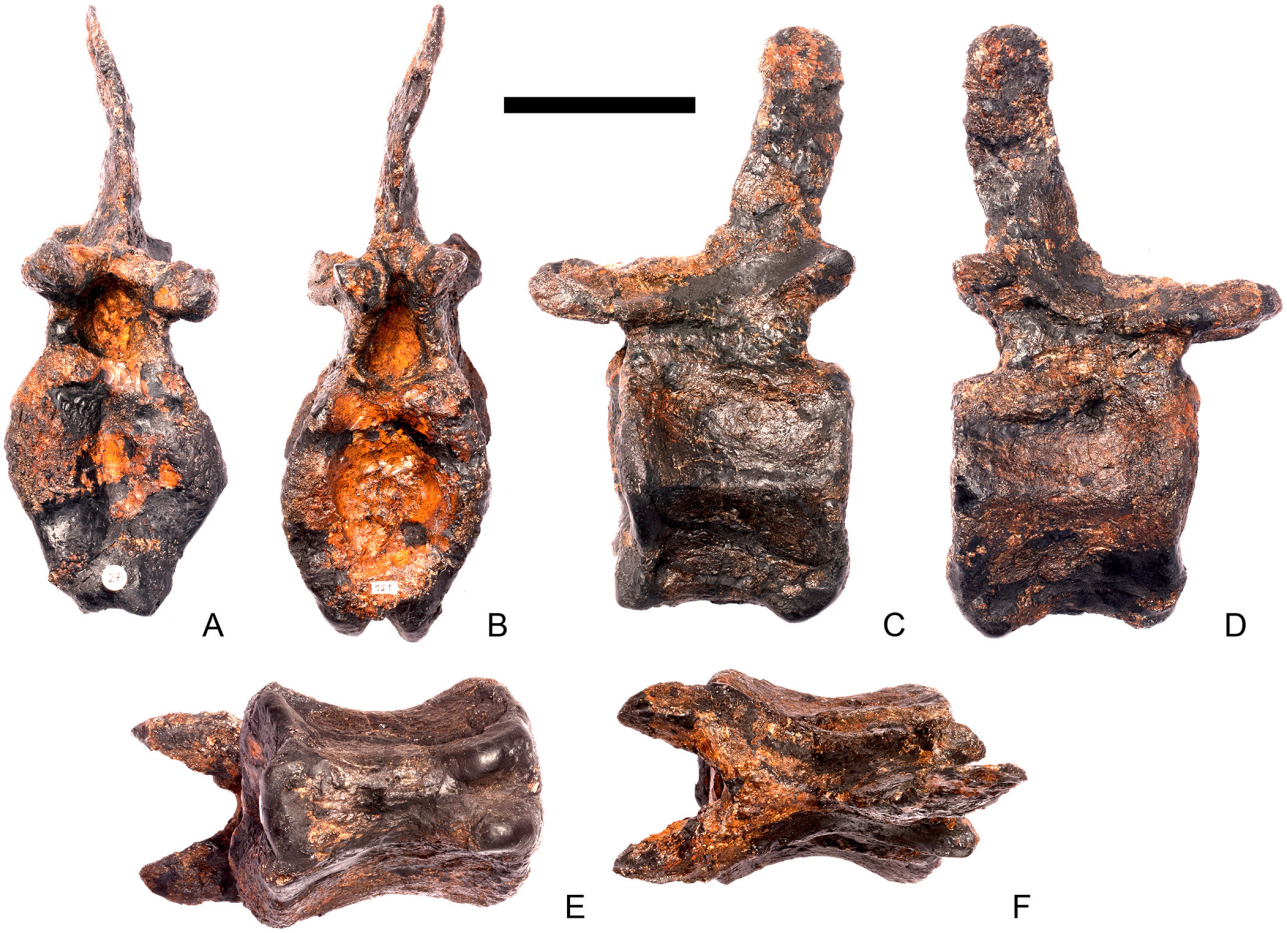
Caudal vertebra 26. **A**, anterior, **B**, posterior, **C**, left lateral, **D**, right lateral, **E**, ventral and **F**, dorsal view. Scale bar equal to 5 cm.

#### Caudal vertebra 27 (Fig 42)

The ventral margin of the centrum and the right prezygapophysis of Cd27 are reconstructed. It is otherwise similar to Cd26 except that the spine is further reduced in height. In lateral view the centrum is square in outline rather than being taller dorsoventrally than long anteroposteriorly.

**Fig 42:**
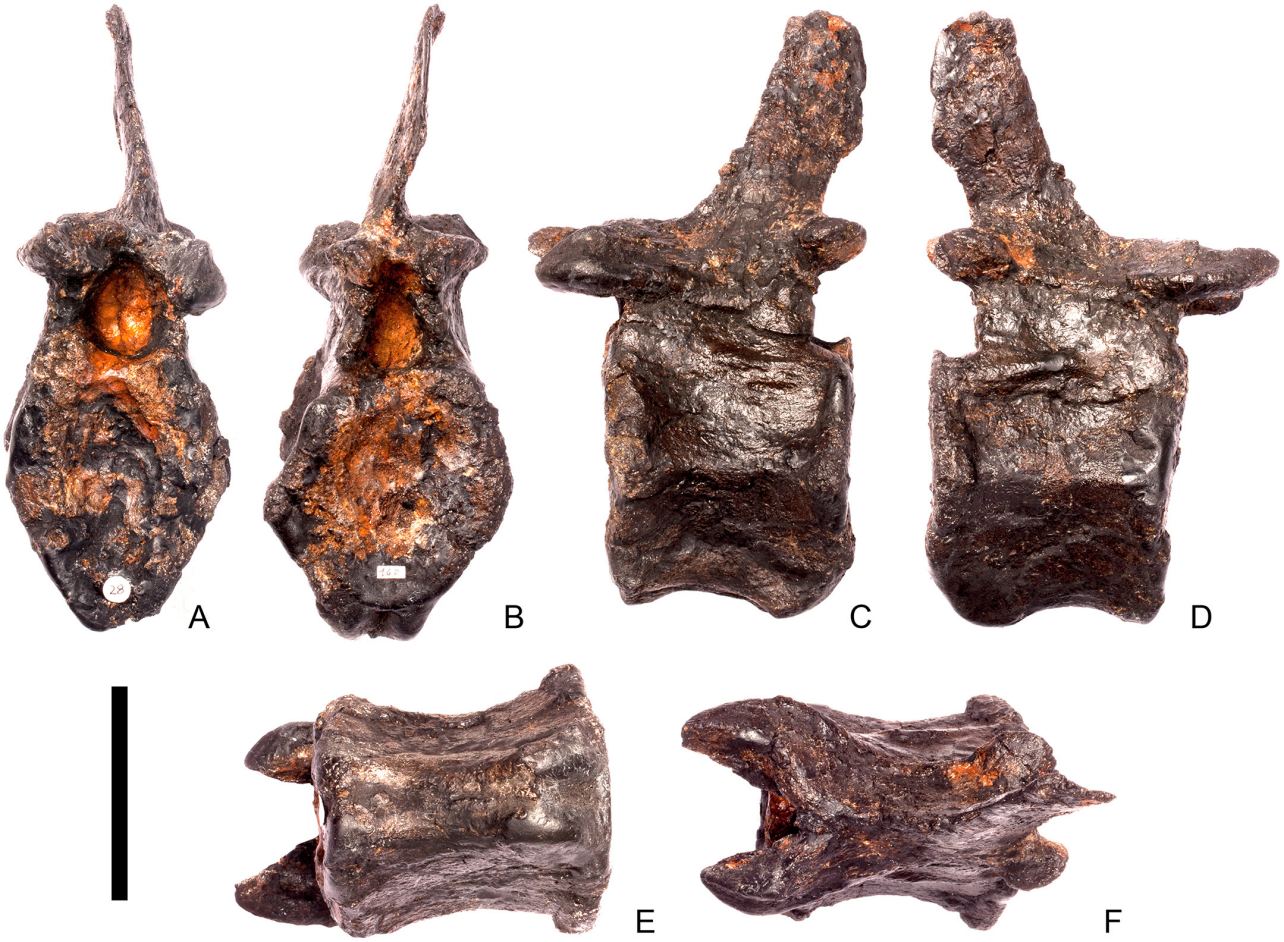
Caudal vertebra 27. **A**, anterior, **B**, posterior, **C**, left lateral, **D**, right lateral, **E**, ventral and **F**, dorsal view. Scale bar equal to 5 cm.

#### Caudal vertebra 28 (Fig 43)

Cd28 is reconstructed ventrally. Compared to Cd27, the only noticeable difference in morphology is that in lateral view, the spine expands anteroposteriorly as it extends dorsally to produce a fan-shaped outline. The spine is also relatively and absolutely shorter than in preceding vertebrae.

**Fig 43:**
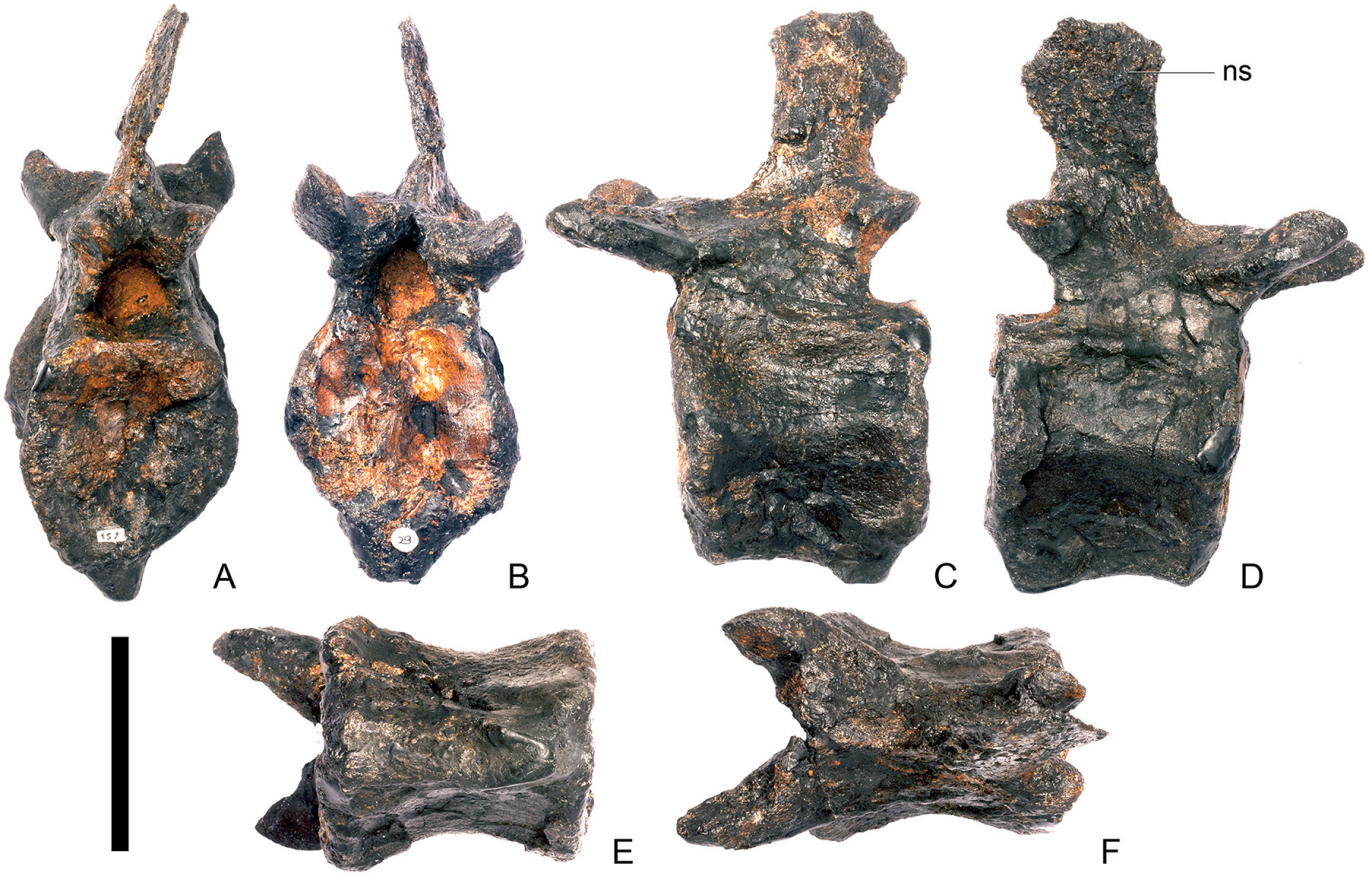
Caudal vertebra 28. **A**, anterior, **B**, posterior, **C**, left lateral, **D**, right lateral, **E**, ventral and **F**, dorsal view. **Ns**, neural spine. Scale bar equal to 5 cm.

#### Caudal vertebra 29 (Fig 44)

Cd29 is very similar in morphology to Cd28. Ventrally, the centrum has been restored and the presence of a chevron facet is therefore difficult to ascertain. The fan-shaped morphology of the neural spine in Cd28 is retained in Cd29. The neural canal is arch-shaped in anterior and posterior view, rather than being teardrop-shaped, as it is in preceding vertebrae.

**Fig 44:**
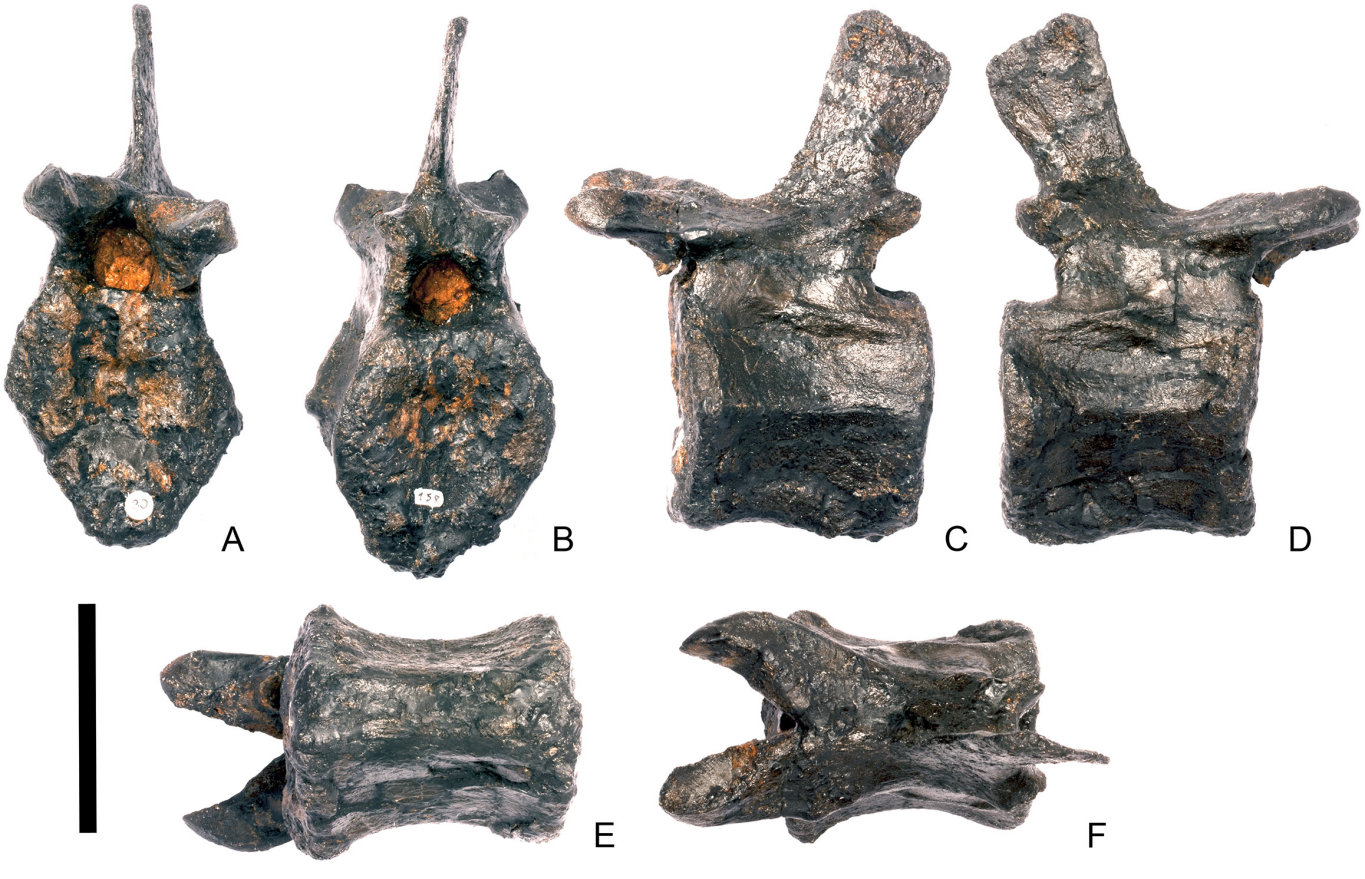
Caudal vertebra 29. **A**, anterior, **B**, posterior, **C**, left lateral, **D**, right lateral, **E**, ventral and **F**, dorsal view. Scale bar equal to 5 cm.

#### Caudal vertebra 30 (Fig 45)

Cd30 is well preserved. It is similar to Cd29, but more anatomical details can be determined. A posterior chevron facet is present ventrally on the centrum. The neural spine is reduced in height and rectangular in lateral view with sub-parallel anterior and posterior margins, in contrast to the condition in Cd28 and 29, where it was fan-shaped. The neural canal is round anteriorly and arch-shaped posteriorly.

**Fig 45:**
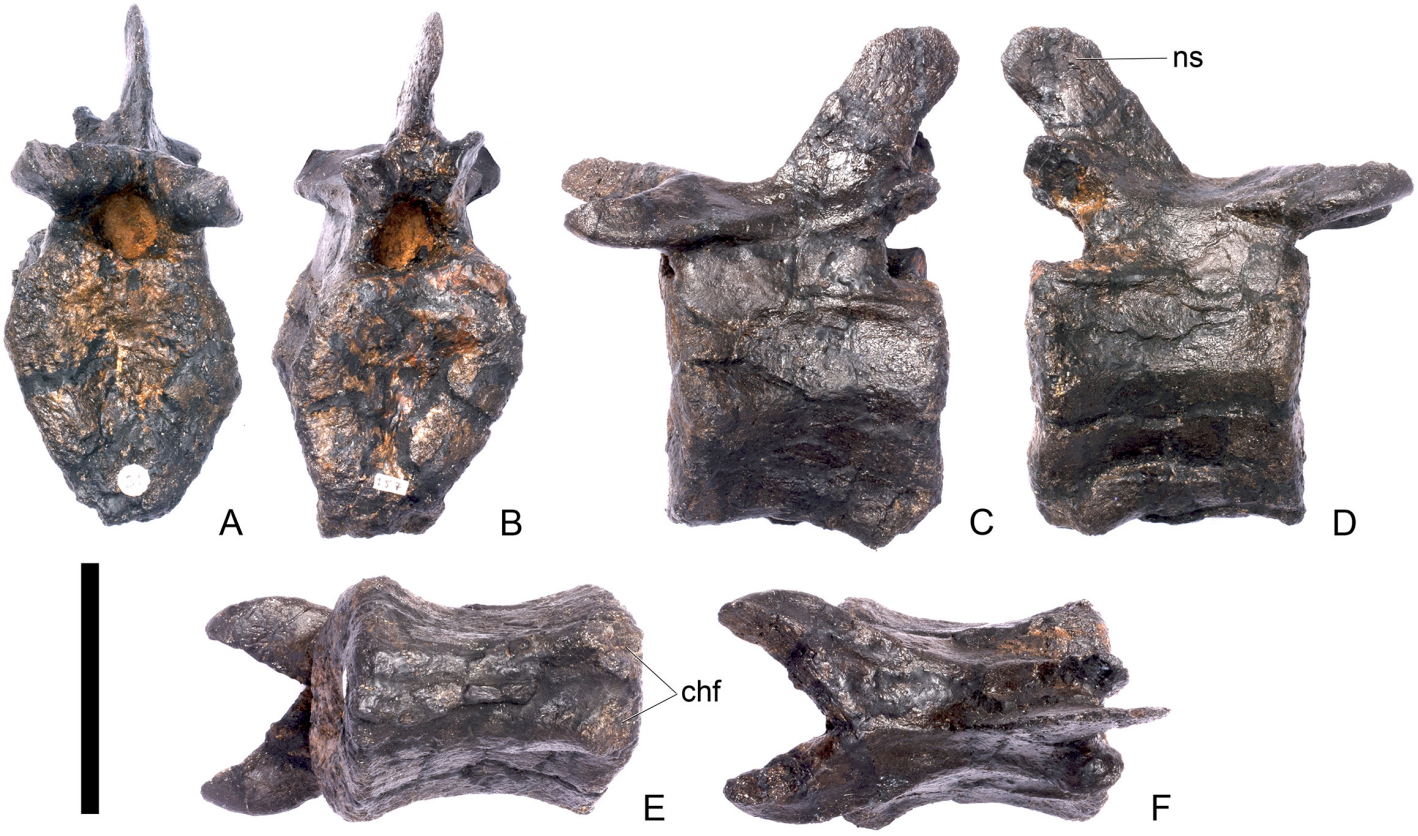
Caudal vertebra 30. **A**, anterior, **B**, posterior, **C**, left lateral, **D**, right lateral, **E**, ventral and **F**, dorsal view. **Chf**, chevron facet; **ns**, neural spine. Scale bar equal to 5 cm.

#### Caudal vertebra 31 (Fig 46)

Cd31 is similar to preceding vertebrae except that the neural spine is slightly fan shaped with a slight anteroposterior expansion at the tip, similar to that in Cd28 and 29 but differing from the condition in Cd30. Very reduced swellings on the lateral surface of the centrum represent the remnants of caudal ribs.

**Fig 46:**
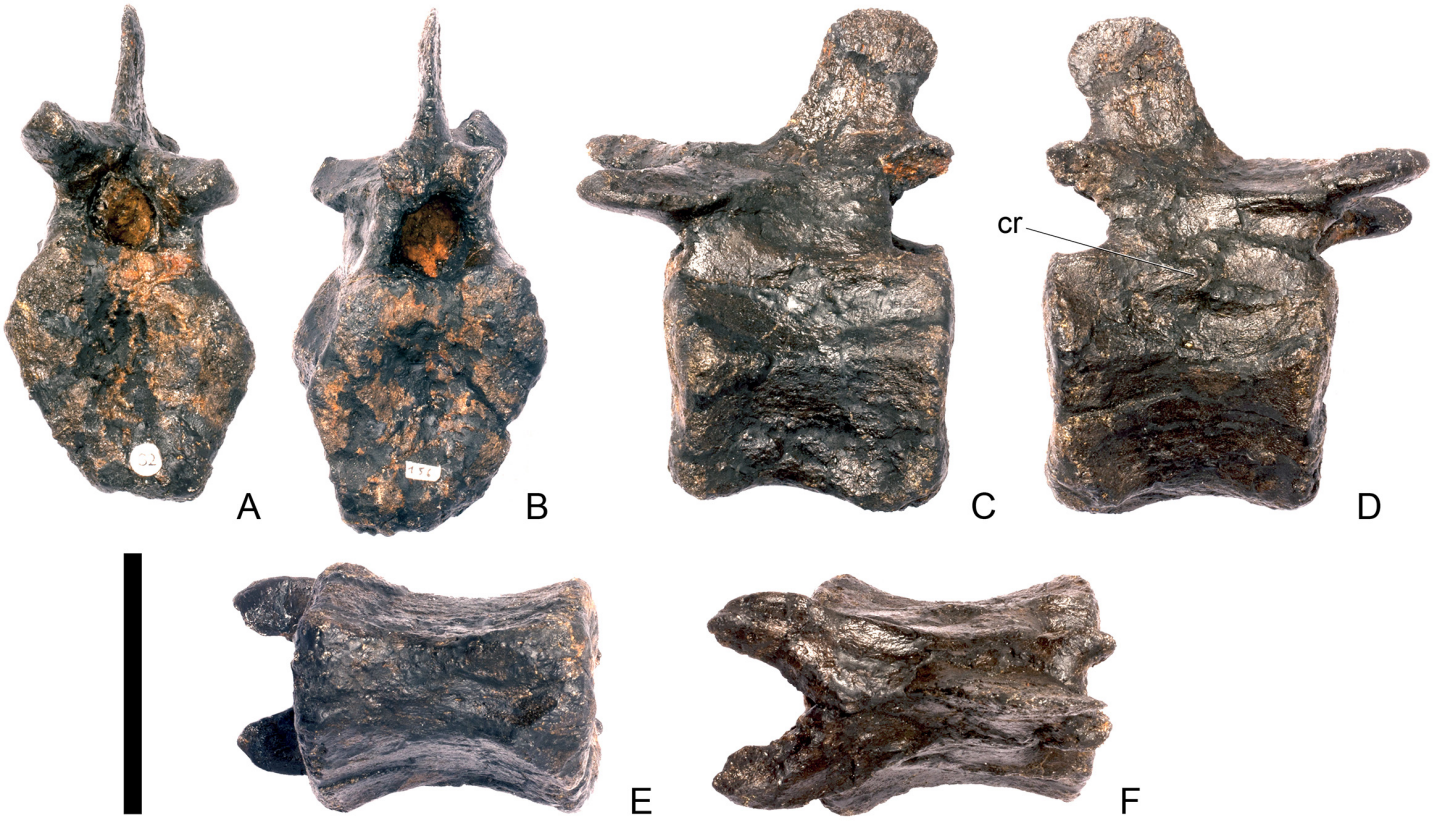
Caudal vertebra 31. **A**, anterior, **B**, posterior, **C**, left lateral, **D**, right lateral, **E**, ventral and **F**, dorsal view. **Cr**, caudal rib. Scale bar equal to 5 cm.

#### Caudal vertebra 32 (Fig 47)

Cd32 is not as well preserved as preceding vertebrae and is missing the postzygapophyses and most of the neural spine. The anteroposteriorly extending ridge on the lateral surface of the centrum has continued to move dorsally so that in Cd32 it is located close to the presumed location of the neurocentral suture. Consequently, the articular facets of the centrum are heart-shaped. The neural canal is round both anteriorly and posteriorly.

**Fig 47:**
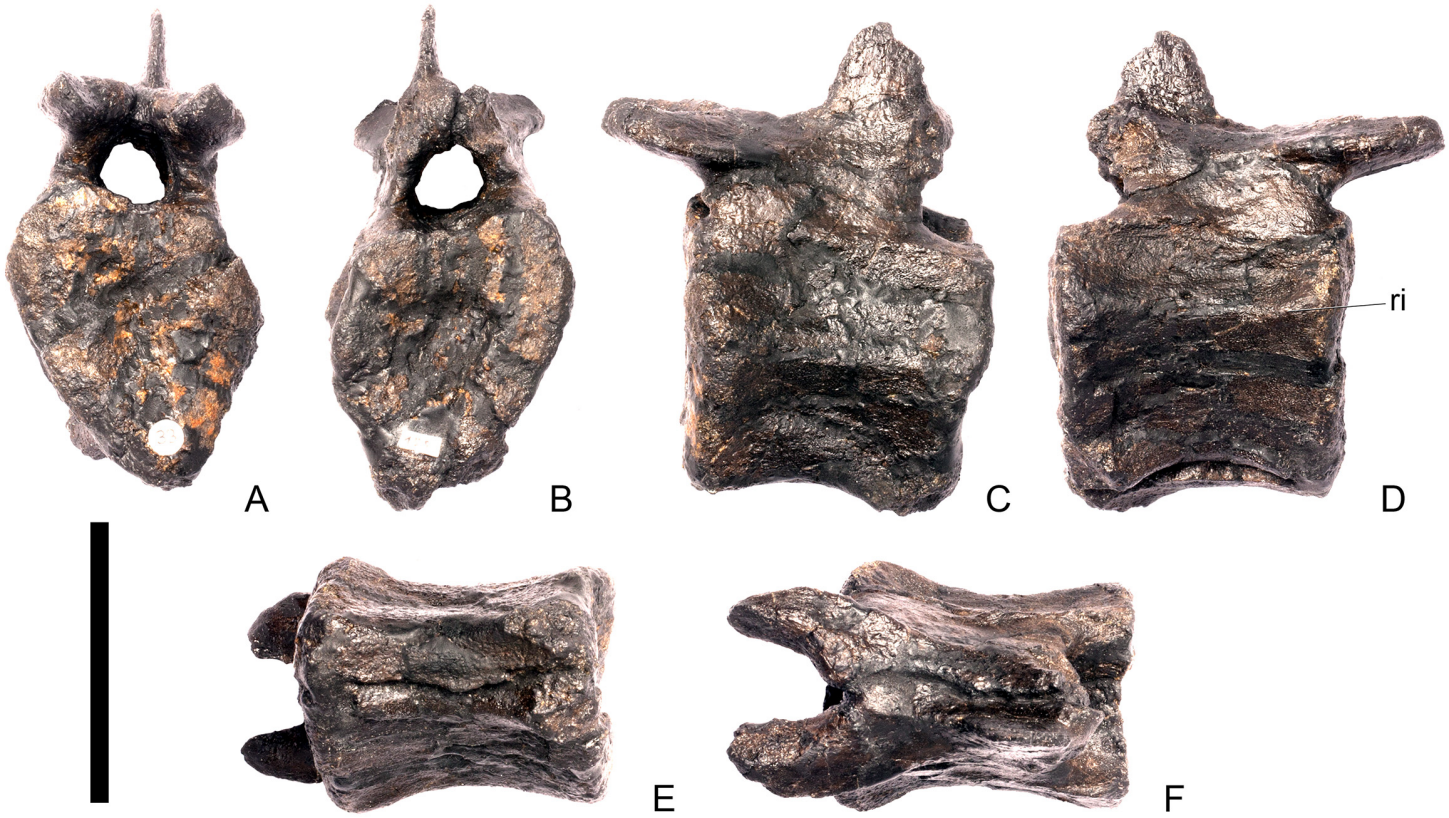
Caudal vertebra 32. **A**, anterior, **B**, posterior, **C**, left lateral, **D**, right lateral, **E**, ventral and **F**, dorsal view. **Ri**, ridge. Scale bar equal to 5 cm.

#### Caudal vertebra 33 (Fig 48)

The centrum of Cd33 is damaged ventrally and the prezygapophyses are broken. Otherwise, it is similar in morphology to the preceding vertebrae, differing from them only in that the neural spine is reduced to a low sub-triangular plate. Postzygapophyses are reduced to small, flange-like processes.

**Fig 48:**
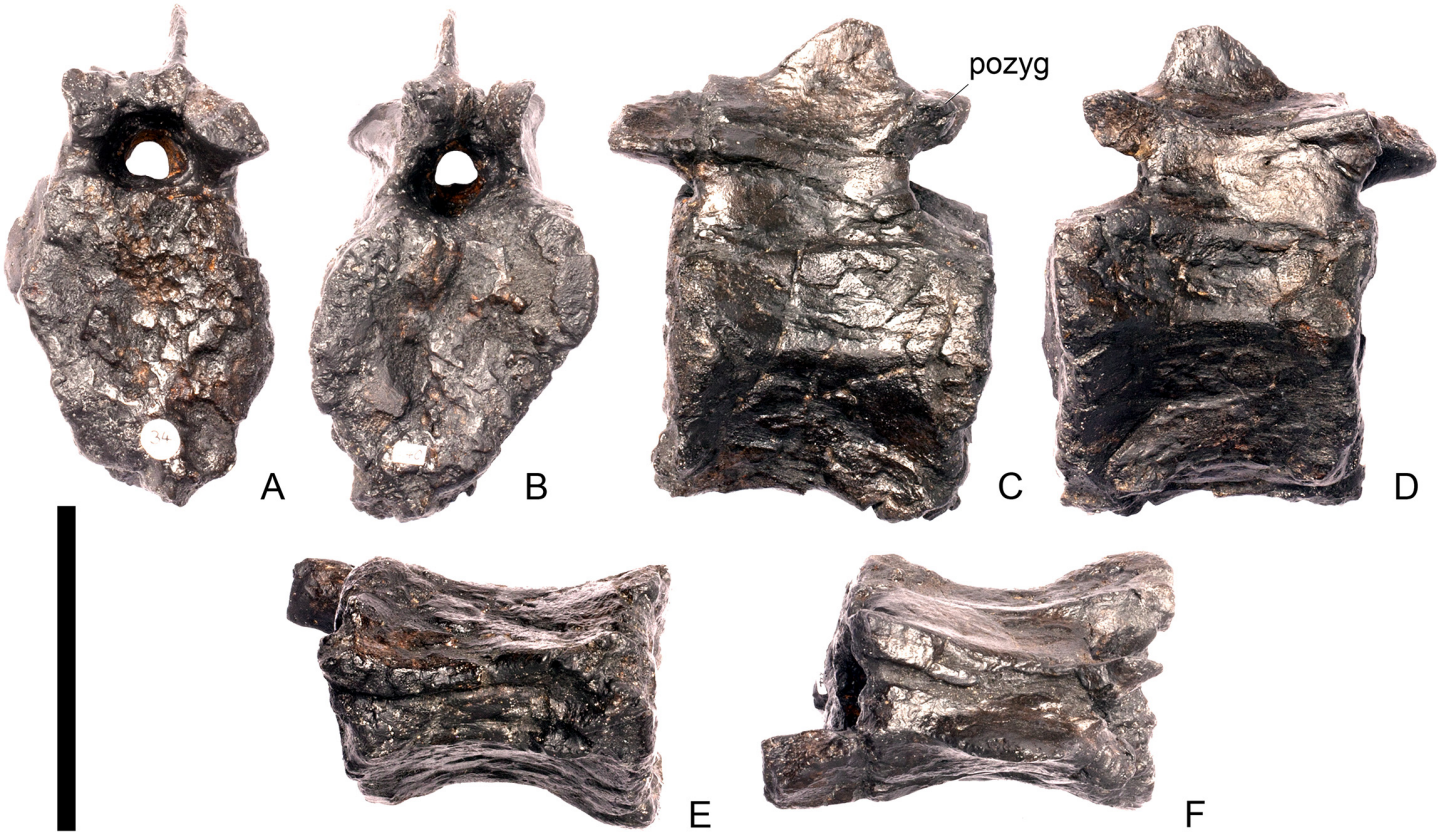
Caudal vertebra 33. **A**, anterior, **B**, posterior, **C**, left lateral, **D**, right lateral, **E**, ventral and **F**, dorsal view. **Pozyg**, postzygapophysis. Scale bar equal to 5 cm.

#### Caudal vertebra 34 (Fig 49)

Cd34 is damaged ventrally, but there is some indication of a chevron facet. The centrum is longer anteroposteriorly than it is tall dorsoventrally, and it is taller dorsoventrally than it is wide transversely. The prezygapophyses are elongate finger-like processes, as in preceding vertebrae, while the postzygapophyses are much reduced but still present. The spine is reduced to a low rounded ridge.

**Fig 49:**
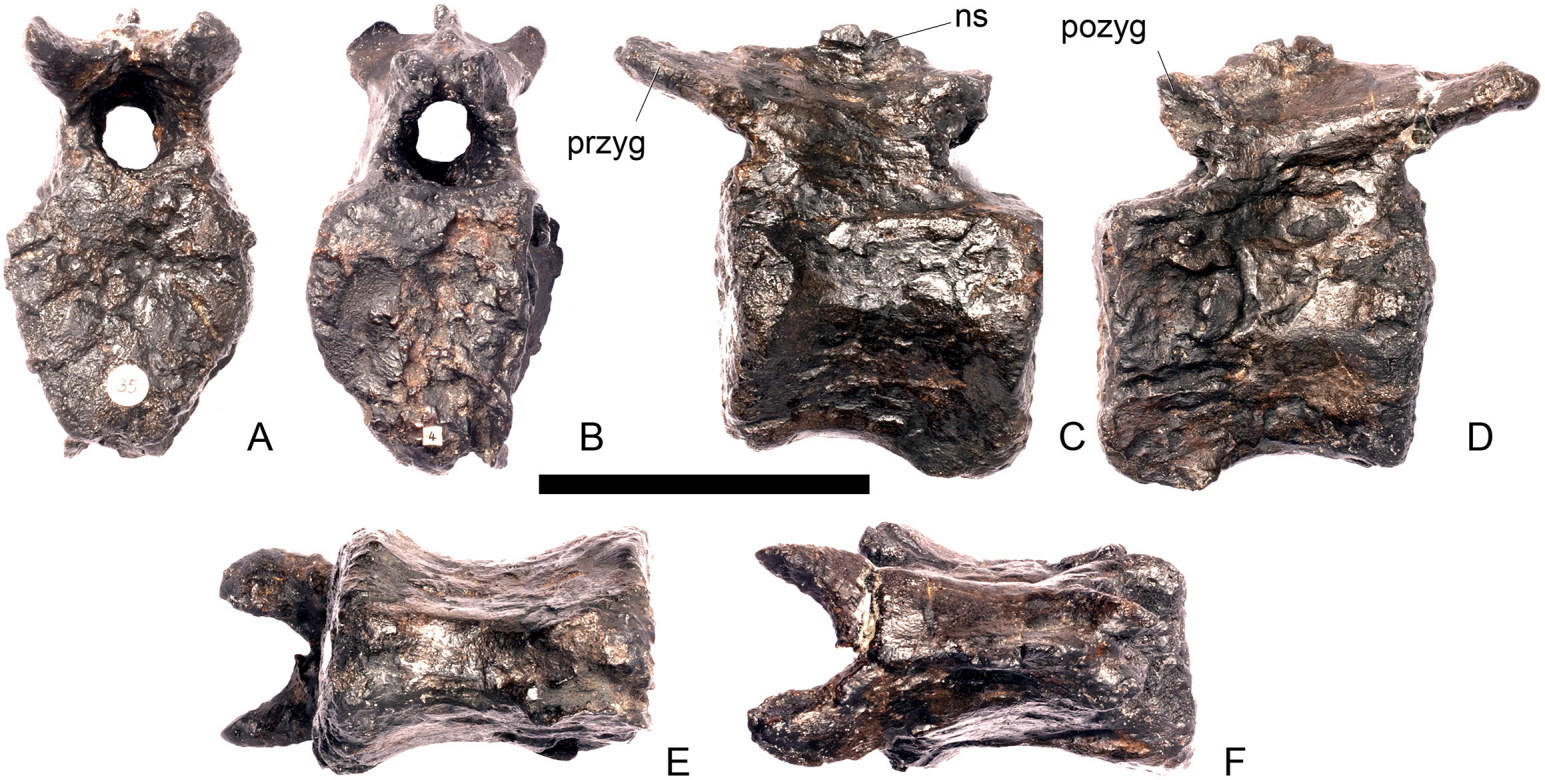
Caudal vertebra 34. **A**, anterior, **B**, posterior, **C**, left lateral, **D**, right lateral, **E**, ventral and **F**, dorsal view. **Ns**, neural spine; **pozyg**, postzygapophysis; **przyg**, prezygapophysis. Scale bar equal to 5 cm.

#### Caudal vertebra 35 (Fig 50)

Cd35 is similar to Cd34 except that the anteroposteriorly extending ridges on the lateral surfaces of the centrum are reduced in prominence. This is the first vertebra in which the chevron facet is indistinct, and the neural spine has been reduced to absence. [2] suggested that the neural spines disappeared on Cd37 or 38, but did not have a complete, articulated tail at his disposal.

**Fig 50:**
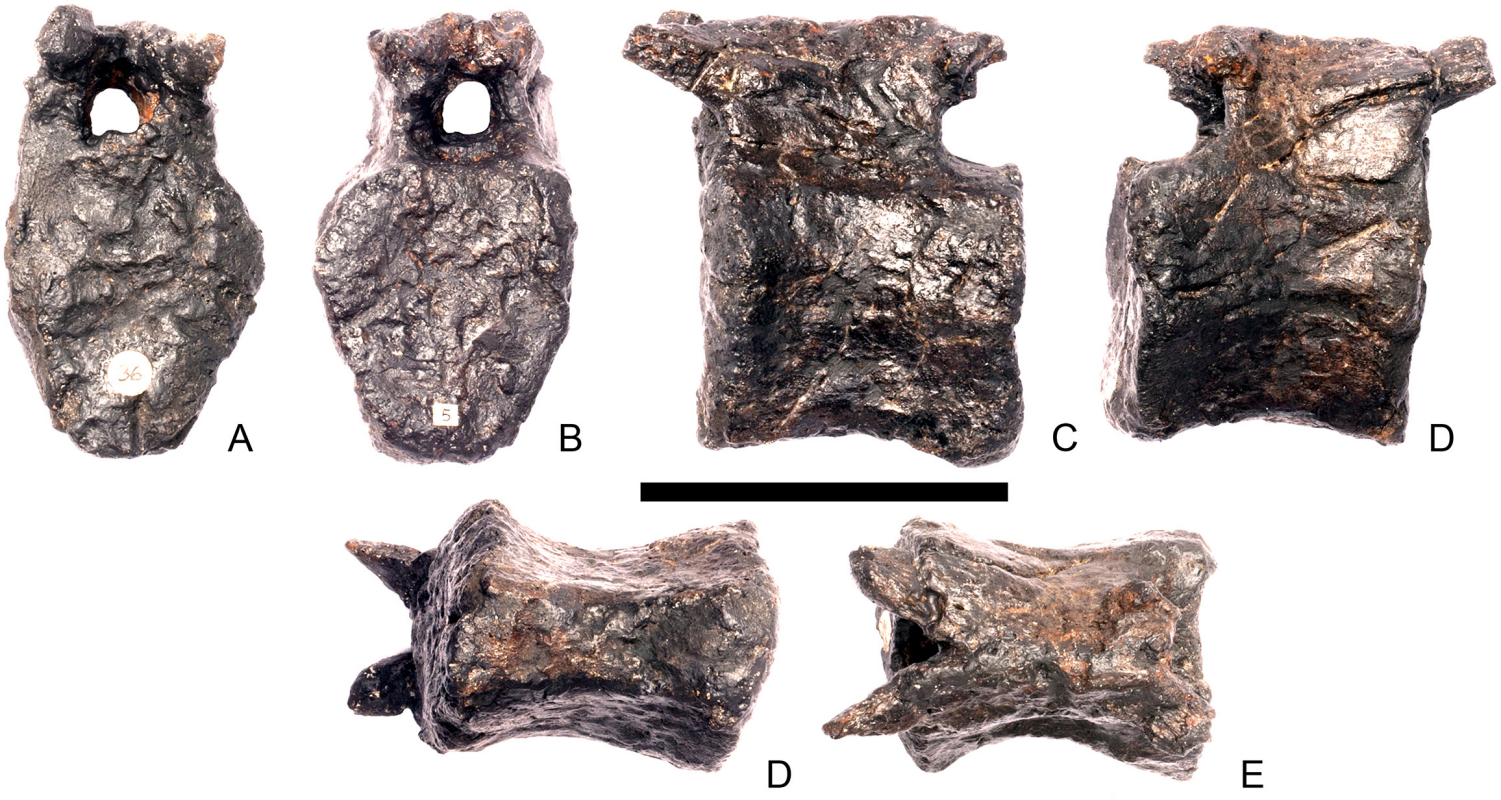
Caudal vertebra 35. **A**, anterior, **B**, posterior, **C**, left lateral, **D**, right lateral, **E**, ventral and **F**, dorsal view. Scale bar equal to 5 cm.

#### Caudal vertebra 36 (Fig 51)

**Fig 51:**
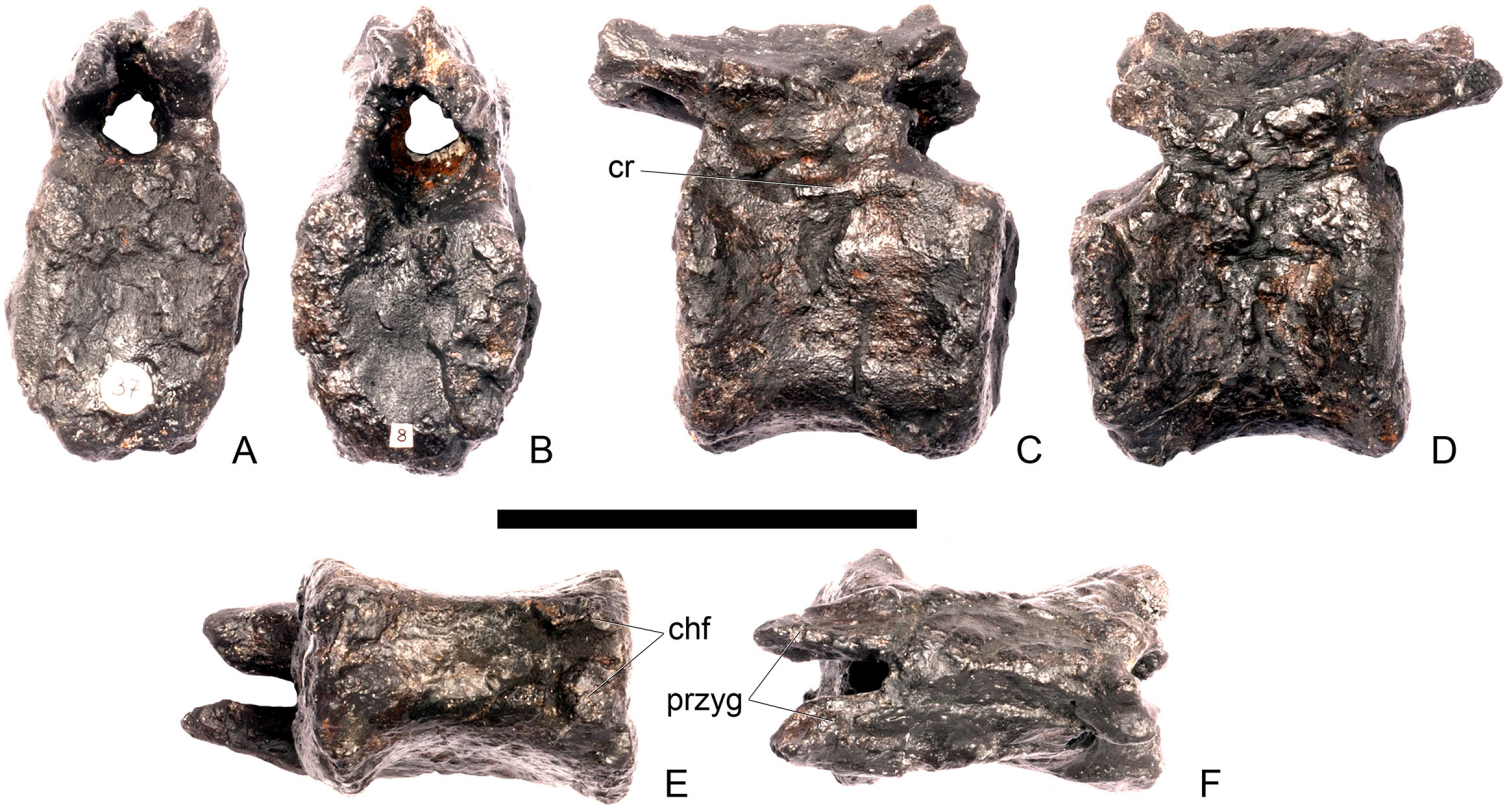
Caudal vertebra 36. **A**, anterior, **B**, posterior, **C**, left lateral, **D**, right lateral, **E**, ventral and **F**, dorsal view. **chf**, chevron facet; **cr**, caudal rib; **przyg**, prezygapophysis. Scale bar equal to 5 cm.

The anterior and posterior articular surfaces of the centrum of Cd36 are sub-elliptical in outline (Fig 51A and 51B). In lateral view, subtle ridges on the centrum that may represent the remnants of caudal ribs are still present (Fig 51C and 51D). In ventral view, there is a very faint bifurcation of posterior surface of the centrum, which may represent the remnants of a chevron facet (Fig 51E). The ventral surface of the centrum appears broader than in preceding vertebrae, and bears a shallow anteroposterior groove along its length (Fig 51E). The prezygapophyses extend almost parallel to each other in dorsal view, rather than being separated by a ‘V’-shaped cleft as in all preceding caudals (Fig 51F). As in preceding vertebrae, postzygapophyses are much reduced and the neural spine is absent.

#### Caudal vertebra 37 (Fig 52)

Cd37 is identical in all respects to Cd36. Ventrally, the chevron facet is absent. [2] noted that in USNM 4714, the third caudal from the end of the tail had an articulating chevron, so although chevron facets are no longer visible, it is likely that chevrons continued to the end of the tail.

**Fig 52:**
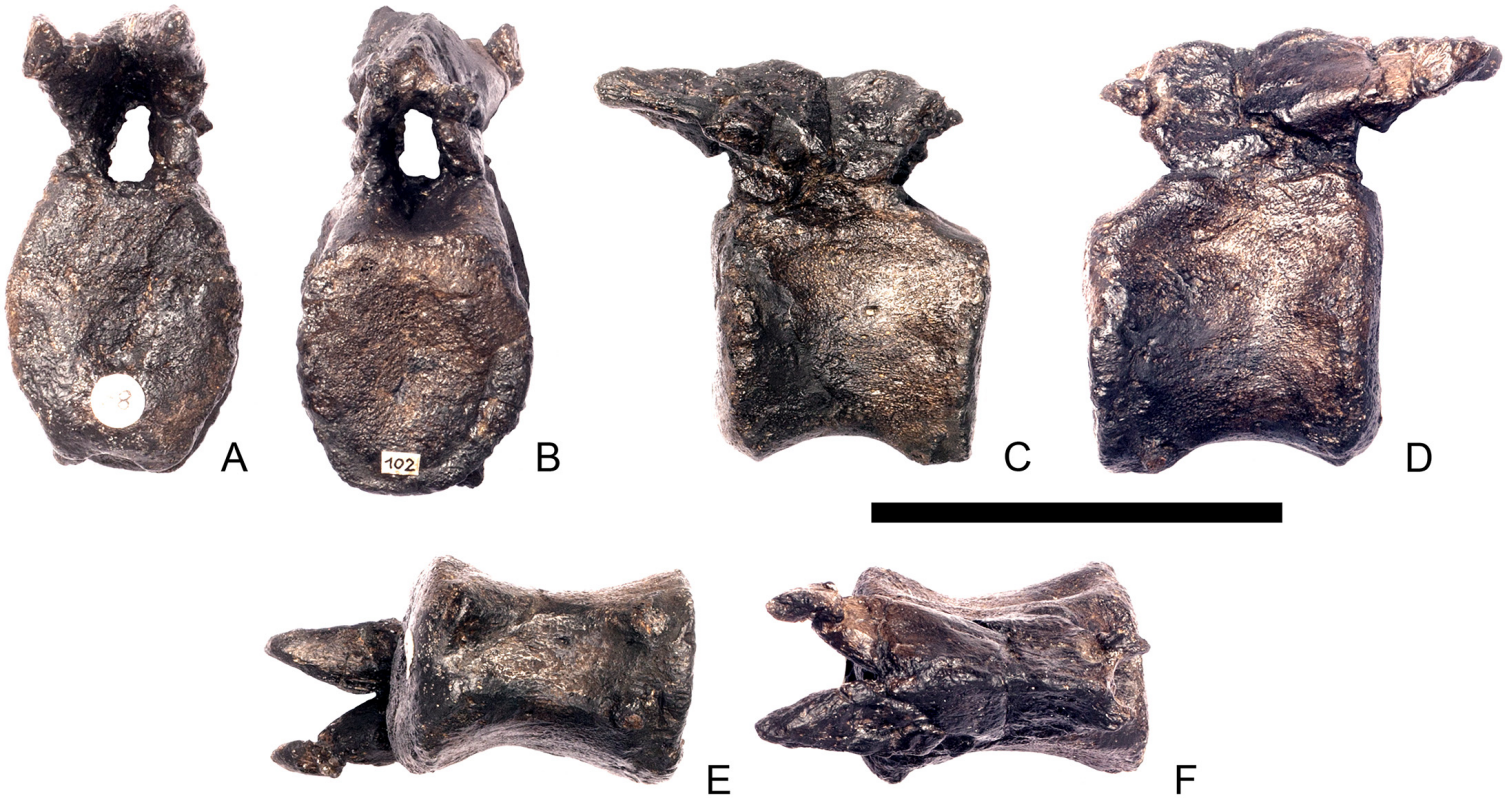
Caudal vertebra 37. **A**, anterior, **B**, posterior, **C**, left lateral, **D**, right lateral, **E**, ventral and **F**, dorsal view. Scale bar equal to 5 cm.

#### Caudal vertebra 39 (Fig 53)

Cd39 is poorly preserved, precluding many observations. The centrum is partially reconstructed and the prezygapophyses are broken, however they appear to have been flattened flanges of bone that would have extended further anteriorly than the anterior margin of the centrum. Otherwise, the vertebra appears similar to Cd37.

**Fig 53:**
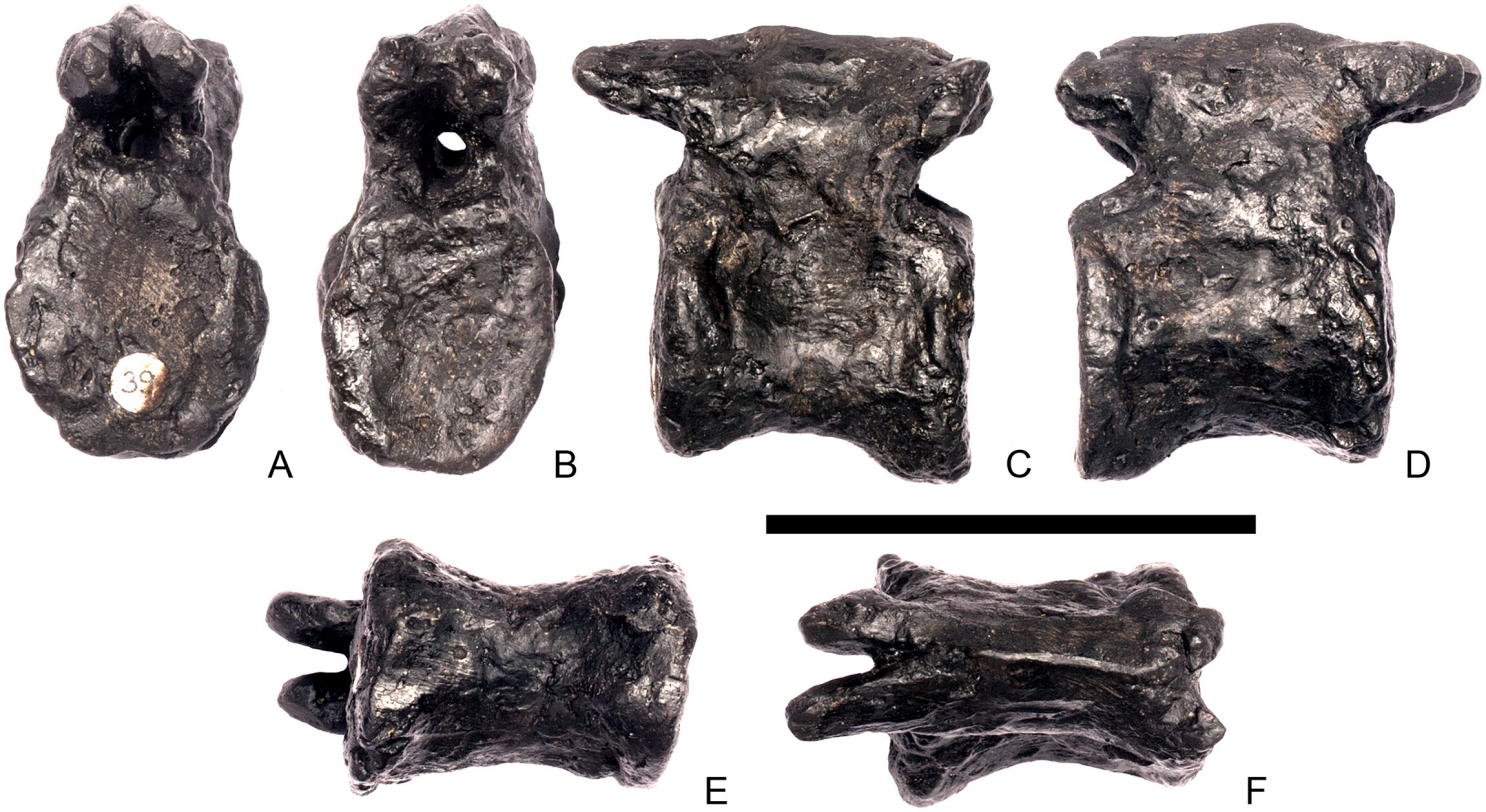
Caudal vertebra 39. **A**, anterior, **B**, posterior, **C**, left lateral, **D**, right lateral, **E**, ventral and **F**, dorsal view. Scale bar equal to 5 cm.

#### Caudal vertebra 40 (Fig 54)

Cd40 is only partially preserved: the anterior part of the neural arch and prezygapophyses are broken. The centrum has sub-circular to sub-quadrate anterior and posterior articular surfaces and lacks longitudinal ridges laterally. A ventral groove is present that extends along the entire length of the centrum, as in preceding vertebrae. The postzygapophyses are fused together to form a posteriorly projecting plate that is concave upwards.

**Fig 54:**
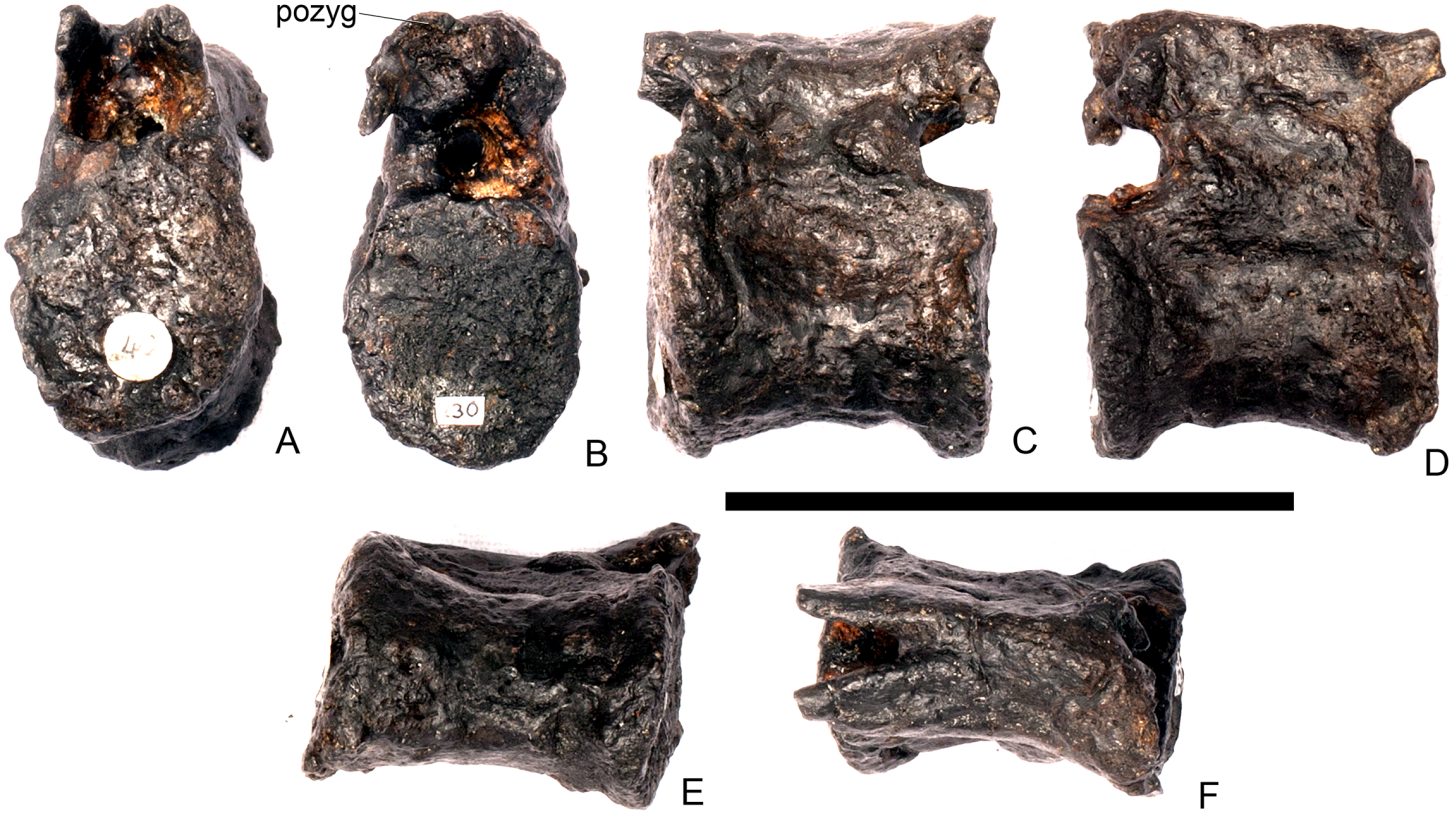
Caudal vertebra 40. **A**, anterior, **B**, posterior, **C**, left lateral, **D**, right lateral, **E**, ventral and **F**, dorsal view. **Pozgy**, postzygapophysis. Scale bar equal to 5 cm.

#### Caudal vertebra 41 (Fig 55)

The neural arch of Cd41 is broken. The centrum appears to be identical in all respects to that of Cd40.

**Fig 55:**
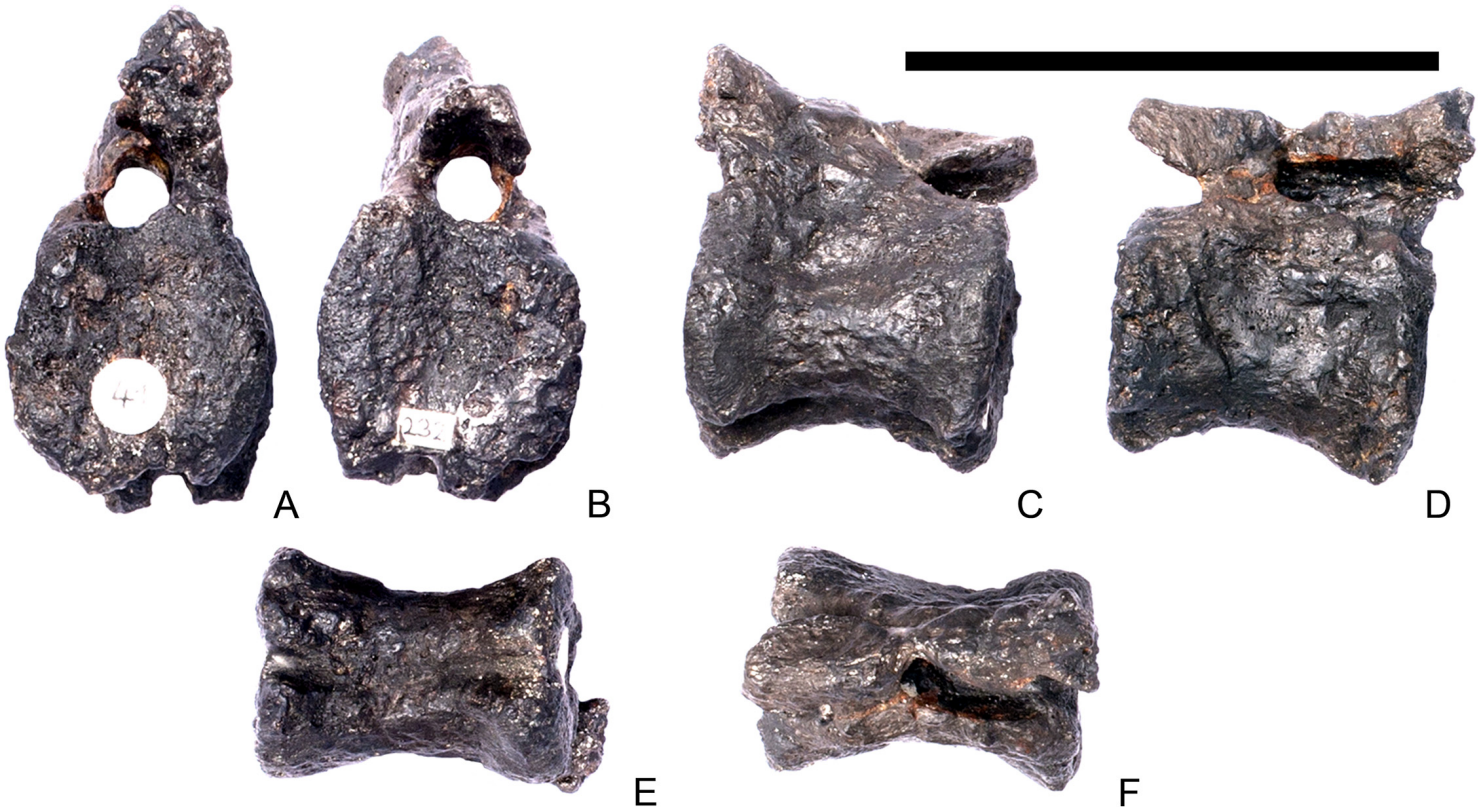
Caudal vertebra 41. **A**, anterior, **B**, posterior, **C**, left lateral, **D**, right lateral, **E**, ventral and **F**, dorsal view. Scale bar equal to 5 cm.

#### Caudal vertebra 42 (Fig 56)

Cd42 is similar to Cd40 and 41; the postzygapophyses are broken. The centrum has a sub-hexagonal outline in anterior and posterior views. A ventral groove is still present. The neural arch is much reduced and the postzygapophyses are combined into a single process.

**Fig 56:**
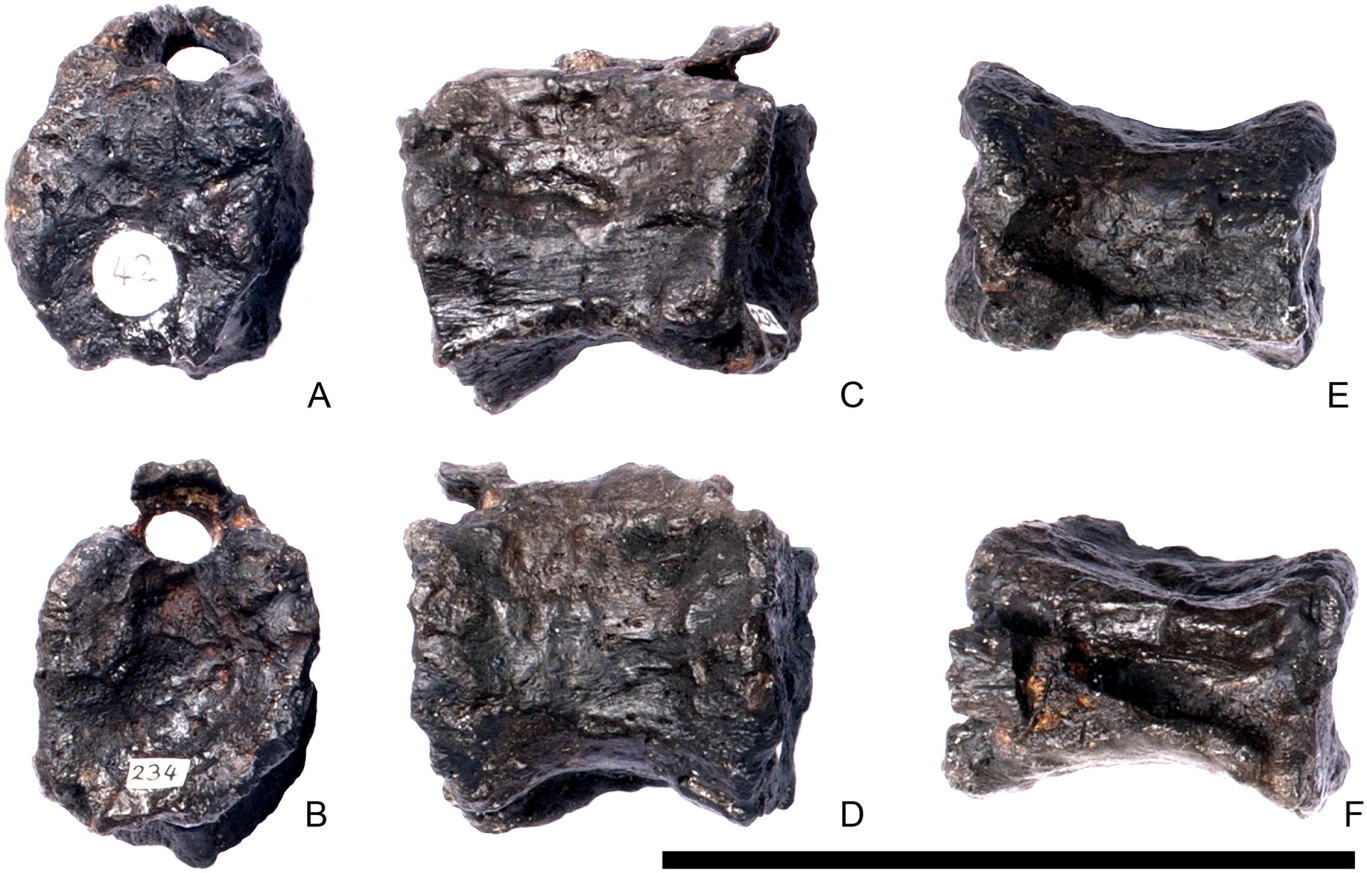
Caudal vertebra 42. **A**, anterior, **B**, posterior, **C**, left lateral, **D**, right lateral, **E**, ventral and **F**, dorsal view. Scale bar equal to 5 cm.

#### Caudal vertebra 43 (Fig 57)

Cd43 is poorly preserved but appears to be similar in all respects to the preceding caudals.

**Fig 57:**
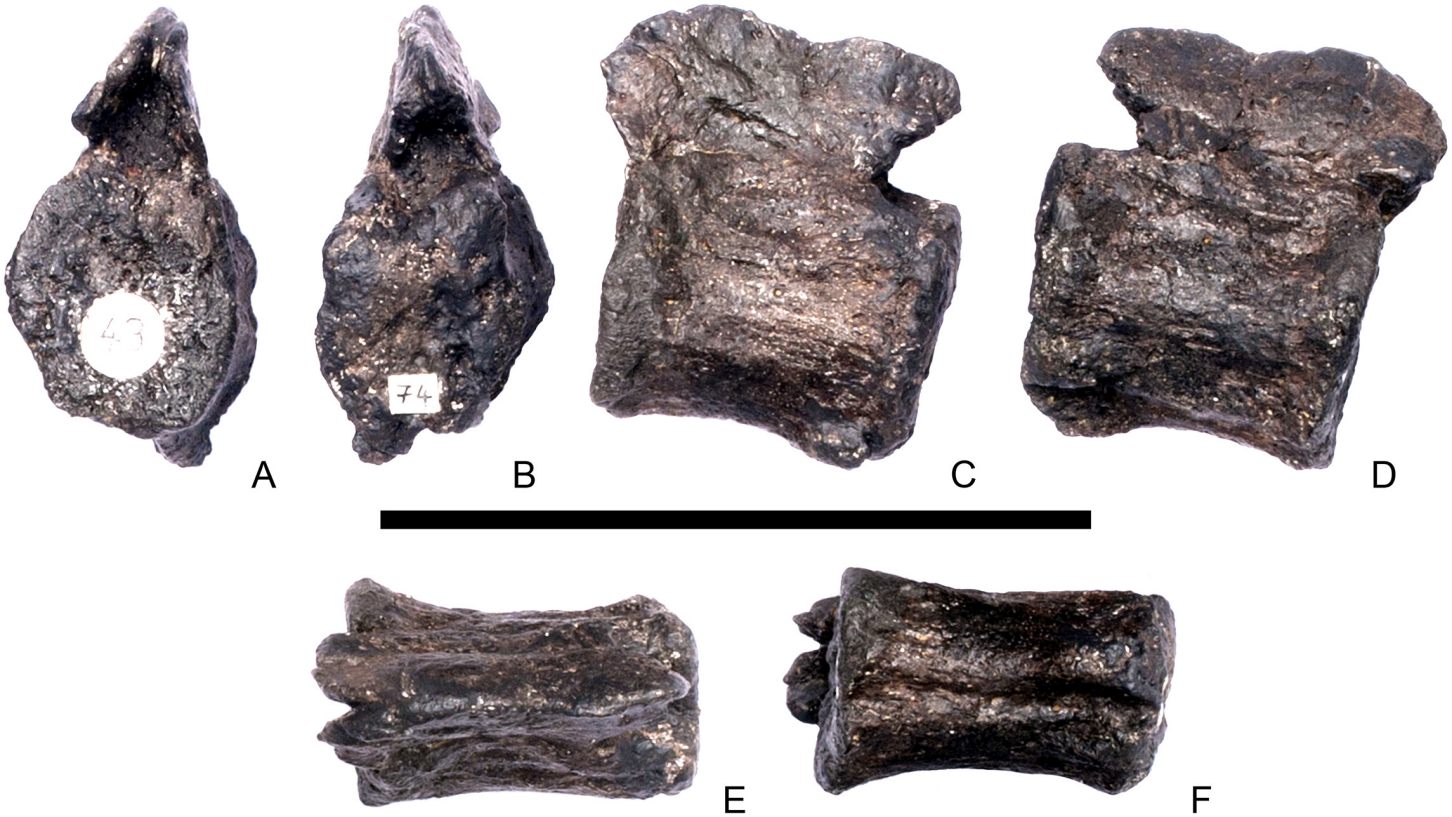
Caudal vertebra 43. **A**, anterior, **B**, posterior, **C**, left lateral, **D**, right lateral, **E**, ventral and **F**, dorsal view. Scale bar equal to 5 cm.

#### Caudal vertebra 44 (Fig 58)

Only the centrum of Cd44 is preserved: the neural arch is broken. The centrum appears to be similar to those of the preceding vertebrae, although there is no groove ventrally.

**Fig 58:**
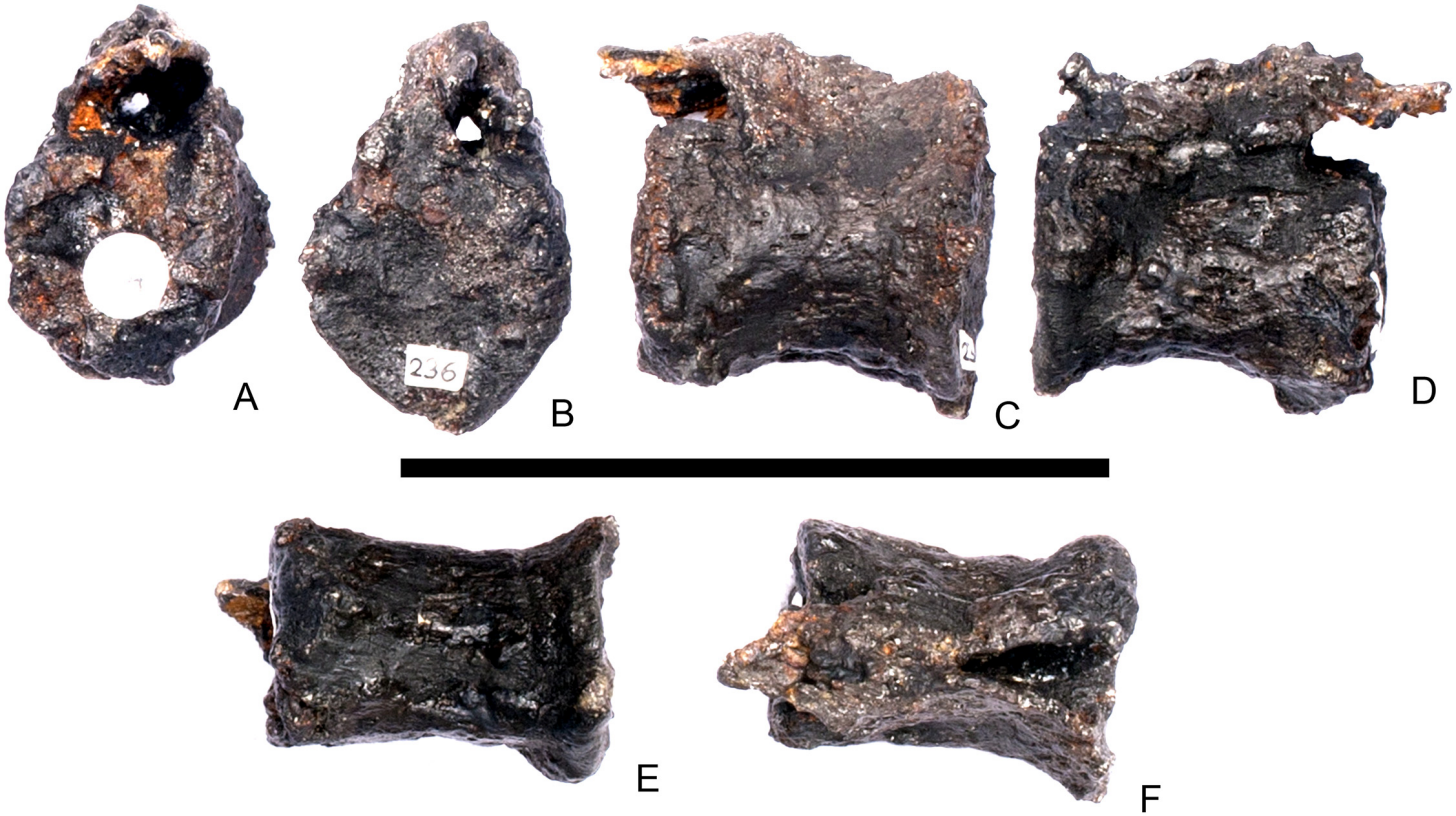
Caudal vertebra 44. **A**, anterior, **B**, posterior, **C**, left lateral, **D**, right lateral, **E**, ventral and **F**, dorsal view. Scale bar equal to 5 cm.

#### Caudal vertebra 45 (Fig 59)

Cd45 is a tiny cylindrical element. A neural arch appears to have been present but is broken.

**Fig 59:**
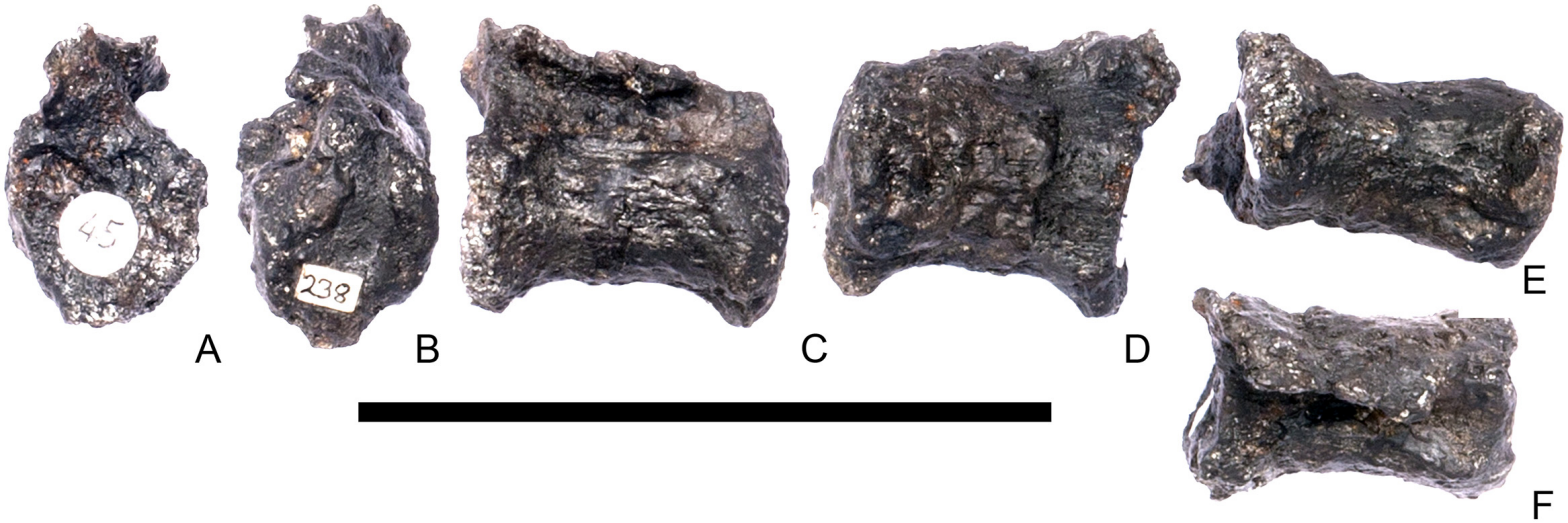
Caudal vertebra 45. **A**, anterior, **B**, posterior, **C**, left lateral, **D**, right lateral, **E**, ventral and **F**, dorsal view. Scale bar equal to 5 cm.

#### Caudal vertebra 46 (Fig 60)

This appears identical to caudal vertebra 45 but is slightly smaller.

**Fig 60:**
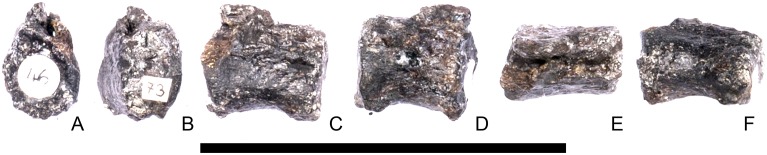
Caudal vertebra 46. **A**, anterior, **B**, posterior, **C**, left lateral, **D**, right lateral, **E**, ventral and **F**, dorsal view. Scale bar equal to 5 cm.

### Ribs (Figs 61–64)

Armature is attached to the medial surfaces of both the cervical and dorsal ribs and, particularly in the dorsal ribs, obscures their anatomy in medial view. By contrast with NHMUK PV R36730, the cervical ribs of *Dacentrurus* sp. (ML 433; [41,42]) are fused to the cervical vertebrae along the entire cervical column. Measurements of ribs can be found in Table 2.

**Fig 61:**
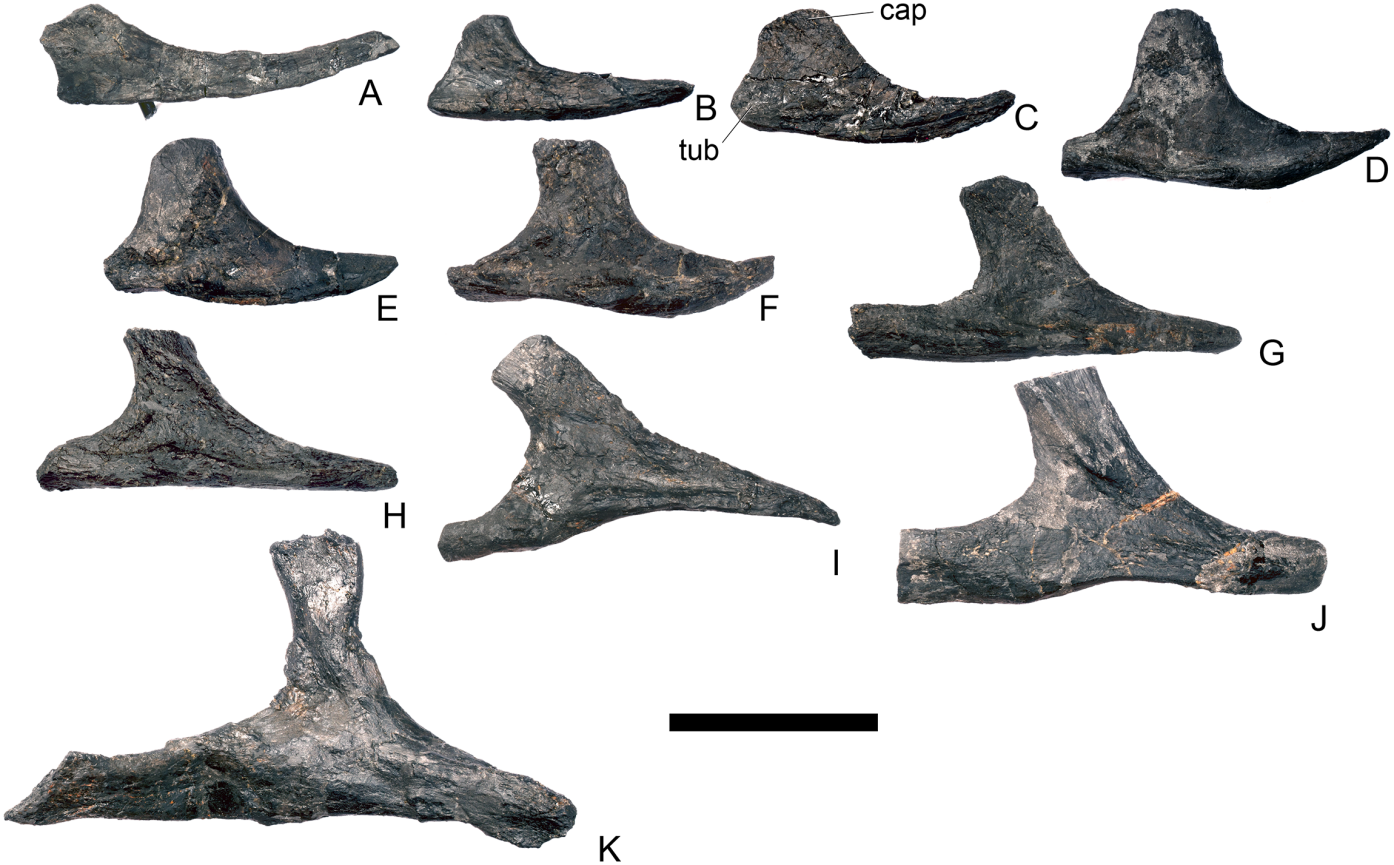
Left cervical ribs in lateral view. **A**, 1; **B**, 2; **C**, 3; **D**, 4; **E**, 5; **F**, 6; **G**, 7; **H**, 8; **I**, 10; **J**, 11; **K**, 13. **Cap**, capitulum; **tub**, tuberculum. Scale bar equal to 5 cm.

**Fig 62:**
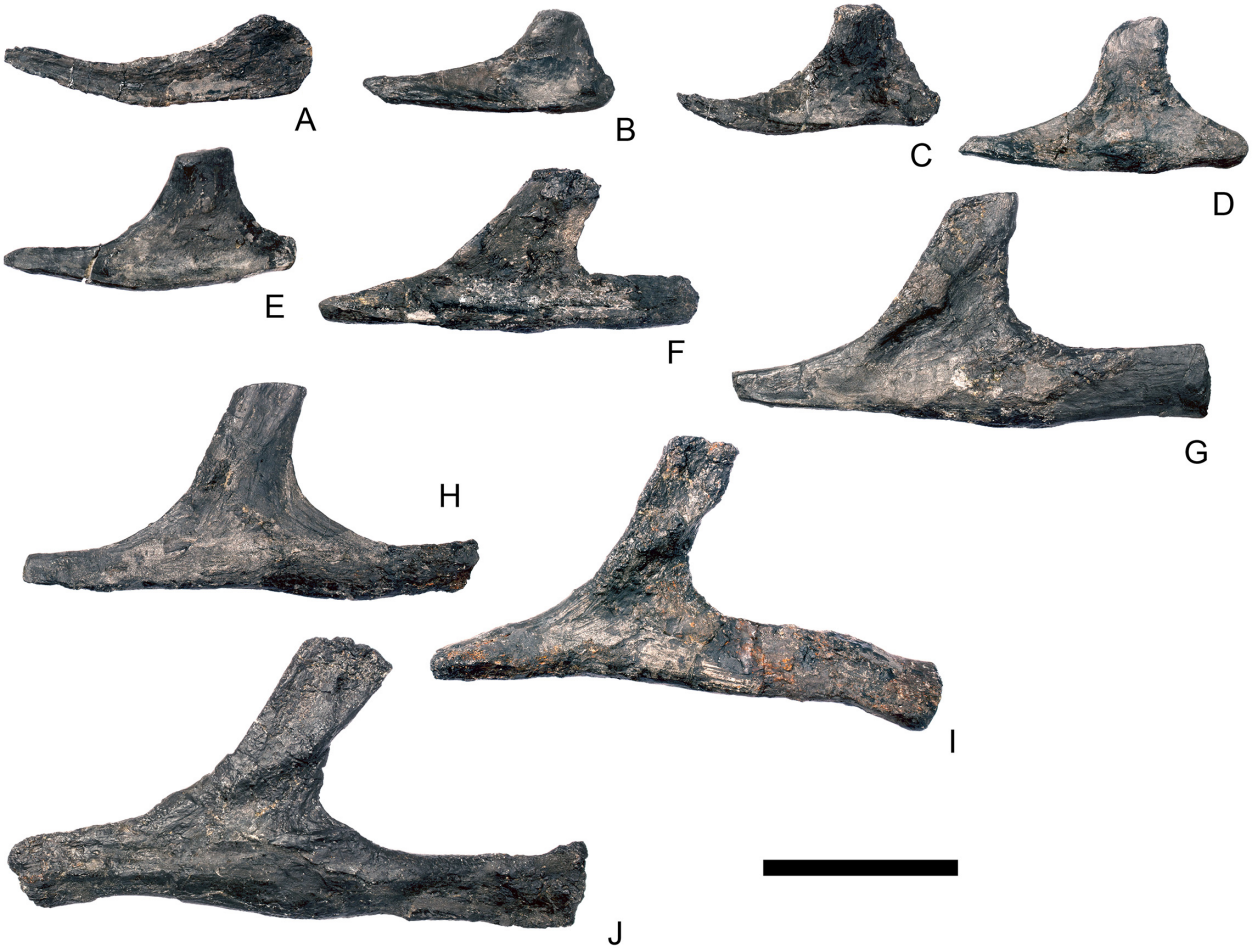
Right cervical ribs in lateral view. **A**, 1; **B**, 2; **C**, 3; **D**, 4; **E**, 5; **F**, 8; **G**, 10; **H**, 11; **I**, 12; **J**, 13. Scale bar equal to 5 cm.

**Fig 63:**
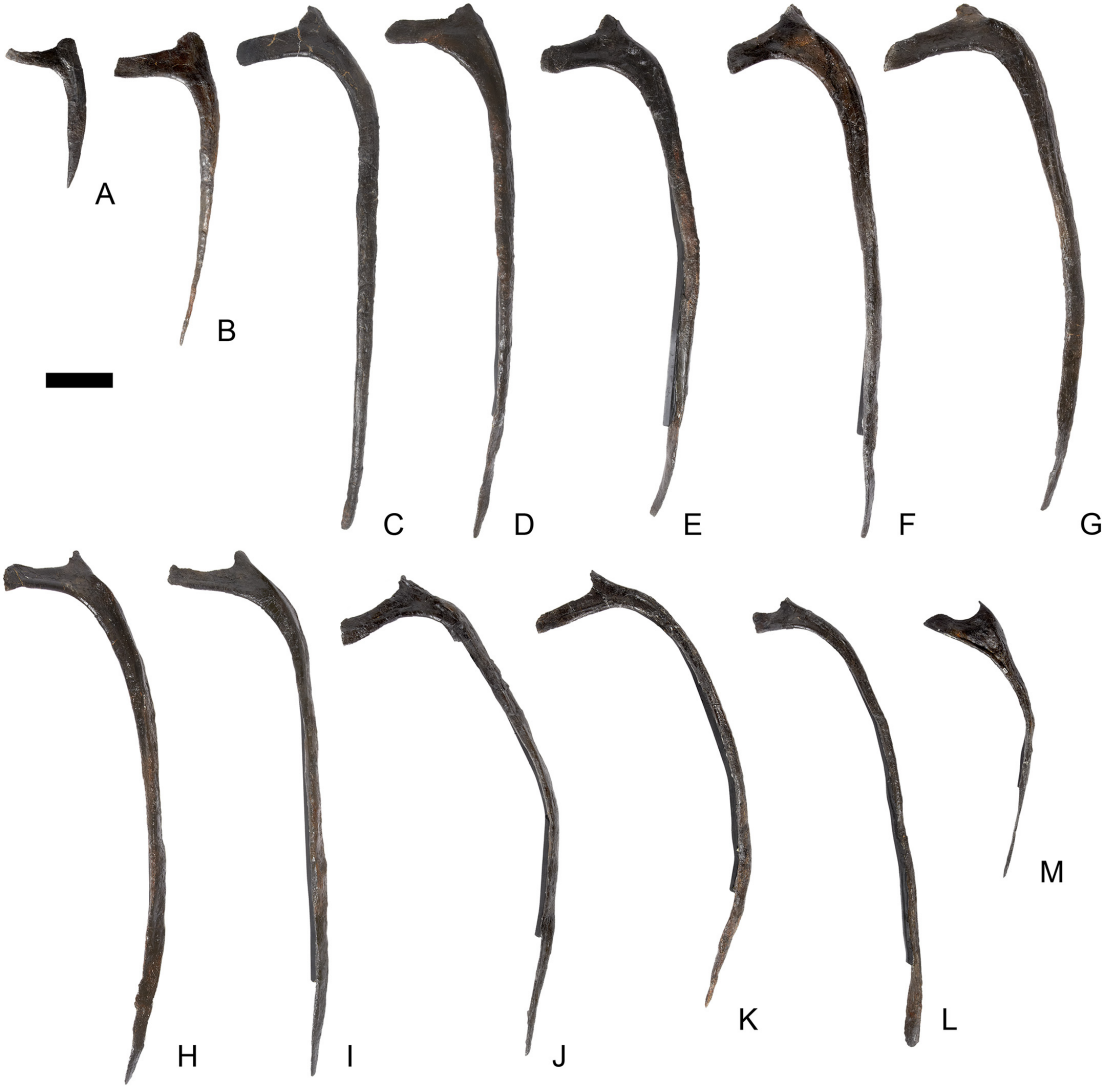
Left dorsal ribs in lateral view. **A**, 1; **B**, 2; **C**, 3; **D**, 4; **E**, 5; **F**, 6; **G**, 7; **H**, 8; **I**, 9; **J**, 10; **K**, 11; **L**, 12; **M**, 13. Scale bar equal to 10 cm.

**Fig 64:**
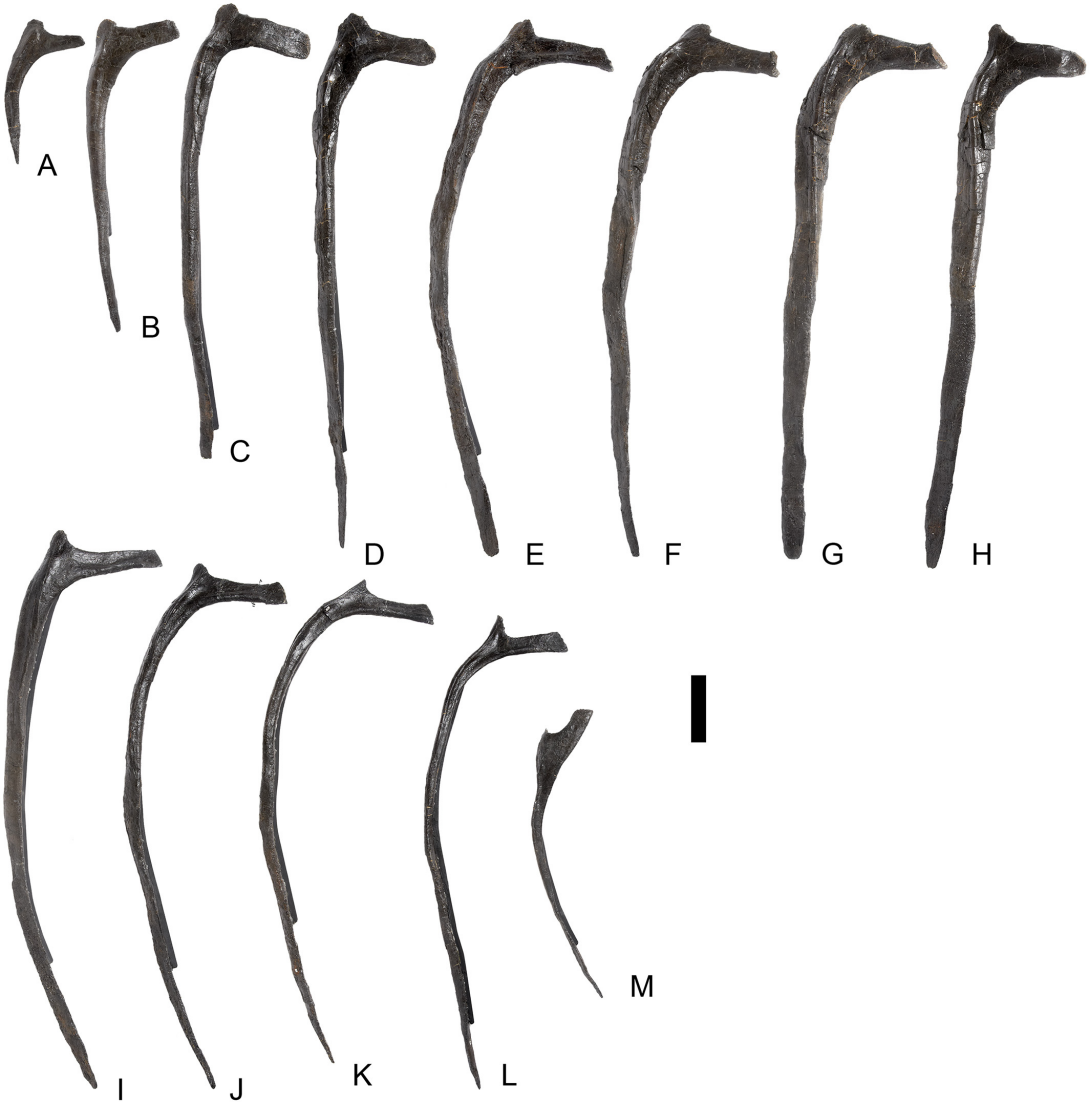
Right dorsal ribs in lateral view. **A**, 1; **B**, 2; **C**, 3; **D**, 4; **E**, 5; **F**, 6; **G**, 7; **H**, 8; **I**, 9; **J**, 10; **K**, 11; **L**, 12; **M**, 13. Scale bar equal to 10 cm.

**Table 2:** Measurements of ribs in mm. All measurements are maxima.

Element	Side	Total Length (mm)	Length of capitulum (mm)	Length of tuberculum (mm)
Atlas rib	Left	71		
	Right	67		
Axis rib	Right	56		
Cervical rib 3	Left	60		
	Right	60		
Cervical rib 4	Left	70		
	Right	66		
Cervical rib 5	Left	62		
Cervical rib 6	Left	71		
Cervical rib 7	Left	86		
Cervical rib 8	Left	76		
Cervical rib 10	Left	86		
Cervical rib 13	Left	120		
	Right	129		
Dorsal rib 1	Right	230	68	16
Dorsal rib 2	Right	480	98	13
Dorsal rib 3	Left	830	122	22
	Right	690	121	13
Dorsal rib 4	Left	830	108	18
	Right	830	122	24
Dorsal rib 5	Right	815	115	24
Dorsal rib 6	Left	830		22
	Right	825	141	21
Dorsal rib 7	Left	820	148	22
	Right	820	132	17
Dorsal rib 8	Left	830	112	25
	Right	835	141	18
Dorsal rib 9	Left	840	114	16
	Right	848	134	15
Dorsal rib 10	Left	798	123	
	Right	814	128	
Dorsal rib 11	Left	764	131	21
	Right	782		
Dorsal rib 12	Left	735		
	Right	740	340	

Progressing along the cervical column, the shafts of the cervical ribs reduce in length proportionately, while the tuberculum becomes increasingly elongate. The dorsal ribs increase in total length posteriorly from dorsal rib 1 to dorsal 3, whereupon they maintain approximately the same proportions until dorsal rib 13, which has a much reduced shaft relative to those anterior to it. The tuberculum also increases in prominence progressing posteriorly along the dorsal vertebral column.

#### Cervical rib 1

The ribs of the atlas are both preserved, although part of the anterior margin of the left rib is missing and the very distal parts of the shafts are broken in both specimens. The ribs are transversely compressed, short, delicate elements that taper to a blunt point posteriorly. The rib shaft curves gently posterodorsally along its length. The anterior end is complete on the right rib and is convex anteriorly. It is single-headed, lacking a distinct capitulum and tuberculum, but bears two transversely thickened areas anteriorly in their approximate positions: one anterodorsally and one anteroventrally. These thickened areas are roughened, particularly medially, but it is not clear if only one or both of these articulated with the atlas. The lateral surface of the rib is shallowly excavated, with this excavation bounded ventrally by a sharp lip of bone. The medial surface is gently convex dorsoventrally.

#### Cervical rib 2

Parts of both axis ribs are present, but the on the right the anterodorsal region and on the left the anterior part are reconstructed. The preserved portion of the right rib is triangular in lateral view. The lateral surface is gently concave, bounded by a prominent ridge ventrally. It tapers to a point posteriorly. The rib shaft is straight. In medial view the surface of the rib is gently convex. Although reconstructed as double-headed, this cannot be confirmed as these areas are reconstructed on both ribs.

#### Cervical rib 3

Both ribs are complete. They are double-headed and triangular in lateral view. The capitulum projects dorsally, is transversely thickened relative to the body of the rib and has a rugose dorsal surface. In dorsal view the facet on the capitulum is oval in outline with the long axis trending anteroposteriorly. The tuberculum projects anteriorly and is not transversely thickened, but maintains the same thickness as the shaft. A thin web of bone connects the anterior margin of the capitulum with the tuberculum. The rib shaft is gently upwardly concave along its length and tapers to a point. The lateral surface is gently concave along its length. A ridge extends from the tuberculum posterodorsally for a short distance towards the shaft of the rib and a second ridge arises ventral to the first ridge and extends posteriorly along the ventral margin of the shaft prior to migrating on to its lateral surface. In medial view, the shaft is flat and there are rugosities around the capitulum.

#### Cervical rib 4

Both ribs are complete. They are ‘Y’-shaped in lateral view and double-headed. The shaft is upwardly concave, transversely compressed and tapers posteriorly. The capitulum projects dorsally, is transversely compressed and is not thickened transversely relative to the body of the rib, in contrast to the cervical rib (Cr) 3. In lateral view, the capitulum has a rectangular outline, while the tuberculum is blunt-ended, but triangular in transverse section with the apex pointing laterally. The capitulum is much larger than the tuberculum, being about twice as wide anteroposteriorly as the tuberculum is dorsoventrally. A 90 degree angle separates the tuberculum and capitulum in lateral view. The notch between the tuberculum and capitulum is gently concave, with reduced web of bone between them. A ridge extends from the lateral surface of the tuberculum posteriorly to the end of the shaft and has a rugose thickening at a point level with the capitulum. This ridge gives the shaft an ‘L’-shaped cross-section. The medial surface of the rib shaft is flat to slightly concave.

#### Cervical rib 5

The left Cr5 is relatively well preserved but the right rib has a largely reconstructed distal shaft and capitulum. The ribs are essentially identical to Cr4 except that the ventral thickening below the capitulum is reduced in prominence. The cross section of the tuberculum is slightly kidney-bean shaped rather than triangular.

#### Cervical rib 6

The left Cr6 is mainly complete but the right was not preserved. A very small part of the distal end of the left is missing. It is similar to Cr4–5. The only significant difference is that the capitulum is slightly anterodorsally inclined. The ridge extending along the rib shaft is more prominent than in the preceding ribs, which probably reflects the greater size of the rib. As a result of the increasing prominence of the ridge there is a trough between the ridge and the rib shaft. The rib is similar to that illustrated by ([2]: fig 31) for USNM 4934 (*S*. *stenops*).

#### Cervical rib 7

The left Cr7 is present although the right was not preserved. The rib differs in morphology from the preceding ribs in that both the capitulum and tuberculum are substantially elongated and, unlike in the preceding ribs, where the capitulum and tuberculum were sub-equal in length, the tuberculum is now longer than the capitulum. An angle of just under 90 degrees separates the capitulum and tuberculum in lateral view. The capitulum is slightly anteriorly inclined. The tuberculum is sub-equal in length to the rib shaft, and it does not taper anteriorly but terminates in a square-ended articular facet that has a narrow, kidney bean-shaped outline in anterior view. A thick ridge extends from the tuberculum posteriorly along the ventral margin of the rib; this ridge merges with the rib shaft at a point where the shaft meets the capitulum. The prominent thickening level with the capitulum observed in preceding vertebrae is absent and the ridge does not enclose a trough, in contrast to the condition in Cr6. The rib shaft does not curve upward distally in contrast to the preceding cervicals, and the ventral margin of the entire rib describes a gentle sinusoidal curve. The distal rib shaft has a sub-triangular cross-section.

#### Cervical rib 8

Both Cr8 are partially preserved, although the left is better preserved than the right. Portions of the shaft are reconstructed on both, and the tuberculum of the right rib is also reconstructed. Determining the extent of the reconstructed areas is difficult. The genuine parts of the left rib are very similar to the morphology of Cr6 in that the tuberculum and capitulum are of sub-equal length; a prominent ridge extends along the entire ventral margin of the rib bounding a shallow trough; a thickening of the ventral margin is present ventral to the capitulum, and the capitulum is slightly anteriorly inclined. It differs from Cr6 but is similar to Cr7 in having a straight ventral margin. There is also a prominent ridge extending posteroventrally across the lateral surface of the capitulum, although this ridge has probably been accentuated by damage. This ridge forms the dorsal margin of a shallow excavation that covers most of the capitulum and the central part of the rib. In medial view a ridge extends ventrally from capitulum a short distance.

#### Cervical rib 9

The left Cr9 is missing, while the right has been heavily restored with only small parts of the capitulum that are genuine. Details of the anatomy cannot therefore be distinguished.

#### Cervical rib 10

The right Cr10 has a reconstructed tuberculum and shaft; only the capitulum and central body of the rib are complete. On the left side, the rib has a badly damaged capitulum, and the proximal ends of both tuberculum and capitulum are reconstructed. The capitulum is a finger-like process that extends anterodorsally. A posteroventrally extending ridge and a shallow excavation ventral to it are present on the lateral surface of the capitulum on the left side, as in cervical rib eight. The ventral surface of the rib shaft is transversely thickened but in contrast to preceding ribs, a ridge in this region is not present. The distal rib shaft tapers to a point. The ventral margin of the rib describes a gentle sinusoidal curve.

#### Cervical rib 11

The capitulum and tuberculum of the right Cr11 are slightly broken at their ends, and while the rib shaft has undergone some restoration, the central part of the rib seems to be real. Only the ventral part of the left rib is preserved, including the entire tuberculum and part of the distal shaft. The capitulum is entirely reconstructed. The ribs appear to be identical in morphology to Cr10.

#### Cervical rib 12

The right Cr12 is poorly preserved but the capitulum, part of the tuberculum and part of the shaft are preserved. The left rib is not preserved. The rib differs from the preceding ribs only in that the capitulum is thickened transversely and exceeds the distal shaft in length.

#### Cervical rib 13

Both ribs are real although the left is deformed such that its tuberculum has been rotated to project anterolaterally rather than anteriorly. In lateral view the tuberculum is the longest process of the rib. It has a sub-elliptical cross-section with the long axis of the ellipse trending vertically. It does not taper and ends in a blunt square anteriorly. It is separated from the capitulum by an angle of around 80 degrees due to the anterior inclination of the capitulum. The ventral margin of the tuberculum and shaft are transversely thickened relative to the rest of the element. Ridges and grooves observed on the shafts of preceding ribs are absent. The prominent ridge on the lateral surface of the capitulum is present and a similar ridge is present in the same position on the medial surface. The medial surface of the rib is flat.

#### Dorsal rib 1

The right rib of dorsal one (Dr1) is almost completely preserved, but on the left side, only the capitulum and a small portion of the proximal shaft are real. The rib is gently curved ventromedially along its entire length. The rib shaft is anteroposteriorly compressed proximally, but expands in its distal part to produce an oval transverse cross-section. The shaft tapers to a point distally. In anterior view, the capitulum forms an angle of 140 degrees with the proximal part of the rib shaft. The dorsal margin of the capitulum is anteroposteriorly compressed to form a thin flange, but it expands ventrally to form a thickened ventral margin, giving it a teardrop-shaped parasagittal cross-section. The articular surface of the capitulum is slightly rugose, but unexpanded anteroposteriorly. The surface linking the capitulum and tuberculum is concave and faces slightly dorsally in anterior view. An angle of 90 degrees separates the capitulum and tuberculum in anterior view. The tuberculum is poorly developed, much shorter than the capitulum, and is also anteroposteriorly compressed. The articular surface of the tuberculum is rugose and slightly expanded anteroposteriorly with respect to the rest of the tuberculum. The tuberculum articular surface is broader transversely than that of the capitulum. The posterior surface of the rib head is gently concave, whereas the anterior surface is gently convex. In anterior view, ventral to the tuberculum and level with the dorsal margin of the capitulum is a protuberance, which is oval with its long axis trending dorsoventrally. The ventral margin of this protuberance continues into a ridge that extends ventrally for approximately half of the length of the rib shaft before merging with its lateral margin. The area medial to this ridge is concave. The dorsal ribs of *Huayangosaurus* differ from those of *Stegosaurus* as they possess a flange at midshaft that extends posteriorly to make contact with the subsequent rib ([31]: fig 19): moreover, in *Huayangosaurus* and *Jiangjunosaurus* there are small, crescentic flanges near the distal ends of the ribs [28] that are absent in *Stegosaurus*.

#### Dorsal rib 2

The right Dr2 is reconstructed at its distal end, while the shaft of the left rib has been heavily restored and reconstructed distally. In anterior view, the rib shaft is straight and it tapers distally. Proximally, the shaft is anteroposteriorly compressed and distally it expands anteroposteriorly so that it has an oval cross-section with its long axis pointing anteroposteriorly, as in Dr1. The capitulum projects at an angle of 130 degrees with respect to the rib shaft in anterior view. The capitulum has a rectangular outline in anterior view. It is anteroposteriorly compressed along its entire length and its articular surface is unexpanded but slightly rugose, as in Dr1. The tuberculum is a small process on the dorsolateral margin of the capitulum. It does not have a distinct shaft. In anterior view, it is dorsally convex, while in posterior view the articular surface is confined to the posterior surface, angled posteroventrally and is oval in shape, with the long axis trending transversely. The articular surface is slightly concave and rugose. A low swelling is present on the anterior surface of the shaft level with the ventral margin of the capitulum and this extends ventrally as a ridge that merges with the shaft approximately one-quarter of the way along its length. The posterior surface of the rib head is shallowly concave, as in Dr1.

#### Dorsal rib 3

Both Dr3 are completely preserved, but the left rib has been slightly deformed, resulting in greater curvature. In most respects, Dr3 is similar to Dr2, but larger and differs in the following respects. The capitulum lies at 90 degrees to the shaft in anterior view, a more acute angle than in preceding dorsal ribs. The articular facet of the tuberculum is proportionally larger and faces more dorsally. The ridge on the anterior surface of the shaft extends ventromedially to merge with the anterior margin of the shaft around one-third of the way along its length. The concavity on the posterior surface of the rib head is more pronounced due to greater curvature of the lateral margin of the shaft posteriorly, and this concavity extends along the posterior surface of the proximal part of the rib shaft.

#### Dorsal rib 4

Both Dr4 are essentially complete. They are very similar to Dr3, except that the distal quarter of the rib shaft is transversely compressed to form a thin blade. The ridge on the anterior surface of the rib is more prominent, forming a low anteriorly-projecting flange.

#### Dorsal rib 5

The right Dr5 is complete, while the left is complete proximally but reconstructed along the shaft distally. The ribs are similar to those of Dr4 except in the following respects. The anterior ridge is more prominent and forms the boundary between a medial concavity on the proximal part of the anterior surface of the shaft and a convex posterolateral surface. Increased posterior curvature of the shaft lateral margin produces an even deeper posterior concavity on the proximal shaft than observed in Dr4. The articular surface of the capitulum is obliquely inclined to face dorsomedially, so that in anterior view the capitulum tapers, rather than being square-ended.

#### Dorsal rib 6

The right Dr6 is complete although the distal end has been restored, whereas the left is poorly preserved, and the capitulum and distal part of the shaft are reconstructed. The ribs are similar to Dr5 except in the following respects. The articular surface of the capitulum is obliquely inclined and anteroposteriorly expanded relative to the rest of the process. It is oval in outline in medial view. The anterior ridge is prominent, located anterolaterally and crosses the shaft to merge with the medial margin about one-third of the way ventrally in anterior view; proximally it defines a medial concavity and a lateral convex surface, as it does in Dr5. The increase in prominence of the ridge and the posterior curvature of the lateral shaft produces a T-shaped cross-section proximally, which becomes ovate where the ridge merges with the shaft, and then blade-like more distally. The articular facet of the tuberculum is anteroposteriorly narrower than in Dr4 and 5.

#### Dorsal rib 7

Distal parts of the shafts of both Dr7 are reconstructed but they are otherwise well preserved and similar to Dr6. The only substantive difference between Dr7 and Dr6 is increased length and prominence of the anterior ridge in the former, which now forms a very distinct anteriorly projecting flange of bone that extends approximately halfway along the shaft. The ridge is slightly curved medially along its apex, partially enclosing the deep concavity on the medial part of the proximal shaft in anterior view. The capitulum projects from the shaft at an angle of 120 degrees.

#### Dorsal rib 8

The distal ends of both Dr8 are reconstructed but they are otherwise complete. The ribs are very similar to Dr6 and 7 except that the ventral margin of the capitulum is thickened anteroposteriorly and the anterior and posterior surfaces of the process are convex. The articular surface of the capitulum is not anteroposteriorly expanded relative to the rest of the process.

#### Dorsal rib 9

Both Dr9 are essentially complete and are almost identical to Dr8. The only minor difference is that the rib ventral to the point where the anterior ridge merges with the shaft is transversely compressed and blade-like.

#### Dorsal rib 10

Both Dr10 are complete, well preserved and essentially identical to those preceding them. The ribs of dorsal ten appear slightly more curved along their length than those of dorsal nine.

#### Dorsal rib 11

The left Dr11 is essentially complete; on the right the tuberculum and capitulum are reconstructed. The rib is similar to Dr10 and is more curved along its length than Dr9. In comparison with Dr10, the whole rib is more transversely compressed so that ventral to the head the shaft is the same width along its length rather than tapering in anterior view. The tuberculum is a more distinct finger-like process with a clear concavity separating it from the dorsal margin of the capitulum, a condition that is similar to that of Dr1. The capitulum is transversely shorter than in preceding ribs. The ridge on the ventral surface of the capitulum is present but stops short of the articular surface. The anterior ridge extends dorsal to the capitulum towards the anterior surface of the tuberculum, in contrast with the condition in previous ribs, where it terminates level with the capitulum.

#### Dorsal rib 12

The tip of the tuberculum is reconstructed on the right Dr12, while on the left, the head is reconstructed. As with many of the ribs, the distal ends have been restored. The ribs are similar to Dr11 but are more gracile. The ridge on the anterior surface is reduced in length, only extending for one-quarter of the length of the shaft, but dorsally extends to the tuberculum. The tuberculum forms a distinct finger-like process whose articular surface is elliptical and faces medially. The ridge on the ventral surface of the capitulum is less pronounced than in Dr11.

#### Dorsal rib 13

Only the shafts of both Dr13 are preserved, and on the left side the distal end is also reconstructed. The ribs were probably significantly shorter than the preceding ribs, but otherwise appear similar to Dr12.

### Chevrons (Fig 65)

Only two chevrons are preserved, and are associated with the distal part of the tail. The more anteriorly-situated of the two is mounted with Cd27. It is Y-shaped in anterior view with a complete bridge of bone enclosing the proximal end. [2] noted that fusion of the articular surfaces of the chevrons to each other was variable from chevron to chevron in USNM 4934 (*S*. *stenops*). The haemal canal is narrow, elongate and elliptical in outline both anteriorly and posteriorly and accounts for approximately one-third of the total length of the chevron. In proximal view the articular surface is gently convex anteroposteriorly and has a dumbbell-shaped outline. In anterior view the ventral part of the chevron tapers ventrally but is incomplete distally. Ventral to the haemal canal a strong midline ridge extends ventrally dividing the anterior surface of the chevron into two separate laterally facing surfaces and giving this part of the chevron blade a sub-triangular cross-section. In lateral view the distal end of the chevron is curved posteriorly. In posterior view the posterior surface of the chevron blade is flat. The more posterior chevron, which is mounted with Cd29, is complete apart from the left side of the proximal end. The haemal canal is relatively larger, accounting for half of the length of the chevron. In most respects it is identical to the other chevron, except that the chevron blade ventral to the haemal canal is more strongly compressed transversely.

**Fig 65:**
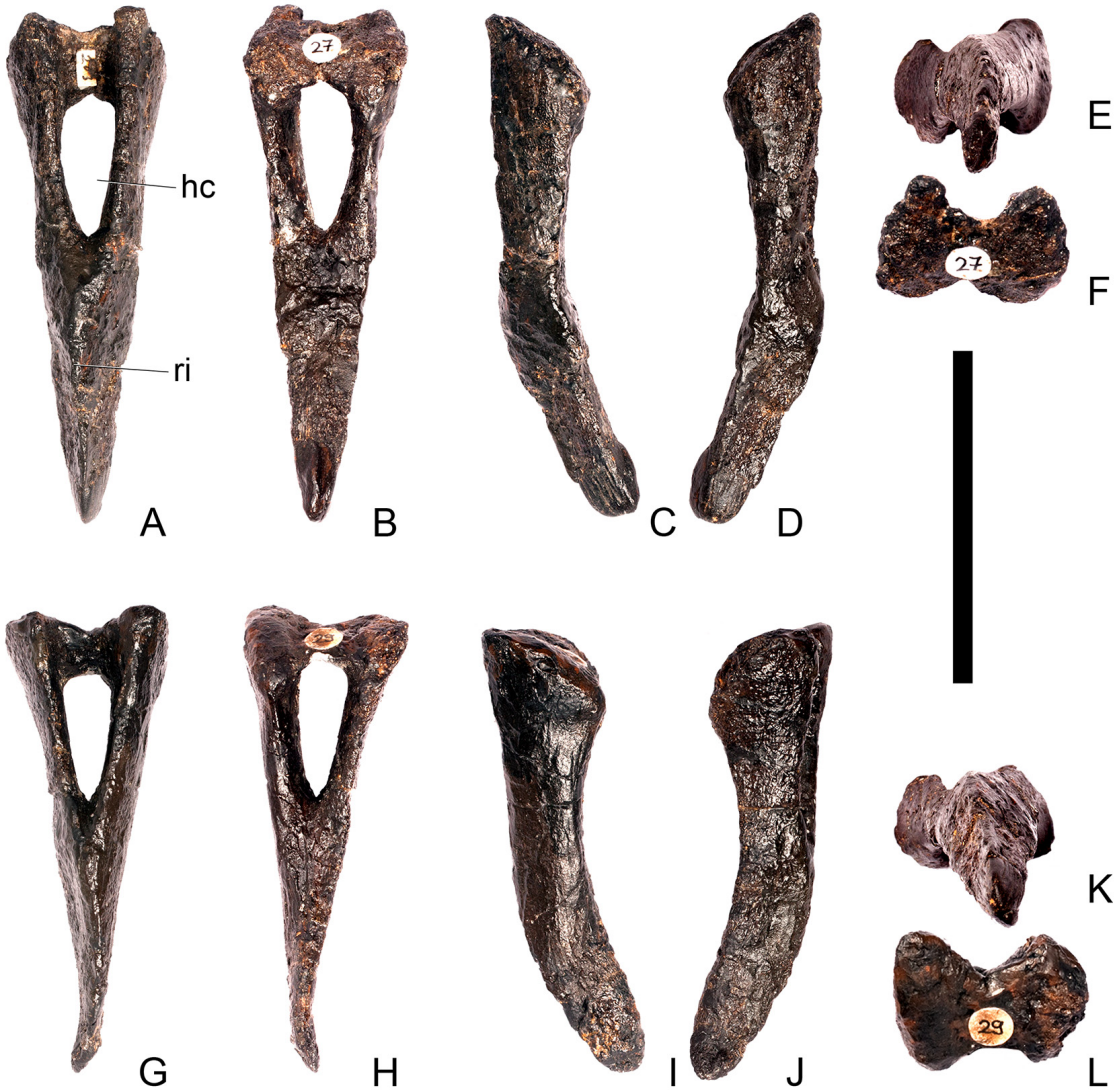
Chevrons. **A-F**, chevron 27 and **G-L**, chevron 29 in **A, G,** anterior; **B, H**, posterior; **C, I**, left lateral; **D, J**, right lateral; **E, K**, ventral and **F, L**, dorsal views. **Hc**, haemal canal; **ri**, ridge. Scale bar equal to 5 cm.

### Pectoral girdle and forelimb

[2]: fig 32 illustrated an element he identified as a left sternal from *Stegosaurus* (USNM 7620), however no such elements were recovered with either NHMUK PV R36730 or USNM 4934, the two most complete specimens of *Stegosaurus* known. Given the completeness of NHMUK PV R36730 and the fact that almost complete pectoral girdles are preserved, if ossified sterna were present in *Stegosaurus* it seems highly likely that they would have been recovered. The lack of sternal elements in NHMUK PV R36730 and USNM 4934 leads us to conclude that ossified sternals were absent in *Stegosaurus* and that the element illustrated by [2]: fig 32 pertains to a different genus. Based on its morphology and size, it is possible that the latter is an atlantal rib of a sauropod. Measurements of all girdle and limb elements can be found in Table 3.

**Table 3:** Measurements of girdle and limb elements in mm. All measurements are maxima.

Element	Measurement	Dimension (mm)
**Scapula, left**	Total length	643
**Scapula, left**	Dorsoventral height of proximal plate	331
**Scapula, left**	Dorsoventral height of distal blade	178
**Scapula, left**	Minimum dorsoventral blade height	122
**Scapula, left**	Dorsoventral height of glenoid fossa	127
**Scapula, left**	Transverse width of glenoid fossa	67
**Coracoid, left**	Maximum dorsoventral height	205
**Coracoid, left**	Maximum anteroposterior length	205
**Coracoid, left**	Anteroposterior length of glenoid fossa	90
**Coracoid, left**	Transverse width of glenoid fossa	65
**Humerus, right**	Total length	450
**Humerus, right**	Maximum width, proximal end	305
**Humerus, right**	Minimum shaft circumference	280
**Humerus, right**	Minimum shaft width	101
**Humerus, right**	Maximum width of distal end	160
**Humerus, right**	Length of deltopectoral crest	260
**Ulna, right**	Total length	412
**Ulna, right**	Midlength shaft width	65
**Ulna, right**	Minimum shaft circumference	180
**Ulna, right**	Transverse width, distal end	51
**Ulna, right**	Transverse width, proximal end	156
**Radius, right**	Total length	312
**Radius, right**	Minimum shaft width	56
**Radius, right**	Minimum shaft circumference	159
**Radius, right**	Transverse width, distal end	112
**Radius, right**	Transverse width, proximal end	104
**Ilium, right**	Total length	860
**Ilium, right**	Maximum width	510
**Ilium, right**	Pracetabular process length	465
**Ischium, right**	Total length	450
**Ischium, right**	Shaft height posterior to proximal plate	90
**Ischium, right**	Anteroposterior length, iliac process	104
**Ischium, right**	Dorsoventral height, pubic process	56
**Pubis, left**	Total length	783
**Pubis, left**	Minimum dorsoventral postpubis width	41
**Pubis, right**	Total length	791
**Pubis, right**	Prepubis length (from iliac peduncle)	276
**Pubis, right**	Postpubis length (to obturator notch)	485
**Pubis, right**	Dorsoventral height, anterior end of prepubis	53
**Pubis, right**	Dorsoventral height of body above obturator notch	80
**Femur, left**	Total length	857
**Femur, left**	Maximum with, proximal end	252
**Femur, left**	Maximum width, distal end	240
**Femur, left**	Midshaft circumference	337
**Femur, left**	Midshaft width	123
**Femur, right**	Total length	868
**Femur, right**	Maximum with, proximal end	258
**Femur, right**	Maximum width, distal end	231
**Femur, right**	Midshaft circumference	347
**Femur, right**	Midshaft width	127
**Tibia, left**	Total length	495
**Tibia, left**	Midshaft circumference	248
**Tibia, left**	Midshaft width	87
**Tibia, left**	Transverse width, distal end	200
**Tibia, left**	Anteroposterior width, proximal end	245
**Tibia, right**	Total length	498
**Tibia, right**	Midshaft circumference	260
**Tibia, right**	Midshaft width	84
**Tibia, right**	Transverse width, distal end	125
**Tibia, right**	Anteroposterior width, proximal end	250
**Fibula, left**	Total length	467
**Fibula, left**	Maximum width, proximal end	99
**Fibula, left**	Maximum width, distal end	86
**Fibula, left**	Midshaft width	50
**Fibula, left**	Midshaft circumference	130
**Fibula, right**	Total length	467
**Fibula, right**	Maximum width, proximal end	72
**Fibula, right**	Maximum width, distal end	75
**Fibula, right**	Midshaft width	34
**Fibula, right**	Midshaft circumference	122
**Astragalus, right**	Transverse width	108 (as preserved)
**Astragalus, right**	Anteroposterior length	79
**Calcaneum, left**	Anteroposterior length	78
**Calcaneum, left**	Transverse width	56
**Calcaneum, left**	Maximum dorsoventral height	32
**Metatarsal 2, left**	Total length	72
**Metatarsal 2, left**	Anteroposterior length of dorsal margin	82
**Metatarsal 2, left**	Anteroposterior length of ventral margin	55
**Metatarsal 2, left**	Transverse with of ventral articular surface	47
**Metatarsal 2, left**	Maximum transverse width	47
**Metatarsal 2, right**	Total length	78
**Metatarsal 2, right**	Anteroposterior length of dorsal margin	82
**Metatarsal 2, right**	Anteroposterior length of ventral margin	57
**Metatarsal 2, right**	Transverse with of ventral articular surface	49
**Metatarsal 2, right**	Maximum transverse width	44
**Metatarsal 3, right**	Total length	100
**Metatarsal 3, right**	Anteroposterior length of dorsal margin	76
**Metatarsal 3, right**	Anteroposterior length of ventral margin	53
**Metatarsal 3, right**	Transverse with of ventral articular surface	52
**Metatarsal 3, right**	Maximum transverse width	64
**Metatarsal 4, right**	Total length	79
**Metatarsal 4, right**	Anteroposterior length of dorsal margin	58
**Metatarsal 4, right**	Anteroposterior length of ventral margin	41
**Metatarsal 4, right**	Transverse with of ventral articular surface	56
**Metatarsal 4, right**	Maximum transverse width	73
**Ungual 1**	Total length	73
**Ungual 1**	Transverse width, articular surface	49
**Ungual 2**	Total length	68
**Ungual 2**	Transverse width, articular surface	50

#### Scapula (Fig 66)

The left scapula is almost complete but a small portion of the proximal plate has been reconstructed dorsally in the region of the acromial ridge. The right scapula is missing the posterodorsal part of the proximal plate. The area around the glenoid and coracoid articulation and small sections of the ventral margin of the scapula shaft are partially reconstructed.

**Fig 66:**
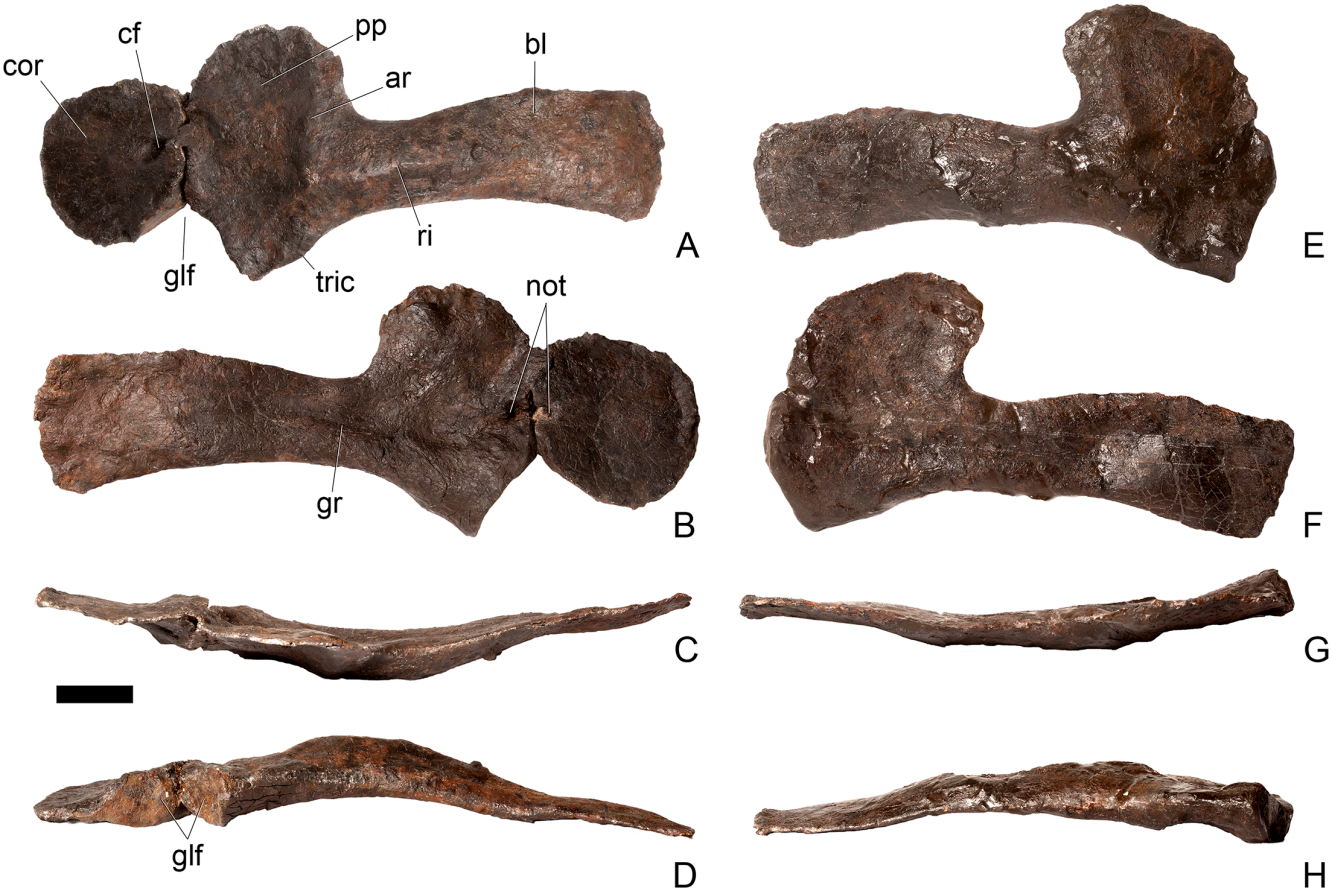
Scapula and coracoid. **A-D**, left scapulocoracoid and **E-H**, right scapula in **A, E**, lateral, **B, F**, medial, **C, G**, dorsal and **D, H,** ventral views. **Ar**, acromial ridge; **bl**, scapula blade; **cf**, coracoid foramen; **cor**, coracoid; **glf**, glenoid fossa; **gr**, groove; **pp**, scapula proximal plate; **ri**, ridge; **tric**, triceps attachment. Scale bar equal to 10 cm.

In lateral view the scapula consists of a proximal plate that is sub-rectangular in outline with the long axis aligned dorsoventrally, and a robust, elongate blade (Fig 66A, 66B, 66E and 66F). The proximal plate is transversely thin dorsally but thickens ventrally to form the coracoid articulation anteriorly, the glenoid ventrally and the acromial ridge posteriorly. In lateral view the anterior part of the proximal plate is gently concave but this becomes convex along the acromial ridge, which extends dorsoventrally close to the posterior margin of the proximal plate (Fig 66A and 66E). It is slightly eroded dorsally. This ridge is better developed in *Kentrosaurus* (MB R.4802; R.4803; [33]: figs. 39–41). The anterior margin of the proximal plate is straight; whereas its anteroventral margin is gently concave, forming the scapula contribution to the glenoid fossa. The glenoid faces anteroventrally and is elliptical in anterior view with the long axis of this ellipse extending anterodorsally (Fig 66A and 66E). A small tubercle on the ventrolateral margin of the proximal plate represents the origin for the triceps. In medial view, the dorsal and central parts of the proximal plate are flat to gently concave whereas the ventral part is strongly convex. A distinct notch is present on the anterior margin of the medial surface. This notch is continuous with that from the coracoid foramen (Fig 66B and 66D). The scapula and coracoid are partially fused but the line dividing them is still clearly visible (Fig 66A–66D. Due to this partial fusion the shape of the articular surface for the coracoid cannot be determined. [2] noted that the fusion of coracoids and scapulae varied among *Stegosaurus* individuals and was likely ontogenetic; it also appears to be variable in *Kentrosaurus* (fused in MB R.4802 and R.4803, unfused in R.4580). At present, there are too few individuals of *Stegosaurus* from varying ontogenetic stages to examine whether scapula and coracoid fusion is ontogenetic or due to individual variation.

The scapula blade forms an angle of approximately 90 degrees with the posterior margin of the proximal plate and emerges from the lower half of it (Fig 66A, 66B, 66E and 66F). In contrast, the angle between the proximal plate and scapula blade in *Huayangosaurus* (ZDM T7001; [31]: fig 23) is greater than 90 degrees, and the proximal plate is posterodorsally rounded. The lateral surface of the proximal scapula blade is strongly convex such that it is divided into dorsolaterally and ventrolaterally facing surfaces by a low ridge that extends along the centre of the blade (Fig 66A). This ridge merges into the blade approximately halfway along its length. The remainder of the lateral surface is very gently convex dorsoventrally and forms a single surface. The distal end of the blade is slightly eroded but it appears to have been square-ended. The dorsal and ventral margins of the blade are sub-parallel along their entire lengths with the dorsal margin slightly convex and the ventral margin slightly concave. This differs from the condition in *Gigantspinosaurus* ([27]: fig 133) where the dorsal margin of the scapula blade dorsoventrally widens abruptly about one-quarter of the way from its anterior end, this widened portion forming a right-angle with the anterior portion of the blade. In dorsal view the scapula blade is bowed laterally to accommodate the rib cage (Fig 66C and 66G). In medial view, the ventral convexity of the proximal plate extends for a short distance along the ventral margin of the blade, helping to define a shallow groove that extends along the medial surface of the blade to a point approximately one-third along its length. The remainder of the medial surface of the blade is flat or very gently concave.

#### Coracoid (Fig 66)

Only the left coracoid is preserved. In lateral view, the coracoid has a sub-circular outline (Fig 66A). This contrasts with the condition in *Kentrosaurus* (MB R.4802; R.4803; [33]: figs. 39–41) where the coracoid is anteroposteriorly longer than it is dorsoventrally high. The dorsal, anterior, ventral and posterior margins merge into each other without distinct corners or breaks of slope. The lateral surface is gently concave. Anterodorsally, there is a small transverse expansion that is asymmetrical, being largely directed medially. This expansion is probably the attachment area for the biceps. In medial view the surface is gently concave; its posterior margin is indented by a deep notch that is continuous with that on the proximal scapula and also with the laterally positioned coracoid foramen (Fig 66B). The coracoid foramen in *Kentrosaurus* (MB R.4802; R.4803; [33]: figs. 39–41) appears to be relatively larger than in NHMUK PV R36730. In lateral view the coracoid foramen is situated in the posterior part of the bone at approximately mid-height. The coracoid foramen is elliptical with the long axis trending anteroposteriorly. The dorsal and anterior margins of the coracoid are transversely thin, but the bone expands in transverse thickness posteroventrally to form the coracoid contribution to the glenoid fossa. In ventral view the glenoid is ovate in outline, being broadest posteriorly and tapering anteroventrally (Fig 66D). The glenoid is very gently concave anteroposteriorly and is about two-thirds the length of the scapula contribution to the glenoid. The articular surface for the scapula faces entirely posteriorly; its morphology is obscured by partial fusion with the scapula. In all respects the morphology of the coracoid is extremely similar to that of *Dacentrurus* sp. (ML 433).

### Humerus (Fig 67)

The right humerus is almost complete, except for the proximomedial corner, which is damaged and a least partially reconstructed, so that the morphology of the medial tubercle and humeral head cannot be determined with confidence. The left humerus is not preserved.

**Fig 67:**
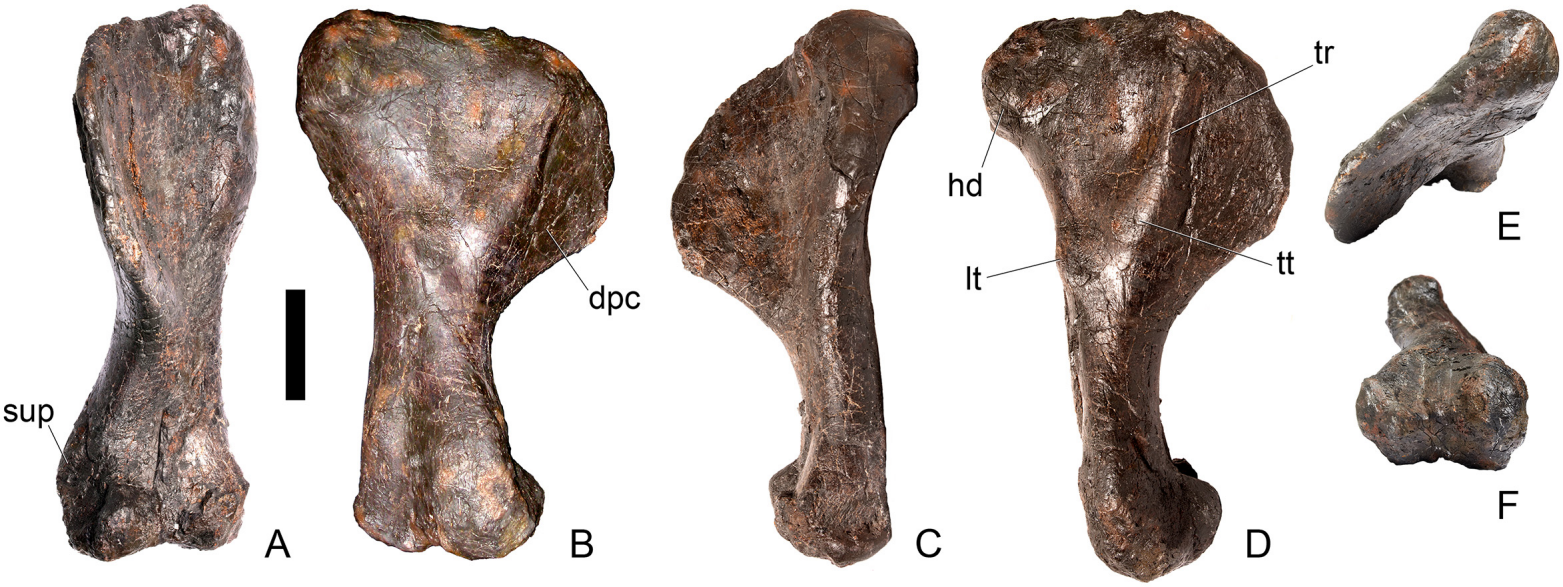
Right humerus. **A**, anterior, **B,** posterior, **C**, medial, **D**, lateral, **E**, dorsal and **F**, ventral views. **Dpc**, deltopectoral crest; **hd**, humeral head; **lt**, latissimus tubercle; **sup**, supinator ridge; **tr**, triceps ridge; **tt**, triceps tubercle. Scale bar equal to 10 cm.

In anterior view, the humerus is dumbbell-shaped with a stout shaft connecting proximal and distal expansions (Fig 67A). The proximal expansion comprises a well-developed deltopectoral crest, with a semi-circular outline (Fig 67B and 67D). The crest projects anterolaterally at an angle of approximately 60 degrees to the anteroposterior axis of the humerus. The crest is a transversely thin flange whose anteromedial surface indistinguishably merges into the anterior surface of the humerus without a distinct break in slope, causing the anterior surface of the proximal humerus to be transversely concave (Fig 67A). The anterolateral margin of the deltopectoral crest has a rugose texture. The crest extends for approximately 60% of humerus length before merging into the lateral surface of the shaft. The medial margin of the proximal end is thickened relative to central portion of the proximal expansion, forming a low ridge that bounds the anterior concavity. The humeral shaft is transversely convex and smooth in anterior view. Distally, it expands transversely to form to the distal expansion that bears the articular condyles. A shallow concavity covers the anterior surface of the distal end (Fig 67A). The distal end is divided into two rugose articular condyles that project anteriorly and which are divided along the midline by a shallow groove that extends onto the distal surface (Fig 67F). The condyles are sub-equal in size, but the medial condyle is slightly eroded. In anterior view, a stout triangular projection, the supinator ridge, extends laterally from the lateral condyle (Fig 67A).

In posterior view, the proximal end is transversely convex below the level of the head, and this area is divided into three distinct surfaces. The humeral head projects posteriorly from the proximomedial corner of the humerus, but is partially reconstructed and its true extent cannot be determined. The medial surface is eroded and has been restored, so the medial tubercle cannot be seen. A flat, narrow, posteriorly-facing medial surface is separated from a larger, posterolaterally-facing and shallowly concave surface by a distinct break in slope, which is continuous with the ventral margin of the humeral head. This larger region comprises most of the proximal end in posterior view and has an inverted triangular outline. In turn, this surface is separated from the lateral surface of the deltopectoral crest by a sharp and well-defined ridge, sometimes known as the descending, or triceps, ridge (Fig 67B and 67D). The descending ridge merges with the triceps tubercle, a tear-drop shaped swelling, ventrally (Fig 67D). The posterior surface of the deltopectoral crest is flat with a semi-circular outline. Its posteroventral surface is rugose. Continuous with and ventral to the break in slope below the humeral head is a second prominent oval tubercle, probably for the M. latissimus dorsi, which is located slightly ventral to the triceps tubercle. The distal one-third of the humerus is slightly crushed, accentuating a concavity present on this area.

In proximal view, the proximal end of the humerus describes a shallow, inverted C-shape due to anterolateral projection of the deltopectoral crest (Fig 67E). The deltopectoral crest is consistently thin along its length in proximal view, except medially, where a spherical anteroposterior expansion defines the poorly preserved humeral head.

In distal view, the humerus is dumbbell-shaped. The lateral and medial condyles form stout diamond shaped expansions separated by a shallow groove forming the waist of the dumb-bell (Fig 67F). The condyles are sub-equal in size; the medial condyle projects slightly further posteriorly than the lateral condyle, although this may have been accentuated by crushing of the posterior surface of the distal humerus. The surfaces of the condyles are slightly rugose, probably due to poor preservation, as there has been some restoration with plaster in this area. The humerus is similar to those of other stegosaurs including *Dacentrurus* sp. (ML 433), *Dacentrurus armatus* (NHMUK OR46013; [29]: fig 9A–E), *Loricatosaurus* (NHMUK PV R3167; MHNH(BR) 001; [29]: fig 18A,B; [32]: fig 2A–D), *Kentrosaurus* (MB R.4804; R.4805; [33]: fig 43) and *Huayangosaurus* (ZDM T7001; [31]: figs 24–25).

#### Ulna (Fig 68)

The right ulna is complete and well preserved although it appears to have been anteroposteriorly crushed and is flattened slightly as a consequence. The left ulna is not preserved.

**Fig 68:**
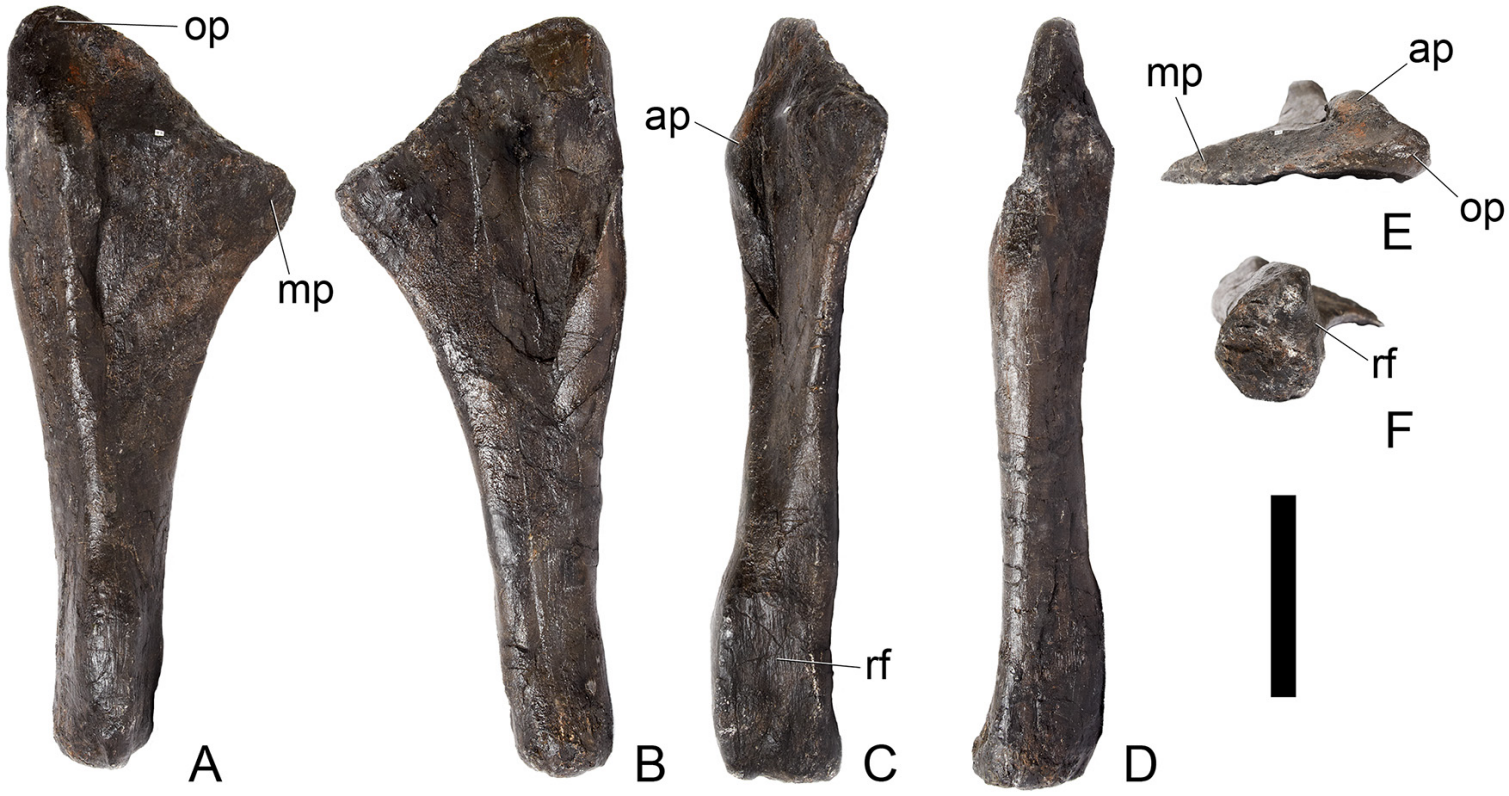
Right ulna. **A**, anterior, **B,** posterior, **C**, medial, **D**, lateral, **E**, dorsal and **F**, ventral views. **Ap**, anterior process; **mp**, medial process; **op**, olecranon process; **rf**, radial facet. Scale bar equal to 10 cm.

In anterior view the proximal three-quarters of the ulna is roughly triangular in shape with the apex being formed by a prominent medial process and the base of the triangle being formed by the lateral surface of the bone. The distal one-quarter of the ulna comprises a parallel-sided shaft (Fig 68A).

In anterior view the proximal ulna comprises three distinct surfaces. At the proximal end a gently concave surface shaped like a right-angled triangle with the hypotenuse facing dorsomedially forms the articular surface of the olecranon process. A second surface, also a right-angled triangle in outline, is gently concave, faces ventromedially, lies ventral to the articular surface of the olecranon process and is separated from it by a gentle, transversely extending ridge. The proximal part of this surface forms the medial process of the ulna, while its distal part merges with the medial surface of the shaft. Its surface is flat and smooth. A third, elongate, narrow and laterally facing surface is separated from the others by a robust dorsoventrally extending ridge that arises slightly ventral to the olecranon process and extends to the distal end of the ulna where it merges with the shaft and increases in robustness and anteroposterior width to become the articular surface for the distal radius. A flat facet for the distal end of the radius is present on the medial surface of this raised ridge. The proximal part of this ridge forms the anterior process of the ulna (Fig 68A). Although it was probably never as prominent as the medial process, the anteroposterior width of this process has probably been reduced by anteroposterior compression and crushing, especially proximally. In anterior view, the lateral surface of the ulna is sinuous, being convex proximally and concave distally. The shaft of the ulna is parallel-sided in anterior view and does not flare distally.

In posterior view, the proximal half of the ulna has been crushed and is partially restored with plaster, so features of its anatomy are obscured. Distally, the shaft of the ulna is sub-rectangular, parallel-sided and flat to gently transversely concave (Fig 68B).

In proximal view, the ulna is ‘L’-shaped, with the medial process forming the downstroke of the ‘L; and the anterior process forming the shorter strut (Fig 68E). In distal view, the articular surface has a sub-triangular outline with the apex pointing anteriorly. The distal end is flat and slightly rugose (Fig 68F). In medial and lateral views the bone is straight (Fig 68C and 68D). The ulna is very similar in morphology to that of *Loricatosaurus* (NMHUK PV R3167; [29]: fig 18E–F, figured upside down). The ulnae of *Dacentrurus* sp. (ML 433) and *Dacentrurus armatus* (NHMUK OR46013; [29]: fig 11A–C) bear a concave, cup-like extension to the olecranon process that is not present in NHMUK PV R36730. In *Kentrosaurus* (MB R.4800; [33]: fig 45) the olecranon process is better developed, projecting further dorsally, than in NHMUK PV R36730, although this may be related to ontogenetic stage [43], or the preservation of articular cartilage [44].

#### Radius (Fig 69)

**Fig 69:**
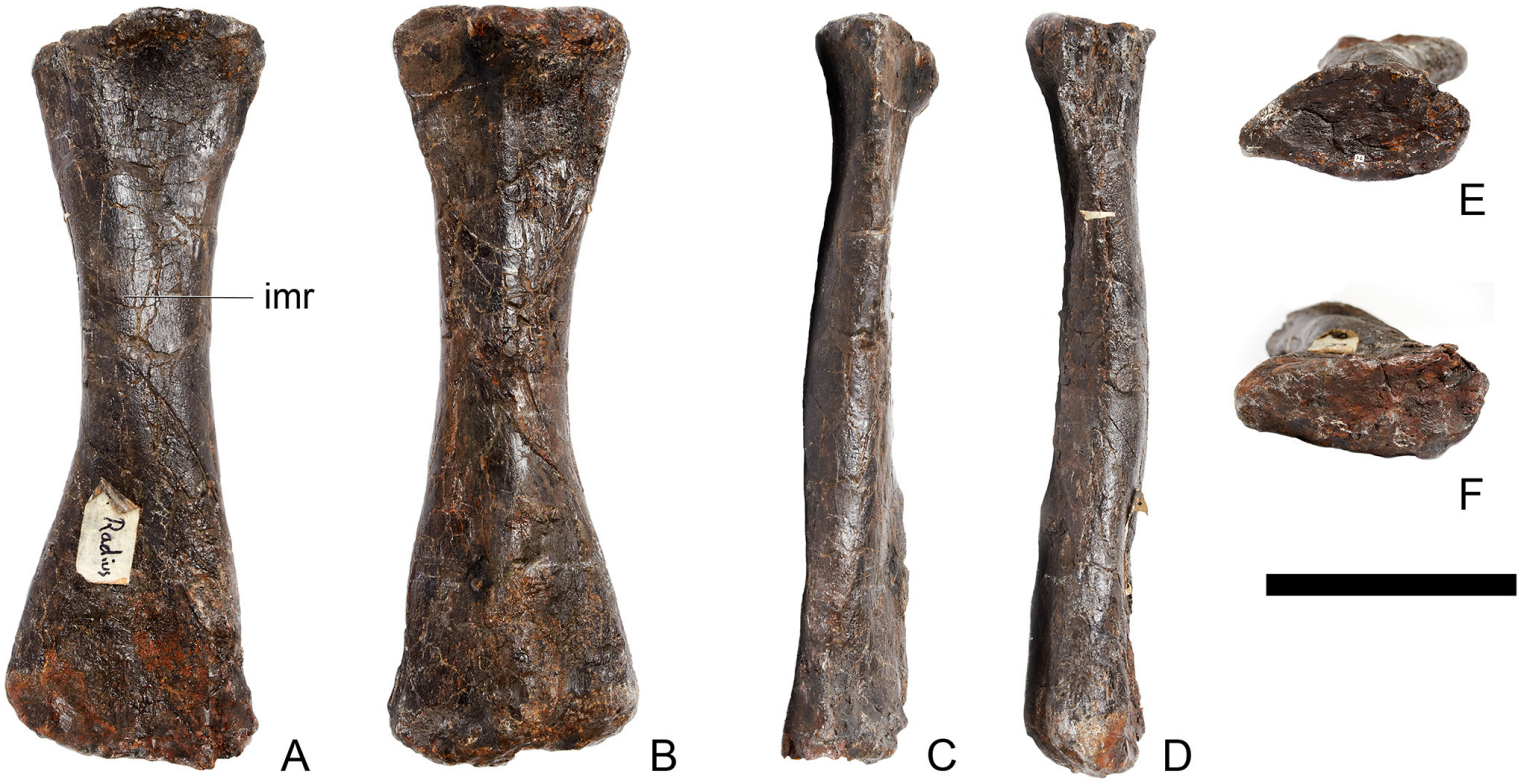
Right radius. **A**, anterior, **B,** posterior, **C**, medial, **D**, lateral, **E**, dorsal and **F**, ventral views. **Imr**, intermuscular ridge. Scale bar equal to 10 cm.

The right radius is well preserved but the left was not recovered. In anterior view, the radius has proximal and distal expansions linked by a narrow shaft (Fig 69A). The distal expansion is slightly wider than the proximal expansion in anterior view and both lie in the same plane. A faint intermuscular ridge extends ventromedially from the proximolateral corner of the anterior surface for approximately three-quarters of the length of the ulna, before merging with the anterior surface of the shaft (Fig 69A). The medial and lateral margins of the bone are concave; the lateral margin is slightly more concave. This is due to the asymmetrical expansion of the distal end, which expands further laterally than it does medially with respect to the shaft long axis. By contrast, the proximal expansion is slightly more expanded medially than laterally. The intramuscular line divides the proximal surface into a convex laterally facing surface and a slightly concave medially facing portion. A shallow fossa extends over the distal part of the anterior surface but has been accentuated by crushing. Proximally the radius has a sub-triangular cross-section that becomes elliptical in the centre of the shaft and sub-rectangular at the distal end. In medial or lateral view the bone is straight, and the posterior margin of the proximal end slightly overhangs the rest of the bone (Fig 69C and 69D). In posterior view there is a low ridge or protuberance situated at a point approximately two-thirds from the proximal end of the bone towards the lateral margin of the shaft. In proximal view the radius has a teardrop-shaped outline that is pointed medially and rounded laterally; the articular surface is shallowly concave and slightly rugose (Fig 69E). The distal end has a sub-elliptical outline that has an irregular surface which is strongly rugose (Fig 69F). The radius appears to be similar to those of *Dacentrurus* sp. (ML 433), *Dacentrurus armatus* (NHMUK OR46013; [29]: Fig 9F and 9G), and *Kentrosaurus* (MB R.4806; [36]: pl. 4.4–6).

#### Carpus and manus

No carpal elements or metacarpals were recovered. A small phalanx and an ungual phalanx were recovered close to the ulna: however, because they were found close to the left astragalus and the ungual phalanx is very similar in size and morphology to another found in association with the pes, we consider it is most likely that these elements are pedal elements that have drifted a short distance from the left foot. Therefore, no elements from either manus were recovered.

### Pelvic girdle and hind limb

#### Ilium (Fig 31)

Only the right ilium is preserved and it is fused to the sacrum. The preacetabular process has an inverted C-shaped transverse cross-section that is laterally convex and medially concave, as in *S*. *mjosi* (DMNH 29431), *S*. *homheni* (IVPP V4006), other specimens of *Stegosaurus* (SCRM pers. obs. 2014), and other stegosaur genera, such as *Dacentrurus armatus* (NHMUK OR46013; [29]: fig 5A, B), *Kentrosaurus* (MB R.4800; [33]: fig 25; [36]: pl. 4.7–9), *Tuojiangosaurus* (CV 209/210; [34]; [35]: fig 2a), *Chungkingosaurus* (CV 206; [34]; [35]: fig 2b) and *Huayangosaurus* (ZDM T7001; [31]: fig 29; [26]: fig 5a). It diverges from the sagittal plane, flaring laterally to form an angle of approximately 40 degrees with the midline (Fig 31A, 31B and 31D). The anterior tip is bluntly rounded. The posterior portion of the preacetabular process consists of dorsal and lateral surfaces divided by a distinct break in slope; anteriorly, these surfaces merge into a single dorsolateral surface. The preacetabular process is separated from the acetabular region of the ilium by a sharp angle in dorsal view (Fig 31D), as in other specimens of *Stegosaurus*, including *S*. *mjosi* (DMNH 29431) and *S*. *homheni* (IVPP V4006). The preacetabular process of the ilium of *Dacentrurus armatus* (NHMUK OR46013) differs from that of NHMUK PV R36730 in that it is shorter relative to the length of the ilium, its ventrolateral margin is convex in ventral view, rather than straight to concave as in NHMUK PV R36730, and it tapers anteriorly to a broadly rounded tip [29]. The preacetabular process is also different from *Dacentrurus* sp. (MIGM 5782; MIGM 4953; [39]: figs. 4B; 9E) from Portugal in that they possess a ventrolateral corner on the ventrolateral margin of the preacetabular process, and they taper to a point anteriorly. The angle between the preacetabular process and the acetabular region is much lower in *Tuojiangosaurus* (CV 209/210; [34]; [35]: fig 2a), *Chungkingosaurus* (CV 206; [34]; [35]: fig 2b) and *Huayangosaurus* (ZDM T7001; [31]: fig 29; [26]: fig 5a) and describes a gentle arc rather than a right angle, as it does in NHMUK PV R36730.

The dorsal margin of the ilium posterior to the preacetabular process and dorsal to the acetabulum is rotated laterally to extend posteroventrolaterally and is hypertrophied to form a subrectangular wing-like flange that overhangs the acetabulum, obscuring it in lateral view (Fig 31A and 31E), as in most stegosaurs [1] except for *Huayangosaurus*, in which this flange is much smaller [26]. The sacral ribs are fused medially to this surface (Fig 31A and 31B) and together the ribs and hypertrophied lateral flange form a flat, dorsally facing surface (Fig 31D). The preacetabular process extends anteroventrally relative to this surface. The dorsal and lateral surfaces of the hypertrophied lateral flange are strongly rugose due to poor preservation, and the posterior margin has suffered some breakage. Ventrally, the acetabulum is oblate, with the long axis extending anteroposteriorly (Fig 31A and 31B). It is deeply concave, with a prominent, rectangular pubic peduncle forming its anterior margin. The posterior margin of the acetabulum is formed by the ischiadic peduncle, which is indistinctly preserved. The sacral ribs are fused to the medial surface of the acetabulum, while its lateral surface blends indistinguishably into the ventral surface of the hypertrophied lateral flange. The postacetabular process was broken off.

#### Ischium (Fig 70)

**Fig 70:**
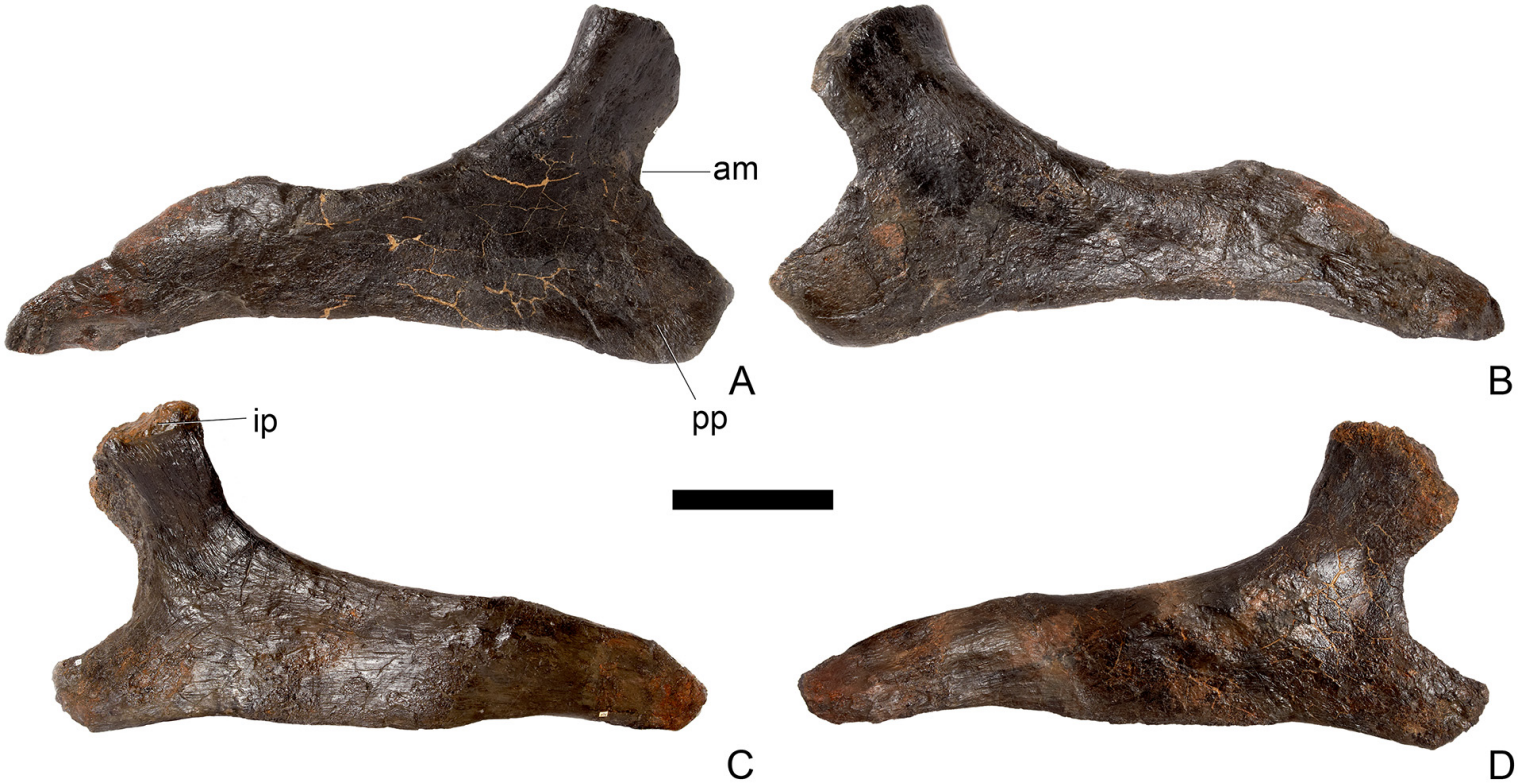
Ischia. **A, B**, right ischium and **C, D**, left ischium in **A, C**, lateral and **B, D,** medial views. **Am**, acetabular margin; **ip**, iliac peduncle; **pp**, pubic peduncle. Scale bar equal to 10 cm.

Both ischia are present, but the right is in better condition than the left: the right ischium is complete, except for its iliac peduncle, whereas the left ischium has been skimmed with plaster on its medial and lateral surfaces, and its distal end is artificially bent medially. In lateral view, the ischium is sub-triangular in outline with the distal end, iliac peduncle and pubic peduncle forming its three corners (Fig 70A and 70C). The dorsal iliac and ventral pubic peduncles are separated by a deep, concave notch anteriorly, which represents the free acetabular margin. In lateral view, the dorsal and ventral margins of the iliac process are sub-parallel so it is sub-quadrate in outline. The articular surface of the iliac peduncle faces dorsally and has a narrow ovate outline with the long axis of the oval trending anteroposteriorly. It tapers posteriorly, is rugose, and is transversely and anteroposteriorly convex. In lateral view, the pubic peduncle comprises dorsal and ventral surfaces that are separated by a distinct change in slope. The dorsal surface faces dorsolaterally and the ventral surface faces laterally. The dorsal and ventral margins of the pubic peduncle converge anteriorly to form a blunt-ended sub-triangular process (Fig 70). The articular surface of the pubic peduncle is transversely compressed and is not rugose.

Posterior to the peduncles, the shaft of the ischium is flat laterally and tapers posteroventrally. The dorsal margin of the ischium posterior to the iliac peduncle is sinuous, initially being concave dorsally, then convex dorsally, while the ventral margin is gently upwardly concave along its length. The distal part of the shaft is therefore offset at an angle of around 15 degrees from the long axis of the proximal part of the shaft. This contrasts with the condition in *Dacentrurus armatus* (NHMUK OR46013; [29]: fig 14H), where the dorsal margin of the ischial shaft is straight, but is similar in morphology to *Loricatosaurus* (NHMUK PV R3167; [29]: fig 14V) and *Kentrosaurus* (MB R.4550; [33]: fig 32, figured upside down; [36]: pl. 5.17–18). The dorsal margin of the ischium is transversely expanded relative to the ventral margin, with the latter being a thin flange. In medial view, the ischium is flat to gently concave, and the distal end is slightly rugose. The convex portion of the dorsal margin bears rugosities on its dorsomedial surface. In medial view, both the iliac and pubic peduncles are gently convex (Fig 70B and 70D). In dorsal view, the distal end of the ischium extends posteromedially. The ischium is very similar in all respects to those of other specimens of *Stegosaurus* (SCRM pers. obs. 2014), that of *S*. *mjosi* (DMNH 29431), *Loricatosaurus* (NHMUK PV R3167; [29]: fig 14V) and *Kentrosaurus* (MB R4550; [33]: fig 32; [36]: pl. 5.17–18).

#### Pubis (Fig 71)

Although parts of both pubes are preserved, neither is complete. On the right side, the prepubis and acetabular part are complete except for small amounts of restoration to the area immediately ventral to the obturator notch, while the shaft is damaged and has been partially restored. On the left side the acetabular part of the pubis is largely reconstructed, although the prepubis and shaft are mainly intact.

**Fig 71:**
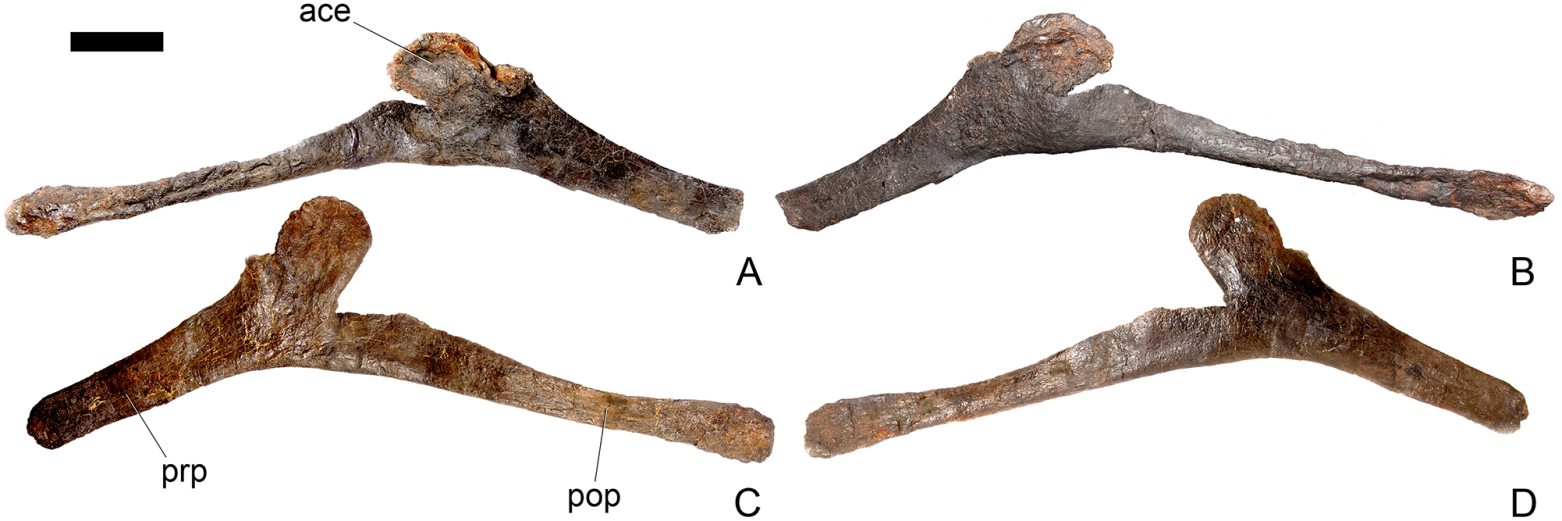
Pubis. **A, B**, right pubis and **C, D**, left pubis in **A, C**, lateral and **B, D,** medial views. **Ace**, acetabular part; **pop**, pubic shaft; **prp**, prepubis. Scale bar equal to 10 cm.

For convenience the pubis can be divided into three regions: an elongate prepubis, the small acetabular region, which bears the iliac and ischiadic articulations; and an elongate pubic shaft arising from the ventral margin of the acetabular region. The pubic shaft is retroverted and its ventral margin forms an angle of approximately 140 degrees with the ventral margin of the prepubis in lateral view (Fig 71). The prepubis tapers slightly as it extends anteriorly from its origin, but largely maintains the same width for the remainder of the process, terminating in a bluntly-squared-off anterior margin (Fig 71D), as in *Kentrosaurus* (MB R.4810). In contrast, the anterior margins of the prepubes in *S*. *mjosi* (DNMH 29431) and *Dacentrurus* sp. (ML 433) are slightly expanded dorsoventrally relative to the rest of the process, while that of *Dacentrurus armatus* (NHMUK OR46013: [29]; fig 14H) is significantly expanded. The dorsal and ventral margins of the prepubis are thus sub-parallel along its entire length. The lateral surface of the prepubis is very gently convex anteroposteriorly but otherwise bears no ridges, processes or rugosities. The prepubis is transversely compressed, thin and plate-like and extends anterolaterally in dorsal view in life position.

The acetabular region forms a sub-rectangular plate with a straight ventral margin, and curved posterior and dorsal margins in lateral view (Fig 71). The ventral part of the posterior margin represents the ischiadic articular surface while the dorsal part represents the free acetabular margin. The ischiadic and acetabular margins are approximately equal in length and are quite short. The iliac articular surface is approximately twice the length of the ischiadic margin. It is difficult to ascertain the boundaries of the articular surfaces because there are no clear boundaries between them. The lateral and medial surfaces of the acetabular region are both flat to gently concave. The acetabular region is a transversely thin sheet that is thickest dorsally and anteriorly and thins posteriorly. Most of the expansion of the anterodorsal portion of the acetabular region is on the lateral side, forming a pedestal to accommodate the iliac articular surface. In dorsal view the iliac articulation is L-shaped with down-stroke of the ‘L’ extending parallel to the long axis of the pubis, and the short branch extending laterally. The articular surface is irregular but includes a large central concavity. Immediately ventral to the articular surface is the broad base of the pubic shaft which extends posteroventrally in lateral view; in dorsal view the shaft extends posteromedially to meet its counterpart in a symphysis. In lateral view the acetabular region and pubic shaft define the borders of an anteroposteriorly elongate but dorsoventrally narrow and slit-like obturator notch, which is open posteriorly. The lateral surface of the shaft is concave ventral to the obturator notch so that the notch is situated in a deep sulcus, whereas the ventral margin of the shaft is convex. The maximum dorsoventral width of the shaft is situated in its proximal part immediately ventral to its junction with the acetabular region. In its proximal quarter the shaft narrows dorsoventrally and the middle portion of the shaft maintains the same width. Distally, the shaft expands again dorsoventrally to form a pubic symphysis (Fig 71) that is similar to those of *Loricatosaurus* (NHMUK R3167; [29]: fig 14V) and *Kentrosaurus* (MB R.4810). It also expands distally in *S*. *mjosi*, (DNHM 29431) and *Huayangosaurus* (ZDM T7001; [31]: fig 30) but to a greater degree. The distal end of the shaft is rounded in lateral view. In medial view the pubic symphysis is flat to gently concave, in contrast to the otherwise convex medial surface of the shaft.

#### Femur (Fig 72)

Both femora are essentially complete though each has suffered some anteroposterior crushing. This crushing is particularly pronounced on the left femur and much of the anteromedial margin of the left femoral shaft is reconstructed.

**Fig 72:**
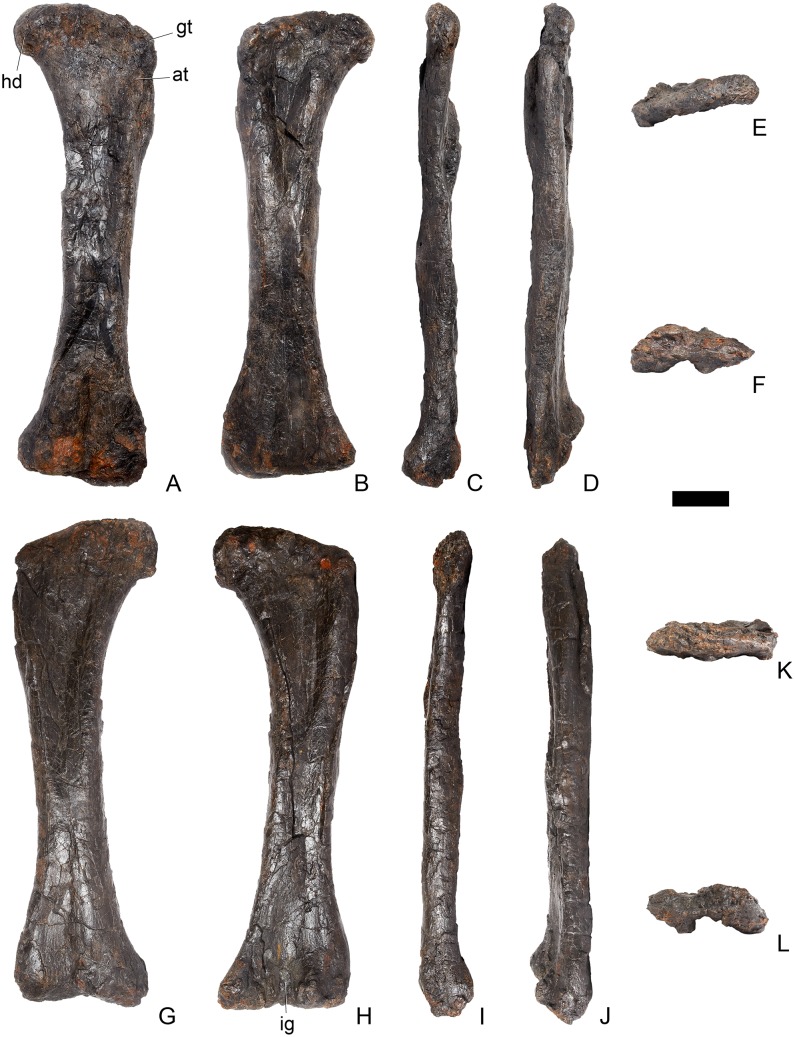
Femur. **A-F**, left femur and **G-L**, right femur in **A, G**, anterior, **B, H**, posterior, **C, I**, medial, **D, J**, lateral, **E, K**, dorsal and **F, L**, ventral views. **At**, anterior trochanter; **gt**, greater trochanter; **hd**, head; **ig**, intercondylar groove. Scale bar equal to 10 cm.

In anterior view the femur is straight and columnar and consists of an elongate shaft linking the transversely expanded proximal and distal ends (Fig 72A and 72G). The femoral head angles medially and slightly dorsally. The ventral margin of the femoral head is continuous with the medial margin of the shaft, describing a smooth curve, and lacking the sharp, angular break that separates these surfaces in larger individuals [43]. The medial margin of the head is gently convex and this is separated from the dorsal margin of the head by a distinct break in slope of around 90 degrees. Lateral to the head the dorsal margin of the greater trochanter slopes ventrolaterally and its dorsolateral corner forms an angle of around 120 degrees with the lateral margin of the shaft. The anterior surface of the proximal end is flat, although in the right femur this area has been depressed by crushing. In proximal view, the femur has an elongate sub-elliptical outline whose long axis trends transversely. The proximal articular surface is heavily rugose, particularly in the region of the head. There is no distinct constriction separating the head from the greater trochanter.

A distinct anterior trochanter is present on the anterolateral surface of the femur. Its dorsal margin is situated well below that of the greater trochanter (Fig 72A and 72G). It forms a prominent, finger-like ridge that extends parallel to the lateral margin of the shaft, merging into the shaft ventrally at a point approximately one-third of the way down the femur. The dorsal margin of the anterior trochanter forms a sub-triangular process but is not separated from the body of the femur by a groove. A deep groove is present lateral to the anterior trochanter which is bounded by the lateral margin of the femur.

The central region of the shaft has a sub-quadrate outline with rounded corners. Distally the anterior surface of the shaft bears a shallow concavity lying immediately dorsal to the articular condyles. The condyles are sub-equal in size in anterior view with the medial condyle being transversely wider than the lateral condyle. In medial view, the femur is straight and the femoral head has a tear drop shaped articular surface in outline with its apex pointing ventrally. In posterior view, the proximal end of the femur is flattened by crushing. A stout, ridge-like fourth trochanter is present but does not form a distinct process. The dorsal margin of the fourth trochanter is pierced by a large, sub-elliptical nutrient foramen. The fourth trochanter is not visible in anterior view and is positioned at shaft mid-length. In posterior view the distal end of the femur bears a deep intercondylar groove that separates the two femoral epicondyles, and this grades dorsally into a flat, shallow concavity that extends for a short distance dorsally (Fig 72B and 72H). In distal view, the medial epicondyle is twice as broad as the lateral epicondyle. The medial epicondyle has a bluntly rounded sub-elliptical outline with the long axis extending anteroposteriorly and it extends further posteriorly than the lateral epicondyle. The lateral epicondyle has a sub-quadrate outline in distal view and is medially inset from the lateral margin of the distal end forming a distinct step (Fig 72F and 72L).

The femur appears similar to those of other stegosaurs including *Dacentrurus armatus* (NHMUK OR46013), *Loricatosaurus* (NHMUK PV R3167), *Kentrosaurus* (MB R.4800) and *Huayangosaurus* (ZDM T7001; [31]: fig 31).

#### Tibia (Fig 73)

**Fig 73:**
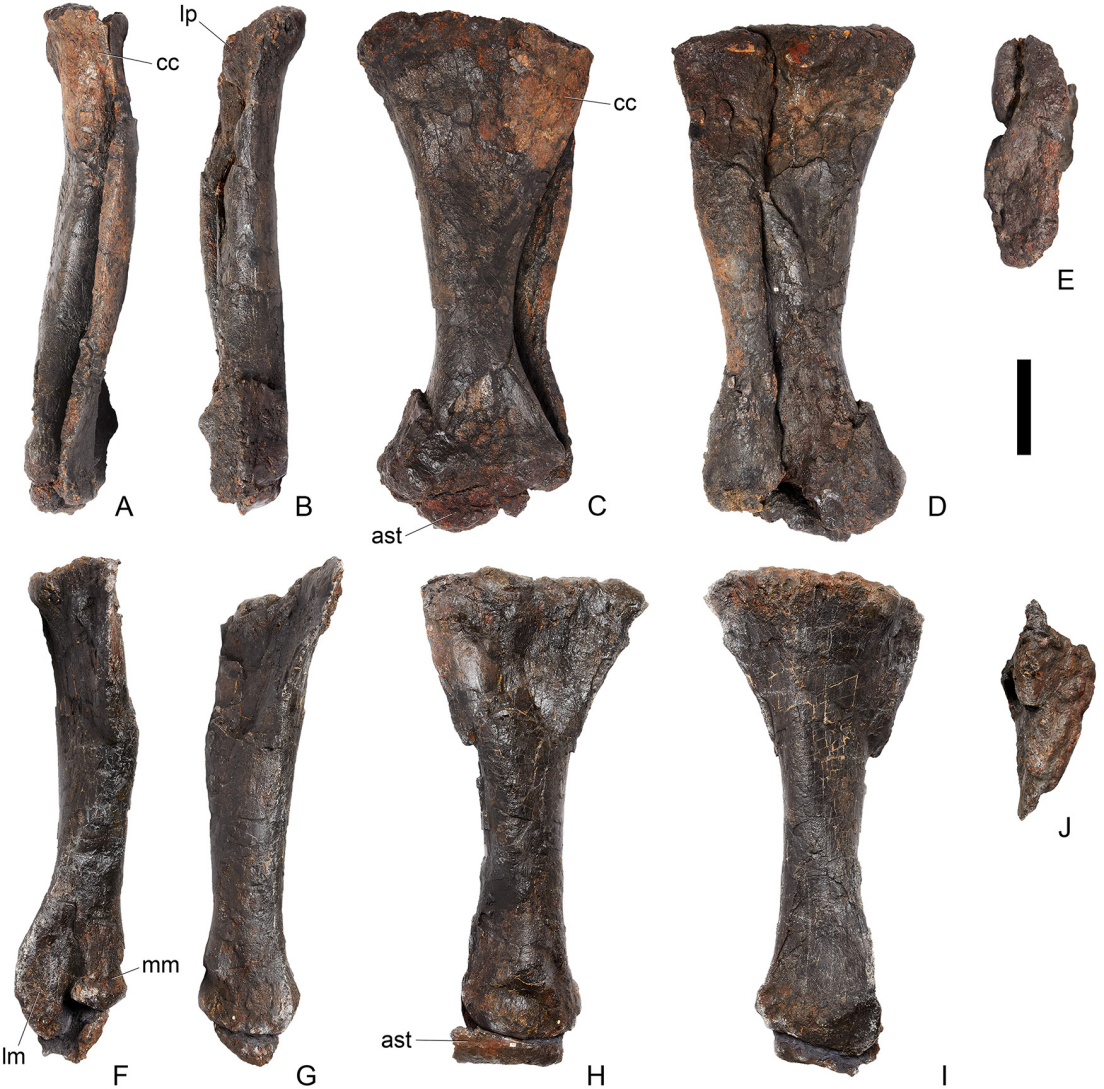
Tibiae and left fibula. **A-E**, left tibia and fibula and **F-J**, left tibia in **A, F**, anterior, **B, G**, posterior, **C, H**, medial, **D, I**, lateral, **E, J**, dorsal views. Note that the distal end of the tibia and fibula has been rotated and flattened relative to the proximal end, so anterior view of the proximal end is lateral view of the distal end. **Ast**, astragalus; **cc**, cnemial crest; **lm**, lateral malleolus; **lp**, lateral process; **mm**, medial malleolus. Scale bar equal to 10 cm.

Both tibiae are preserved. The right tibia is attached to the astragalus by plaster although these two bones are not fused (Fig 73F–73I), while the left tibia is articulated with the fibula and astragalus and the elements were probably fused (Fig 73A–73E). The right tibia is better preserved than the left, as it is less crushed, though both elements have suffered some degree of damage. The proximal end of the right tibia is crushed, whereas the lateral surface of the left tibia is now rotated so it lies in an anteroposterior plane and has a poorly preserved distal end. Nevertheless, most aspects of the anatomy can be determined by combining information from both elements.

In anterior view the tibia comprises a straight shaft with a transversely expanded distal end and a transversely compressed, crushed proximal end that is expanded anteroposteriorly (Fig 73A and 73F). The cnemial crest is represented by a sharp ridge that extends ventrally from the anterodorsal margin to a point approximately one-third of the way down the length of the shaft, where it terminates in an anteriorly extending lip (Fig 73C and 73H). Presumably it would have merged with the shaft originally, and the anteriorly extending lip is an artifact due to crushing. The shaft has a D-shaped cross-section with a flat anterior surface. The distal end is transversely expanded and comprises two surfaces: the medial and lateral malleoli, separated by an upwardly V-shaped groove (Fig 73F). The surface of the lateral malleolus is flat for articulation with the fibula, is situated slightly posteriorly to the medial malleolus and extends further ventrally than the medial malleolus. The medial malleolus is gently transversely convex. In lateral view the proximal end is expanded asymmetrically so that the posterior side extends further posterior than the cnemial crest does anteriorly.

In lateral view the cnemial crest is convex, while the posterior surface is concave, forming a fan-like posterior process, though the morphology of this area has been strongly altered by crushing. A large, rugose protuberance, the lateral process, projects laterally from the dorsal margin of the shaft at a point approximately one-quarter of the distance from the anterior margin of the cnemial crest (Fig 73E and 73J). This overhangs the lateral surface of the shaft and merges into it a short distance ventrally. Posterior to the lateral process, an intramuscular ridge extends ventrally across the lateral surface to about the mid-length of the tibia, where it merges with the shaft. The distal end of the tibia is triangular in cross section with flat to gently concave posterolateral and posteromedial surfaces. In proximal view the tibia is triangular in cross-section with a long straight medial margin and the lateral process forming the apex. The surface is rugose and has been restored with plaster. Details of the ventral surface are obscured by the attached astragalus but it appears to be concave.

#### Fibula (Fig 74)

**Fig 74:**
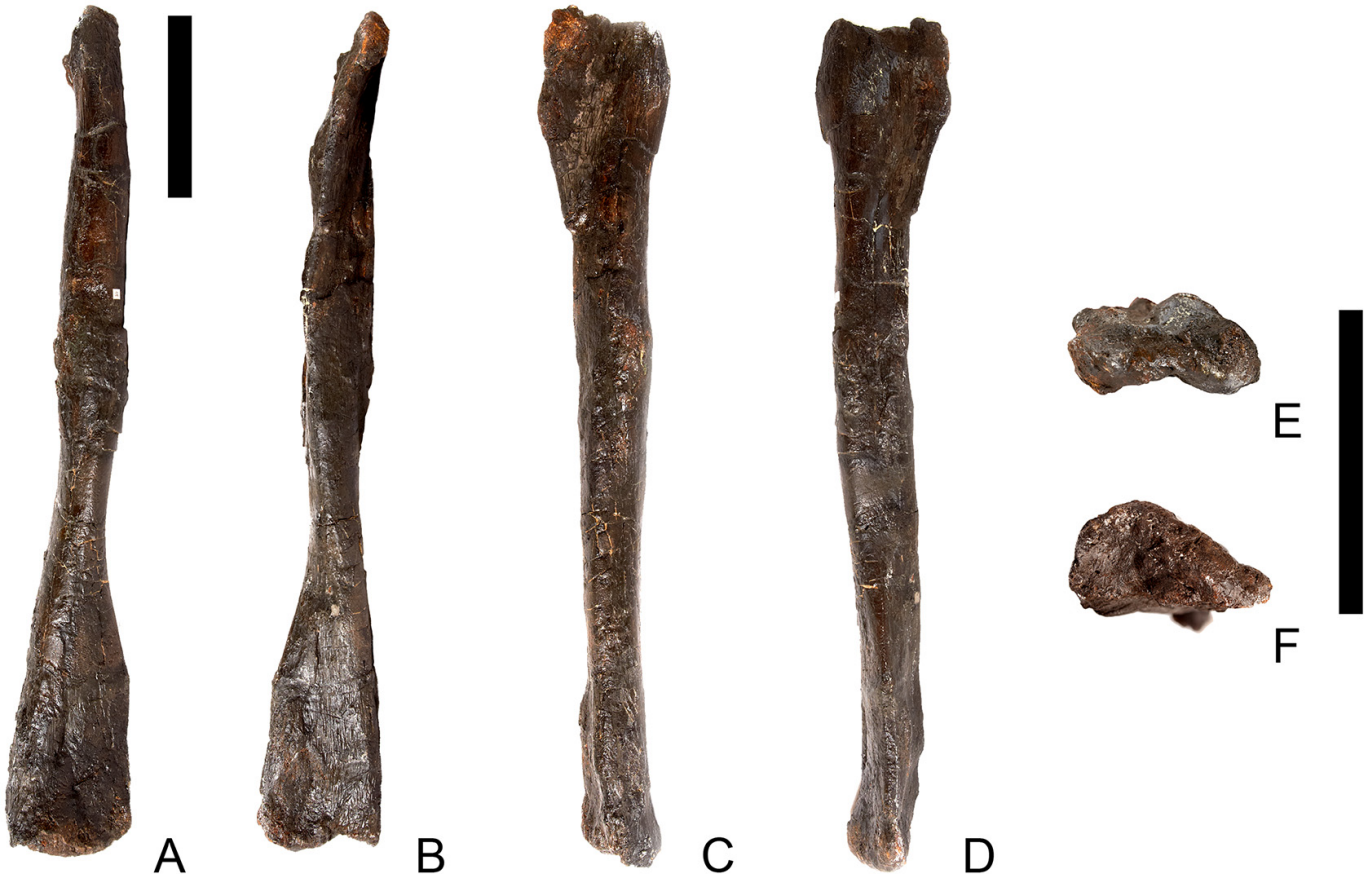
Right fibula in **A**, anterior, **B**, posterior, **C**, lateral, **D**, medial, **E**, dorsal and **F**, ventral views. Scale bars equal to 10 cm.

Both fibulae complete. The left element, which is appressed to the left tibia (Fig 73), has been compressed and the proximal end has been rotated to lie in the same plane as the distal end, which is crushed. The right fibula (Fig 74) is only slightly distorted, but it is damaged distally.

The fibula is straight in both anterior and lateral views, and is expanded anteroposteriorly proximally and transversely distally, and undergoes some torsion along its length. The shaft is D-shaped in cross-section, with the flattened surface facing medially. In lateral view the proximal end is expanded asymmetrically so that the anterior margin is straight but the posterior margin flares posteriorly and is convex. The proximal surface where the fibula articulates with the tibia is flattened and triangular. In proximal view the fibula is D-shaped in cross-section with the flat surface lying medially. On the left side, there is some evidence that the fibula articulated with the astragalus, although due to distortion of the distal ends of the fibulae and of the astragali, this is difficult to determine.

#### Astragalus (Fig 75)

**Fig 75:**
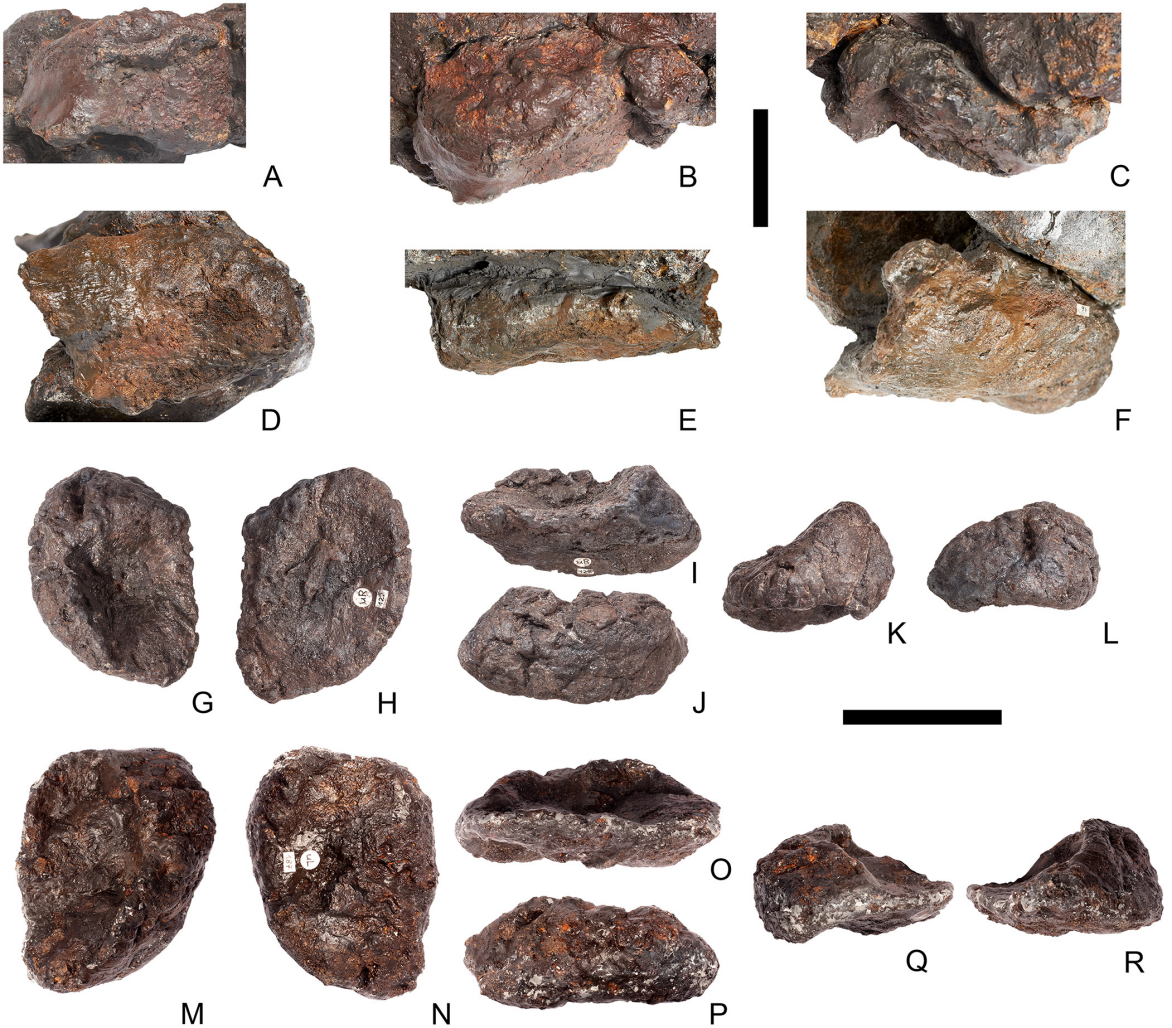
Tarsus. **A-C**, left astragalus; **D-F**, right astragalus, in **A, D**, ventral, **B, E,** anterior and **C, F**, posterior views. **G-L**, left calcaneum; **M-R**, right calcaneum, in **G, M**, dorsal, **H, N**, ventral, **I, O**, medial, **J, P**, lateral, **K, Q**, anterior and **L, R,** posterior views. Scale bars equal to 5 cm.

Both astragali are preserved, but the left astragalus is in poor condition and is incomplete. Both are attached to their respective tibiae so their dorsal surfaces are obscured (Fig 75A–75F). [2] noted that the astragalus was fused to the tibia in most of the USNM *Stegosaurus* specimens, while the calcaneum was often unfused. In *Loricatosaurus* (NHMUK PV R3167; [29]: fig 22M–P) and *Kentrosaurus* (MB R.4800; [36]: pl. 6.10–12), both the astragalus and calcaneum are fused to the crus. The astragalus is transversely broader than it is wide anteroposteriorly. The anterior, medial and posterior surfaces all merge seamlessly with the ventral surface to form an anteroposteriorly convex and transversely flat surface (Fig 75F). In anterior view the astragalus is dorsoventrally tallest at a point approximately two thirds of the distance from its medial end and tapers laterally, forming a blunt lateral margin and giving it a sub-oval outline in anterior view. The posterior surface is dorsoventrally narrower than the anterior surface. The lateral margins of both astragali are poorly preserved but are ‘C’-shaped and tallest anteriorly. The dorsal surface is obscured either by the apposition of the tibia or plaster. There are no distinct processes along either the anterior or posterior margins of the astragalus, which is a simple, block like element. In ventral view the astragalus has a sub-elliptical outline with rugose margins.

#### Calcaneum (Fig 75)

Both calcanea are present (Fig 75G–75R), although the left is better preserved than the right. It is fused neither to the astragalus nor fibula. In ventral view the calcaneum has an elliptical outline with a straight medial margin and rounded anterior, lateral and posterior margins and all are rugose (Fig 75H and 75N). The ventral surface is gently concave. In anterior and posterior view it has a sub-triangular outline with the apex of the triangle sloping medially towards the astragalus (Fig 75K, 75L, 75Q and 75R). The posterior surface smoothly grades into the lateral and anterior surfaces without a distinct break in slope. This combined surface is gently convex dorsoventrally, and in dorsal view has a crescentic outline. In lateral view it is sub-trapezoidal. The bone is thickest laterally and tapers medially. The dorsal surface is excavated to form a deep sub-semicircular articular surface for the distal end of the fibula (Fig 75G). There is no indication of any contact between the tibia and calcaneum.

### Pes (Figs 76 and 77)

**Fig 76:**
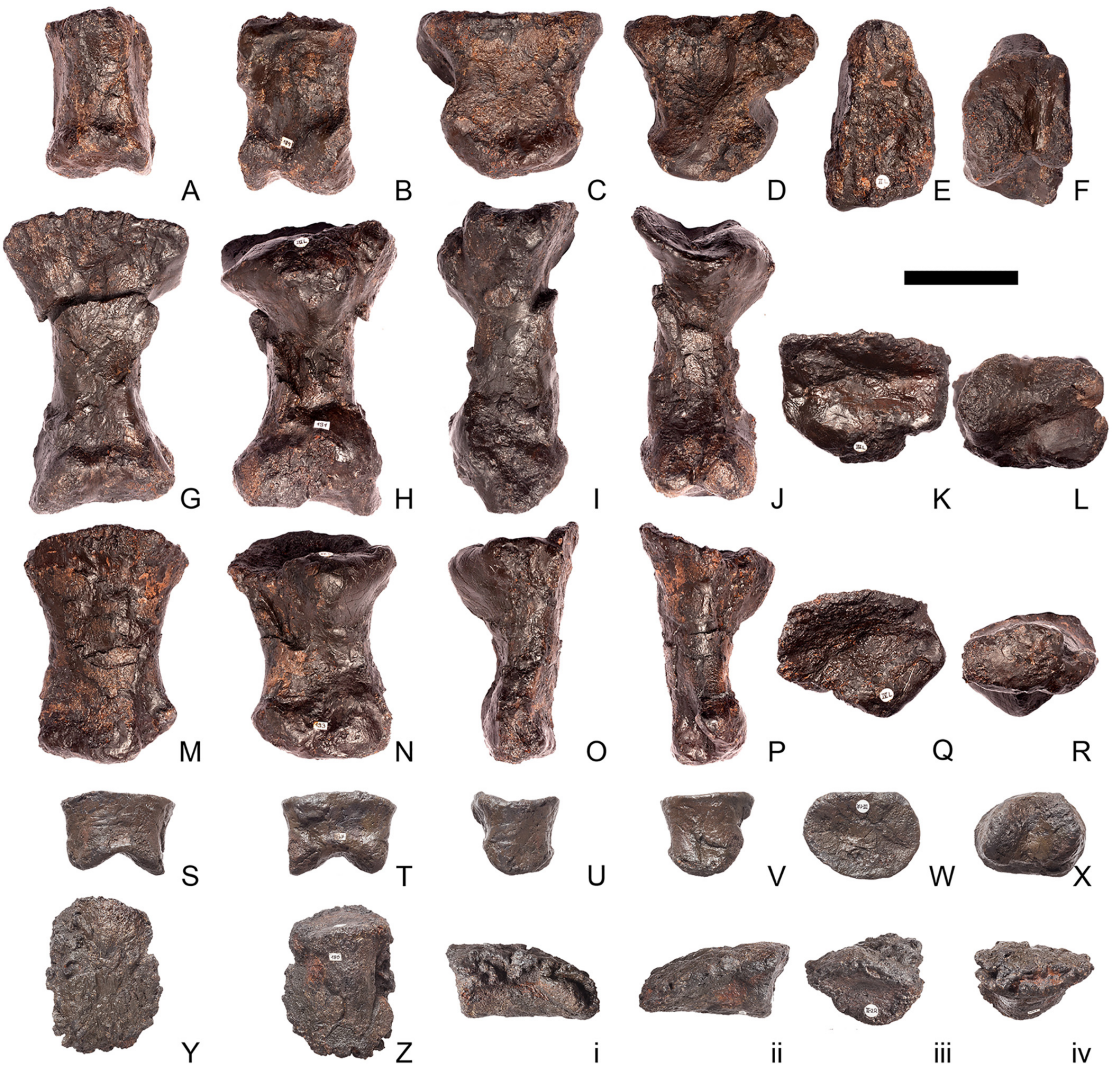
Left pes. **A-F**, Metatarsal 2; **G-L**, metatarsal 3; **M-R**, metatarsal 4; **S-X**, phalanx; **Y-iv**, ungual phalanx in **A, G, M, S, iv**, anterior, **B, H, N, T, iii**, posterior, **C, I, O, U, i,** medial, **D, J, P, V, ii**, lateral, **E, K, Q, W, Y**, dorsal and **F, L, R, X, Z**, ventral views. Scale bar equal to 5 cm.

Metatarsals 2–4 are preserved on both sides, although the left metatarsals are not as well preserved as those on the right. The elements on the left side have been restored with plaster and metatarsal 3 is crushed (Fig 76). Consequently, the description that follows is based on the right metatarsals. A phalanx and two unguals are also preserved (Fig 76S–76Z; i-iv). The phalanx was found in association with the left metatarsals, while both unguals were found along with the left calcaneum some distance away: given their association with the left calcaneum it seems likely they are also from the left pes.

**Fig 77:**
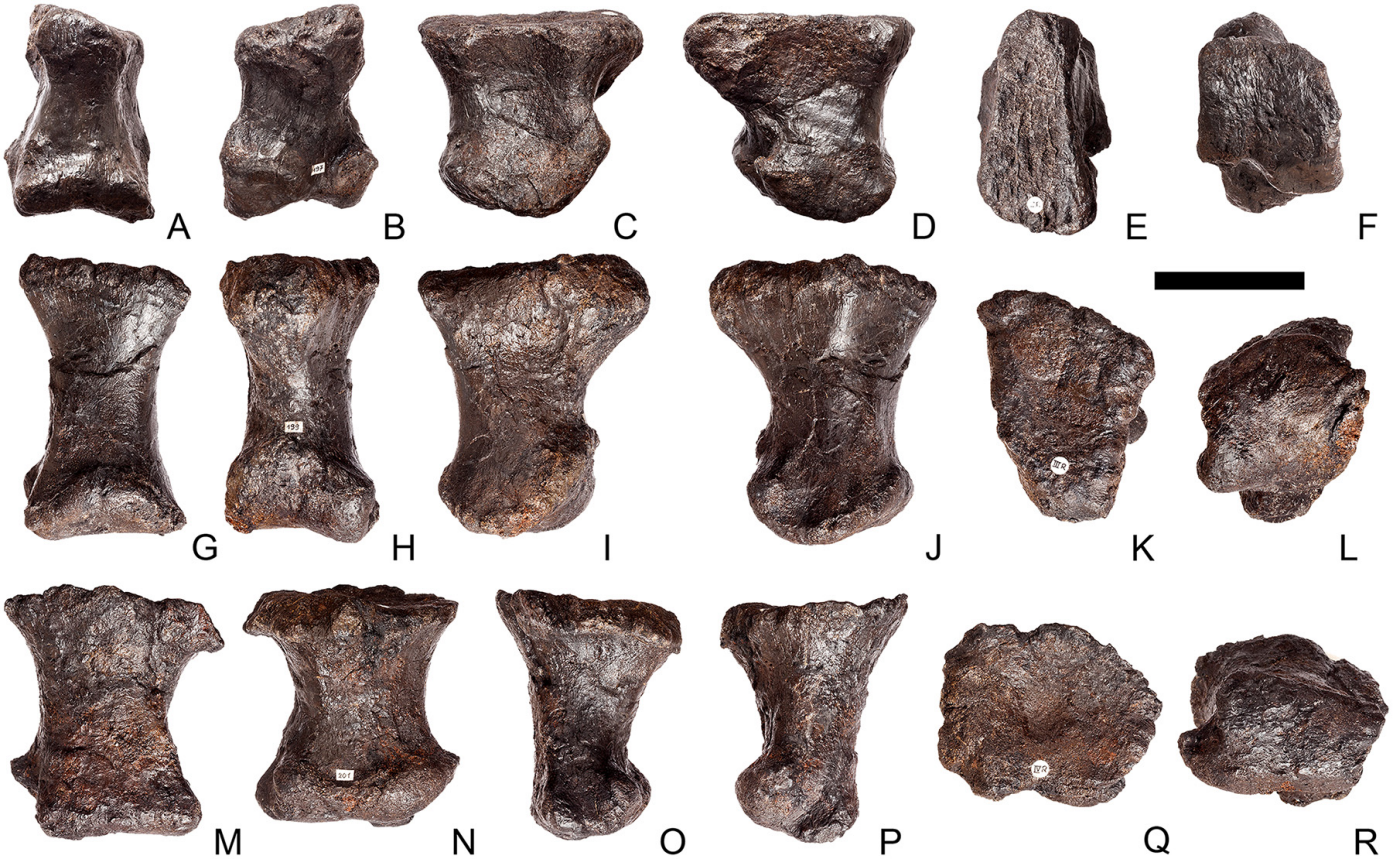
Right pes. **A-F**, Metatarsal 2; **G-L**, metatarsal 3; **M-R**, metatarsal 4 in **A, G, M**, anterior, **B, H, N**, posterior, **C, I, O,** medial, **D, J, P**, lateral, **E, K, Q**, dorsal and **F, L, R**, ventral views. Scale bar equal to 5 cm.

The metatarsals are closely appressed to each other. Metatarsal 3 is the longest metatarsal, and metatarsal 4 is longer than metatarsal 2. [2]: fig 53 noted that a fourth, rudimentary metatarsal was present in *Stegosaurus* (USNM 7419), but these elements were not recovered in NHMUK PV R36730.

#### Metatarsal 2 (Fig 77A–77F)

In proximal view, metatarsal 2 is sub-rectangular with the long axis trending anteroposteriorly. The medial and lateral surfaces are straight. It flares very slightly posteriorly, so that the posterior margin is slightly longer then the anterior margin. The dorsal surface is flat. In anterior view the element is transversely compressed with dorsal and ventral expansions. The dorsal margin of the dorsal expansion is upwardly convex. The anterior surface is sub-triangular or wedge-shaped in anterior view and wider ventrally. The articular surface for the phalanges wraps from the ventral surface onto the ventral-most part of the anterior surface. In medial view the metatarsal has a sub-quadrate outline with a long straight dorsal margin, a concave anterior margin, convex ventral margin forming the articular surface, and a straight posterior margin that slopes obliquely posterodorsally. A distinct notch separates the posterior margin from the ventral articular surface. The lateral surface is gently concave dorsoventrally forming a facet for the reception of metatarsal 3. In posterior view the metatarsal has a sub-rectangular outline with a saddle-shaped posterior surface. The dorsal margin of the bone overhangs the posterior surface and the posterior part of the articular surface for the phalanges extends onto the ventral part of the posterior surface. The articular surface for the phalanges bifurcates into weakly developed lateral and medial ginglymi separated by a shallow groove. The lateral ginglymus is half the size of the medial ginglymus. In medial view metatarsal 2 is dumbbell-shaped and the medial surface is gently concave. In ventral view the articular surface has straight medial, anterior and lateral margins. The posterior margin is notched. The medial part of the posterior articular surface extends further posteriorly than the lateral part. The articular surface for the phalanges is strongly convex anteroposteriorly and a shallow groove extends from the posterior groove anteriorly for a short distance.

#### Metatarsal 3 (Fig 77G–77L)

In proximal view metatarsal 3 has a sub-triangular outline that is longest anteroposteriorly. The medial and lateral surfaces meet to form the rounded apex of this triangle posteriorly. The articular surface is very gently concave and the margins are slightly rugose, especially anteriorly and laterally. The straight medial margin articulated with metatarsal 2 and the slightly rugose lateral margin with metatarsal 4. In anterior view metatarsal 3 is hourglass-shaped with sub-equal proximal and distal expansions joined by a waisted shaft. The articular surface for the phalanges wraps around the ventral-most part of the anterior surface. In lateral view the expansion of the proximal end is asymmetrical so that the posterior part overhangs the posterior margin of the bone. The anterior margin is slightly sinuous, the dorsal margin is straight, and the posterior margin is strongly concave in its proximal part but becomes convex ventrally to merge with the convex ventral margin of the bone. Most of the lateral surface is strongly concave dorsoventrally. A deep ligament pit is present at the distal end on the lateral surface, defined by a stout curved ridge that surrounds it posteriorly and ventrally. The dorsal margin of the distal articular surface forms a raised lip that is situated on the posterior surface just dorsal to the ligament pit. In posterior view the element is hourglass-shaped. In medial view the element is similar to that in lateral view but there is no ligament pit or ridge. The ventral surface is strongly convex anteroposteriorly and shallowly convex transversely. In contrast to metatarsal 2, it is not divided into ginglymi. The articular surface extends further up the shaft posteriorly than anteriorly.

#### Metatarsal 4 (Fig 77M–77R)

Metatarsal 4 consists of proximal and distal expansions linked by a short, stout shaft. The proximal expansion is transversely wider than the ventral one. In anterior view, the dorsomedial corner is angled slightly dorsomedially in anterior view but this may be the result of distortion. The medial and lateral surfaces of the shaft are mildly concave whereas the dorsal and ventral margins are straight. The articular surface wraps onto the ventral-most part of the anterior surface of the shaft only and does not extend as far dorsally as in metatarsals 2 and 3. In lateral view both the proximal and lateral ends are expanded relative to the shaft; the proximal expansion is greater. An indistinct ligament pit is present on the distal surface of the shaft defined anteriorly by a shallow ridge and posteriorly by a small sharp process (which is also visible in anterior and posterior views). The anterior shaft margin is straight to very slightly concave and the posterior margin is strongly concave, particularly in its ventral part, whereas the dorsal margin is straight. The posterior surface is similar to the anterior surface, and the medial surface is similar to the lateral surface except that there is no ligament pit. Instead there is a groove on the mediodistal part of the shaft in that position and it lacks the sharp process, although a posterior ridge is present in its place. In dorsal view the proximal surface has an oval outline that is broadest medially and tapers laterally. The ventral articular facet is transversely wider than it is long anteroposteriorly and convex.

In comparison with the metatarsals of *Kentrosaurus* (MB R.2951; [33]: fig 50; [34]: pl. 5) and *Huayangosaurus* ([31]: fig 34), those of NHMUK PV R36730 appear stockier, with transversely broader midshafts and less expanded dorsal and ventral ends. Otherwise, they are similar in overall shape.

#### Phalanx (Fig 76S–76X)

The proximal articular surface has a straight posterior margin and continuously curved lateral, anterior and medial margins giving it a D-shaped outline in dorsal view. This surface is shallowly concave. In anterior view, the phalanx has a sub-rectangular outline with a strongly concave ventral surface that divides it into two equal-sized portions. The anterior surface is gently concave dorsoventrally and gently convex transversely and merges into the lateral and medial surfaces with no distinct break of slope. The posterior surface is divided from the others by a slight break in slope and is gently concave dorsoventrally. The distal articular surface is saddle-shaped with a deep midline groove dividing it into distinct ginglymi. There are no distinct co-lateral ligament pits. In distal view the articular surface has a trapezoidal outline with a long posterior margin and lateral and medial margins that converge anteriorly to meet the shorter anterior margin.

#### Unguals (Fig 76Y–76iv)

Two unguals are preserved. One is well preserved, but the other is a rugose, pitted lump of bone that appears to be pathological. The following description is consequently based on the former. In lateral and medial views, the ungual is wedge-shaped, with a straight articular margin and dorsal and ventral margins that converge anteriorly to meet at a blunt point. The dorsal margin is convex, whereas the ventral margin is flat. The articular surface is oval in outline with the long axis trending transversely and is gently concave dorsoventrally. The margins of this facet are rugose. In dorsal view, the ungual is hoof-shaped and blunt-ended, with gently convex lateral margins that are offset from the articular region by shallow notches both medially and laterally. The area posterior to the notches is smooth, whereas the rest of the dorsal surface is strongly rugose. In ventral view, the surface is strongly convex transversely and smooth. Small pits are situated either side of this central convexity.

### Dermal armor (Figs 78–84)

Eighteen plates and four spines were recovered in place along the spine. When the specimen was discovered, there was a space above the proximal portion of the tail suggesting that another plate was originally present but became disarticulated post-mortem and was not preserved, so it is likely that the full complement of plates for *Stegosaurus* was 19, with four spines at the end of the tail. [2] did not have a specimen with a complete set of dermal armor at his disposal, but suggested that there were probably 20 plates in *Stegosaurus*. Since the armor was found largely *in situ*, the order of the plates preserved in NHMUK PV R36730 is likely to be correct, as are their anteroposterior orientations; however, it is not possible to determine whether the plates angled to the left or right side of the body. Herein, the plates are described as they are mounted. [45] presented a revised plate arrangement for NHMUK PV R36730 ([45]: fig S21). In this figure [45] suggested that the plate labeled herein as plate 5 belongs at the distal end of the tail. However, on the quarry map, Fig 3, it can be seen that plates 6, 8, 9 and 10 were found in articulation, with a further two small plates (plates 5 and 7) lying less than a meter from the cervical vertebral column. The plate labeled as plate 5 herein was therefore found associated with the anterior part of the skeleton, not the end of the tail, and we consider that it was located as described here and as shown on the mounted skeleton (SI 1). Measurements of plates can be found in Table 4, and spikes in Table 5.

**Fig 78:**
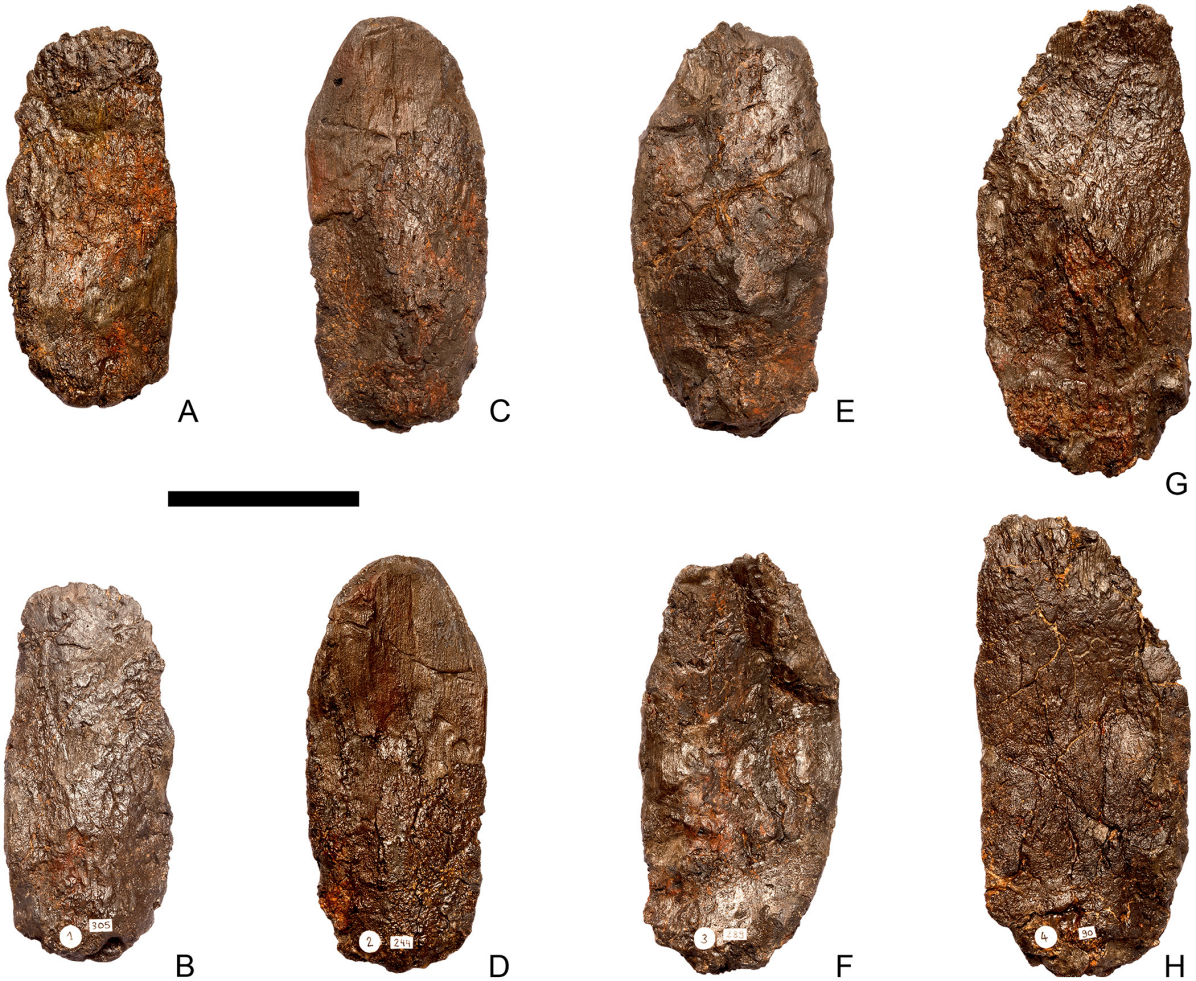
Plates 1–4. **A, B**, plate 1; **C, D**, plate 2; **E, F**, plate 3; **G, H**, plate 4 in **A, C, E, G**, left lateral and **B, D, F, H**, right lateral views. Scale bar equal to 10 cm.

**Fig 79:**
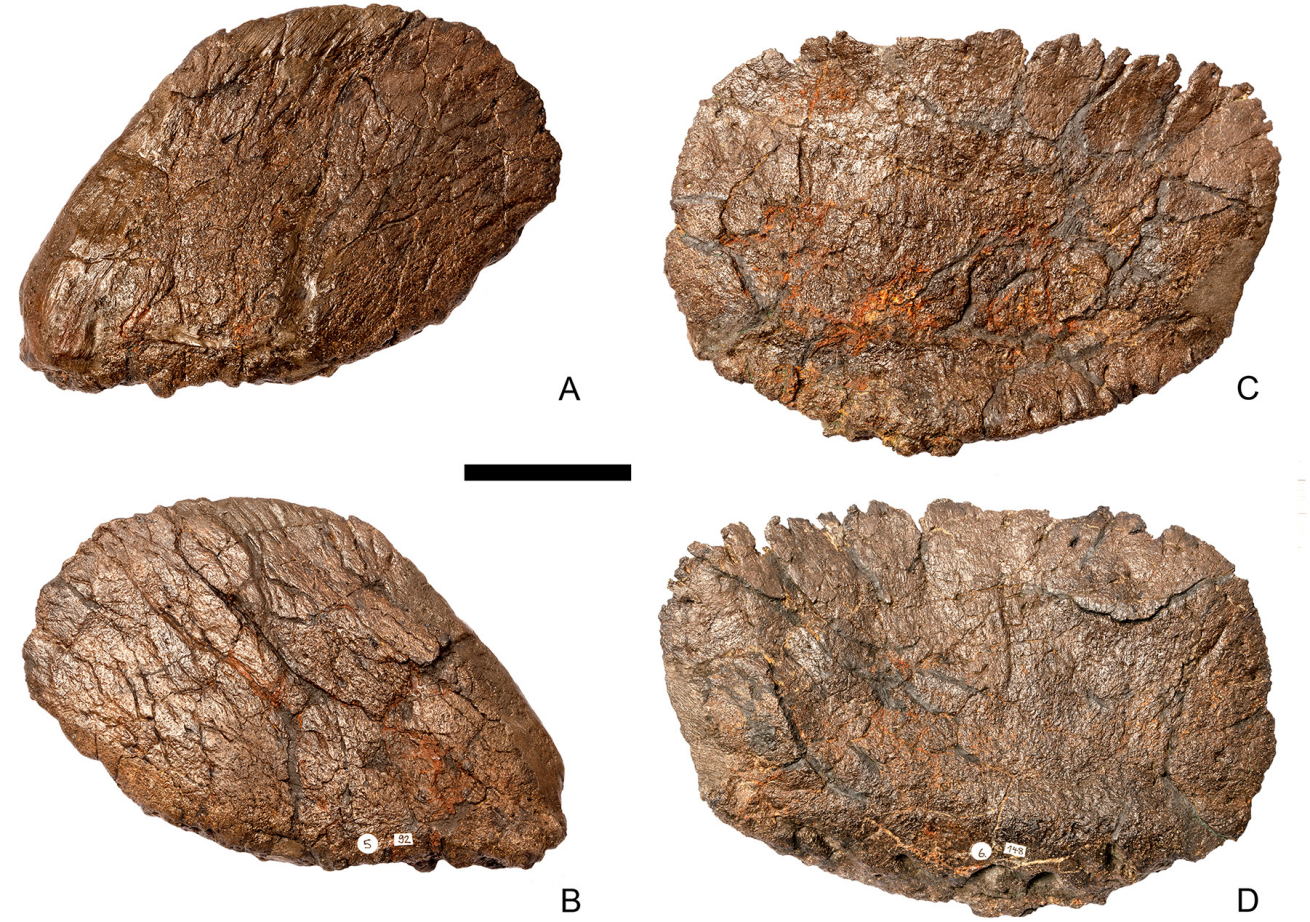
Plates 5–6. **A, B**, plate 5; **C, D**, plate 6 in **A, C**, left lateral and **B, D**, right lateral views. Scale bar equal to 10 cm.

**Fig 80:**
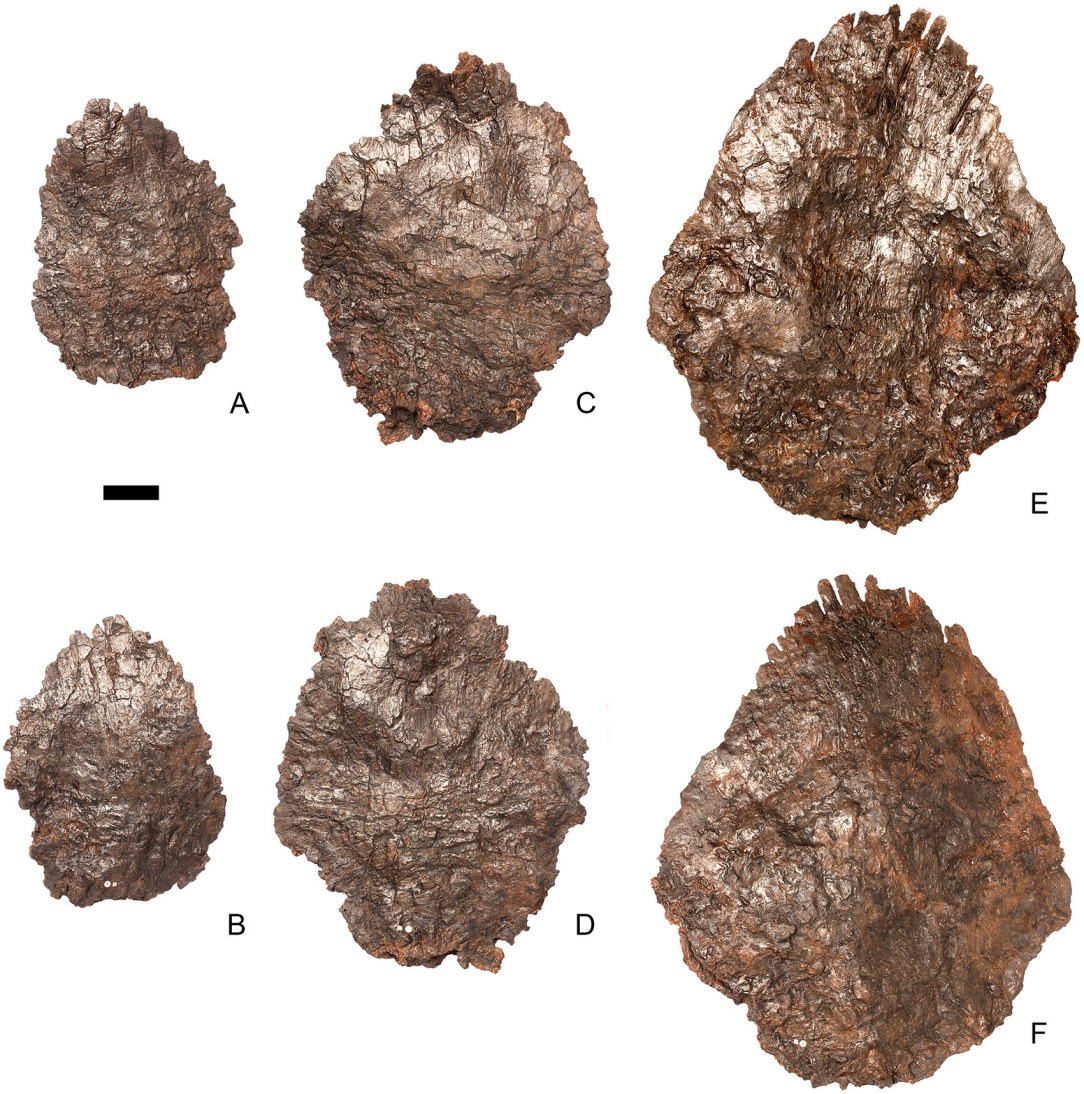
Plates 7–9. **A, B**, plate 7; **C, D**, plate 8; **E, F**, plate 9 in **A, C, E**, left lateral and **B, D, F**, right lateral views. Scale bar equal to 10 cm.

**Fig 81:**
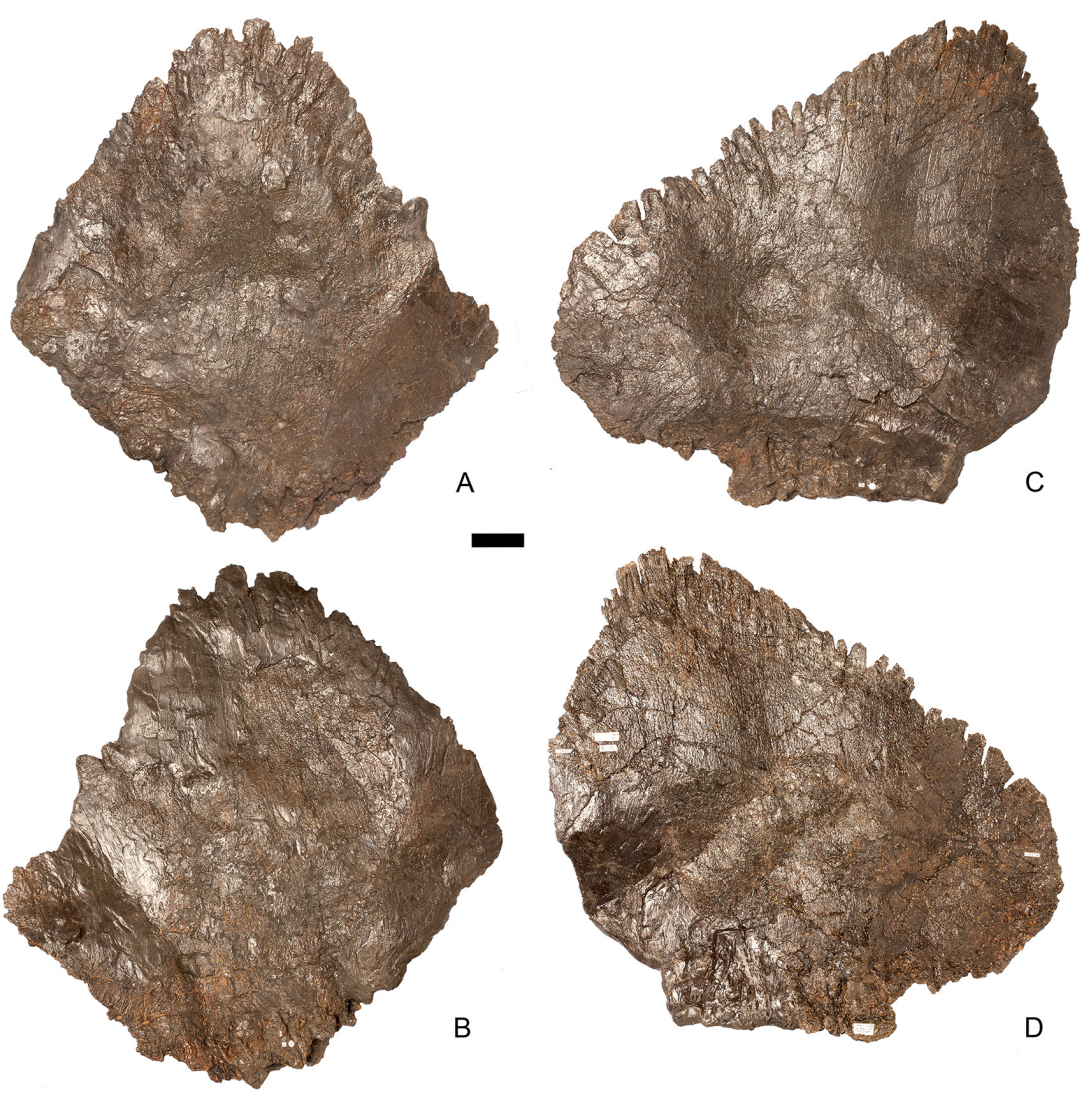
Plates 10–11. **A, B**, plate 10; **C, D**, plate 11 in **A, C**, left lateral and **B, D**, right lateral views. Scale bar equal to 10 cm.

**Fig 82:**
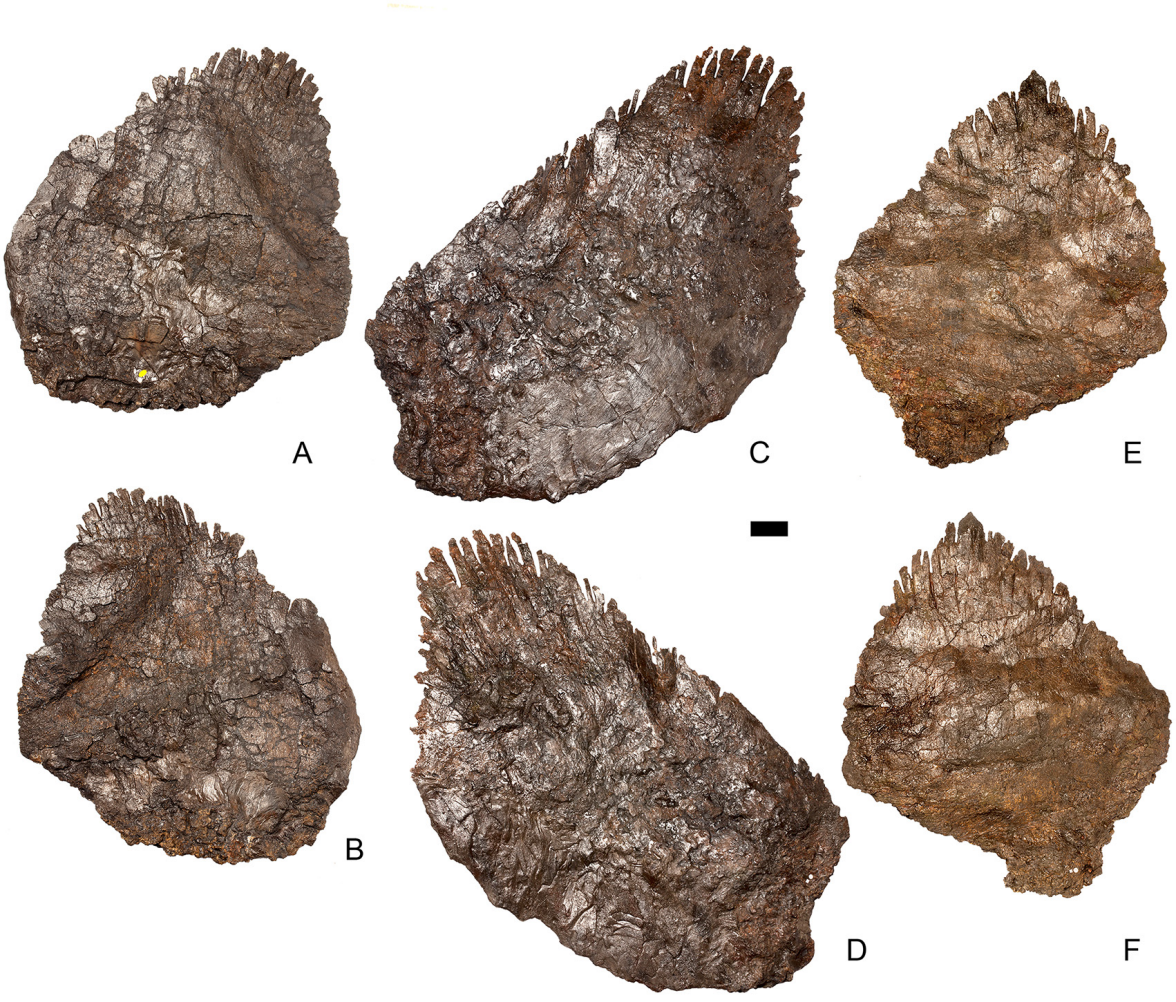
Plates 12–15. **A, B**, plate 12; **C, D**, plate 13; **E, F**, plate 15 in **A, C, E**, left lateral and **B, D, F**, right lateral views. Scale bar equal to 10 cm.

**Fig 83:**
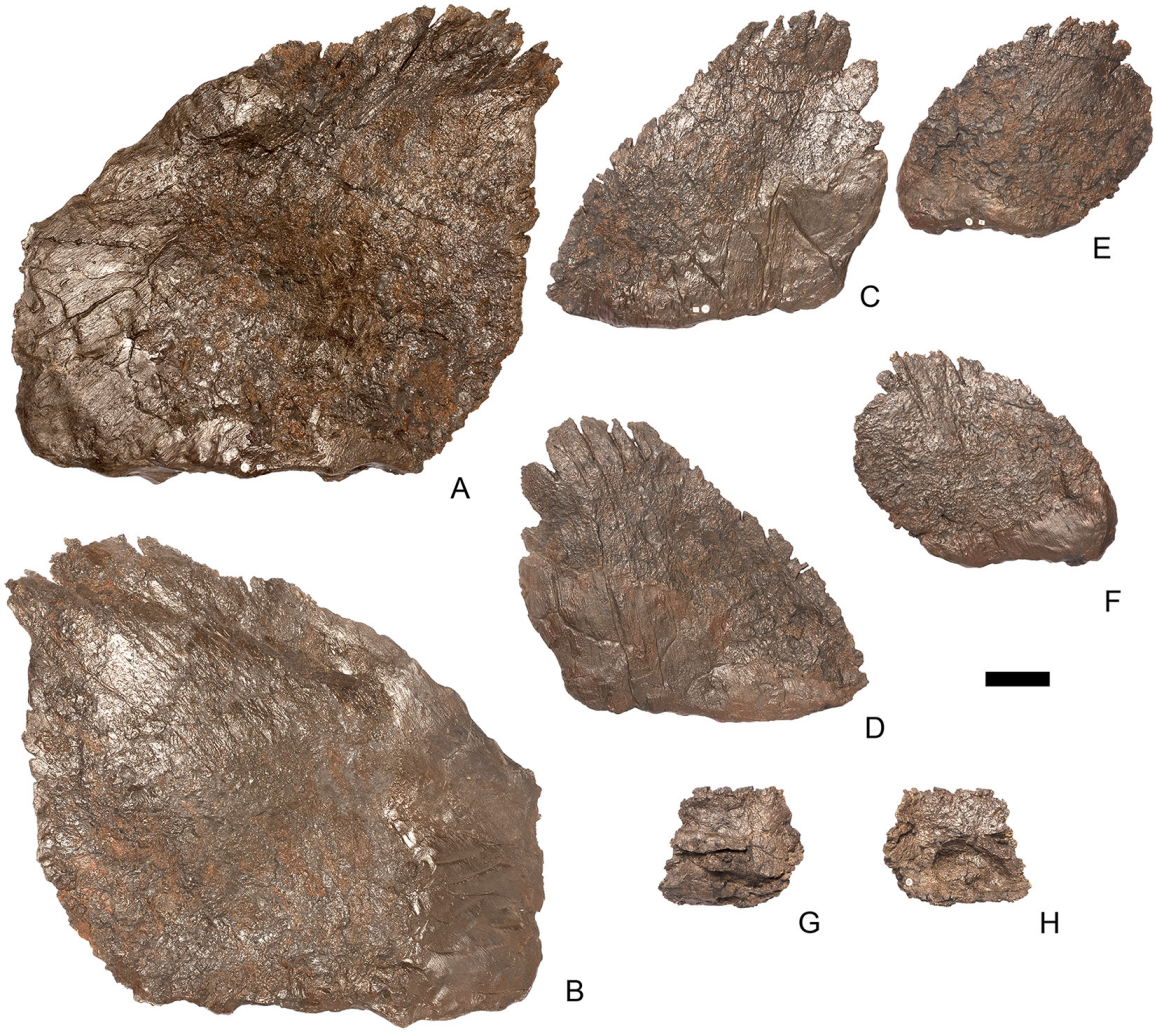
Plates 16–19. **A, B**, plate 16; **C, D**, plate 17; **E, F**, plate 18; **G, H**, plate 19 in **A, C, E, G**, left lateral and **B, D, F, H**, right lateral views. Scale bar equal to 10 cm.

**Fig 84:**
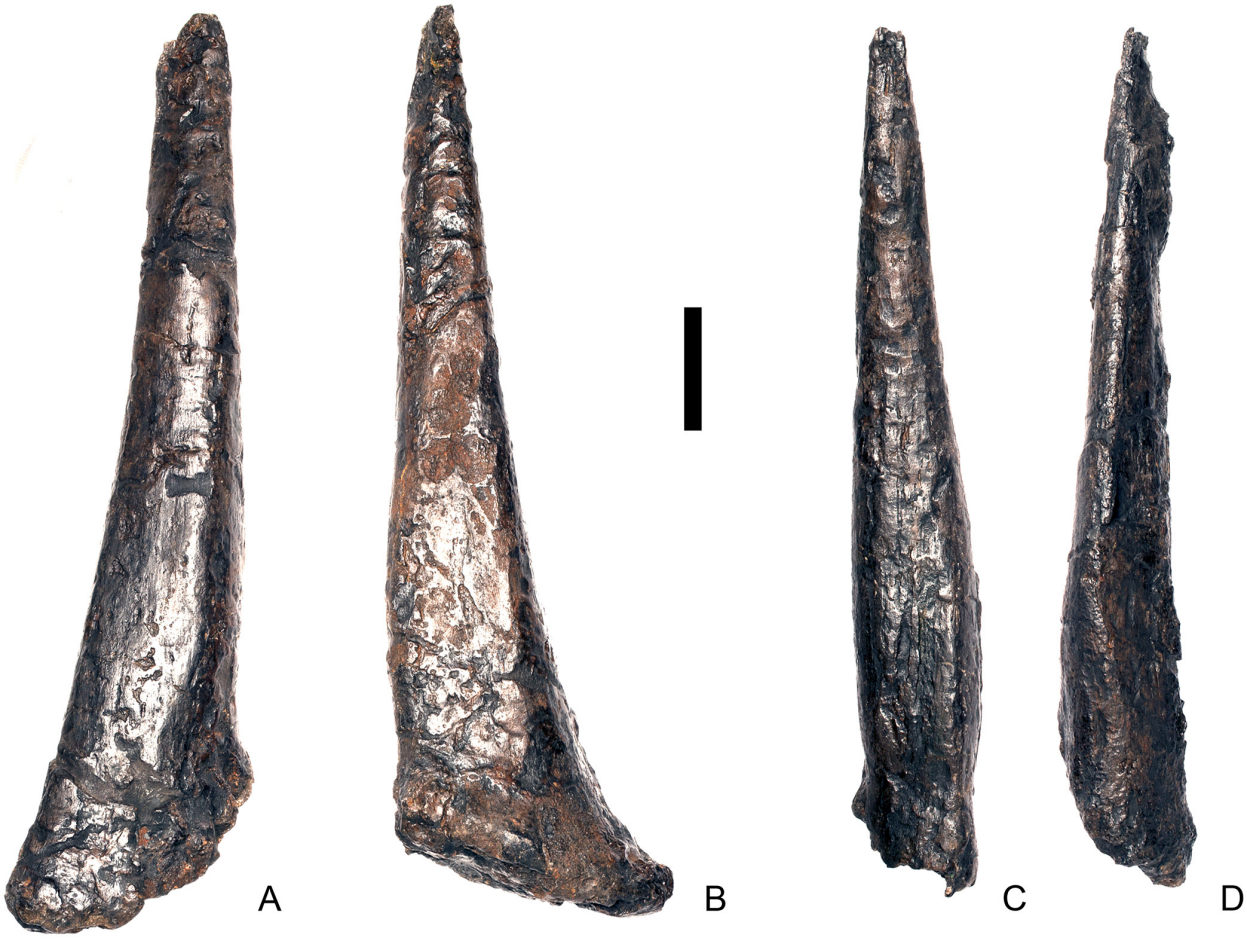
Tail spikes. **A, B**, anterior pair; **C, D**, posterior pair in lateral view. Scale bar equal to 10 cm.

**Table 4:** Measurements of plates in mm. All measurements are maxima.

	Maximum dorsoventral height	Maximum anteroposterior length
**Plate 1**	103	45
**Plate 2**	Poorly preserved	48
**Plate 3**	112	54
**Plate 4**	126	57
**Plate 5**	107	167
**Plate 6**	127	181
**Plate 7**	258	200
**Plate 8**	352	280 as preserved
**Plate 9**	463	368
**Plate 10**	490	465
**Plate 11**	445	447
**Plate 12**	535	460
**Plate 13**	785	
**Plate 15**	536	440
**Plate 16**	382 as reconstructed	Poorly preserved
**Plate 17**	Poorly preserved	Poorly preserved
**Plate 18**	175 as reconstructed	193
**Plate 19**	Poorly preserved	Poorly preserved

**Table 5:** Measurements of spikes in mm. All measurements are maxima.

	Spine height	Maximum anteroposterior length of base	Maximum transverse width of base
**Left 1**	380	124	57
**Right 1**	375	120	57
**Left 2**	345	58	60
**Right 2**	351	42	44

#### Plates 1–4 (Fig 78)

The four anterior-most plates (Pl) are almost identical in morphology, differing only in size. All are complete, with the exception of Pl2, which is missing its dorsal-most third, and this is reconstructed with plaster. The plates are elliptical in outline with the long axis of the ellipse directed dorsoventrally. They are transversely thickest along their ventral margins, which are strongly rugose and have sub-elliptical outlines in ventral view. Dorsally, the plates narrow in width to form thin vertically extending sheets that are reduced to a transverse thickness of only 3 mm around their dorsal margins. The anterior margins of the plates are slightly thickened with respect to the rest of the dorsal part of the plates. In lateral view, the margins of the plates are smooth and merge into each other with no distinct breaks of the slope and the plate apices are broadly rounded. The lateral and medial surfaces are coarsely textured, but there are no distinct vascular grooves. The maximum anteroposterior width of these plates lies at a point approximately level with their mid-height, and they contract in anteroposterior width both dorsal and ventral to this point. The bases of these plates are symmetrical in anterior and posterior view.

#### Plate 5 (Fig 79A and 79B)

Pl5 is parallelogram-shaped in lateral view, and the plate apex overhangs the posterior margin of the base. It is longer than tall. The anterior margin is straight and slopes posterodorsally, the dorsal margin is convex, the posterior margin is parallel to the anterior margin, but shorter, and the basal margin is very gently convex. The margins of the plate are smooth. The basal region is transversely expanded with respect to the rest of the plate, which tapers in thickness dorsally to form an isosceles triangle-shaped cross-section. The ventral margin of the basal region is strongly rugose and has a narrow, elongate elliptical outline. Numerous fine, shallow vascular grooves extend posterodorsally across both plate surfaces. The base of the plate is symmetrical in anterior and posterior view.

#### Plate 6 (Fig 79C and 79D)

Pl6 differs from Pl1–5 in that its base is asymmetrical: when the base is held vertically, the plate is angled dorsolaterally in anterior view, and in the mount the plate is angled to the left. The plate has a sub-oval outline, with a convex, dorsally orientated anterior margin that merges smoothly with the straight and only slightly dorsally extending dorsal margin. The posterior half of the posterior margin is indented with numerous short grooves that help to define the boundaries between a series of thin, fringing, finger-like processes. The dorsal margin of the plate is also the longest. Posteriorly, a distinct break in slope separates the dorsal and posterior margins; the latter is straight and extends ventrally and slightly anteriorly. The ventral margin is convex and rugose. The lateral surface bears some evidence of vascular grooves, but these are neither as numerous nor as well defined as on Pl5. In ventral view, the attachment surface is rugose and sub-crescentic in outline. A distinct break in slope separates the ventral surface from the medial surface, forming a low ridge that divides the surface that was presumably embedded in the dermis from that which was free. The plate is thickest basally and tapers dorsally. The anterior and posterior margins are equally thin.

#### Plate 7 (Fig 80A and 80B)

Pl7 is not well preserved and little surface detail is visible. The basal region of the plate is crushed and the margins are damaged and incomplete. However, the plate appears to have been taller than long and oval in outline, being broader basally and tapering apically, and having its maximum anteroposterior dimension at a point about halfway between the base and apex. The apex of the plate is located in line with the centre of the plate base in lateral view. There is some evidence for the presence of finger-like processes around the dorsal margin. The plate is uniformly thin transversely.

#### Plate 8 (Fig 80C and 80D)

Pl8 is deformed, with the dorsal and anterior margins being warped relative to the central part of the plate. The anteroventral and posteroventral margins of the plate are damaged and small sections of both are missing. The original outline of the plate is difficult to determine due to broken margins but as preserved it has a sub-pentagonal outline. The plate is taller than long and the maximum anteroposterior dimension is positioned at a point about halfway up the plate. The tallest point of the plate is situated just posterior to the anteroposterior midpoint of the element. The base is poorly preserved but is transversely expanded relative to the rest of the plate. Surface preservation is poor but there are some indications of narrow vascular grooves extending dorsoventrally across the plate surface.

#### Plate 9 (Fig 80E and 80F)

Pl9 is sub-rhomboidal in outline but is reconstructed along its anterodorsal margin and the surface is covered in plaster. It has been slightly deformed so that the right lateral surface is gently concave whereas the left lateral surface is convex. The anterior margin is anteriorly convex, sloping anterodorsally from the base, then posterodorsally towards the apex of the plate. The plate apex is positioned posterior to the anteroposterior midpoint of the plate base. The plate is taller than it is long. The posterior margin of the plate descends more steeply than the anterior margin and is constricted in its ventral-most part as it merges into the basal surface. The maximum anteroposterior dimension is in the lower half of the plate just below mid-length, in contrast to the condition in preceding plates, where the maximum anteroposterior dimension is at mid-length. The dorsal margin of the plate is divided into a number of slender finger-like processes. The plate is transversely compressed and thickest ventrally at its base. The basal region bears a number of low rugosities, is irregular, and is indistinctly defined. There are a number of fine elongate vascular grooves on the surface that are poorly preserved.

#### Plate 10 (Fig 81A and 81B)

Pl10 is rhomboidal in outline, with straight anteroventral, anterodorsal, posterodorsal and posteroventral margins that are separated from each other by a distinct break in slope. The area just dorsal to the junction between the anterodorsal and anteroventral margins is reconstructed, as the entire posterodorsal margin. Deformation has resulted in the left lateral surface being anteroposteriorly convex, while the right lateral surface is correspondingly concave. The dorsal part of the right lateral surface has been restored extensively with plaster. The maximum anteroposterior dimension of the plate is situated in its ventral half below mid-height, as in Pl9. The apex of the plate is situated above the anteroposterior midpoint of the plate basal margin, and the plate is sub-equally expanded anteriorly and posteriorly either side of this axis. The basal region is substantially transversely thicker than in any of the preceding plates and is rugose and irregular. It is transversely thickest in the centre of the plate and tapers anteriorly and posteriorly, merging with the plate margins. There are abundant dorsoventrally extending vascular grooves and the dorsal margin bears numerous finger-like processes both anterior and posterior to the plate apex.

#### Plate 11 (Fig 81C and 81D)

Pl11 is sub-rhomboidal in outline. It is missing a small portion of its anteroventral surface and part of the posterodorsal region is reconstructed. The body of the plate is slightly deformed and warped, particularly posteroventrally. The anterior margin of the plate is smoothly convex in its ventral-most quarter but the slope changes abruptly to extend posterodorsally toward the apex of the plate. This anterodorsal margin is subdivided into a large number of finger-like processes of varying size, giving it a feathered appearance. The apex of the plate lies dorsal to the posterior margin of the basal region so that it is strongly asymmetrical in lateral view. The posterior margin of the plate is gently convex along its entire length, although the dorsal-most part immediately adjacent to the apex is also subdivided into delicate finger-like processes. The basal margin is straight, rugose, clearly defined, and is the transversely widest part of the plate. The plate surface is well preserved and there are numerous sub-parallel, dorsoventrally-extending vascular grooves on its surface. The anteroposteriorly longest dimension is situated at a point within the basal third of the plate in lateral view. The maximum anteroposterior and dorsoventral lengths of the plate are sub-equal.

#### Plate 12 (Fig 82A and 82B)

Pl12 is very similar in overall shape to Pl10. The anterior margin is partially reconstructed, as are sections of the dorsal part of the plate, and much of the posterior margin is damaged. The central region of the plate has been damaged and warped. The apex of the plate lies posterior to the posterior margin of the basal region. The maximum anteroposterior dimension is in the lower third of the plate. The anterodorsal and dorsal-most part of the posterodorsal margins are subdivided into numerous finger-like processes. The plate is taller than long as preserved.

#### Plate 13 (Fig 82C and 82D)

Pl13 is the largest in the series but its anterior margin is missing and a considerable part is reconstructed in plaster. As a result it has an elongate elliptical outline, although originally it was probably more similar in shape to Pl11 or 12. Deformation has resulted in the right lateral surface of the plate being convex anteroposteriorly, while the left lateral surface is concave. The posterior margin is complete and well preserved and from a point approximately one-third along this surface to the dorsal apex the plate is divided into numerous finger-like elongate processes giving it a strongly feathered appearance. These continue onto the anterodorsal surface but as this region is incomplete it is unclear how far they extended anteriorly. There are some grooves that are continuous with with the indentations below the finger-like processes but otherwise the surface of the plate is poorly preserved. The posterior margin is straight. The basal region, which comprises the basal one-fifth of the plate in lateral videw, is irregular and rugose. It may have been entirely embedded in the skin, so that the anteroposteriorly widest part of the plate would have projected immediately above the level of the skin in life.

#### Plate 14

This plate was not recovered with the specimen (see above) and a reconstruction has been positioned in its place within the mount.

#### Plate 15 (Fig 82E and 82F)

Pl15 is missing its posteroventral corner. As with many of the other plates, it is slightly deformed so that the right lateral surface is dorsoventrally and anteroposteriorly concave and the left lateral surface is correspondingly convex. It is very similar in overall outline and size to Pl12, with a steeply convex posterior margin and shallowly convex anterior margin, resulting in an asymmetrical outline with more expansion anteriorly than posteriorly. The anteroposteriorly longest dimension of the plate is situated in its ventral half. Fine, elongate, finger-like processes are present both anterior and posterior to the plate apex. The basal region is strongly rugose and expanded transversely. A few vascular grooves are present on the plate surface.

#### Plate 16 (Fig 83A and 83B)

Pl16 is reconstructed along its anterior and basal margins, and some reconstruction is also present posteriorly. As in the preceding plates, there is some deformation so that the plate is convex on the left lateral surface and concave on the right lateral surface. As reconstructed, the plate has a sub-triangular outline in lateral view with the apex directed posterodorsally. One or two deep vascular channels are present on the surface of the plate, and finger-like processes are present around the plate apex. The plate apex is situated in line with the posterior margin of the basal region but due to reconstruction of the anterior margin it is not clear where the level of the maximum anteroposterior length would have been situated.

#### Plate 17 (Fig 83C and 83D)

Pl17 is reconstructed posteroventrally, including its posterior margin, the ventral half of the posterior part of the plate and the entire basal region. As a result its dimensions cannot be accurately estimated. However, it is clearly smaller than the preceding plates, and part of a decreasing size trend along the remainder of the tail. The anterior margin of the plate is almost straight, while the dorsal margin is steeply convex. Six stout, finger-like processes are present around the plate apex, and these are more robust than any of those in the preceding plates. There is faint evidence for shallow grooves on the dorsal part of the plate. The plate is asymmetrical so that its apex lies in line with the posterior margin of the basal region, as reconstructed.

#### Plate 18 (Fig 83E and 83F)

Pl18 is sub-elliptical in outline, although its ventral margin has been reconstructed. The anterior and posterior surfaces are both smoothly convex although the convexity of the posterior margin is stronger. This results in the posterior margin of the plate strongly overhanging the posterior margin of the basal region. The plate is longer than tall. The maximum anteroposterior dimension of the plate is situated at approximately mid-height. The left lateral surface is convex anteroposteriorly while its right lateral surface is concave, and this appears to be the natural condition. The tip of the plate has several short grooves incipiently dividing the apical region into finger-like processes, although these are not well developed.

#### Plate 19 (Fig 83G and 83H)

Pl19 is incomplete and poorly preserved. It appears to be a small plate but it lacks its original margins. It has been extensively crushed and offers no useful anatomical details.

#### Spines (Fig 84)

All four spines are present. They are well-preserved, although heavily consolidated, with surfaces lightly skimmed with plaster. They are undeformed and appear to be missing only their distal-most tips.

#### Anterior spine pair (Fig 84A and 84B)

In lateral, anterior and posterior views, the margins of the spine are straight and converge dorsally to form a narrow pointed tip. In lateral view, the basal margin is asymmetrical so that it extends further anteriorly than posteriorly. With the flat basal region held horizontally, the spine extends posterodorsolaterally at an angle of 70 degrees to the horizontal. The anterior margin bears a distinct ridge that extends dorsally from the basal region to a point approximately one-half of the length of the spine. This acts as a break in slope to divide the spine into lateral and medial surfaces. The lateral surface is convex in its lower half, whereas the medial surface is straight, giving it a D-shaped transverse cross-section at this point. The transverse cross-section in the dorsal half of the spine becomes more symmetrical and is elliptical. In ventral view, the basal region is elliptical in outline with its long axis trending anteroposteriorly. The surface of the basal region is rugose and gently convex in its posterior part.

#### Posterior spine pair (Fig 84C and 84D)

The posterior spines are slightly shorter than their anterior counterparts. In anterior, posterior and lateral views, the margins of the spine are essentially straight and converge dorsally to meet in a sharp pointed tip. In contrast to the anterior pair of spines, the basal region is not expanded anteriorly, but is of the same dimensions as the ventral part of the spine. Assuming the basal region is held horizontally, the spine angles at approximately 80 degrees from the horizontal to extend posterodorsolaterally. Both spines are slightly crushed, but probably maintained an ovate cross-section throughout their entire lengths. In ventral view, the basal region of the plate has an ovate outline, with its apex pointing anteriorly. The basal surface is rugose and generally flat anteriorly, but merges seamlessly with a convex posterior region, which grades into the posterior surface of the spine without a distinct break in slope.

#### Comparisons with the dermal armor of other stegosaurs

The dermal armor of *Stegosaurus homheni* (IVPP V4006) has been illustrated as a series of long, low plates. However, the single plate associated with the specimen is broken dorsally, so that the shape of the plate is unknown. It is, however, elongate, being 58 cm anteroposteriorly at its base, and transversely compressed, similar to the plates of NHMUK PV R36730 and other individuals of *Stegosaurus* (e.g. USNM 4934). The holotype of *S*. *stenops* ([2]; USNM 4934) and DNMH 2818 [46] each possess a group of small pebble-like osteoderms in the throat region, but these are absent in NHMUK PV R36730 and *S*. *mjosi* [17]. Currently, is unclear if the presence/absence of these osteoderms is due to taphonomic factors or individual, sexual, ontogenetic or taxonomic variation.

Thirteen dermal plates, from the cervical and anterior dorsal region, were recovered with *Dacentrurus* sp. (ML 433; [25]). In contrast to the situation in NHMUK PV R36730, there appears to be little differentiation between the plates associated with the cervical and dorsal vertebrae; they are all uniformly transversely thin, small, roughly triangular in outline, and bear a hook-like process anteriorly. Furthermore, in ML 433 the plates are clearly paired, whereas they are staggered in NHMUK PV R36730 and other individuals of *Stegosaurus*.

A single partial plate and a tail spine are preserved in *Dacentrurus armatus* (NHMUK OR46013; [29]: fig 10D, E). The plate is transversely thicker than those of NHMUK PV R36730 and is relatively small, whereas the spine is similar to the posterior spine pair in that it is not transversely expanded at the base and has a sub-rounded cross-section.

Several partial plates are preserved in the holotype of *Loricatosaurus* (NHMUK PV R3167; [29]: fig 18G–L) and all are transversely compressed, as in NHMUK PV R36730 and other specimens of *Stegosaurus*. However, a referred specimen of *Loricatosaurus* (MHNH(BR) 001; [32]: fig 2G–K) possesses a spine that projects at a very small angle from a broad, flat base. In stegosaurs, this type of spine is considered to have been located on the shoulder because a pair of spines with this morphology was discovered in place alongside the scapulae of *Gigantspinosaurus* [37, 47]; thus, these spines are known as ‘parascapular’ spines. No parascapular spine has ever been found in *Stegosaurus*.

The dermal armor of *Kentrosaurus* (MB R.4830–R.4843; [36]: pls 1, 4) comprises a series of transversely compressed or rounded spines and a parascapular spine, and is quite different in morphology to that of NHMUK PV R36730 and other individuals of *Stegosaurus*.

The armor of *Chungkingosaurus* (CV 206; [35]: fig 96) comprises small plates that are transversely thickened, particularly in the middle region of each plate. In *Huayangosaurus*, there are two plate morphotypes. One type, probably from the cervical and anterior dorsal region, comprises transversely compressed, small plates, while the second type comprises transversely flattened spines similar to those of *Kentrosaurus*. *Huayangosaurus* also possessed parascapular spines and small osteoderms over the rib cage [26].

## Ontogeny, Sex and Death

Several postcranial features of NHMUK PV R36730 indicate that this individual was not fully skeletally mature at time of death. The presence of visible neurocentral sutures on many of the cervical vertebrae indicates that sutural fusion may have just occurred or was occurring at the time of death, but had not advanced to the stage where the sutures were completely obliterated [48]. Scapula and coracoid fusion appears to vary between *Stegosaurus* individuals [2] and may be under ontogenetic control [43]. In NHMUK PV R36730, the scapula and coracoid are fused on one side but appear to be unfused the other (though this is difficult to confirm due to the absence of the ‘unfused’ coracoid), perhaps suggesting that the individual was still growing when it died. The olecranon process of the ulna of NHMUK PV R36730 is relatively small: the development of a larger, more prominent process is thought to occur late in *Stegosaurus* development [43]. In NHMUK PV R36730, the femoral head is separated from the shaft by a gentle angle and the anterior trochanter of the femur is visible as a distinct, finger-like process. In the largest stegosaurian femora, by contrast, the femoral head is separated from the shaft by a sharper right-angled bend and the anterior trochanter is fully fused to the greater trochanter and is effectively obliterated [43]. These features, in combination with the fusion of the tibia, fibula and astragalus, indicate that NHMUK PV R36730 was not a juvenile, but probably a subadult or young adult [43].

Prior to its acquisition by the Natural History Museum, the limb bones of NHMUK PV R36730 were sampled histologically ([49]; NHMUK PV R36730 is listed therein as SMA RCR0603). This earlier study documented the presence of longitudinally orientated primary osteons and some secondary osteons within the bone cortex, and femoral cross-sections revealed five lines of arrested growth (LAGs) and the absence of an external fundamental system (EFS). Although [49] used their study to classify growth stages in *Stegosaurus*, their results should be treated with caution because the majority of specimens included in their growth series are referable to a different species of *Stegosaurus*, *S*. *mjosi* (SCRM pers. obs. 2009), and at least some individuals of that species are much smaller than those of *S*. *stenops* when fully skeletally mature (SCRM pers. obs. 2005–2009), a problem not considered by those authors. Growth stages of *Stegosaurus stenops* have been determined histologically elsewhere [50] and based on these results, the presence of LAGs in the femur of NHMUK PV R36730, and the absence of an external fundamental system, indicate that the specimen can be referred to their growth stage 3 (young adult). At this stage, growth had slowed and, by comparison with growth patterns in extant mammals, [50] infer that sexual maturity had been attained. Thus, NHMUK PV R36730 should be considered as a young adult individual that had attained sexual maturity at time of death. This is congruent with the overall body size of the individual: NHMUK PV R36730 is 5.5–6 m in length, whereas larger individuals of *Stegosaurus* (e.g. DMNH 2818, femoral length >1 m, in contrast the right femur of NHMUK PV R36730 is 86.8 cm long) show histological and morphological features consist with skeletal maturity (growth stage 4: [50]).

The sex of the individual is unknown, although the nicknames associated with the specimen during its collection and preparation (‘Sarah’ [24]) and acquisition and display (‘Sophie’) may have given the misleading impression that NHMUK PV R36730 is a female. However, there are, as yet, no reliable indicators for determining sex in *Stegosaurus*. Histological work on the specimen did not reveal the presence of medullary bone [49], so NHMUK PV R36730 was not an egg-laying female. However, the absence of this tissue could be taken as equal evidence that NHMUK PV R36730 was either male or a non-gravid female. [51] proposed a bimodal distribution of proximal femur shape in *Kentrosaurus* might represent a sexual difference, but it is also possible that this observation captures a population-level difference rather than sexual dimorphism [52].

Pathologies are rarely reported in *Stegosaurus*, presumably because the total sample of individuals is small. Nevertheless, several cases of broken armor elements have been reported [53,54] as well as histological evidence for osteomyelitis [53,55]. The cause of death for NHMUK PV R36730 is unknown: there is no direct evidence of predation or scavenging on the skeleton (i.e. no tooth marks or puncture wounds), there are no indications of any broken bones and there are no clear pathologies associated with the skeleton (with the possible exception of one badly preserved ungual, see above). Consequently, the most likely causes of death are either starvation or a disease that affected only the soft tissue systems (e.g. a respiratory or gastrointestinal illness).

## Supporting Information

S1 Fig 3D pdf containing a 3D model of all elements of the postcrania. (PDF)

## Abbreviations

AMNH: American Museum of Natural History, New York, U.S.A.
BYU: Brigham Young University, Salt Lake City, U.S.A.
CEUM: Prehistoric Museum, College of Eastern Utah, Price, U.S.A.
CM: Carnegie Museum, Pittsburgh, Philadelphia, U.S.A.
CV: Chongqing Museum, People’s Republic of China
DMNH: Denver Museum of Nature and Science, Colorado, U.S.A.
HMNH: Hayashibara Museum of Natural History, Okayama, Japan
IVPP: Institute of Vertebrate Paleontology and Paleoanthropology, Beijing, People’s Republic of China
LHNB: Laboratório de História Natural da Batalha, Portugal
MB: Museum für Naturkunde, Berlin, Germany
MHNH(BR): Muséum Histoire Naturelle du Havre, Brun Collection, Le Havre, France
ML: Museu da Lourinhã, Portugal
NHMUK: Natural History Museum, London, U.K.
SMA: Sauriermuseum, Aathal, Switzerland
USNM: National Museum of Natural History, Smithsonian Institution, Washington D.C., U.S.A
YPM: Peabody Museum, Yale University, New Haven, Connecticut, U.S.A.
ZDM: Zigong Dinosaur Museum, Sichuan, People’s Republic of China
