# Supplementary figures and images for: The Postcranial Skeleton of an Exceptionally Complete Individual of the Plated Dinosaur Stegosaurus stenops (Dinosauria: Thyreophora) from the Upper Jurassic Morrison Formation of Wyoming, U.S.A
Source: PLoS One. 2015 Oct 14;10(10):e0138352. doi: 10.1371/journal.pone.0138352 (PMC4605687; doi:10.1371/journal.pone.0138352)

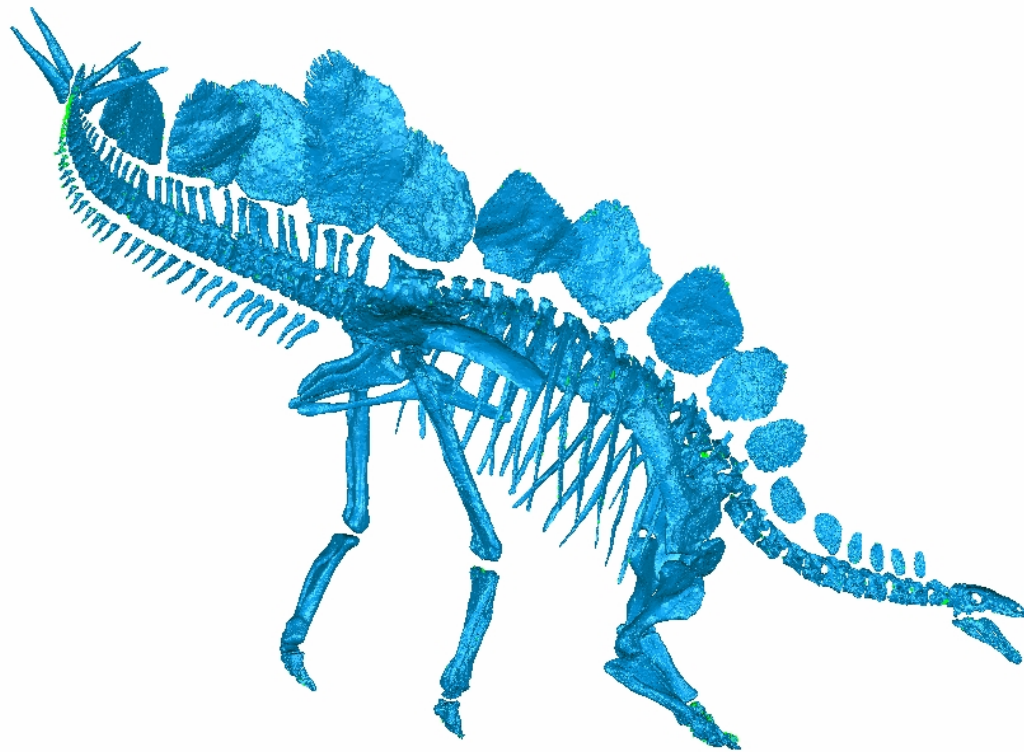

Click on the image to activate the 3D Model.

Supplement: S1 Fig — (PDF) [file pone.0138352.s001.pdf]
